# A look under the hood of genomic-estimated breed compositions for brangus cattle: What have we learned?

**DOI:** 10.3389/fgene.2023.1080279

**Published:** 2023-03-28

**Authors:** Zhi Li, Jun He, Fang Yang, Shishu Yin, Zhendong Gao, Wenwu Chen, Chuanyu Sun, Richard G. Tait, Stewart Bauck, Wei Guo, Xiao-Lin Wu

**Affiliations:** ^1^ College of Animal Science and Technology, Hunan Agricultural University, Changsha, Hunan, China; ^2^ Biostatistics and Bioinformatics, Neogen GeneSeek, Lincoln, NE, United States; ^3^ Department of Animal and Dairy Sciences, University of Wisconsin, Madison, WI, United States; ^4^ Council on Dairy Cattle Breeding, Bowie, MD, United States

**Keywords:** admixture model, ancestry, composite animals, genomic hitchhiking, linkage disequiblibrium, SNP

## Abstract

The Brangus cattle were developed to utilize the superior traits of Angus and Brahman cattle. Their genetic compositions are expected to be stabilized at 3/8 Brahman and 5/8 Angus. Previous studies have shown more than expected Angus lineage with Brangus cattle, and the reasons are yet to be investigated. In this study, we revisited the breed compositions for 3,605 Brangus cattle from three perspectives: genome-wise (GBC), per chromosomes (CBC), and per chromosome segments (SBC). The former (GBC) depicted an overall picture of the “mosaic” genome of the Brangus attributable to their ancestors, whereas the latter two criteria (CBC and SBC) corresponded to local ancestral contributions. The average GBC for the 3,605 Brangus cattle were 70.2% Angus and 29.8% Brahman. The K-means clustering supported the postulation of the mixture of 1/2 Ultrablack (UB) animals in Brangus. For the non-UB Brangus animals, the average GBC were estimated to be 67.4% Angus and 32.6% Brahman. The 95% confidence intervals of their overall GBC were 60.4%–73.5% Angus and 26.5%–39.6% Brahman. Possibly, genetic selection and drifting have resulted in an approximately 5% average deviation toward Angus lineage. The estimated ancestral contributions by chromosomes were heavily distributed toward Angus, with 27 chromosomes having an average Angus CBC greater than 62.5% but only two chromosomes (5 and 20) having Brahman CBC greater than 37.5%. The chromosomal regions with high Angus breed proportions were prevalent, tending to form larger blocks on most chromosomes. In contrast, chromosome segments with high Brahman breed proportion were relatively few and isolated, presenting only on seven chromosomes. Hence, genomic hitchhiking effects were strong where Angus favorable alleles resided but weak where Brahman favorable alleles were present. The functions of genes identified in the chromosomal regions with high ( ≥75% ) Angus compositions were diverse yet may were related to growth and body development. In contrast, the genes identified in the regions with high ( ≥37.5% ) Brahman compositions were primarily responsible for disease resistance. In conclusion, we have addressed the questions concerning the Brangus genetic make-ups. The results can help form a dynamic picture of the Brangus breed formation and the genomic reshaping.

## Introduction

Brangus beef cattle were developed to combine the desirable traits of Angus and Brahman cattle. Angus cattle are well known for their superior carcass qualities, and Angus cows have excellent fertility and milking capability. The Brahman cattle have developed disease resistance, overall hardiness, and outstanding maternal instincts thanks to rigorous natural selection. The crossbreeding to create the Brangus breed dated to 1932, according to the USDA 1935 Yearbook in Agriculture. Yet, Brangus registration by the International Brangus Breeders Association (IBBA) started in 1949. For official registration, a Brangus animal needs to be genetically stabilized at 3/8 Brahman and 5/8 Angus by pedigree, be solid black or red, and be polled (Briggs and Briggs, 1980). Both sire and dam must be recorded with IBBA (San Antonio, TX). Hence, knowing the breed compositions of individual animals is a requisite to official animal registrations. Such information also allows for utilizing the “stable” heterosis to explore methods for predicting heterosis (Akanno et al., 2017), and it permits the implementation of precise animal farming management decisions (Berry, 2019).

After the breed formation, subsequent *inter-se* mating and selection have been conducted with Brangus over time. For example, the United States IBBA has developed expected progeny differences (EPD) for quantitative traits, such as birth weight, weaning weight, yearling weight, milk production, total maternal calving ease, and intramuscular fat. Likely, artificial selection pressure employed at varying levels on these traits of interest in the past decades could have resulted, to some extent, in the deviation of the ancestral genomic proportions in Brangus from the previously targeted ratios. Previous studies showed significantly elevated Angus genomic breed composition (GBC) in Brangus cattle (He et al., 2018; Paim T. D. P. et al., 2020; Paim T. D. P. et al., 2020; Li et al., 2020; Wang et al., 2020; Wu et al., 2020), and the reasons are yet to be revealed. There were several plausible assumptions. For example, it was postulated that selecting Brangus for Angus favorable traits (e.g., carcass, growth, feed efficiency) could increase the genomic breed compositions of Brangus cattle toward Angus (Paim T. D. P. et al., 2020; Wu et al., 2020). In theory, the proportion of actual genotypes passed from one generation to the next can vary between individuals owing to Mendelian sampling, genetic recombination rate and linkage disequilibrium (LD) (Falconer and Mackay, 1996). Hence, selecting Brangus for traits more prevalent in Angus (e.g., carcass, growth, feed efficiency) can favor Angus alleles, sweeping more ‘Angus’ haplotypes to further generations. Another possible reason could be the mixture of 1/2 Ultrablack (UB) animals (i.e., the first-generation progenies derived from Brangus × Angus), which are expected to have 81.25% Angus lineage. In October 2005, the International Brangus Breeders Association (IBBA) board of directors approved the creation of the Ultrablack and Ultrared (UR) program to take advantage of the strengths of the Brangus and Angus or Red Angus breeds, which combine environmental adaptability and maternal excellence of Brangus with the exceptional marbling, calving ease and name recognition of Angus or Red Angus. The UB and UR animals are registered composite animals with a validated and documented lineage between 12.5% and 87.5% Brangus breeding. The remaining 87.5%–12.5% must be a registered Angus to be a UB or a Red Angus to be a UR. The second assumption is likely, yet scientifically supporting evidence is needed. Apart from the possible mixture, the actual genomic breed compositions of Brangus cattle are not known precisely after decades of crossbreeding and selection.

Selection may have left signatures on the genome after the breed formation (Goszczynski et al., 2017; Paim T. D. P. et al., 2020). These genomic regions with selection sweeps can have different breed compositions than expected due to the selective advantages of genes from one of the founders. Paim T. D. P. et al. (2020) evaluated the overall ancestral breed compositions and local ancestral contributions by chromosomes in Brangus cattle. They also related haplotypes to ancestral traits under selection. Such information could lead to a better understanding of how hybridization and crossbreeding systems have shaped the genetic architecture of these composite animals. However, their conclusions were built on small samples of the two founder breeds with genotypes (i.e., 68 Brahman and 95 Angus). Statistically, the inference of allelic frequencies and haplotypes, and, therefore, ancestry origins, are subject to large errors in small samples.

In this study, we revisited the estimation of breed compositions for Brangus cattle from three perspectives, genomic-wise, per chromosome, and per chromosome segment. The genotyped animals in the two ancestral breeds included 20,359 Angus and 509 Brahman cattle. Hence, our sample sizes for the two ancestral breeds were significantly larger than those used by Paim T. D. P. et al. (2020). We took a consistent approach to estimate ancestral breed compositions from the three perspectives; all were assessed with an admixture model. Three measures of breed compositions were defined: 1) genomic-estimated breed compositions (GBC), 2) chromosomal-estimated breed compositions (CBC), and 3) segmental-estimated breed compositions (SBC). Note that the latter two quantities corresponded to local ancestral genomic contributions, measured per chromosome and chromosomal regions, respectively. Possible population stratification was inferred based on global ancestral genomic proportions, whereas genomics dynamics due to crossbreeding and selection were visualized through local ancestral contributions.

## Materials and methods

### Animals and genotype data

The experimental data consisted of 3,605 Brangus cattle, 20,359 Angus cattle, and 483 Brahman cattle. The latter two ancestral breeds were used as the reference populations for estimating GBC for Brangus cattle. All the animals were genotyped with a GeneSeek Genomic Profiling (GGP) bovine 50 K V1 (version 1) chip, except 349 Brahman cattle were genotyped with an Illumina 777 K bovine SNP chip (Table 1). The genotypes were extracted from the Neogen GeneSeek genotyping databases representing samples shared between the Neogen global laboratories. The SNP map positions were based on the UMD 3.1 reference bovine genome assembly (Merchant et al., 2014).

**TABLE 1:** Descriptive statistics of genotype data for Brangus and their ancestral breeds (Angus and Brahman)^a^.

Type	Breed	Number of animals	Number of SNPs	Allele A frequency	
				Mean	SD
Composite	Brangus	3,605	49,463	0.477	0.231
Ancestry	Angus	20,359 (20,322)	49,463	0.492	0.247
	Brahman	349 (349)	777,962	0.439	0.343
		160 (134)	49,463	0.431	0.363

^a^ The numbers in the brackets are genotyped animals that remained after data cleaning.

Reference SNPs were selected from the common set between the GGP bovine 50 K V1 chip and the Illumina 777 K bovine SNP Beadchip. The data cleaning removed SNPs with a call rate of less than 95%, SNPs on the two sex chromosomes, and SNPs without map position. SNPs violating the Hardy-Weinberg equilibrium (*p* < 1.0E-8) were also excluded. For SNPs with greater than 0.99 correlations on each chromosome, only the one with higher or the highest minor allelic frequency was kept. When there were ties in allelic frequencies, a random one was taken. The final SNP set retained 41,672 common SNPs for the subsequent analyses. In each ancestral population, outlier individuals were excluded as those with (−2)log (likelihood) exceeding a given cutoff value (i.e., 2.0 by default) (He et al., 2018). This test excluded 37 Angus and 26 Brahman animals from the reference populations. The means and standard deviations of allele A frequencies of the SNPs after data cleaning in the three populations are shown in Table 1. On average, Angus cattle had a higher allele A frequency (0.492) than Brahman cattle (0.431–0.439). The average allele A frequency for Brangus cattle was 0.477, which fell between the two ancestral populations yet closer to Angus. The Angus population had a smaller standard deviation of allele A frequency than the Brahman population. The Brangus cattle had a smaller standard deviation of allele A frequencies than the two ancestral populations (Table 1).

### Estimation of breed compositions

Breed compositions were estimated for individual Brangus animals using the admixture model by Bansal and Libiger (2015) (BL-Admixture). This method utilizes the same form of likelihood model as in the STRUCTURE (Pritchard et al., 2000) and ADMIXTURE (Alexander et al., 2009) software packages. However, the BL-Admixture model runs faster. In particular, STRUCTURE takes a Bayesian approach and relies on a Markov Chain Monte Carlo (MCMC) algorithm to sample the posterior distribution, which can extremely computationally intensive with large data. STRUCTURE and Admixture represent unsupervised analysis of the ancestry of multiple individuals and jointly estimate allele frequencies for the ancestral populations and the relative contribution of each ancestral population to each individual’s genome. The BL-Admixture estimates the ancestry for a single individual using information about allele frequencies at a large number of loci for multiple reference populations. The latter allele frequencies are obtained from previous unsupervised admixture analysis, or simply from the reference population assuming no genetic drift and selection after these breeds were formed. We took the latter approach.

Consider one biallelic locus (say *j*) genotyped on an animal (say *i*). Assume that allele frequencies on this locus are known for each ancestry breed. The admixture model postulates that a progeny’s genotype at this locus is determined according to an allelic frequency as a weighted average of *T* reference (ancestry) breeds: $f_{ij}=\sum_{t=1}^{T}w_{it}q_{tj}$ , where $q_{tj}$ is the frequency of allele B for the *j*th SNP in the *t*th reference population,; $w_{it}$ is the corresponding weight. Then, under the assumption of Hardy-Weinberg equilibrium, the probabilities of observing each genotype ( $g_{ij}$ ) on this animal are the following:

$$\mathrm{Pr}\left(g_{ij}|f_{ij}\right)=\left\{\begin{array}{l} {\left(1-f_{ij}\right)}^{2}\;g_{ij}=0\\ 2f_{ij}\left(1-f_{ij}\right)\;g_{ij}=1\\ f_{ij}^{2}\;g_{ij}=2 \end{array}\right.$$ (1)

Now, consider j=1,…,M SNPs genotyped on this animal, assuming their mutual independence. The log-likelihood computed for all the *M* SNPs measured on the *i*th animal, denoted by $l_{i}$ , is as follows.

$$\begin{array}{c} l_{i}=\sum_{j=1}^{M}\ln\left(\mathrm{Pr}\left(g_{ij}|f_{ij}\right)\right)\\ =\left[\sum_{j=1}^{M}g_{ij}\ln\left(f_{ij}\right)+\left(2-g_{ij}\right)\ln\left(1-f_{ij}\right)\right]+C \end{array}$$ (2)

where $C=\sum_{j=1}^{M}\ln\left(\begin{array}{c} 2\\ g_{ij} \end{array}\right)$ . Note that the assumption of mutual independence between SNPs does not hold precisely due to linkage or/and random associations between them. Nevertheless, the admixture model is often robust to this assumption violation because the percentage of SNPs in high LD are rare when using moderate to high-density SNP panels (He et al., 2018; Li et al., 2020).

The admixture coefficients, $W_{i}={\left(\begin{array}{ccc} w_{i1} & \ldots & w_{iT} \end{array}\right)}^{\prime }$ , taken to the GBC of animal *i* attributable to the reference (ancestral) breeds, are obtained using the Broyden-Fletcher-Goldfarb-Shanno (BFGS) method under the restrictions that $w_{ik}\geq 0$ ; $\sum_{k=1}^{T}w_{iT}=1$ (Nocedal and Wright, 2006). BFGS is a powerful, Quasi-Newton second derivative line search family method to solve non-linear optimization problems. Optimizing the likelihood function of BFGS iteratively removed the non-zero mixing coefficient, which did not significantly improve the model fitting, thus obtaining a concise set of individual mixture coefficients. In the admixture model, the value of each admixture coefficient is bounded between 0 and 1, and the sum of admixture coefficients (GBC) computed for each animal is one under the assumption of 100% genetic contributions by the *T* ancestral breeds to each animal.

Breed compositions for the Brangus animals were estimated genome-wide, per chromosome, and per chromosomal segment, respectively. The number of reference SNPs per chromosome varied from 712 (chromosome 25) to 2,568 (chromosome 1), and the average distance between SNPs ranged from 0.05 to 0.07 Mb. SBC were obtained on three window sizes: 1 Mb, 5 Mb, and 10 Mb, respectively. The widow sizes were taken arbitrarily yet still based on two factors, the average length of gene in the bovine genome and the minimum number of SNPs to give stable estimates. To minimize the errors in the estimated SBC due to insufficient SNP coverage, chromosomal segments with less than five SNPs were excluded from computing SBC. There were 2,522 1-Mb segments, 518 5-Mb segments, and 269 10-Mb segments, respectively. The average number of SNPs per segment ranged from 15.2 to 18.8 (1 Mb), 72.0 to 91.1 (5 Mb), and 132.0 to 169.1 (10 Mb), respectively (Table 2). Overall, the average *R* ^2^ LD per segment decreased as the window size increased, ranging from 0.107 to 0.164 on 1 Mb chromosomal regions, from 0.062 to 0.104 on 5-Mb chromosomal regions, and from 0.050 to 0.081 on 10 Mb chromosomal regions (Table 2). The *R* ^2^ LD on 1-Mb regions also had a larger average standard deviation (0.05) than those on 5-Mb and 10-Mb regions (0.02).

**TABLE 2:** Mean and standard deviation (SD) of *R* ^2^ linkage disequilibrium (LD) among SNPs evaluated by varying window sizes (1 Mb, 5 Mb, and 10 Mb) on each chromosome^a^.

Chromosome	Window = 1 Mb			Window = 5 Mb			Window = 10 Mb		
	N	Mean	SD	N	Mean	SD	N	Mean	SD
1	159	0.144	0.044	32	0.089	0.016	16	0.068	0.014
2	137	0.137	0.042	28	0.087	0.015	14	0.066	0.010
3	122	0.155	0.052	25	0.095	0.029	13	0.073	0.025
4	121	0.159	0.054	25	0.101	0.028	13	0.081	0.026
5	122	0.135	0.049	25	0.088	0.025	13	0.068	0.019
6	120	0.127	0.051	24	0.073	0.020	12	0.055	0.016
7	113	0.150	0.070	23	0.090	0.028	12	0.069	0.021
8	114	0.153	0.062	23	0.093	0.028	12	0.072	0.021
9	106	0.129	0.049	22	0.083	0.023	11	0.064	0.014
10	105	0.131	0.052	21	0.080	0.015	11	0.063	0.014
11	108	0.148	0.049	22	0.086	0.020	11	0.064	0.015
12	91	0.136	0.050	19	0.088	0.024	10	0.070	0.019
13	85	0.156	0.057	17	0.097	0.031	9	0.071	0.017
14	84	0.164	0.063	17	0.099	0.029	9	0.070	0.013
15	86	0.157	0.055	18	0.104	0.038	9	0.073	0.016
16	82	0.150	0.078	17	0.091	0.039	9	0.069	0.021
17	75	0.120	0.042	15	0.066	0.007	8	0.051	0.010
18	66	0.145	0.047	14	0.082	0.017	7	0.058	0.005
19	64	0.126	0.038	13	0.077	0.013	7	0.059	0.009
20	72	0.125	0.045	15	0.083	0.030	8	0.073	0.043
21	72	0.146	0.090	15	0.086	0.026	8	0.066	0.018
22	62	0.129	0.033	13	0.078	0.016	7	0.060	0.016
23	53	0.130	0.083	11	0.077	0.015	6	0.061	0.021
24	63	0.138	0.051	13	0.083	0.012	7	0.065	0.019
25	43	0.107	0.038	9	0.062	0.013	5	0.050	0.018
26	52	0.146	0.043	11	0.091	0.016	6	0.072	0.022
27	46	0.129	0.041	10	0.084	0.043	5	0.056	0.017
28	47	0.113	0.036	10	0.074	0.021	5	0.053	0.010
29	52	0.122	0.046	11	0.079	0.026	6	0.069	0.039

^a^ N = number of segments with valid SNP, genotypes on a chromosome.

### Clustering of brangus cattle

K-means clustering (Jain, 2010) was conducted on the GBC for the 3,605 Brangus cattle to reveal possible population stratifications, where *K* = 2 and 3, respectively. Initially, the number of clusters *K* was specified *a priori*, and randomly assigned all the animals to each of *K* distinct, non-overlapping clusters as their initial clusters. Then, the *K*-means algorithm computed the cluster centroid for each cluster, which is a vector of genotype means for the *M* reference SNPs, and it re-assigned each animal to the cluster whose centroid was the closest. The last two steps proceeded iteratively till the total within-clustering variation, defined by the sum of all the pairwise squared Euclidean distance in each cluster and summed over all the *K* clusters, was minimized as much as possible (Hartigan and Wong, 1979):

$$minimize\left\{\sum_{k=1}^{K}\frac{1}{n_{k}}\underset{i,i^{\prime }\in C_{k}}{\sum }\sum_{j=1}^{M}{\left(g_{ij}-g_{i^{\prime }j}\right)}^{2}\right\}$$

where $C_{k}$ stands of cluster *k*, and $n_{k}$ is the number of animals in the *k*th cluster. The K-mean clustering analysis was implemented by the “stats” R package.

### Gene set enrichment analysis

Gene ontology (GO) term enrichment analysis, or gene set enrichment analysis, was conducted using the “gprofiler” R package with genes identified in 1-Mb chromosomal regions featuring either ancestral breed. This R package performed functional gene enrichment analysis on the input gene lists, mapped genes to known functional resources, and detected statistically significantly enriched terms. Briefly, chromosomal segments satisfying Angus SBC ≥0.75 or Brahman SBC ≥0.50 were extracted. Both cutoff thresholds represented equal upward GBC deviations (i.e., 12.5%) from their expected values. Then, the gene information was extracted by querying in the Ensembl database according to their chromosomal locations on the selected chromosomal segments. Finally, GO clustering analysis was performed on the gene list generated with the different filters. The annotation databases included Gene Ontology–biological processes, cellular components, molecular function (http://geneontollogy.org/) (Ashburner et al., 2000), and Kyoto Encyclopedia of Genes and Genomes (KEGG; http://www.genome. jp/kegg/) (Kanehisa et al., 2016). Other relevant R packages used in this study included “org. Bt.e.g., db”, “biomaRt” (Smedley et al., 2015), and “clusterProfiler” (Yu et al., 2012). Gene lists were extracted using the “getBM” function in the “biomaRt” R package. Briefly, given a set of filters (e.g., the chromosome number, the start and end positions), it retrieved attributes of all genes in this interval from the BioMart database. The attributes information includes “ensembl gene id”, “chromosome number”, “gene start position”, “gene end position”, and “gene description”, etc. Finally, gene extraction was performed on all eligible SBC fragments, and all extracted genes were merged to form a gene list after removing duplication. Enrichment analysis was then performed. All the QTLs were queried and aggregated in the QTLdb database (Release 49, https://www.animalgenome.org/cgi-bin/QTLdb/BT/index) (Hu et al., 2022).

## Results and discussion

### Genomic-estimated breed compositions

The estimated GBC for the 3,605 Brangus cattle, on average, were 70.2% Angus and 29.8% Brahman (Table 3), which significantly deviated from the officially expected values (i.e., 62.5% Angus and 37.5% Brahman) (*p* < 2.2e-16). The 95% confidence intervals were 61.1%–85.8% Angus and 14.2%–38.9% Brahman. There were 459 (12.7%) Brangus cattle with Angus GBC ≥80.0% and 19 (0.5%) Brangus cattle with Angus GBC ≥90.0%. Similarly, elevated Angus GBC for the Brangus animals were documented in some previous studies. For example, Paim T. D. P. et al. (2020) estimated that Brangus were 70.4% Angus and 29.6% Brahman based on high-density SNP genotypes (777,962 SNP, BovineHD Beadchip, Illumina, San Diego, CA, United States). Li et al. (2020) showed that Brangus cattle was 69.8%–70.5% Angus and 29.5%–30.2% Brahman based on multiple models, including an admixture model, linear regression, and ridge-regression BLUP, each with a selectively uniform 20 K SNP panel. Wang et al. (2020) proposed using regularized admixture models to estimate GBC for purebred animals to deal with the so-called “Impure Purebred Paradox”, a phenomenon suggesting a higher-than-expected false-negative rate in the identification of purebred animals. They showed that Brangus were, on average, 71.1%–77.1% Angus and 22.9%–28.9% Braham based on various regularized admixture models. Using path analysis model, Wu et al. (2020) showed that Brangus cattle were 68.2%–71.8% Angus and 28.2%–31.8% Brahman. Hence, regardless of the population sizes and the statistical methods used, these studies have consistently pinpointed that significantly higher-than-expected Angus breed proportions in Brangus cattle.

**TABLE 3:** Means and standard deviations (SD) of genomic-estimated breed composition (GBC) for the 3,605 Brangus cattle as a single population (K = 1) and as subpopulations according to K-means clustering (K = 2 and 3, respectively)^1,2,3^.

K	Group	Number of animals	Angus-GBC %		Brahman %	
			Mean	SD	Mean	SD
1	A	3,605	70.22	6.75	29.78	6.75
2	B-1	713	81.63	4.66	18.37	4.66
	B-2	2,892	67.40	3.37	32.60	3.37
3	C-1	640	82.08	3.62	17.92	3.62
	C-2	381	70.93	4.03	29.07	4.03
	C-3	2,584	67.17	3.40	32.83	3.40

**FIGURE 1:**
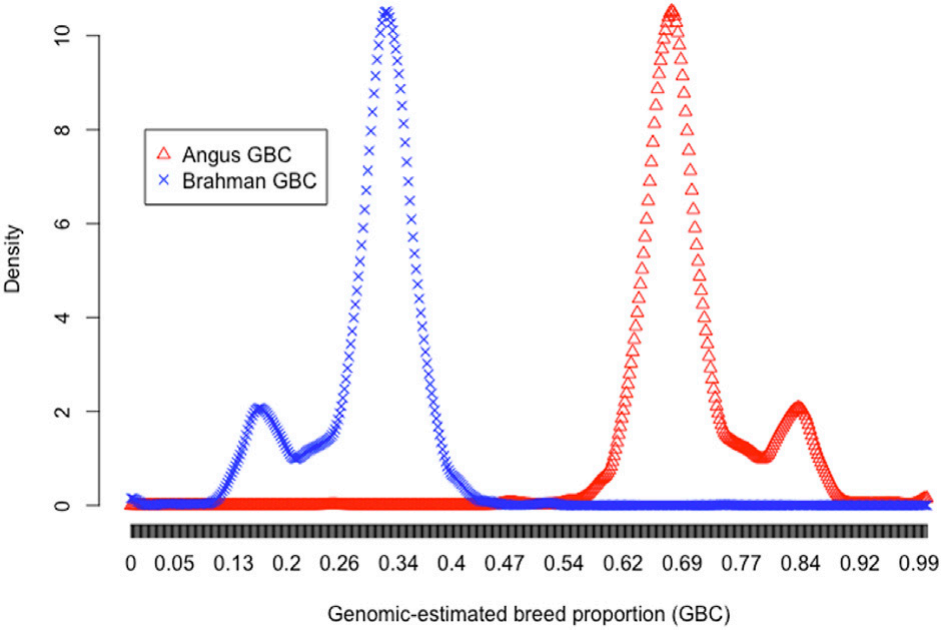
Density plots of genomic-estimated Angus (red triangle) and Brahman (blue cross) breed compositions for 3,605 Brangus cattle.

Multiple reasons are likely responsible for the elevated Angus lineage in Brangus. Firstly, population stratification could exist with Brangus, given the fact that the IBBA approved the creation of UB and UR animals in 2005. The density plots of the estimated Angus or Brahman breed proportions in the 3,605 animals were bimodal, which served as preliminary evidence for the Brangus population stratification (Figure 1). Then, *K*-means clustering was conducted to partition the 3,605 animals into two and three clusters, respectively (Table 3). With K = 2 (i.e., two clusters), the B-1 cluster with a higher average Angus GBC (81.6%) consisted of 713 (19.8%) Brangus cattle, whereas the B-2 cluster with a lower average Angus GBC (67.4%) included 2,892 (80.2%) animals. With *K* = 3 (i.e., three clusters), the cluster (C-1) with the highest average Angus GBC (82.1%) had 640 (17.8%) Brangus animals. The cluster with the lowest average Angus GBC (67.2%) included 2,584 (71.7%) Brangus animals. In both sets of clustering results (*K* = 2 *versus K* = 3), the average Angus GBC for animals in the top Angus composition clusters (B-1 *versus* C-1) agreed approximately with each other (81.6% *versus* 82.1%), and they corresponded roughly to the expected Angus breed proportion (81.25%) for the ½ UB animals. We thus suspected that these animals could be the ½ UB animals. Using a path analysis approach, Wu et al. (2020) confirmed that the ½ UB animals were, on average, 81.25% Angus and 18.75% Brahman. The path analysis decomposed the relationships between the ancestors and the composite animals into direct and indirect path effects. The above percentage only accounted for direct effects by the path-analysis interpretation, assuming a zero correlation between the two ancestral breeds (Wu et al., 2020). In reality, however, Angus and Brahman cattle are connected due to sharing common remote ancestors, though the correlation can be low. For example, the correlation of allele A frequencies ranged from 0.05 to 0.10 between the two ancestral breeds, subject to the SNP panel sizes. If considering the indirect path effects, the actual Angus breed proportions for the ½ UB animals could be slightly higher. On the other hand, the two majority clusters, B-2 and C-3, had roughly comparable averages of Angus GBC (67.4% *versus* 67.2%). These were likely Brangus cattle without the mixture of ½ UB animals. The average Angus breed proportion for non-UB Brangus was 67.4% based on the clustering analysis with *K* = 2. The 95% confidence interval of Angus breed proportions in Brangus was between 60.4% and 73.5%. Hence, there was, on average, an approximately 5% deviation of GBC toward Angus breed proportions in non-UB Brangus cattle, possibly resulting from selecting Brangus cattle for phenotypes where Angus has advantages. Without selection, genomic-estimated breed compositions would agree approximately with the expected ratios (e.g., Funkhouser et al., 2017; Gobena et al., 2018). Note that ½ UB animals are not precisely registered Brangus. They are officially given a “UB” prefix for registration purposes and are shown on their registered IDs. It is also worth mentioning that the B-2 (or C-3) cluster could include some advanced UB crosses because they had comparable Angus breed proportions as those in the non-UB Brangus cattle in clusters B-2 and C-3. In early 2013, the IBBA further approved the breeding-up of UB (or UR) cattle to registered Brangus. The rule states that an IBBA-registered Brangus sire or dam mated to an IBBA-registered UB or UR sire or dam that results in at least 7/8th (87.5%) Brangus also qualifies as a registered Brangus. For example, mating a ¾ UB animal (i.e., progenies derived from crossing ½ UB animals with Brangus) to a registered Brangus resulted in 7/8 UB animals, which meets the 87.5% Brangus makeup. On average, a 7/8 UB animal was 67.2% Angus. Finally, the average Angus GBC for the animals in cluster C2 was 70.9%, which roughly corresponded to the expected Angus lineage (71.9%) for ¾ UB animals.

### Chromosomal-estimated breed compositions

The ancestral breed proportions were estimated by chromosomes in the 3,605 Brangus (Table 4). Overall, the average estimated CBC per chromosome varied substantially, from 56.9% (chromosome 5) to 79.8% (chromosome 15). Converse to the Angus CBC, the average Brahman CBC was the lowest on chromosome 15 (20.2%) and the highest on chromosome 5 (CBC 43.1%). We noted that the estimated ancestral breed proportions were heavily distributed toward Angus, which agreed with the breeding target for greater Angus “blood” than Brahman. There were 27 chromosomes with Angus CBC greater than 62.5%, but only two chromosomes (5 and 20) had Brahman CBC greater than 37.5%. Our results coincided with a previous study by Paim T. D. P. et al. (2020). They showed that chromosome 15 had the highest Angus proportion (84.7%), and chromosome five had the largest Brahman proportion (43.7%). Still, there were some differences. Paim T. D. P. et al. (2020) used principal component analysis to describe the population relationships. They showed that the first principal components (PC1), which accounted for ancestral breeds, were uniformly distributed between the two ancestral breeds when evaluated on chromosomes 16, 25, and 29. Because the average Brahman breed proportion is expected to be 5/8 (not 5/5), a uniform distribution of PC1 for Brangus between the two ancestral breeds would suggest equal ancestral genomic proportions. Hence, their results were an indication of significant deviates in breed compositions of Brangus toward Brahman on these three chromosomes. A principal analysis is a popular feature-reducing technique for analyzing large, high-dimension data, yet ignoring the detailed information of individual features, which were local ancestral contributions on specific chromosomal regions. In the present study, we directly evaluated breed compositions by chromosomes. Our results showed that only chromosomes 5 and 20 had less than 62.5% Angus breed proportions on average, which agreed with Paim T. D. P. et al. (2020). But all the other chromosomes (including 16, 25, and 29) had an average Angus CBC greater than 62.5%, meaning they deviated toward Angus instead. These differences could also arise from sampling biases, because the two ancestral breeds used by Paim T. D. P. et al. (2020) were small. There was a high chance that the sampled allelic frequencies may deviate substantially from the actual allelic frequencies. Furthermore, because the variance of an allelic frequency is inversely proportional to the population size (Falconer and Mackay, 1996), the estimated allelic frequencies for ancestral breeds were also subject to large deviations in small samples.

**TABLE 4:** Means and standard deviations (SD) of chromosomal-estimated breed compositions (CBC) for the 3,605 Brangus cattle.

Chromosome	Angus-CBC %		Brahman-CBC %		Number of SNPs	Average spacing, Mb
	Mean	SD	Mean	SD		
1	72.31	12.28	27.69	12.28	2,568	0.06
2	70.19	14.10	29.81	14.10	2,218	0.06
3	74.67	12.51	25.33	12.51	2,081	0.06
4	75.67	12.24	24.33	12.24	1,889	0.06
5	56.86	15.91	43.14	15.91	2,120	0.06
6	69.7	14.18	30.3	14.18	1,988	0.06
7	67.66	15.17	32.34	15.17	1,815	0.06
8	70.08	14.26	29.92	14.26	1,774	0.06
9	65.94	15.57	34.06	15.57	1,830	0.06
10	73.77	12.89	26.23	12.89	1,697	0.06
11	72.27	13.50	27.73	13.50	1,709	0.06
12	67.47	16.30	32.53	16.30	1,419	0.06
13	65.13	16.82	34.87	16.82	1,446	0.06
14	73.58	15.22	26.42	15.22	1,405	0.06
15	79.84	12.89	20.16	12.89	1,371	0.06
16	64.74	18.31	35.26	18.31	1,334	0.06
17	79.21	13.09	20.79	13.09	1,214	0.06
18	73.86	13.07	26.14	13.07	1,152	0.06
19	71.51	15.47	28.49	15.47	1,184	0.05
20	59.76	16.22	40.24	16.22	1,352	0.05
21	62.58	16.53	37.42	16.53	1,215	0.06
22	78.17	13.51	21.83	13.51	1,002	0.06
23	72.09	14.81	27.91	14.81	953	0.06
24	72.97	14.72	27.03	14.72	1,045	0.06
25	73.33	17.64	26.67	17.64	712	0.06
26	78.30	15.38	21.70	15.38	862	0.06
27	78.66	15.64	21.34	15.64	736	0.06
28	67.99	17.40	32.01	17.40	789	0.06
29	68.77	18.53	31.23	18.53	792	0.07
Average	70.93	14.97	29.07	14.97	1,437	0.06

Despite the large chromosome-by-chromosome variability for ancestral breed proportions, GBC estimated by the mean of the average CBC across the 29 chromosomes per animal (denoted by C_GBC) agreed roughly with the average GBC in the 3,605 Brangus cattle. The mean (standard deviation) of Angus C_GBC for the 3,605 Brangus cattle was 70.9% (6.7%). The mean (standard deviation) of Brahman C-GBC was 29.1% (6.7%). The correlation between C_GBC and GBC for the 3,605 Brangus animals was 0.99 (See Supplementary Figure S1A). Within each subpopulation, the average CBC approximately agreed to (or slightly larger than) the corresponding GBC on average. For example, in the two clusters obtained by the K-means clustering with K = 2, the cluster for the mixed UB animals had, on average, 82.5% Angus and 17.5% Brahman breed compositions, and the cluster for non-UB animals had, on average, 68.2% Angus and 31.8% Brahman breed compositions.

### Segmental-estimated breed compositions

**FIGURE 2:**
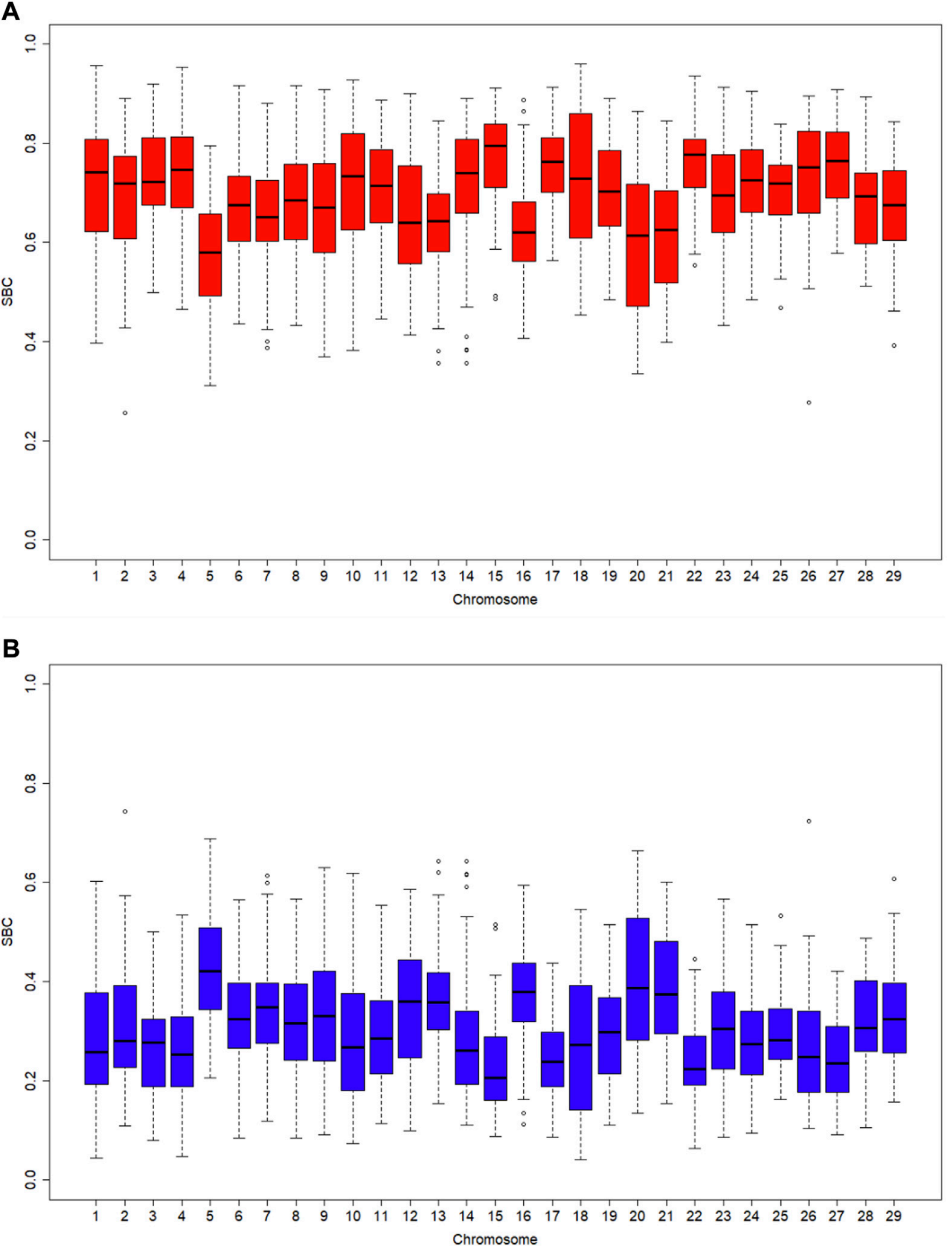
Boxplots of segmental-estimated breed compositions (SBC) for 3,605 Brangus cattle with the window size being 1 Mb on each chromosome: **(A)** Angus breed proportion; **(B)** Brahman breed proportion.

**FIGURE 3:**
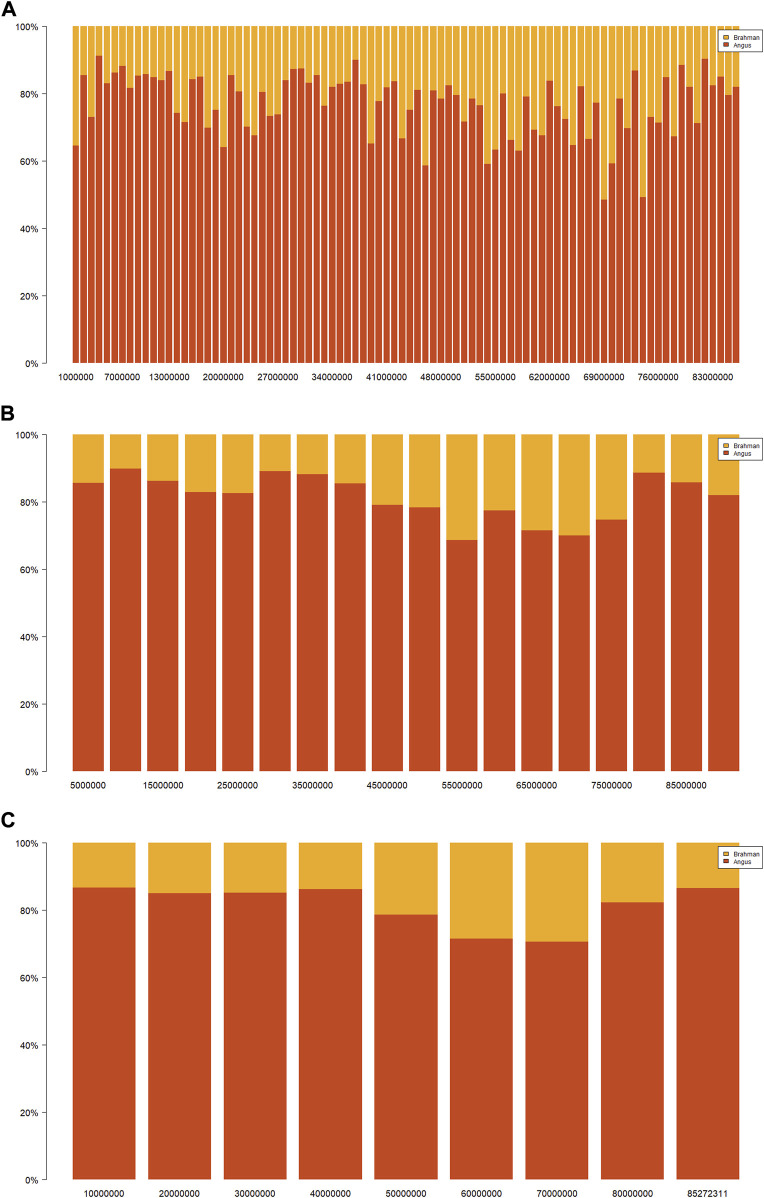
Distributions of Angus (red) or Brahman (yellow) breed proportions by varying window sizes on chromosome 15 in 3,605 Brangus cattle: **(A)** 1 Mb, **(B)** 5 Mb, and **(C)** 10 Mb. The black dashed line represents the population mean of the estimated genomic-estimated breed compositions (GBC), and the blue dashed line represents the population mean of the chromosomal-estimated breed compositions (CBC).

Ancestral breed compositions were evaluated on 1-Mb, 5-Mb, and 10-Mb windows on each chromosome (Table 5). Like CBC, the mean and range of the estimated SBC per segment varied substantially with chromosomes (Figure 2). Regardless of the window sizes, all the chromosomes, except 5 and 20, had the mean Angus SBC per chromosome exceeding 62.5%, and only chromosomes 5 and 20 had greater than 37.5% Brahman SBC. The average Angus SBC was the largest on chromosome 15 (76.9%–81.4%) and the smallest on chromosome 5 (57.6%–59.7%) (Table 5). Complementary to the Angus SBC, the Brahman SBC was the largest on chromosome 5 (40.3%–42.4%) and the smallest on chromosome 15 (18.6%–23.1%) (Table 5). Nevertheless, the segment with the maximum Angus proportion appeared on chromosome 18 (94.0%–95.9%), although chromosome 15 had the largest average Angus proportion. The maximum Brahman proportion segment was found on chromosome 2 (74.4%) when evaluated on 1 M windows and chromosome 5 (62.2%–64.4%) on 5-Mb and 10-Mb windows. The overall average of Angus SBC across the 29 chromosomes in the 3,605 Brangus cattle were 69.2% (1-Mb windows), 72.3% (5-Mb windows), and 72.0% (10-Mb windows), respectively. The overall average of Brahman SBC across the 29 chromosomes was 30.8% (1-Mb windows), 27.7% (5-Mb windows), and 28.0% (10-Mb windows), respectively. These overall averages of genomic breed compositions by averaging Angus and Brahman SBC across all 29 chromosomes per animal (denoted by S_GBC) roughly agreed with the average Angus and Brahman CBC (70.9% Angus *versus* 29.1% Brahman; Table 4) and the average GBC (70.2% Angus *versus* 29.8% Brahman; Table 3). The correlation between Angus S_GBC and GBC for the 3,605 Brangus animals was greater than 0.99, regardless of the window sizes (See Supplementary Figures S1B–D). Overall, the variation was larger when evaluated on a smaller interval than a larger interval (Figure 3). The Angus SBC on chromosome 15 varied between 68.6% and 89.7% when assessed with a 5-Mb window size (Table 5). The range became smaller (70.5%–86.6%) when evaluated with a larger window size (i.e., 10-Mb), and the range became larger (48.6%–91.1%) when assessed with a smaller window size (e.g., 1-Mb). Similar trends generally held on all the chromosomes (Table 5). The Brahman SBC showed precisely opposite trends.

**TABLE 5:** Minimum (min), maximum (max), and mean of segmental-estimated breed compositions (SBC) per chromosome for Brangus.

Chrom	Window = 1 Mb						Window = 5 Mb						Window = 10 Mb					
	SBC-Angus (%)			SBC-Brahman (%)			SBC-Angus (%)			SBC-Brahman (%)			SBC-Angus (%)			SBC-Brahman (%)		
	Min	Max	Mean	Min	Max	Mean	Min	Max	Mean	Min	Max	Mean	Min	Max	Mean	Min	Max	Mean
1	39.8	95.6	71.5	4.4	60.2	28.5	52.2	95.4	75.3	4.6	47.8	24.7	53.4	92.6	74.5	7.4	46.6	25.5
2	25.6	89.1	68.1	10.9	74.4	31.9	47.0	87.4	72.2	12.6	53.0	27.8	53.3	82.8	71.9	17.2	46.7	28.1
3	49.9	92.0	73.4	8.0	50.1	26.6	51.6	90.9	75.3	9.1	48.4	24.8	51.6	88.9	74.0	11.1	48.4	26.0
4	46.5	95.3	73.9	4.7	53.5	26.1	62.7	88.5	76.1	11.5	37.3	23.9	65.1	86.1	75.5	13.9	34.9	24.5
5	31.1	79.4	57.6	20.6	68.9	42.4	35.6	80.9	59.2	19.1	64.4	40.8	37.9	80.9	59.7	19.1	62.2	40.3
6	43.5	91.6	67.0	8.4	56.5	33.0	52.4	87.7	69.5	12.3	47.6	30.5	55.5	82.4	69.4	17.6	44.5	30.6
7	38.7	88.1	65.9	11.9	61.3	34.1	51.0	86.0	69.0	14.1	49.0	31.0	58.2	84.4	68.7	15.6	41.8	31.3
8	43.3	91.6	68.6	8.4	56.7	31.4	57.9	83.2	70.6	16.8	42.2	29.4	60.7	79.9	70.0	20.1	39.3	30.0
9	37.0	90.9	66.3	9.2	63.0	33.7	49.8	89.2	69.6	10.8	50.3	30.4	49.5	86.9	69.1	13.1	50.5	30.9
10	38.2	92.7	71.9	7.3	61.8	28.1	51.4	92.3	75.5	7.7	48.6	24.5	59.2	90.1	75.0	9.9	40.8	25.0
11	44.6	88.7	70.7	11.3	55.4	29.3	58.8	87.6	73.1	12.4	41.2	26.9	61.5	87.4	72.6	12.6	38.5	27.4
12	41.3	90.0	65.5	10.0	58.7	34.5	48.8	86.1	69.1	13.9	51.2	31.0	50.3	86.1	70.1	13.9	49.7	29.9
13	35.7	84.5	64.0	15.5	64.3	36.0	54.0	81.4	65.2	18.6	46.1	34.8	53.9	74.0	65.6	26.0	46.1	34.5
14	35.7	89.0	71.2	11.0	64.3	28.8	53.5	84.6	74.7	15.4	46.5	25.3	54.1	83.4	74.9	16.6	45.9	25.1
15	48.6	91.1	76.9	8.9	51.5	23.1	68.6	89.7	81.4	10.3	31.4	18.6	70.5	86.6	81.4	13.4	29.5	18.7
16	40.6	88.8	63.1	11.3	59.4	37.0	54.7	81.3	65.2	18.7	45.3	34.8	54.9	72.8	64.7	27.3	45.2	35.3
17	56.3	91.3	75.6	8.7	43.7	24.4	70.8	88.4	80.9	11.6	29.2	19.1	75.3	88.1	80.7	12.0	24.8	19.3
18	45.4	95.9	72.5	4.1	54.7	27.5	47.0	95.6	74.4	4.4	53.1	25.6	51.7	94.0	74.4	6.0	48.3	25.6
19	48.5	89.0	70.8	11.0	51.5	29.2	62.7	82.8	72.1	17.2	37.3	27.9	65.0	82.5	71.4	17.5	35.0	28.6
20	33.6	86.5	59.8	13.5	66.5	40.2	41.4	76.9	61.8	23.1	58.7	38.2	48.2	75.5	61.4	24.6	51.9	38.6
21	39.9	84.5	61.5	15.5	60.1	38.5	42.1	84.7	64.6	15.3	57.9	35.4	51.5	75.4	63.5	24.6	48.5	36.5
22	55.4	93.6	76.0	6.4	44.6	24.0	69.8	87.8	79.4	12.2	30.2	20.6	72.5	85.2	79.3	14.8	27.5	20.7
23	43.3	91.3	69.8	8.7	56.7	30.2	61.3	89.3	73.5	10.7	38.7	26.6	63.8	85.4	74.7	14.6	36.2	25.3
24	48.5	90.5	72.4	9.5	51.6	27.6	60.5	87.4	74.0	12.6	39.5	26.1	60.8	81.4	73.1	18.7	39.2	27.0
25	46.8	83.8	69.9	16.2	53.2	30.1	56.6	85.7	74.0	14.3	43.4	26.0	56.6	80.8	71.9	19.2	43.4	28.1
26	27.7	89.6	73.2	10.4	72.3	26.8	69.4	89.6	81.0	10.4	30.6	19.0	73.4	87.7	80.8	12.3	26.6	19.2
27	57.9	90.9	75.9	9.1	42.1	24.1	71.7	88.9	81.2	11.2	28.3	18.8	72.7	87.2	81.0	12.8	27.3	19.0
28	51.3	89.4	67.1	10.6	48.7	32.9	60.0	81.6	69.7	18.4	40.0	30.3	63.5	75.0	69.5	25.0	36.5	30.5
29	39.2	84.3	67.3	15.7	60.8	32.7	57.6	84.0	69.6	16.0	42.4	30.4	57.1	78.6	68.6	21.4	42.9	31.4
Overall	25.6	95.9	69.2	4.1	74.4	30.8	35.6	95.6	72.3	4.4	64.4	27.7	37.9	94.0	72.0	6.0	62.2	28.0

Ancestral breed proportions per segment varied considerably on each chromosome (Supplementary Figure S2). For example, the Brahman SBC on chromosome one were 4.4%–60.2% (1-Mb windows), 4.6%–47.8% (5-Mb windows), and 7.4%–46.6% (10-Mb windows), respectively (Table 5). There were segments with high Angus proportions and high Brahman proportions, respectively, on the chromosomes. For example, the average Angus SBC in 1–5 Mb and 6–10 Mb segments on chromosome 15 were 85.5% and 89.7%, respectively, which were substantially higher than the CBC for that chromosome. The average Angus SBC computed in the 40–75 Mb segment was between 68.6% and 79.1%, still higher than the officially expected Angus proportion (62.5%). Approximately 95% of the 2,522 1-Mb chromosomal segments had, on average, between 45.0% and 90.0% Angus breed proportions and between 10.0% and 65.0% Brahman breed proportions (Table 6). Likewise, approximately 90% of the total 5-Mb (or 10-Mb) chromosomal segments had between 50.0% and 90.0% Angus breed proportions and between 10.0% and 65.0% Brahman breed proportions (Table 6).

**TABLE 6:** Distributions of segmental-estimated Angus and Brahman breed compositions (Angus-SBC and Brahman-SBC) with the segments defined by 1 MB, 5 Mb, and 10 Mb intervals, respectively.

Range	Angus-SBC						Brahman-SBC					
	1 Mb		5 Mb		10 Mb		1 Mb		5 Mb		10 Mb	
	n	n%	n	n%	n	n%	n	n%	n	n%	n	n%
1	0	0	0	0	0	0	0	0	0	0	0	0
(1, 0.95]	5	0.2	2	0.4	0	0	0	0	0	0	0	0
(0.95, 0.9]	38	1.5	7	1.3	4	1.5	0	0	0	0	0	0
(0.9, 0.85]	139	5.5	62	11.9	23	8.6	0	0	0	0	0	0
(0.85, 0.8]	301	11.9	69	13.3	39	14.5	0	0	0	0	0	0
(0.8, 0.75]	369	14.6	78	15.0	41	15.2	0	0	0	0	0	0
(0.75, 0.7]	395	15.7	76	14.6	45	16.7	2	0.1	0	0	0	0
(0.7, 0.65]	377	14.9	89	17.1	47	17.5	4	0.2	0	0	0	0
(0.65, 0.6]	331	13.1	67	12.9	33	12.3	18	0.7	1	0.2	1	0.4
(0.6, 0.55]	238	9.4	32	6.2	18	6.7	54	2.1	5	1.0	0	0
(0.55, 0.5]	160	6.3	25	4.8	14	5.2	91	3.6	6	1.2	4	1.5
(0.5, 0.45]	91	3.6	6	1.2	4	1.5	160	6.3	25	4.8	14	5.2
(0.45, 0.4]	54	2.1	5	1.0	0	0	238	9.4	32	6.2	18	6.7
(0.4, 0.35]	18	0.7	1	0.2	1	0.4	331	13.1	67	12.9	33	12.3
(0.35, 0.3]	4	0.2	0	0	0	0	377	14.9	89	17.1	47	17.5
(0.3, 0.25]	2	0.1	0	0	0	0	395	15.7	76	14.6	45	16.7
(0.25, 0.2]	0	0	0	0	0	0	369	14.6	78	15.0	41	15.2
(0.2, 0.15]	0	0	0	0	0	0	301	11.9	69	13.3	39	14.5
(0.15, 0.1]	0	0	0	0	0	0	139	5.5	62	11.9	23	8.6
(0.1, 0.05]	0	0	0	0	0	0	38	1.5	7	1.3	4	1.5
(0.05, 0]	0	0	0	0	0	0	5	0.2	2	0.4	0	0
SUM	2,522	100	519	100	269	100	2,522	100	519	100	269	100

The variability of ancestral breed compositions per chromosome or segment could be a direct effect of selection. For example, Goszczynski et al. (2017) showed increased indicine haplotypes in the bovine leucocyte antigen region of Brangus cattle raised in Argentina, potentially due to selection for adaptation to the environments. In a brief search of bovine QTL in the QTLdb database, we found two postnatal growth traits and one body mass QTL reported on these two chromosomes, which were located on chromosome 15 at 61.6 Mb (body weight gain) and 17.0 Mb (birth body weight) (Snelling et al., 2010), and chromosome 17 at 12.0 Mb (average daily gain) (Rolf et al., 2012). These two chromosomes had the highest Angus CBC (chromosome 15: 79.84%; chromosome 17: 79.21%). These QTL were also located in regions with high Angus proportions. There are QTLs associated with disease resistance on chromosome 5 (Machado et al., 2010), which had the highest Braham CBC (43.14%). There were also health-related QTLs on chromosome 5, which included infectious bovine keratoconjunctivitis susceptibility (Kizilkaya et al., 2013), cold tolerance (Howard et al., 2014), and immune capacity (Leach et al., 2010), to list a few of them. All these QTLs were located in regions with high Brahman proportions in chromosome 5. There was a pleiotropic QTL affecting birth, yearling, and mature weights on chromosome 20 at 7–8 Mb (Weng et al., 2016), which was a region with a high Angus proportion. However, this chromosome had a high Brahman proportion (40.2%) in our study and Paim T. D. P. et al. (2020). The presence of Brahman favorable alleles in chromosomal regions with high Angus breed proportions, or *vice versa*, exemplifies the successful complementary of favorable alleles for the traits of interest from both ancestral breeds.

### Gene set enrichment analysis

**FIGURE 4:**
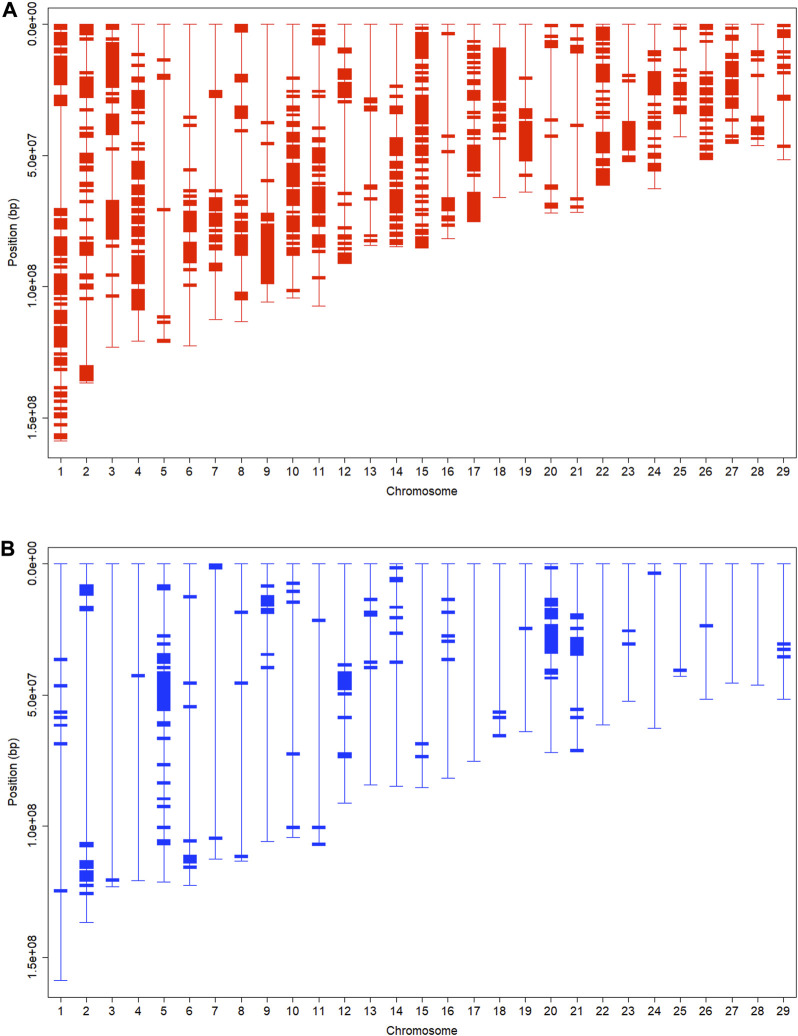
Chromosomal fragments with: **(A)** high ( ≥75% ) Angus breed proportion (upper), and **(B)** high ( ≥50% ) Brahman breed proportions (bottom), evaluated with a 1 Mb window size on 29 chromosomes in 3,605 Brangus cattle.

Gene set enrichment analysis was conducted with functional genes and QTL extracted on chromosome segments with high ( ≥75% ) Angus and high ( ≥50% ) Brahman breed proportions, respectively, evaluated on 1-Mb chromosomal intervals (Figure 4). We adopted these two cutoff threshold values because each represented an equal (12.5%) upward deviation from their expected values. We computed SBC on 1-Mb intervals for the subsequent gene set enrichment analysis because we rendered it an appropriate window size to capture gene regions of interest. Based on the *Bos taurus* UMD 3.1.1 assembly, the genome size of domestic cattle is approximately 2,670 Mb, which contains 26,815 genes (https://www.ncbi.nlm.nih.gov/genome/annotation_euk/Bos_taurus/105/#FeatureCountsStats). Hence, the average spacing between neighboring genes is around 0.1 Mb. In contrast, the chromosome regions defined on 5-Mb or 10-Mb intervals could be too broad. Nevertheless, SNPs on 1 Mb intervals had higher LD than those on 5-Mb or 10-Mb intervals. The average *R* ^2^ LD per segment on each chromosome ranged from 0.11 to 0.16 in 1-Mb intervals, from 0.06 to 0.10 in 5-Mb intervals, and from 0.05 to 0.08 in 10-Mb intervals (Table 2). When computing the likelihood, we noted that SNPs with higher LD tended to give more weight to these highly linked SNPs or their regions.

There were 852 segments with Angus SBC ≥75% and 169 segments with Brahman SBC ≥50%, which accounted for 33.8% and 6.7%, respectively, of the 2,522 1-Mb chromosomal segments on the genome (Table 7.). The total length of chromosomal segments with ≥ 75% Angus proportions was the longest on chromosome one and the shortest on chromosomes 5 and 13. Relatively speaking, chromosome 15 had the largest percentage (64.5%) of high Angus breed proportion segments, and Chromosome five had the least percentage (5.8%) of total high Angus breed proportion segments. Twenty-five chromosomes had more than 20% of the chromosome length as high ( ≥75% ) Angus proportion regions. In contrast, only two chromosomes (5 and 20) had more than 20% of their total length as high ( ≥50% ) Brahman breed proportions. Adjacent high Angus proportion segments often joined together to form larger blocks, presenting almost in all 29 chromosomes (Figure 4A). In contrast, high Brahman proportion segments were relatively sparse and isolated, though some formed small blocks, and they were present only on seven (2, 5, 6, 9, 10, 20, and 21) chromosomes (Figure 4B). These results agreed with a previous report yet with a different approach. Using a chromosome painting approach based on a copying model (Lawson et al., 2012), Paim T. D. P. et al. (2020) showed that chromosomal regions with high Angus breed proportions were prevalent on chromosomes, often large blocks, yet chromosome segments with high Brahman breed proportion were fewer and isolated. A follow-up study on selection signatures in Brangus revealed that the majority of selection signatures in Brangus cattle came from Angus (Paim T. D. P. et al., 2020). The copying model related the patterns of LD across chromosomes to the underlying recombination process and used a hidden Markov method to reconstruct a sampled haplotype. In the present study, we directly estimated breed compositions on each chromosome flanked by varying window sizes based on a mixture model. The admixture coefficients inferred from the admixture model can be probabilistically interpreted as reflecting that as identity-in-state (Jannink and Wu, 2003). Yet, when confined to long chromosomal chunks, it approximated the probability of identity by descent (Browning, 2008). Hence, though using a different approach, we came to similar findings.

**TABLE 7:** Summary of chromosomal segments with high Angus breed proportions (Angus SBC ≥ 75%) and high Brahman breed proportions (Brahman SBC ≥ 50%), respectively.

Chromosome	Total length	Angus SBC ≥ 75%			Brahman SBC ≥ 50%		
		N	Length	Percent (%)	N	Length	Percent (%)
1	158855123	79	79,000,000	49.7	7	7,000,000	4.4
2	136769635	46	46,000,000	33.6	17	17,000,000	12.4
3	123148964	50	50,000,000	40.6	1	1,000,000	0.8
4	120635950	60	60,000,000	49.7	1	1,000,000	0.8
5	121183174	7	7,000,000	5.8	34	34,000,000	28.1
6	122509741	25	25,000,000	20.4	8	8,000,000	6.5
7	112628884	23	23,000,000	20.4	3	3,000,000	2.7
8	113367096	32	32,000,000	28.2	3	3,000,000	2.6
9	105695468	29	29,000,000	27.4	9	9,000,000	8.5
10	104301732	46	46,000,000	44.1	5	5,000,000	4.8
11	107274061	42	42,000,000	39.2	3	3,000,000	2.8
12	91131021	25	25,000,000	27.4	12	12,000,000	13.2
13	84229982	9	9,000,000	10.7	5	5,000,000	5.9
14	84628243	37	37,000,000	43.7	7	7,000,000	8.3
15	85272311	55	55,000,000	64.5	2	2,000,000	2.3
16	81701834	11	11,000,000	13.5	5	5,000,000	6.1
17	75132928	41	41,000,000	54.6	0	0	0.0
18	65999195	30	30,000,000	45.5	3	3,000,000	4.5
19	64007021	21	21,000,000	32.8	1	1,000,000	1.6
20	71992748	11	11,000,000	15.3	22	22,000,000	30.6
21	71573501	8	8,000,000	11.2	13	13,000,000	18.2
22	61379134	38	38,000,000	61.9	0	0	0.0
23	52465632	16	16,000,000	30.5	2	2,000,000	3.8
24	62685898	25	25,000,000	39.9	1	1,000,000	1.6
25	42851121	13	13,000,000	30.3	1	1,000,000	2.3
26	51663776	26	26,000,000	50.3	1	1,000,000	1.9
27	45388171	25	25,000,000	55.1	0	0	0.0
28	46248750	10	10,000,000	21.6	0	0	0.0
29	51484561	12	12,000,000	23.3	3	3,000,000	5.8

In theory, crossing breaks and shuffles chromosomes randomly over time when directional selection is absent, which reflects genetic drift. However, with selection, it increases and even fixes favorable alleles. Meanwhile, it also changes the allelic frequencies of genes in LD with the genes under selection due to genomic hitchhiking, also known as genetic draft (Smith and Haigh, 1974; Ma et al., 2019). In other words, genomic hitchhiking occurs when a polymorphic locus is in LD with a second locus undergoing a selective sweep. As a result, the linked allele will also increase in frequency, in some cases, until it becomes fixed in the population. Overall, genomic hitchhiking reduces genetic variation and leaves footprints across the genome known as the signatures of selection (Sabeti et al., 2007; Singh et al., 2020). The many high Angus breed proportion regions reflected the presence of multiple favorable alleles scattered across the chromosomes. We observed the prevalence of large blocks with high Angus breed proportions (Figure 4A), possibly resulting from the genomic hitchhiking effects when selecting Brangus for Angus favorable traits. This is equivalent to saying that genomic hitchhiking effects were strong around the genomic regions with Angus favorable alleles, leading to the presence of large blocks with high Angus breed proportions. In contrast, genomic hitchhiking effects were weak around Brahman favorable alleles because chromosomal blocks with high Brahman breed proportions were relatively few, isolated, and small in size.

There were 9,025 genes on the chromosomal segments with Angus SBC ≥75% (Supplementary Table S1) and 1,877 genes on the chromosomal segments with Brahman SBC ≥50% (Supplementary Table S2). Many genes in high (≥75%) Angus regions are responsible for biological processes related to animal development, such as regulation of biological processes, anatomical structure development, anatomical structural morphogenesis, animal organ development, and skeletal system development, and, in KEGG, related to hormone regulation (such as the Estrogen signaling pathway). For example, the system development (GO:0048731) category’s related child terms include system development, such as central nervous, respiratory, and endocrine. We also found many genes associated with carcass and meat quality traits, such as *PPP1R3B* (Edwards et al., 2003; Cinar et al., 2012), *ASXL1* (Grigoletto et al., 2020), *DNMT3B* (Liu et al., 2012), and *TMEM68* (Lindholm-Perry et al., 2012; Terakado et al., 2018; Edea et al., 2020), just to list a few. Our gene list also included LEP on chromosome four and PLAG1 on chromosome 14. Paim T. D. P. et al. (2020) previously found these two genes in high Angus regions. The LEP gene is expressed in adipose tissue and codes for leptin, a hormone known to regulate feed intake and energy balance in mammals (Woronuk et al., 2012). This gene is associated with marbling, fat thickness, rib eye area, and feed intake in several beef cattle breeds (Souza et al., 2010; Woronuk et al., 2012; Kononoff et al., 2017). Leptin is an essential gene for puberty onset (Williams et al., 2002). This gene could be inherited from Angus ancestors, or its frequency was increased by the selection of Brangus for early puberty since breed formation because *Bos indicus* heifers have challenges achieving puberty early in life (Sartori et al., 2010; Fortes et al., 2012). PLAG1 is involved in regulating stature and weight (Littlejohn et al., 2012; Pryce et al., 2012; Song et al., 2016). This gene is associated with yearling weight in Australian Tropical Composite breeds (Porto-Neto et al., 2014). There is still another gene, XKR4, which is close to PLAG1. The XKR4 gene is associated with subcutaneous rump fat thickness, scrotal circumference, serum concentration of prolactin, and sexual precocity (Fortes et al., 2012; Porto Neto et al., 2012; Bastin et al., 2014; Takada et al., 2018). The genes in the high ( ≥50% ) Brahman regions are primarily responsible for biosynthetic-related biological processes (e.g., cytolysis), molecular biological functions related to enzyme activity (e.g., lysozyme activity, peptidoglycan muralytic activity, hydrolase activity, and serine-type endopeptidase activity), and diseases and immunity (e.g., MHC class II protein complex, MHC protein complex, type I diabetes mellitus, allograft rejection, graft-versus-host disease, autoimmune thyroid disease, and pathogenic *Escherichia coli* infection). For example, peptidoglycan muralytic activity (GO:0061783), which contributes to the degradation of peptidoglycan, is a major structural component of bacterial cell walls (Nelson et al., 2012); Another example is the MHC Class II protein complex (GO:0042613). MHC is involved in the immune process and plays the role of transmitting antigens (Rodgers and Cook, 2005). Still, the gene functionalities in chromosomal regions with high Angus breed proportions were diverse, possibly due to genomic hitchhiking of genes linked to the favorable alleles under selection. For example, there was a category of genes called “cellular response to stress”. Cells respond to stress in various ways, from activating pathways that promote survival to eliciting programmed cell death that eliminates damaged cells. Cell death research has attracted much attention in the last 2 decades also because of its relevance to development, degenerative diseases, and cancer in human (Fulda et al., 2010). Overall, the Brangus cattle have successfully combined the favorable traits of the two highly successful parent breeds.

QTLs were extracted from the chromosomal regions with the top 1% highest Angus (Angus SBC ≥91.1% ) and Brahman (Braham SBC ≥59.4% ) breed proportions, respectively (Supplementary Table S3). The QTLs in the top 1% Angus regions are related to meat, carcass, production, and reproduction. This list included, for example, birth weight QTL (chromosomes three and 8), weaning weight QTL (chromosomes 1, 3, 15, 18, and 23), yearling weight QTL (chromosomes 3 and 10), mature body weight QTL (chromosomes 3, 4, 10, 17, and 18), carcass weight QTL (chromosomes three and 8), marbling score QTL (chromosomes 15, 18, and 22), fat thickness QTL (chromosomes 3, 4, 17, 18, and 23), calving ease QTL (chromosomes 3, 8, 10, and 17), and QTL for Longissimus muscle area (chromosomes 3, 8, 17, and 18). Some QTL have pleiotropic effects. The QTL in the regions with 1% highest Brahman proportions are related to health, such as bovine respiratory disease susceptibility (chromosome 2: 117.3–134.4 Mb), insulin-like growth factor 1 level on (chromosome 5: 40.3–59.7 Mb; chromosome 14: 26.5–27.0 Mb), and blood cortisol level (chromosome 16: 29.7 Mb).

## Conclusions

We have revisited the genomic breed compositions in 3,605 Brangus cattle from three perspectives, genome-wise, per chromosome, and per chromosome segment. The K-means clustering analysis revealed population stratification. The B-1 cluster consisted of ½ UB (i.e., first generation from crossing Brangus with Angus), with 81.6% Angus genomic lineage. The non-UB Brangus animals (B-2), on average, were 67.4% Angus and 32.6% Brahman. It was likely that selecting Brangus for traits where Angus had advantages led to an approximately 5% average deviation in the estimated GBC toward Angus lineage. Further deviation of Brangus breed compositions from the officially expected ancestral breed ratios is worth paying attention to because it could diminish the complementarity of Brahman and Angus over time.

The ancestral breed proportions varied substantially by chromosomes and by chromosomal segments, likely shaped by cross-breeding and subsequent inter-se mating and selection over time. There were strong hitchhiking effects on genomic regions where favorable Angus alleles resided. The functions of genes identified in the chromosomal regions with high Angus proportions were diverse, yet many were responsible for biological processes related to growth and body development. The genes identified in the regions with high Brahman compositions were primarily responsible for biosynthetic-related processes, molecular biological functions related to enzyme activity, diseases, and immunity. The co-presence of segments of high-Angus and high-Brahman lineage, respectively, on the same chromosomes, exemplified the successful complementary of favorable alleles from both ancestral breeds.

While genomic-estimated breed compositions depicted an overall picture of the “mosaic” genome of animals attributable to their ancestors, local ancestry genomic compositions were visualized through chromosomal- and segmental-estimated breed compositions. Precisely, the admixture coefficients based on the admixture model inferred the probability of alleles identical in state when estimating the ancestral breed compositions for individual animals. Nevertheless, they corresponded approximately to the probability of alleles identical by descent when confined to long chromosome chunks. We noted that alternative approaches existed, such as path analysis (Wu et al., 2020) and the breed-of-origin (BOA) approach (Calus et al., 2022), that could better handle the situation when ancestral breeds were correlated. In the present study, however, the correlation between Angus and Brahman was low (0.05–0.10). Hence, we used the admixture model because is theoretically instructive and easily implemented in practice.

## Data Availability

All data were taken from the databases of the Neogen Global Laboratories. Example data and programs are available at: https://doi.org/10.6084/m9.figshare.22303009.v1. Inquiries can be directed to the corresponding author.
