# Supplementary material for: A look under the hood of genomic-estimated breed compositions for brangus cattle: What have we learned?
Source: Front Genet. 2023 Mar 28;14:1080279. doi: 10.3389/fgene.2023.1080279 (PMC10086375; doi:10.3389/fgene.2023.1080279)
Supplement: Supplementary file 1 [file Table1.docx]

**Table S1.** Annotation enrichment analysis of functional genes in chromosome segments with high ($\geq75\%$) Angus breed proportions ^1,2^

| Term name | Term ID | P | -log10(P) | N_term | N_query | N_inter | N_domain |
| --- | --- | --- | --- | --- | --- | --- | --- |
| olfactory receptor activity | GO:0004984 | 1.46E-07 | 6.83 | 1042 | 4968 | 378 | 17921 |
| identical protein binding | GO:0042802 | 7.69E-06 | 5.11 | 1466 | 4968 | 499 | 17921 |
| protein binding | GO:0005515 | 2.26E-05 | 4.65 | 7343 | 4968 | 2193 | 17921 |
| molecular transducer activity | GO:0060089 | 7.89E-05 | 4.10 | 1966 | 4968 | 642 | 17921 |
| signaling receptor activity | GO:0038023 | 7.89E-05 | 4.10 | 1966 | 4968 | 642 | 17921 |
| transmembrane signaling receptor activity | GO:0004888 | 9.79E-05 | 4.01 | 1818 | 4968 | 597 | 17921 |
| G protein-coupled receptor activity | GO:0004930 | 1.82E-04 | 3.74 | 1456 | 4968 | 486 | 17921 |
| odorant binding | GO:0005549 | 1.48E-03 | 2.83 | 148 | 4968 | 67 | 17921 |
| calcium-dependent phospholipase A2 activity | GO:0047498 | 5.46E-03 | 2.26 | 16 | 4968 | 13 | 17921 |
| C-C chemokine binding | GO:0019957 | 1.73E-02 | 1.76 | 17 | 4968 | 13 | 17921 |
| chemokine receptor activity | GO:0004950 | 3.35E-02 | 1.47 | 24 | 4968 | 16 | 17921 |
| G protein-coupled chemoattractant receptor activity | GO:0001637 | 3.35E-02 | 1.47 | 24 | 4968 | 16 | 17921 |
| kinase binding | GO:0019900 | 4.09E-02 | 1.39 | 533 | 4968 | 187 | 17921 |
| protein homodimerization activity | GO:0042803 | 4.61E-02 | 1.34 | 518 | 4968 | 182 | 17921 |
| ion binding | GO:0043167 | 4.88E-02 | 1.31 | 4208 | 4968 | 1261 | 17921 |
| multicellular organismal process | GO:0032501 | 5.70E-17 | 16.24 | 5344 | 5184 | 1716 | 18965 |
| regulation of cellular process | GO:0050794 | 3.05E-13 | 12.52 | 9623 | 5184 | 2881 | 18965 |
| cellular response to stimulus | GO:0051716 | 4.70E-13 | 12.33 | 6359 | 5184 | 1975 | 18965 |
| developmental process | GO:0032502 | 4.81E-12 | 11.32 | 4362 | 5184 | 1397 | 18965 |
| anatomical structure development | GO:0048856 | 1.72E-11 | 10.76 | 4059 | 5184 | 1305 | 18965 |
| cell communication | GO:0007154 | 2.09E-11 | 10.68 | 5630 | 5184 | 1755 | 18965 |
| signaling | GO:0023052 | 3.69E-11 | 10.43 | 5581 | 5184 | 1739 | 18965 |
| biological regulation | GO:0065007 | 1.95E-10 | 9.71 | 10997 | 5184 | 3228 | 18965 |
| response to stimulus | GO:0050896 | 2.80E-09 | 8.55 | 7430 | 5184 | 2241 | 18965 |
| regulation of biological process | GO:0050789 | 5.73E-09 | 8.24 | 10390 | 5184 | 3050 | 18965 |
| signal transduction | GO:0007165 | 1.84E-08 | 7.73 | 5229 | 5184 | 1615 | 18965 |
| positive regulation of biological process | GO:0048518 | 1.29E-07 | 6.89 | 4642 | 5184 | 1440 | 18965 |
| multicellular organism development | GO:0007275 | 1.50E-07 | 6.83 | 3355 | 5184 | 1069 | 18965 |
| positive regulation of cellular process | GO:0048522 | 1.17E-06 | 5.93 | 4198 | 5184 | 1304 | 18965 |
| animal organ development | GO:0048513 | 1.43E-06 | 5.84 | 2439 | 5184 | 793 | 18965 |
| positive regulation of metabolic process | GO:0009893 | 1.94E-06 | 5.71 | 2838 | 5184 | 909 | 18965 |
| positive regulation of cellular metabolic process | GO:0031325 | 5.73E-06 | 5.24 | 2452 | 5184 | 792 | 18965 |
| system development | GO:0048731 | 1.35E-05 | 4.87 | 2980 | 5184 | 943 | 18965 |
| localization | GO:0051179 | 4.06E-05 | 4.39 | 4876 | 5184 | 1481 | 18965 |
| regulation of multicellular organismal process | GO:0051239 | 5.71E-05 | 4.24 | 1866 | 5184 | 611 | 18965 |
| G protein-coupled receptor signaling pathway | GO:0007186 | 8.10E-05 | 4.09 | 1732 | 5184 | 570 | 18965 |
| regulation of biological quality | GO:0065008 | 1.06E-04 | 3.98 | 2633 | 5184 | 834 | 18965 |
| positive regulation of macromolecule metabolic process | GO:0010604 | 1.28E-04 | 3.89 | 2615 | 5184 | 828 | 18965 |
| negative regulation of cellular component organization | GO:0051129 | 1.93E-04 | 3.71 | 476 | 5184 | 182 | 18965 |
| transport | GO:0006810 | 2.76E-04 | 3.56 | 3447 | 5184 | 1065 | 18965 |
| cellular developmental process | GO:0048869 | 3.35E-04 | 3.47 | 2832 | 5184 | 887 | 18965 |
| positive regulation of nitrogen compound metabolic process | GO:0051173 | 3.89E-04 | 3.41 | 2318 | 5184 | 737 | 18965 |
| cell differentiation | GO:0030154 | 4.92E-04 | 3.31 | 2810 | 5184 | 879 | 18965 |
| establishment of localization | GO:0051234 | 7.27E-04 | 3.14 | 3585 | 5184 | 1100 | 18965 |
| macromolecule localization | GO:0033036 | 1.26E-03 | 2.90 | 2050 | 5184 | 654 | 18965 |
| negative regulation of organelle organization | GO:0010639 | 1.59E-03 | 2.80 | 260 | 5184 | 107 | 18965 |
| anatomical structure morphogenesis | GO:0009653 | 1.60E-03 | 2.80 | 1961 | 5184 | 627 | 18965 |
| phosphate-containing compound metabolic process | GO:0006796 | 2.45E-03 | 2.61 | 2220 | 5184 | 701 | 18965 |
| positive regulation of biosynthetic process | GO:0009891 | 2.47E-03 | 2.61 | 1502 | 5184 | 490 | 18965 |
| phosphorus metabolic process | GO:0006793 | 2.78E-03 | 2.56 | 2239 | 5184 | 706 | 18965 |
| homeostatic process | GO:0042592 | 2.89E-03 | 2.54 | 1149 | 5184 | 384 | 18965 |
| positive regulation of cellular biosynthetic process | GO:0031328 | 3.34E-03 | 2.48 | 1482 | 5184 | 483 | 18965 |
| protein localization | GO:0008104 | 3.43E-03 | 2.46 | 1692 | 5184 | 545 | 18965 |
| system process | GO:0003008 | 3.56E-03 | 2.45 | 1645 | 5184 | 531 | 18965 |
| positive regulation of RNA metabolic process | GO:0051254 | 3.64E-03 | 2.44 | 1372 | 5184 | 450 | 18965 |
| positive regulation of nucleobase-containing compound metabolic process | GO:0045935 | 5.10E-03 | 2.29 | 1531 | 5184 | 496 | 18965 |
| skeletal system development | GO:0001501 | 5.32E-03 | 2.27 | 400 | 5184 | 151 | 18965 |
| regulation of transport | GO:0051049 | 6.26E-03 | 2.20 | 1074 | 5184 | 359 | 18965 |
| regulation of cellular component organization | GO:0051128 | 6.56E-03 | 2.18 | 1629 | 5184 | 524 | 18965 |
| catabolic process | GO:0009056 | 7.33E-03 | 2.13 | 1804 | 5184 | 575 | 18965 |
| regulation of localization | GO:0032879 | 7.67E-03 | 2.12 | 1890 | 5184 | 600 | 18965 |
| embryonic skeletal system morphogenesis | GO:0048704 | 7.80E-03 | 2.11 | 85 | 5184 | 43 | 18965 |
| cell cycle | GO:0007049 | 1.09E-02 | 1.96 | 1180 | 5184 | 389 | 18965 |
| microtubule-based movement | GO:0007018 | 1.16E-02 | 1.94 | 306 | 5184 | 119 | 18965 |
| positive regulation of macromolecule biosynthetic process | GO:0010557 | 1.21E-02 | 1.92 | 1420 | 5184 | 460 | 18965 |
| response to chemical | GO:0042221 | 1.45E-02 | 1.84 | 3168 | 5184 | 966 | 18965 |
| macromolecule modification | GO:0043412 | 1.58E-02 | 1.80 | 3124 | 5184 | 953 | 18965 |
| protein modification process | GO:0036211 | 2.18E-02 | 1.66 | 2962 | 5184 | 905 | 18965 |
| cellular protein modification process | GO:0006464 | 2.18E-02 | 1.66 | 2962 | 5184 | 905 | 18965 |
| cellular response to stress | GO:0033554 | 2.54E-02 | 1.59 | 1365 | 5184 | 441 | 18965 |
| cytoskeleton organization | GO:0007010 | 3.05E-02 | 1.52 | 1118 | 5184 | 367 | 18965 |
| intracellular signal transduction | GO:0035556 | 3.57E-02 | 1.45 | 1895 | 5184 | 595 | 18965 |
| embryonic skeletal system development | GO:0048706 | 3.58E-02 | 1.45 | 111 | 5184 | 51 | 18965 |
| regulation of organelle organization | GO:0033043 | 3.61E-02 | 1.44 | 860 | 5184 | 289 | 18965 |
| animal organ morphogenesis | GO:0009887 | 3.85E-02 | 1.41 | 765 | 5184 | 260 | 18965 |
| cytoplasm | GO:0005737 | 4.46E-11 | 10.35 | 8379 | 5154 | 2494 | 19011 |
| nucleoplasm | GO:0005654 | 4.32E-09 | 8.36 | 2996 | 5154 | 963 | 19011 |
| cytosol | GO:0005829 | 3.83E-07 | 6.42 | 2792 | 5154 | 888 | 19011 |
| membrane | GO:0016020 | 1.80E-06 | 5.74 | 7852 | 5154 | 2301 | 19011 |
| endomembrane system | GO:0012505 | 7.35E-05 | 4.13 | 2690 | 5154 | 838 | 19011 |
| cytoskeleton | GO:0005856 | 6.18E-04 | 3.21 | 1684 | 5154 | 538 | 19011 |
| cell periphery | GO:0071944 | 1.12E-03 | 2.95 | 4235 | 5154 | 1263 | 19011 |
| cell junction | GO:0030054 | 1.52E-03 | 2.82 | 1219 | 5154 | 398 | 19011 |
| intrinsic component of membrane | GO:0031224 | 2.10E-03 | 2.68 | 5003 | 5154 | 1474 | 19011 |
| anchoring junction | GO:0070161 | 2.38E-03 | 2.62 | 464 | 5154 | 168 | 19011 |
| plasma membrane | GO:0005886 | 2.68E-03 | 2.57 | 3921 | 5154 | 1170 | 19011 |
| membrane-enclosed lumen | GO:0031974 | 3.78E-03 | 2.42 | 4324 | 5154 | 1281 | 19011 |
| organelle lumen | GO:0043233 | 3.78E-03 | 2.42 | 4324 | 5154 | 1281 | 19011 |
| intracellular organelle lumen | GO:0070013 | 3.78E-03 | 2.42 | 4324 | 5154 | 1281 | 19011 |
| plasma membrane bounded cell projection | GO:0120025 | 4.77E-03 | 2.32 | 1263 | 5154 | 407 | 19011 |
| cell projection | GO:0042995 | 5.76E-03 | 2.24 | 1313 | 5154 | 421 | 19011 |
| cell-cell junction | GO:0005911 | 7.25E-03 | 2.14 | 348 | 5154 | 129 | 19011 |
| integral component of membrane | GO:0016021 | 7.27E-03 | 2.14 | 4930 | 5154 | 1446 | 19011 |
| nuclear lumen | GO:0031981 | 8.77E-03 | 2.06 | 4049 | 5154 | 1199 | 19011 |
| endosome | GO:0005768 | 2.11E-02 | 1.68 | 572 | 5154 | 196 | 19011 |
| nuclear speck | GO:0016607 | 2.37E-02 | 1.63 | 299 | 5154 | 111 | 19011 |
| nuclear body | GO:0016604 | 3.27E-02 | 1.49 | 662 | 5154 | 222 | 19011 |
| Olfactory transduction | KEGG:04740 | 8.03E-06 | 5.10 | 922 | 2647 | 380 | 7941 |
| Estrogen signaling pathway | KEGG:04915 | 2.69E-04 | 3.57 | 125 | 2647 | 67 | 7941 |
| Staphylococcus aureus infection | KEGG:05150 | 9.74E-03 | 2.01 | 95 | 2647 | 50 | 7941 |
| Developmental Biology | REAC:R-BTA-1266738 | 7.06E-03 | 2.15 | 206 | 1387 | 85 | 4894 |
| Keratinization | REAC:R-BTA-6805567 | 9.54E-03 | 2.02 | 97 | 1387 | 46 | 4894 |
| bta-miR-17-5p | MIRNA:bta-miR-17-5p | 2.94E-02 | 1.53 | 27 | 48 | 17 | 117 |

^1^ P = adjusted P value by Bonferroni correction of multiple tests.

^2^ N_Term = number of terms; N_Query = number of queries; N_Inter = number of intersections; N_domain = number of effective domians.

**Supplementary Table S2.** Annotation enrichment analysis of functional genes in chromosomal segments with high ($\geq50\%$) Brahman breed proportions ^1,2^

| Term name | Term ID | P_adj | -log10(P_adj) | N_Term | N_Query | N_Inter | N_domain |
| --- | --- | --- | --- | --- | --- | --- | --- |
| lysozyme activity | GO:0003796 | 7.89E-05 | 4.10 | 18 | 1,021 | 9 | 17,921 |
| peptidoglycan muralytic activity | GO:0061783 | 6.56E-04 | 3.18 | 22 | 1,021 | 9 | 17,921 |
| serine-type endopeptidase activity | GO:0004252 | 2.44E-03 | 2.61 | 184 | 1,021 | 27 | 17,921 |
| catalytic activity | GO:0003824 | 6.77E-03 | 2.17 | 5,396 | 1,021 | 368 | 17,921 |
| serine-type peptidase activity | GO:0008236 | 9.54E-03 | 2.02 | 198 | 1,021 | 27 | 17,921 |
| serine hydrolase activity | GO:0017171 | 1.49E-02 | 1.83 | 203 | 1,021 | 27 | 17,921 |
| endopeptidase activity | GO:0004175 | 2.76E-02 | 1.56 | 457 | 1,021 | 47 | 17,921 |
| hydrolase activity, hydrolyzing O-glycosyl compounds | GO:0004553 | 4.70E-02 | 1.33 | 107 | 1,021 | 17 | 17,921 |
| keratin filament | GO:0045095 | 1.22E-15 | 14.92 | 84 | 1,054 | 31 | 19,011 |
| intermediate filament cytoskeleton | GO:0045111 | 9.03E-14 | 13.04 | 159 | 1,054 | 40 | 19,011 |
| intermediate filament | GO:0005882 | 1.03E-13 | 12.99 | 130 | 1,054 | 36 | 19,011 |
| supramolecular complex | GO:0099080 | 5.43E-06 | 5.26 | 875 | 1,054 | 89 | 19,011 |
| polymeric cytoskeletal fiber | GO:0099513 | 7.09E-06 | 5.15 | 471 | 1,054 | 57 | 19,011 |
| supramolecular polymer | GO:0099081 | 2.13E-05 | 4.67 | 624 | 1,054 | 68 | 19,011 |
| cytoskeleton | GO:0005856 | 6.21E-05 | 4.21 | 1,684 | 1,054 | 142 | 19,011 |
| supramolecular fiber | GO:0099512 | 6.23E-05 | 4.21 | 616 | 1,054 | 66 | 19,011 |
| MHC class II protein complex | GO:0042613 | 5.93E-03 | 2.23 | 23 | 1,054 | 8 | 19,011 |
| MHC protein complex | GO:0042611 | 8.47E-03 | 2.07 | 24 | 1,054 | 8 | 19,011 |
| cytoplasm | GO:0005737 | 2.58E-02 | 1.59 | 8,379 | 1,054 | 524 | 19,011 |
| Type I diabetes mellitus | KEGG:04940 | 4.55E-04 | 3.34 | 54 | 501 | 14 | 7,941 |
| Allograft rejection | KEGG:05330 | 9.72E-04 | 3.01 | 50 | 501 | 13 | 7,941 |
| Graft-versus-host disease | KEGG:05332 | 2.40E-03 | 2.62 | 54 | 501 | 13 | 7,941 |
| Autoimmune thyroid disease | KEGG:05320 | 2.38E-02 | 1.62 | 58 | 501 | 12 | 7,941 |
| Thiamine metabolism | KEGG:00730 | 4.60E-02 | 1.34 | 17 | 501 | 6 | 7,941 |
| Keratinization | REAC:R-BTA-6805567 | 2.21E-06 | 5.66 | 97 | 327 | 24 | 4,894 |
| Developmental Biology | REAC:R-BTA-1266738 | 1.74E-02 | 1.76 | 206 | 327 | 29 | 4,894 |
| Pathogenic Escherichia coli infection | WP:WP3157 | 3.97E-02 | 1.40 | 52 | 253 | 11 | 3,688 |

^1^ P = adjusted P value by Bonferroni correction of multiple tests.

^2^ N_Term = number of terms; N_Query = number of queries; N_Inter = number of intersections; N_domain = number of effective domians.

**Supplementary Table S3.** Examples of reported QTLs in chromosomal regions with high Angus or Brahman breed proportions

| Chr:segment | Angus% | Brahman% | Position, Mb | Trait | Category | PUBMED ID |
| --- | --- | --- | --- | --- | --- | --- |
| 1:7-8 | 92.9 | 7.1 | 1.02-10.25 | Body weight (weaning) | Production | 20477797 |
| 3:35-36 | 91.9 | 8.1 | 24.56-43.41 | Carcass weight | Meat and Carcass | 20477797 |
|  | 91.9 | 8.1 | 32.08-55.96 | Carcass weight | Meat and Carcass | 20477797 |
|  | 91.9 | 8.1 | 32.08-55.96 | Body weight (weaning) | Production | 20477797 |
|  | 91.9 | 8.1 | 32.08-55.96 | Body weight (birth) | Production | 20477797 |
| 3:68-69 | 91.4 | 8.6 | 64.09-82.33 | Carcass weight | Meat and Carcass | 20477797 |
|  | 91.4 | 8.6 | 64.09-82.33 | Calving ease | Reproduction | 20477797 |
|  | 91.4 | 8.6 | 64.09-82.33 | Body weight (mature) | Production | 20477797 |
|  | 91.4 | 8.6 | 64.09-73.17 | Body weight (birth) | Production | 20477797 |
|  | 91.4 | 8.6 | 64.09-73.17 | Body weight (yearling) | Production | 20477797 |
|  | 91.4 | 8.6 | 64.09-73.17 | Height (mature) | Production | 20477797 |
|  | 91.4 | 8.6 | 64.09-82.33 | Longissimus muscle area | Meat and Carcass | 20477797 |
| 3:73-74 | 91.2 | 8.8 | 64.09-82.33 | Carcass weight | Meat and Carcass | 20477797 |
|  | 91.2 | 8.8 | 64.09-82.33 | Calving ease | Reproduction | 20477797 |
|  | 91.2 | 8.8 | 64.09-82.33 | Body weight (mature) | Production | 20477797 |
|  | 91.2 | 8.8 | 64.09-73.17 | Body weight (birth) | Production | 20477797 |
|  | 91.2 | 8.8 | 64.09-73.17 | Body weight (yearling) | Production | 20477797 |
|  | 91.2 | 8.8 | 64.09-73.17 | Height (mature) | Production | 20477797 |
|  | 91.2 | 8.8 | 64.09-82.33 | Longissimus muscle area | Meat and Carcass | 20477797 |
| 3:76-77 | 92.0 | 8.0 | 64.09-82.33 | Carcass weight | Meat and Carcass | 20477797 |
|  | 92.0 | 8.0 | 64.09-82.33 | Calving ease | Reproduction | 20477797 |
|  | 92.0 | 8.0 | 64.09-82.33 | Body weight (mature) | Production | 20477797 |
|  | 92.0 | 8.0 | 64.09-82.33 | Longissimus muscle area | Meat and Carcass | 20477797 |
| 4:95-96 | 95.3 | 4.7 | 76.59-100.47 | Fat thickness at the 12th rib | Meat and Carcass | 20477797 |
|  | 95.3 | 4.7 | 88.01-107.93 | Scrotal circumference | Reproduction | 20477797 |
|  | 95.3 | 4.7 | 88.01-100.47 | Body weight (mature) | Production | 20477797 |
| 4:96-97 | 91.5 | 8.5 | 76.59-100.47 | Fat thickness at the 12th rib | Meat and Carcass | 20477797 |
|  | 91.5 | 8.5 | 88.01-107.93 | Scrotal circumference | Reproduction | 20477797 |
|  | 91.5 | 8.5 | 88.01-100.47 | Body weight (mature) | Production | 20477797 |
| 6:71-72 | 91.6 | 8.4 | 64.78-78.91 | Body weight (mature) | Production | 20477797 |
| 8:84-85 | 91.6 | 8.4 | 74.04-104.66 | Carcass weight | Meat and Carcass | 20477797 |
|  | 91.6 | 8.4 | 81.75-113.38 | Body weight (birth) | Production | 20477797 |
|  | 91.6 | 8.4 | 81.75-104.66 | Calving ease | Reproduction | 20477797 |
|  | 91.6 | 8.4 | 81.75-104.66 | Marbling score | Meat and Carcass | 20477797 |
|  | 91.6 | 8.4 | 81.75-104.66 | Calving ease | Reproduction | 20477797 |
|  | 91.6 | 8.4 | 81.75-104.66 | Longissimus muscle area | Meat and Carcass | 20477797 |
|  | 91.6 | 8.4 | 81.75-104.66 | Height (mature) | Production | 20477797 |
| 10:48-49 | 92.5 | 7.5 | 21.08-52.25 | Calving ease | Reproduction | 20477797 |
|  | 92.5 | 7.5 | 30.79-52.25 | Body weight (yearling) | Production | 20477797 |
|  | 92.5 | 7.5 | 38.85-52.25 | Marbling score | Meat and Carcass | 20477797 |
|  | 92.5 | 7.5 | 38.85-52.25 | Body weight (mature) | Production | 20477797 |
| 10:61-62 | 92.7 | 7.3 | 52.25-69.36 | Height (yearling) | Production | 20477797 |
| 15:3-4 | 91.1 | 8.9 | 0-6.42 | Body weight (weaning) | Production | 20477797 |
|  | 91.1 | 8.9 | 0-6.42 | Marbling score | Meat and Carcass | 20477797 |
|  | 91.1 | 8.9 | 0-6.42 | Height (mature) | Production | 20477797 |
| 17:71-72 | 91.3 | 8.7 | 52.12-72.18 | Calving ease | Reproduction | 20477797 |
|  | 91.3 | 8.7 | 63.39-75.16 | Body weight (mature) | Production | 20477797 |
|  | 91.3 | 8.7 | 63.39-75.16 | Longissimus muscle area | Meat and Carcass | 20477797 |
|  | 91.3 | 8.7 | 63.39-72.18 | Fat thickness at the 12th rib | Meat and Carcass | 20477797 |
| 18:10-11 | 93.8 | 6.2 | 0.78-16.19 | Longissimus muscle area | Meat and Carcass | 20477797 |
|  | 93.8 | 6.2 | 2.23-10.55 | Marbling score | Meat and Carcass | 20477797 |
|  | 93.8 | 6.2 | 2.23-16.19 | Fat thickness at the 12th rib | Meat and Carcass | 20477797 |
| 18:11-12 | 95.9 | 4.1 | 0.78-16.19 | Longissimus muscle area | Meat and Carcass | 20477797 |
|  | 95.9 | 4.1 | 2.23-16.19 | Fat thickness at the 12th rib | Meat and Carcass | 20477797 |
| 18:12-13 | 92.7 | 7.3 | 0.78-16.19 | Longissimus muscle area | Meat and Carcass | 20477797 |
|  | 92.7 | 7.3 | 2.23-16.19 | Fat thickness at the 12th rib | Meat and Carcass | 20477797 |
| 18:13-14 | 92.3 | 7.7 | 0.78-16.19 | Longissimus muscle area | Meat and Carcass | 20477797 |
|  | 92.3 | 7.7 | 2.23-16.19 | Fat thickness at the 12th rib | Meat and Carcass | 20477797 |
| 18:14-15 | 95.2 | 4.8 | 0.78-16.19 | Longissimus muscle area | Meat and Carcass | 20477797 |
|  | 95.2 | 4.8 | 2.23-16.19 | Fat thickness at the 12th rib | Meat and Carcass | 20477797 |
| 18:18-19 | 95.8 | 4.3 | 18.09-18.09 | Long-chain fatty acid content | Meat and Carcass | 24156620 |
| 18:20-21 | 92.6 | 7.4 | 19.15-31.46 | Longissimus muscle area | Meat and Carcass | 20477797 |
|  | 92.6 | 7.4 | 19.15-26.12 | Body weight (mature) | Production | 20477797 |
| 18:21-22 | 93.6 | 6.4 | 19.15-31.46 | Longissimus muscle area | Meat and Carcass | 20477797 |
|  | 93.6 | 6.4 | 19.15-26.12 | Body weight (mature) | Production | 20477797 |
| 18:26-27 | 92.2 | 7.8 | 19.15-31.46 | Longissimus muscle area | Meat and Carcass | 20477797 |
|  | 92.2 | 7.8 | 19.15-26.12 | Body weight (mature) | Production | 20477797 |
|  | 92.2 | 7.8 | 26.12-43.43 | Body weight (weaning) | Production | 20477797 |
| 18:27-28 | 92.9 | 7.1 | 19.15-31.46 | Longissimus muscle area | Meat and Carcass | 20477797 |
|  | 92.9 | 7.1 | 26.12-43.43 | Body weight (weaning) | Production | 20477797 |
| 18:39-40 | 94.1 | 5.9 | 26.12-43.43 | Body weight (weaning) | Production | 20477797 |
|  | 94.1 | 5.9 | 36.75-43.43 | Body weight (mature) | Production | 20477797 |
| 22:7-8 | 93.6 | 6.4 | 0-13.28 | Carcass weight | Meat and Carcass | 20477797 |
|  | 93.6 | 6.4 | 4.04-13.28 | Marbling score | Meat and Carcass | 20477797 |
| 23:40-41 | 91.2 | 8.8 | 34.32-44.58 | Fat thickness at the 12th rib | Meat and Carcass | 20477797 |
|  | 91.2 | 8.8 | 38.19-44.58 | Body weight (weaning) | Production | 20477797 |
| 23:43-44 | 91.3 | 8.7 | 34.32-44.58 | Fat thickness at the 12th rib | Meat and Carcass | 20477797 |
|  | 91.3 | 8.7 | 38.19-44.58 | Body weight (weaning) | Production | 20477797 |
| 2:119-120 | 25.6 | 74.4 | 117.27-134.4 | Bovine respiratory disease susceptibility | Health | 21148784 |
| 5:34-35 | 40.3 | 59.7 | 34.14-34.14 | Insulin-like growth factor 1 level | Health | 22811567 |
|  | 40.3 | 59.7 | 34.18-34.18 | Insulin-like growth factor 1 level | Health | 22811567 |
|  | 40.3 | 59.7 | 34.22-34.22 | Insulin-like growth factor 1 level | Health | 22811567 |
| 14:26-27 | 38.3 | 61.7 | 26.08-26.08 | Insulin-like growth factor 1 level | Health | 22811567 |
|  | 38.3 | 61.7 | 26.47-26.47 | Insulin-like growth factor 1 level | Health | 22811567 |
|  | 38.3 | 61.7 | 26.51-26.51 | Insulin-like growth factor 1 level | Health | 22811567 |
|  | 38.3 | 61.7 | 26.54-26.54 | Insulin-like growth factor 1 level | Health | 22811567 |
|  | 38.3 | 61.7 | 26.71-26.71 | Insulin-like growth factor 1 level | Health | 22811567 |
|  | 38.3 | 61.7 | 26.77-26.77 | Insulin-like growth factor 1 level | Health | 22811567 |
|  | 38.3 | 61.7 | 26.87-26.87 | Insulin-like growth factor 1 level | Health | 22811567 |
|  | 38.3 | 61.7 | 26.9-26.9 | Insulin-like growth factor 1 level | Health | 22811567 |
|  | 38.3 | 61.7 | 26.95-26.95 | Insulin-like growth factor 1 level | Health | 22811567 |
| 16:29-30 | 40.6 | 59.4 | 29.73-29.73 | Blood cortisol level | Health | 32292413 |
|  | 40.6 | 59.4 | 29.73-29.73 | Blood cortisol level | Health | 32292413 |
|  | 40.6 | 59.4 | 29.73-29.73 | Blood cortisol level | Health | 32292413 |
|  | 40.6 | 59.4 | 29.73-29.73 | Blood cortisol level | Health | 32292413 |
|  | 40.6 | 59.4 | 29.73-29.73 | Blood cortisol level | Health | 32292413 |
