# Supplementary material for: A look under the hood of genomic-estimated breed compositions for brangus cattle: What have we learned?
Source: Front Genet. 2023 Mar 28;14:1080279. doi: 10.3389/fgene.2023.1080279 (PMC10086375; doi:10.3389/fgene.2023.1080279)

**Supplementary Figure S1.** Correlations and scatterplots of genomic breed compositions for 3,605 Brangus cattle obtained using different strategies: (A) GBC vs. C_GBC; (B) GBC vs. S_GBC (1Mb); (C) GBC vs. S_GBC (5Mb); (D) GBC vs. S_GBC (10 Mb).

(A)

**
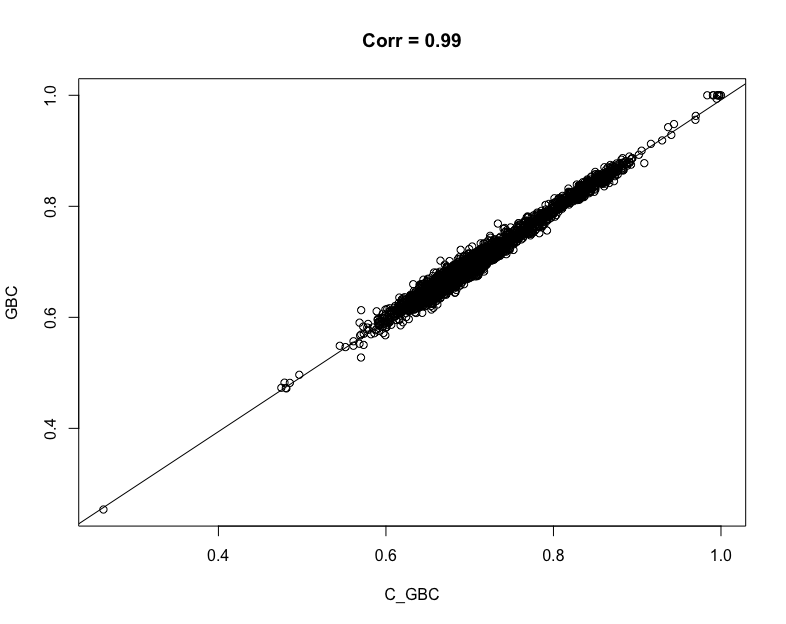
**

(B)

**
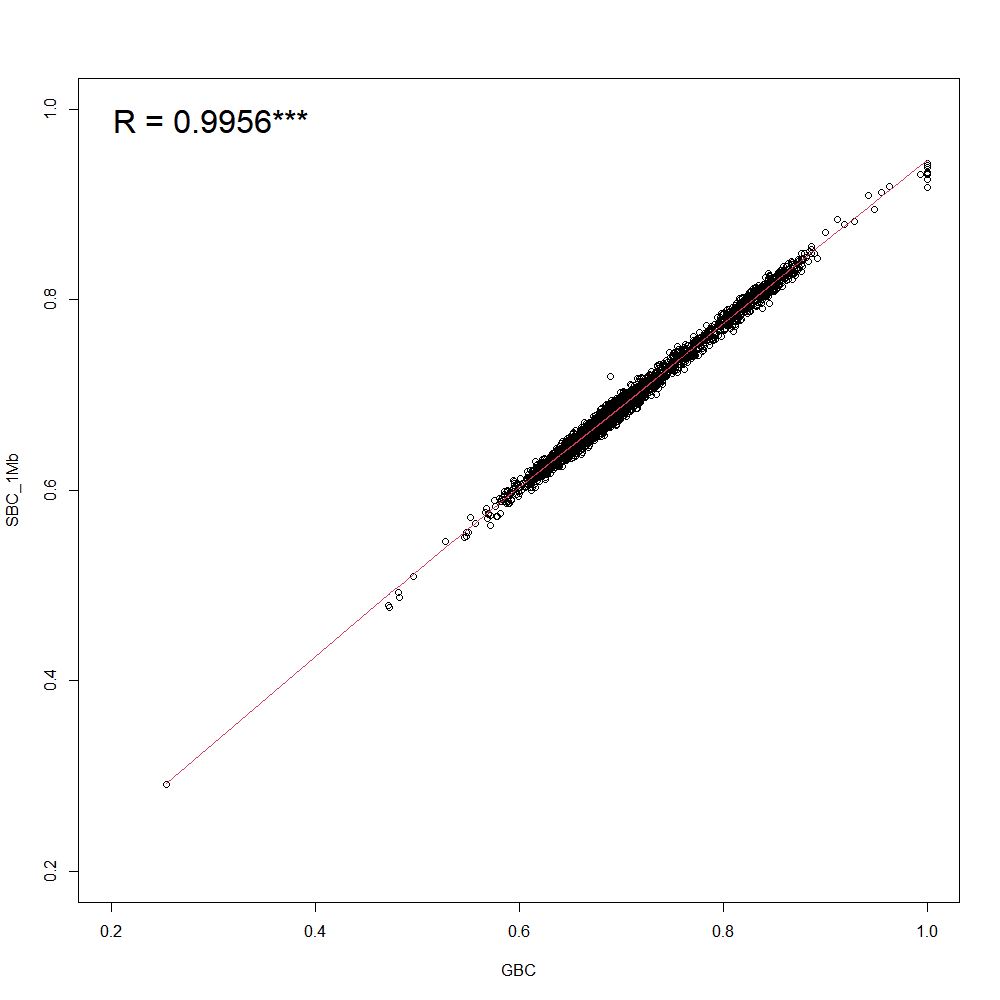
**

(C)

**
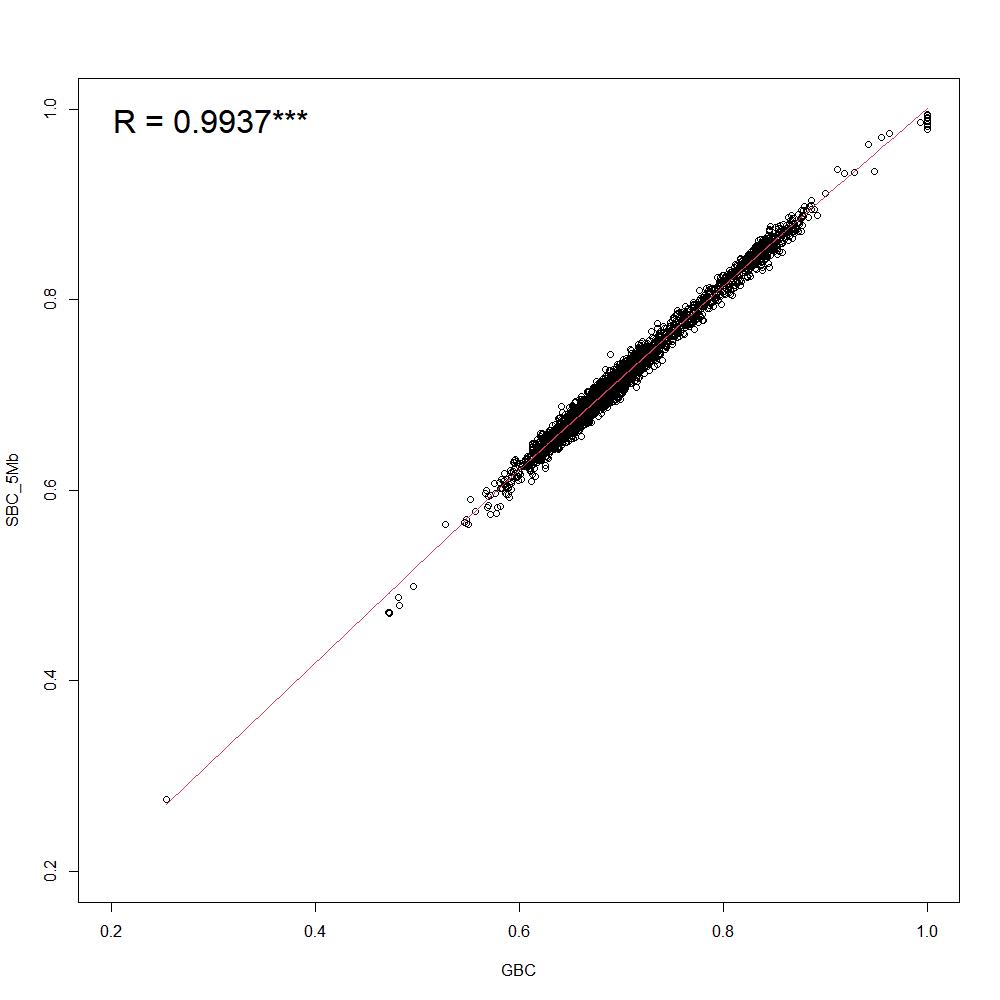
**

(D)


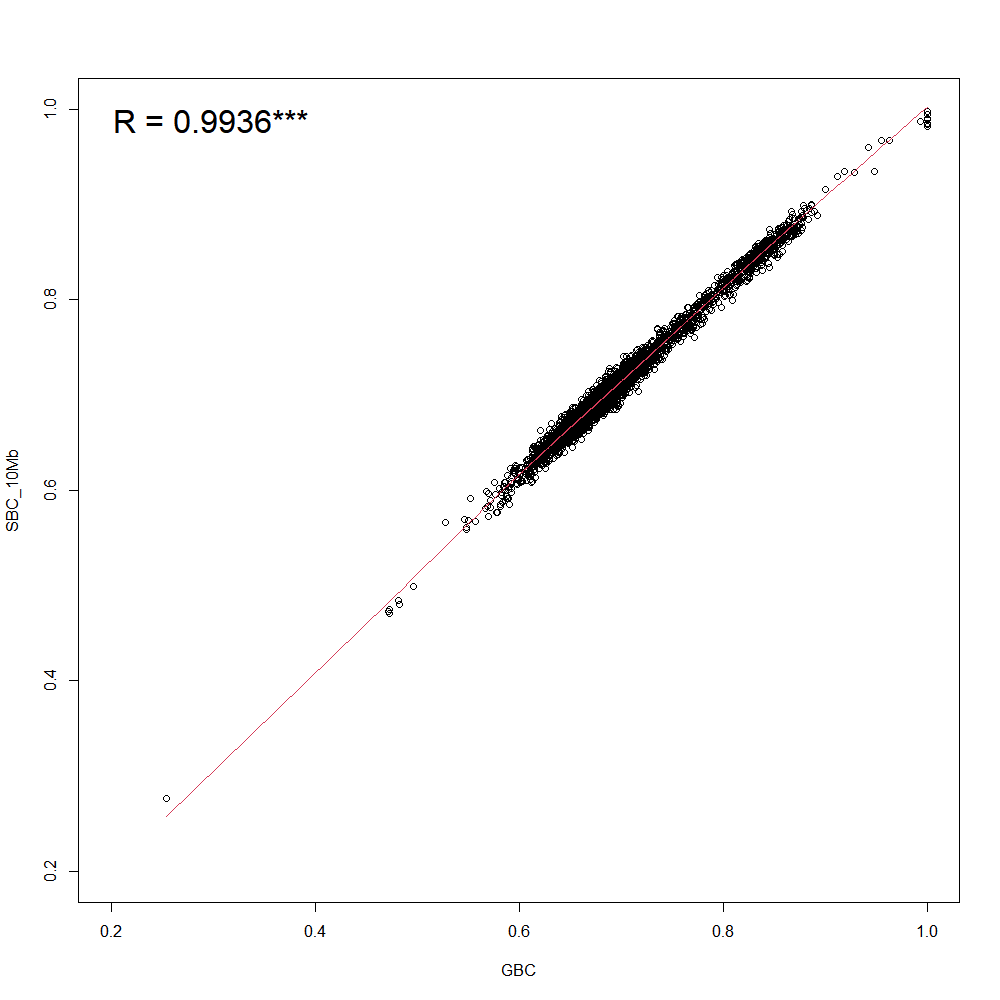


**Supplementary Figure S2.** Distribution of estimated Angus (red) and Brahman (yellow) breed proportion by varying window sizes in 3,605 Brangus cattle: (A) window size = 1Mb; (B) window size = 5 Mb; (C) window size = 10 Mb. The black dashed line is the population mean of genomic-estimated breed compositions (GBC), and the blue dashed line is the population mean of chromosomal-estimated breed compositions (CBC)

(A)


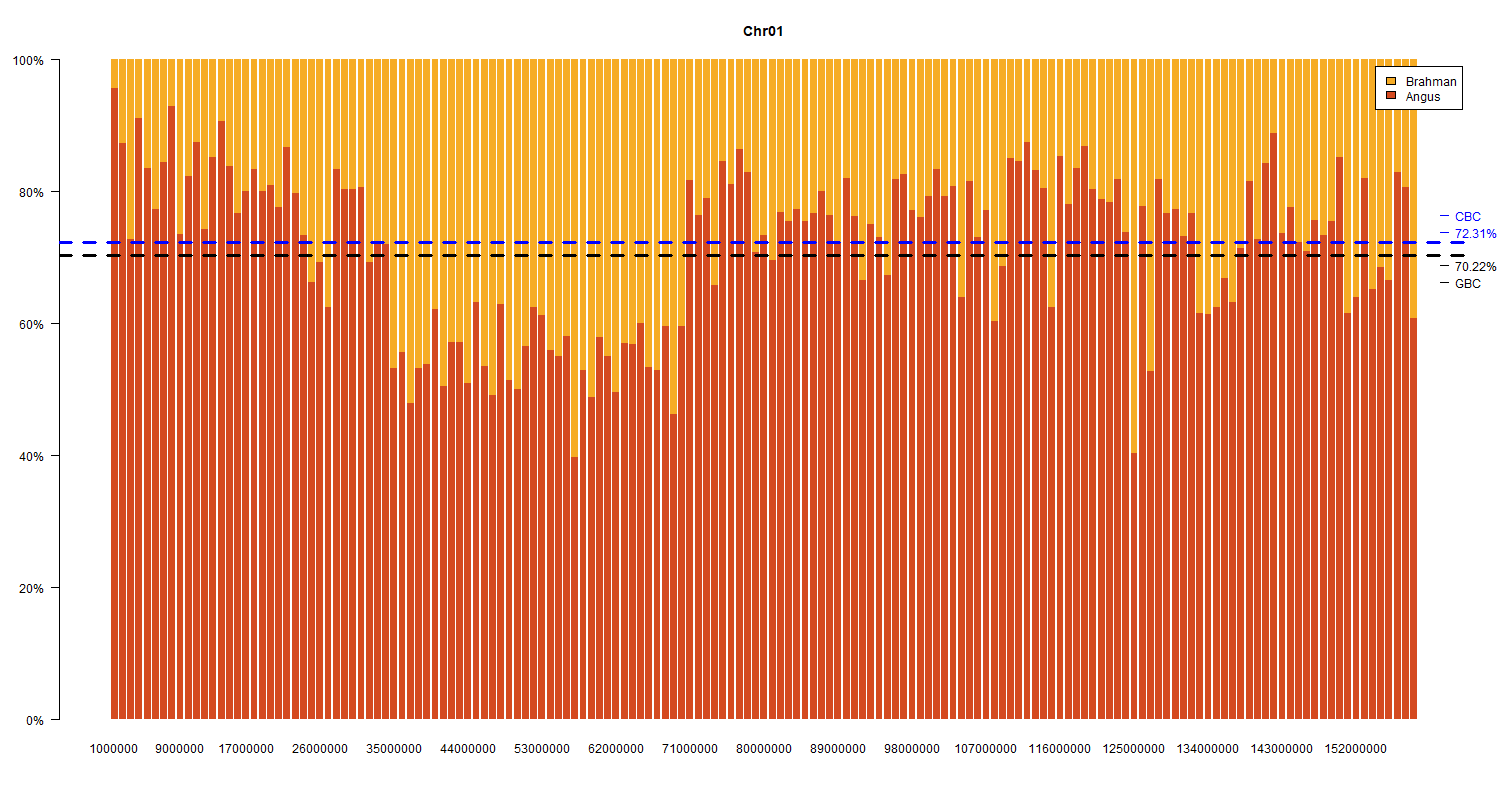

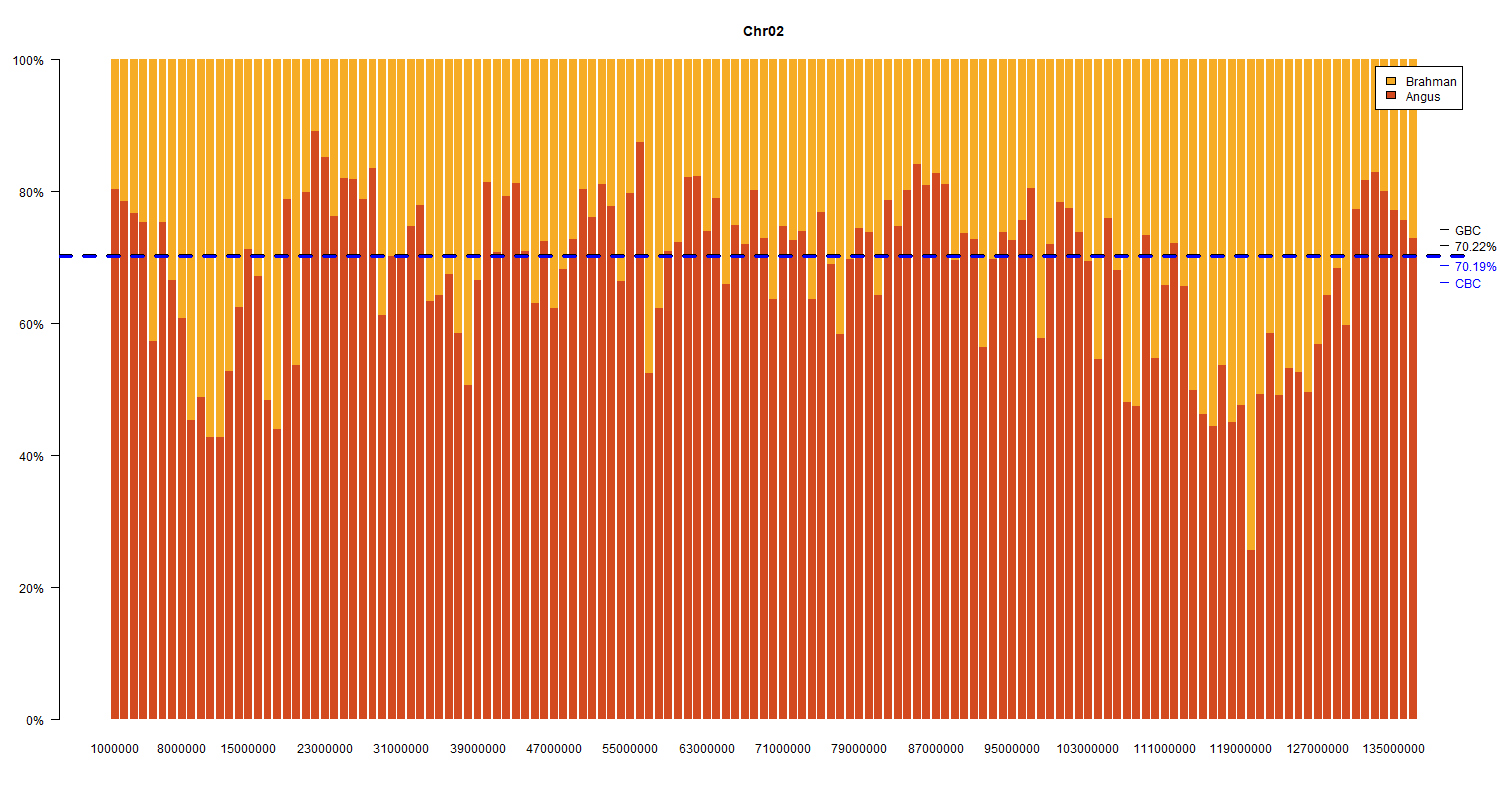

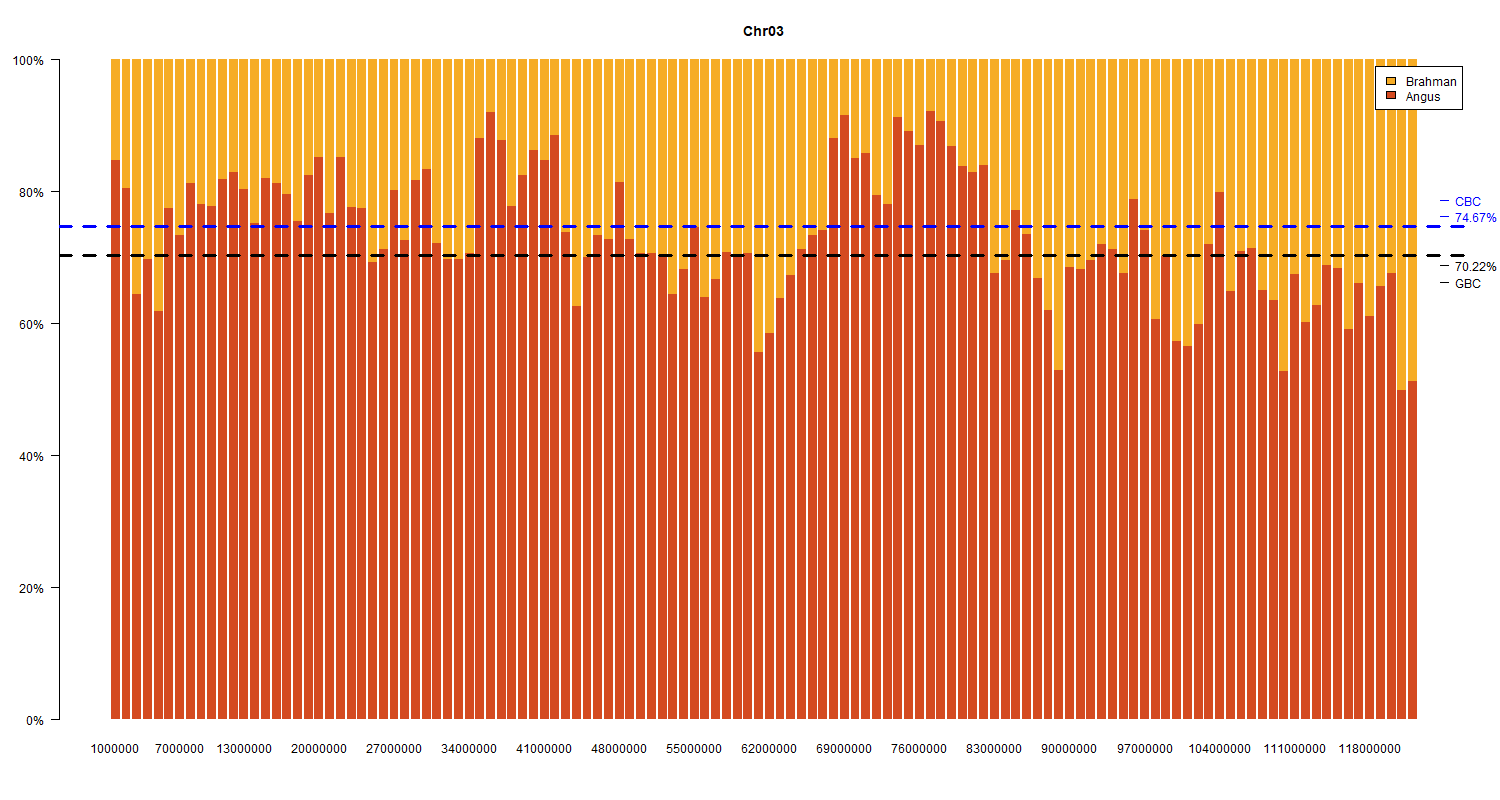

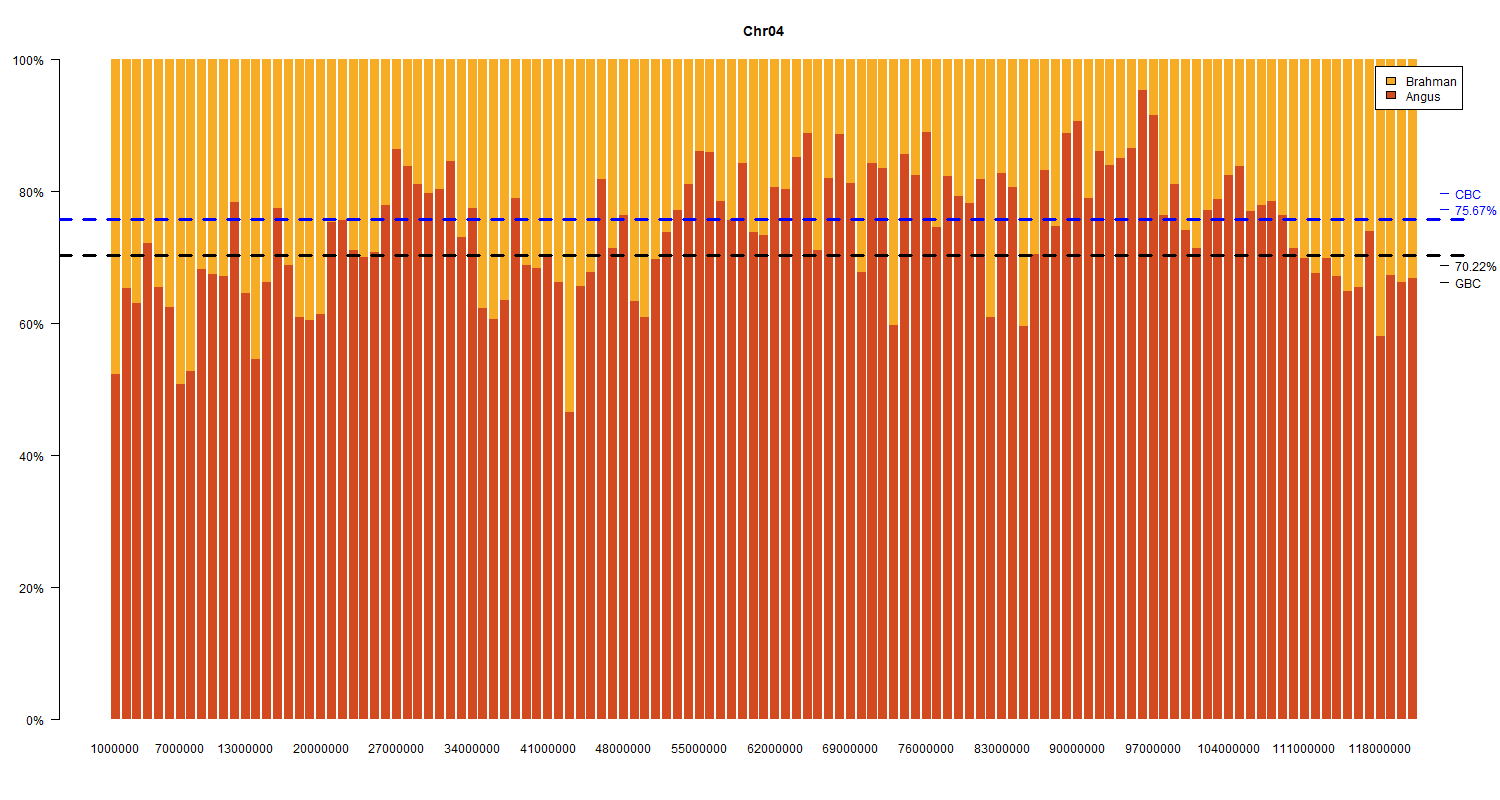

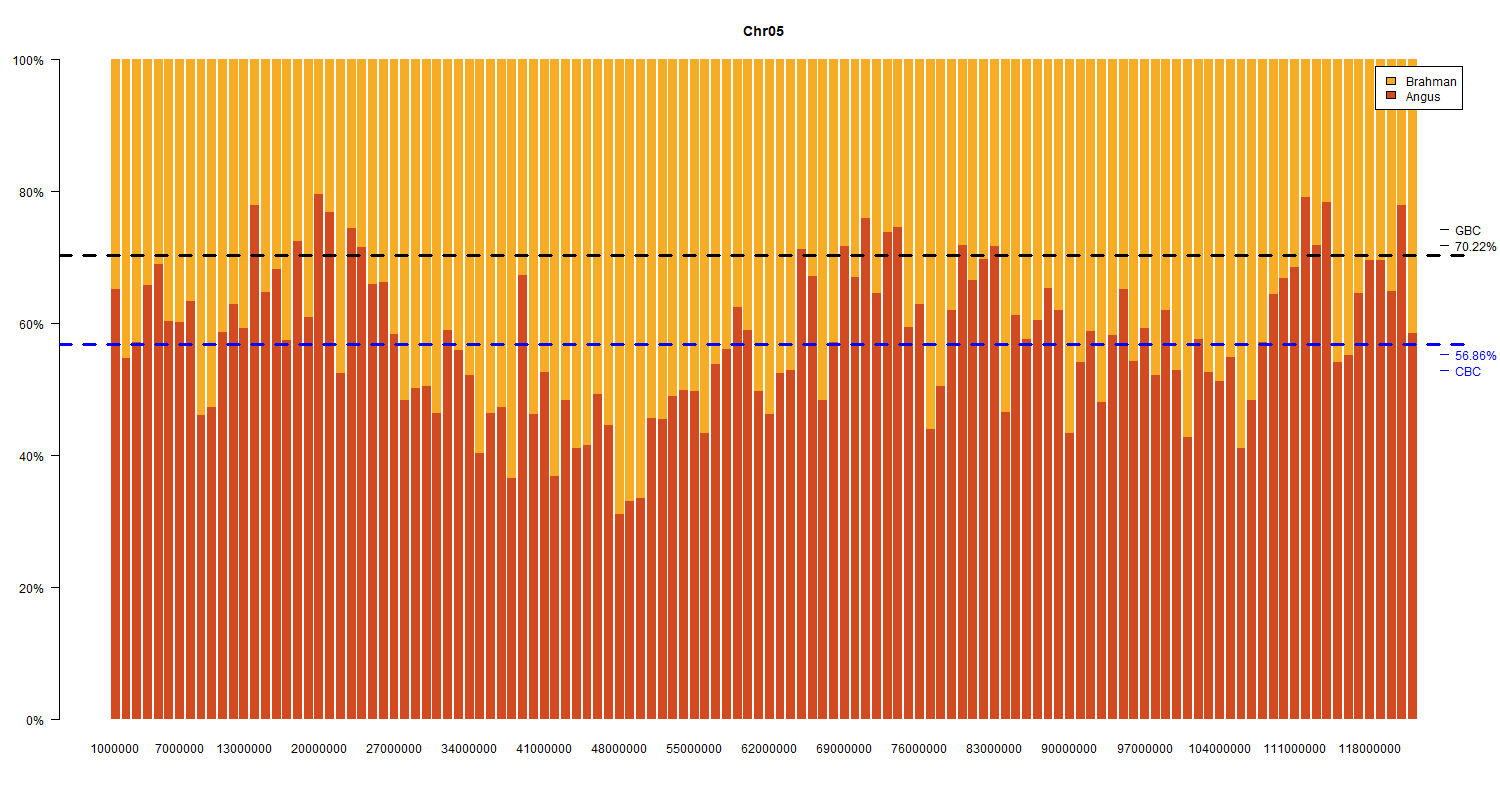

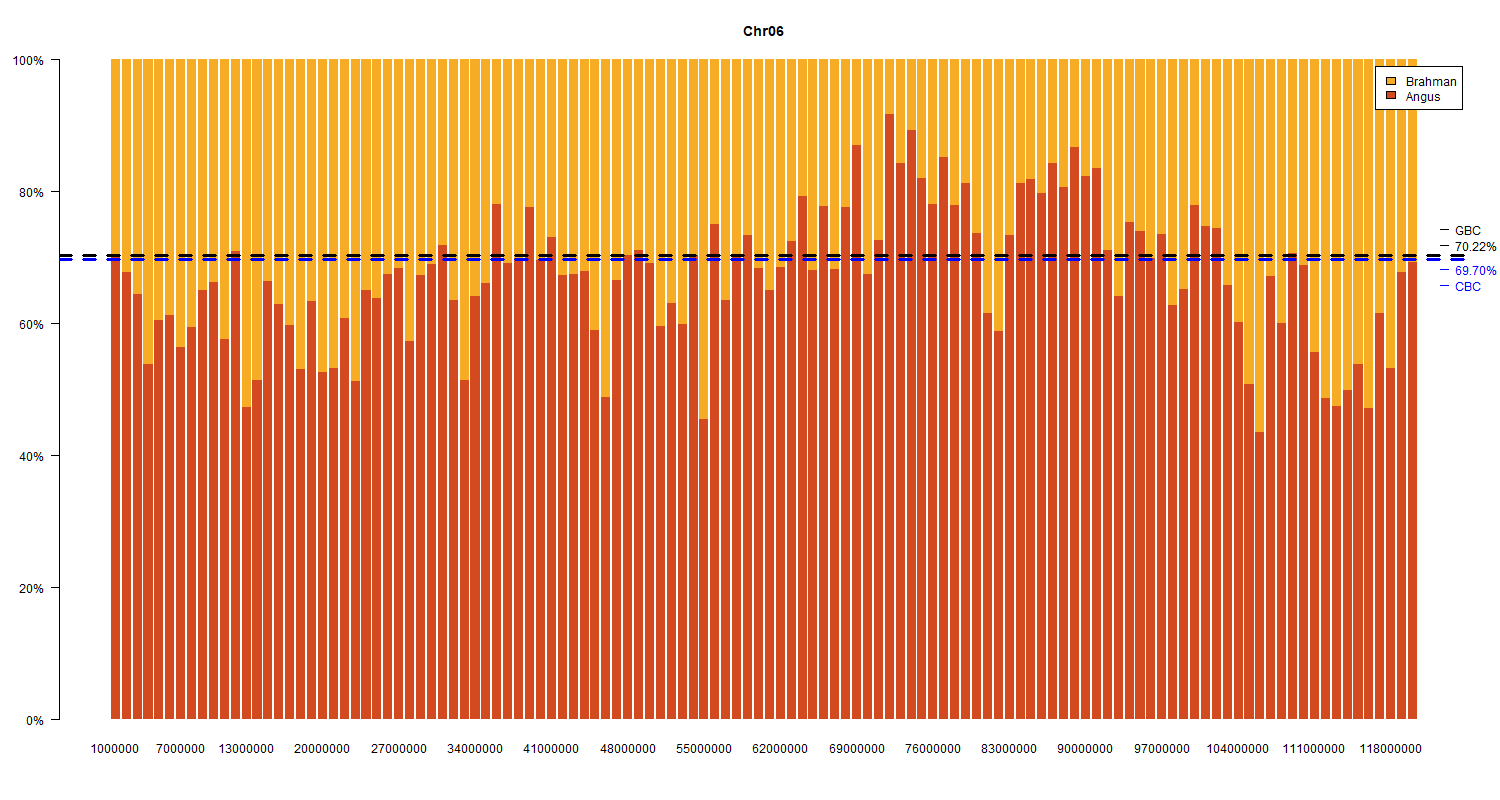

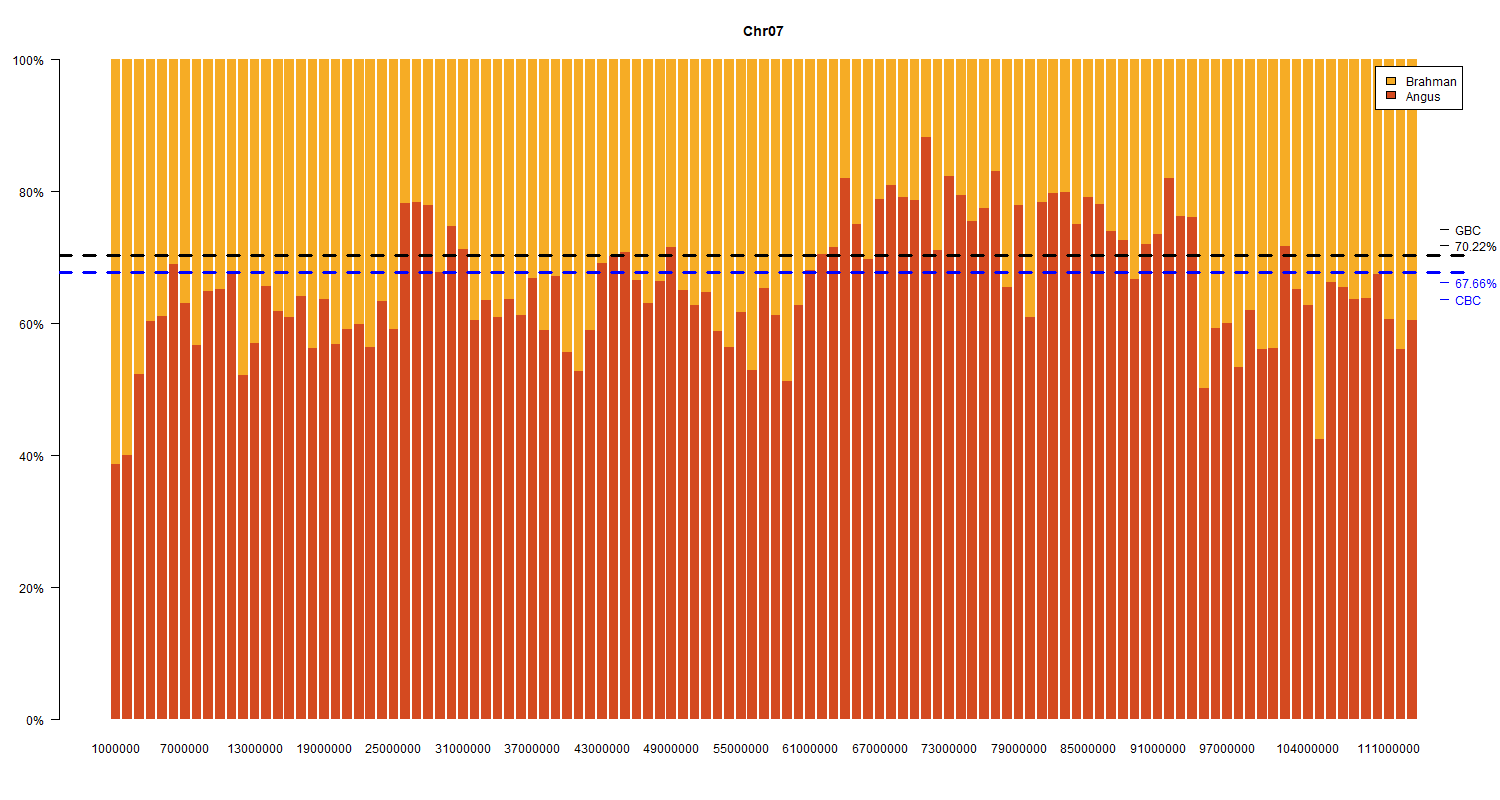

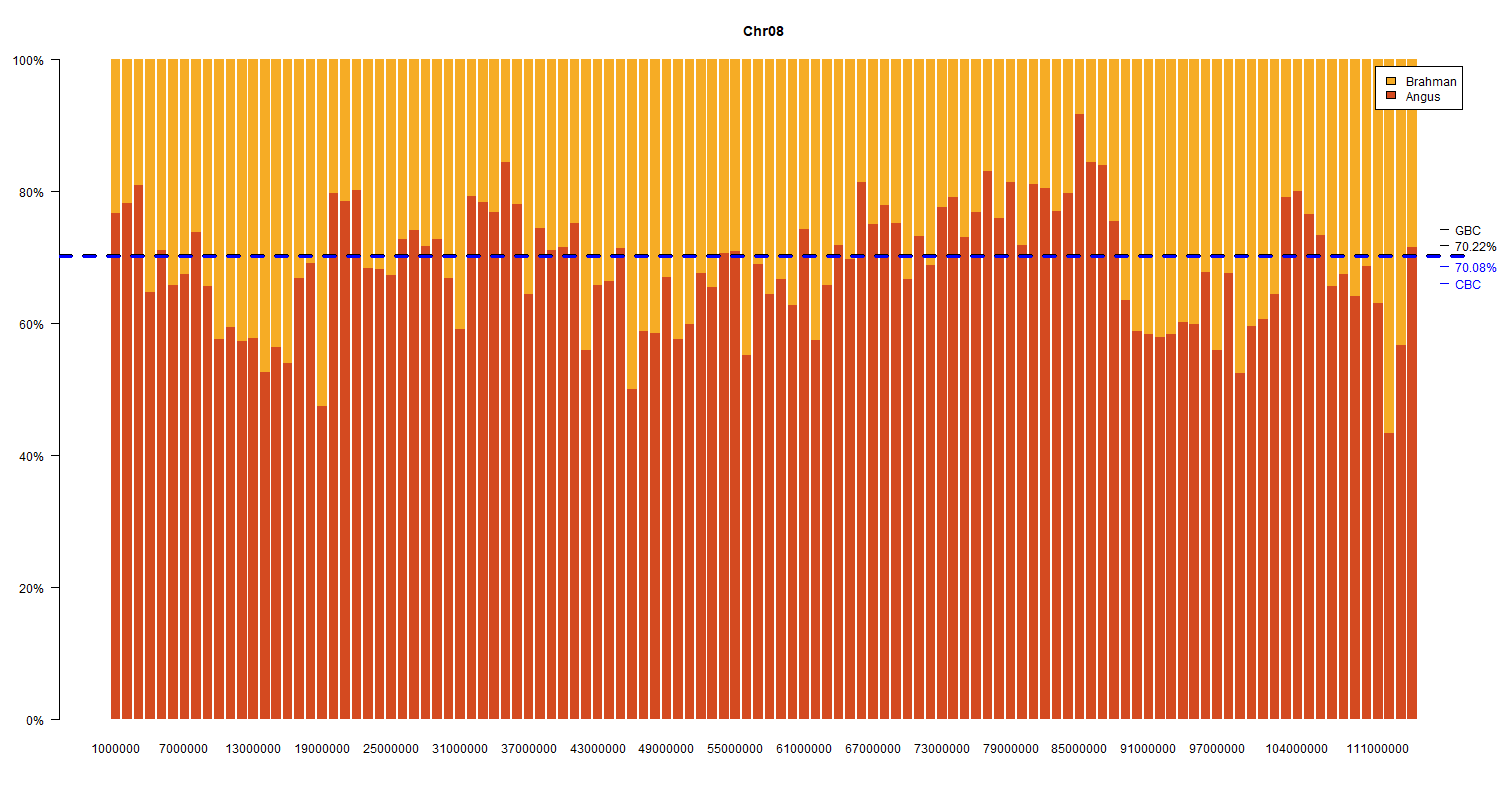

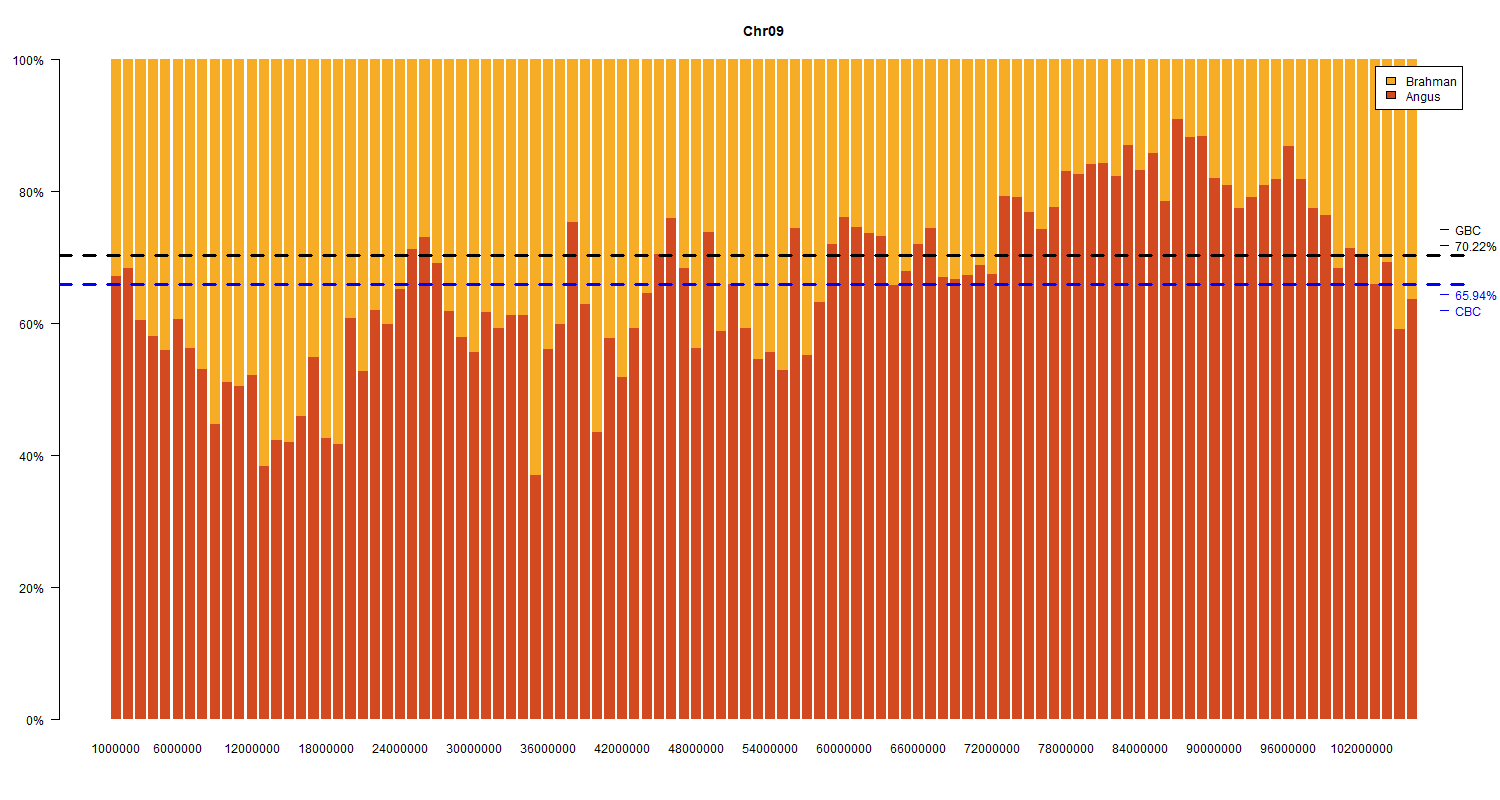

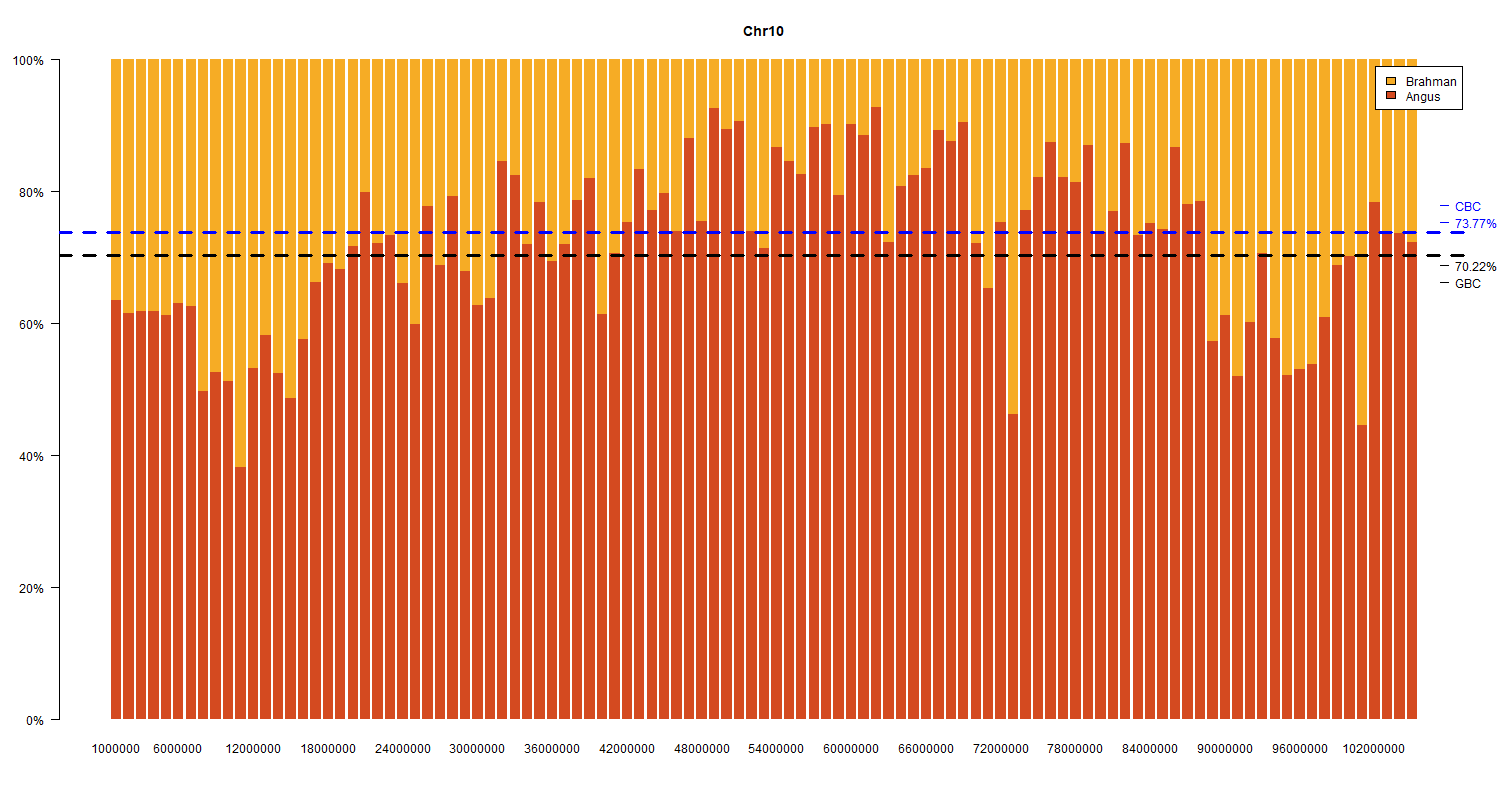

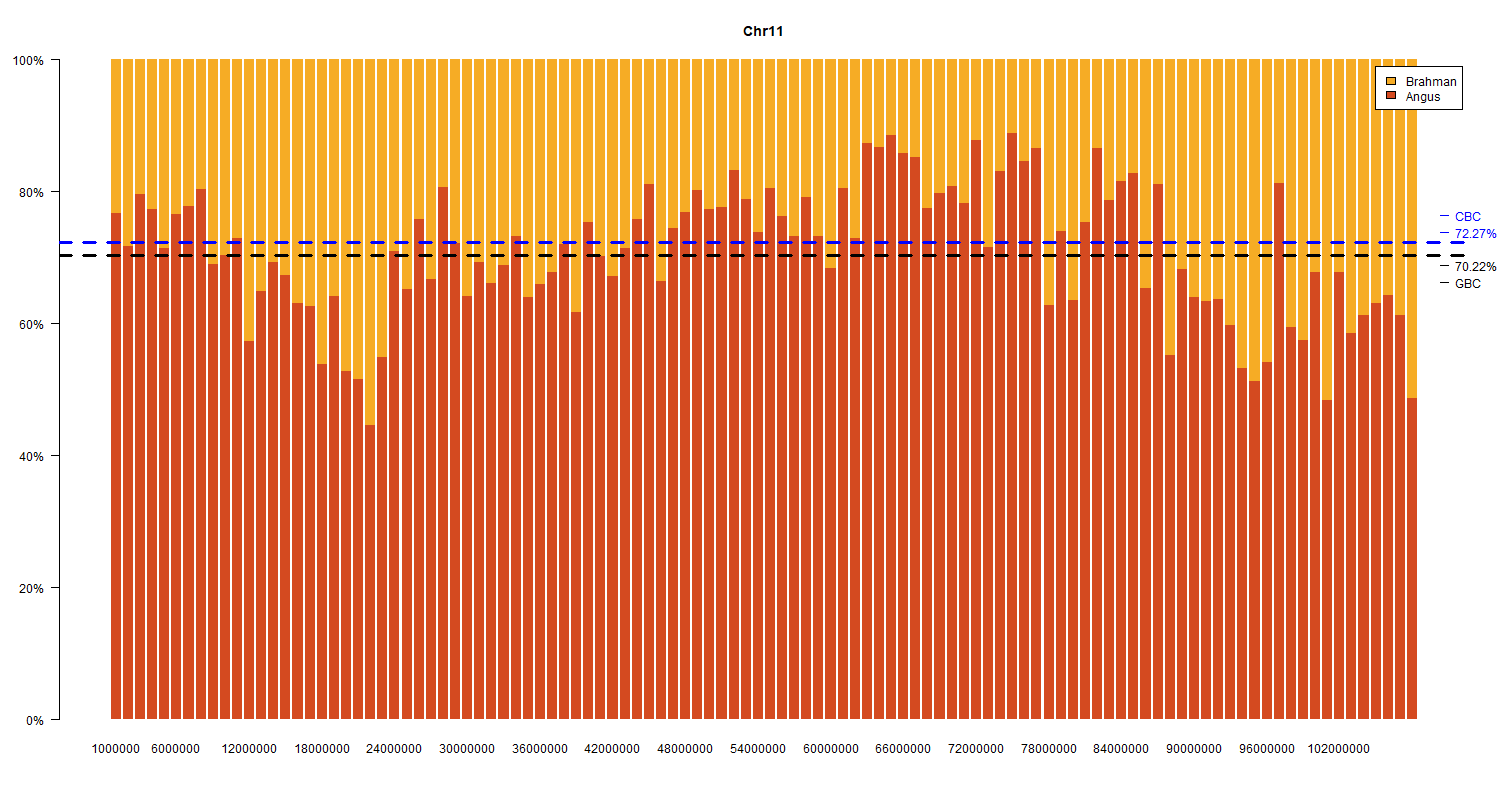

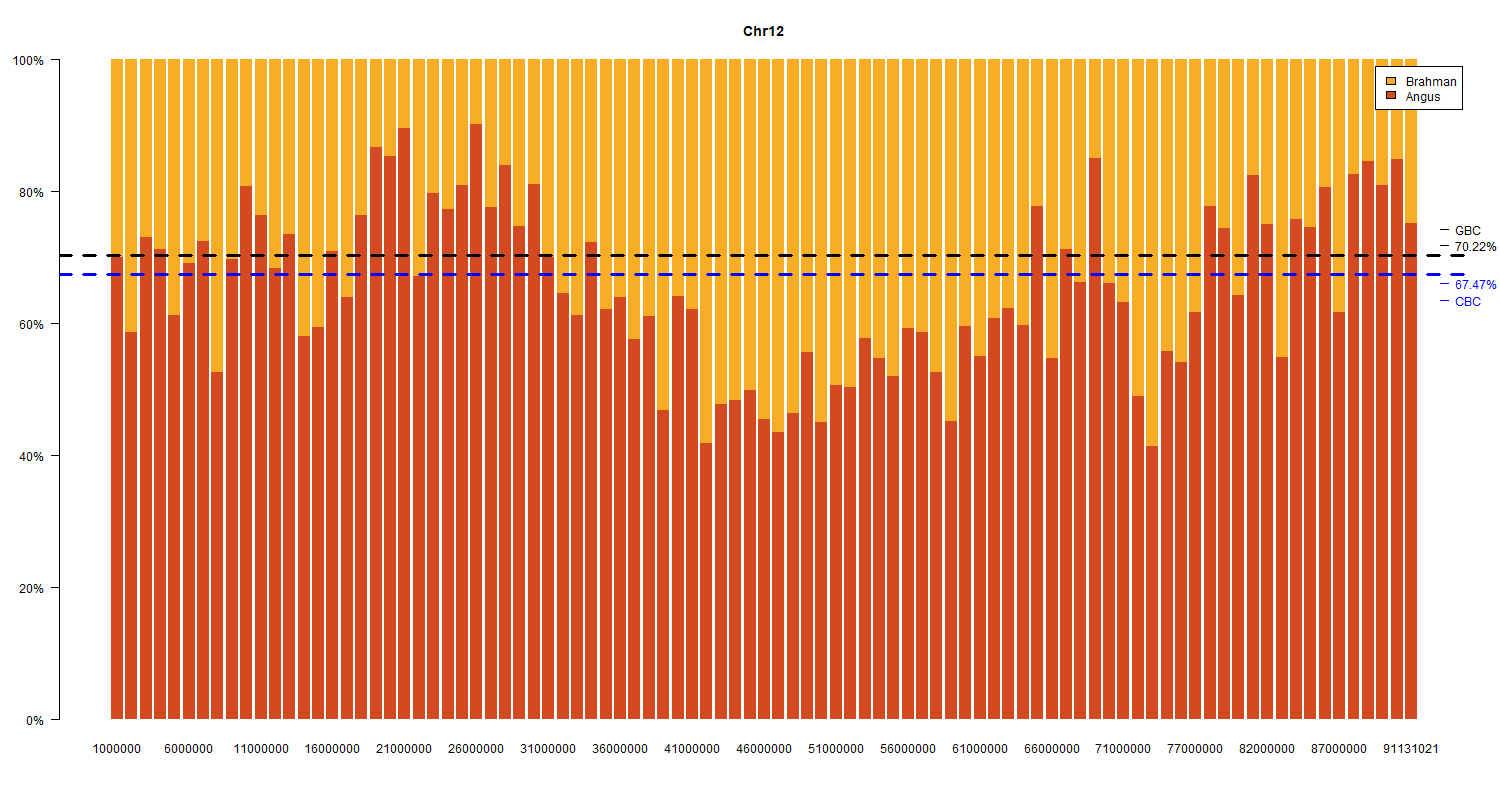

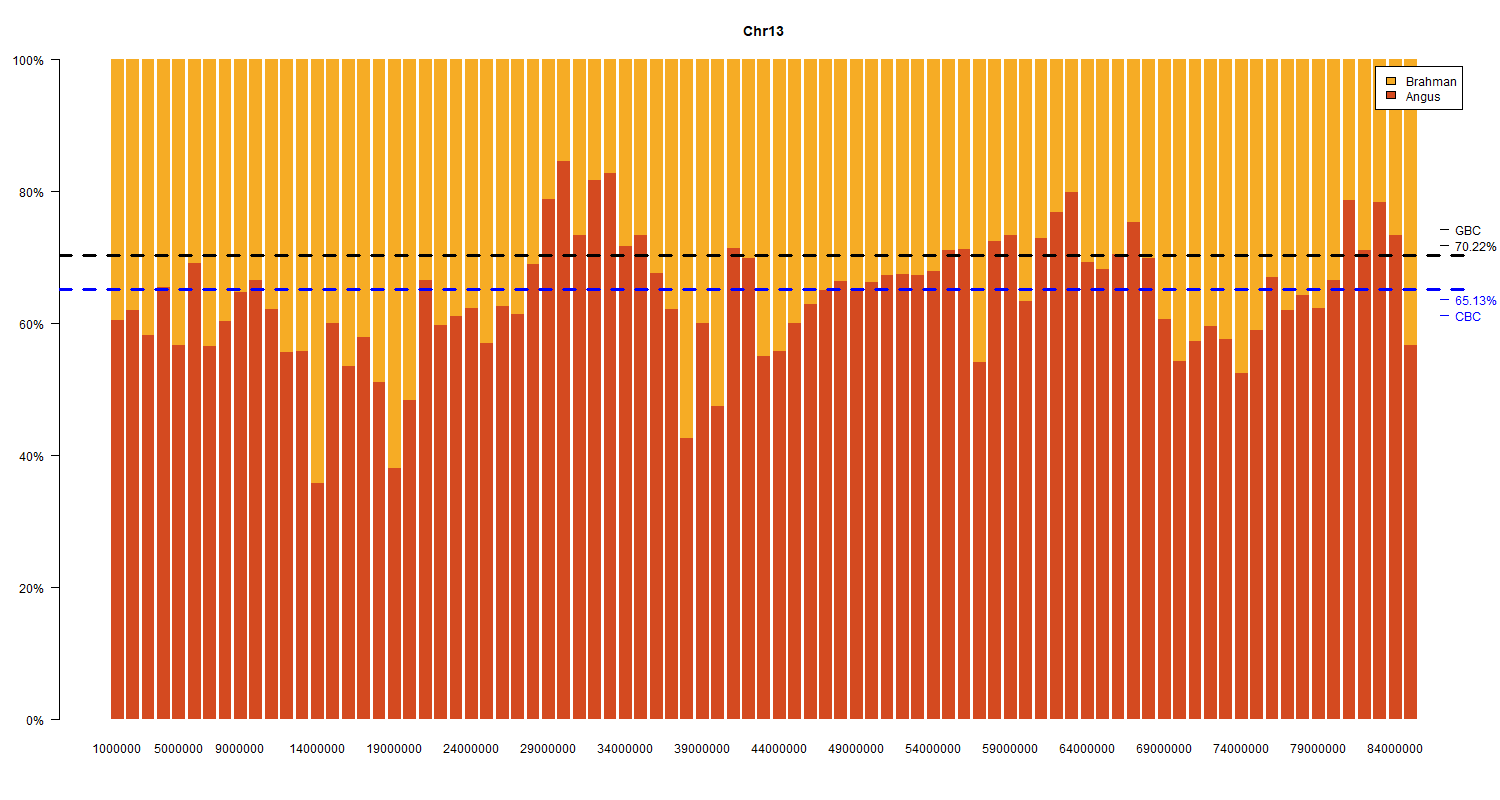

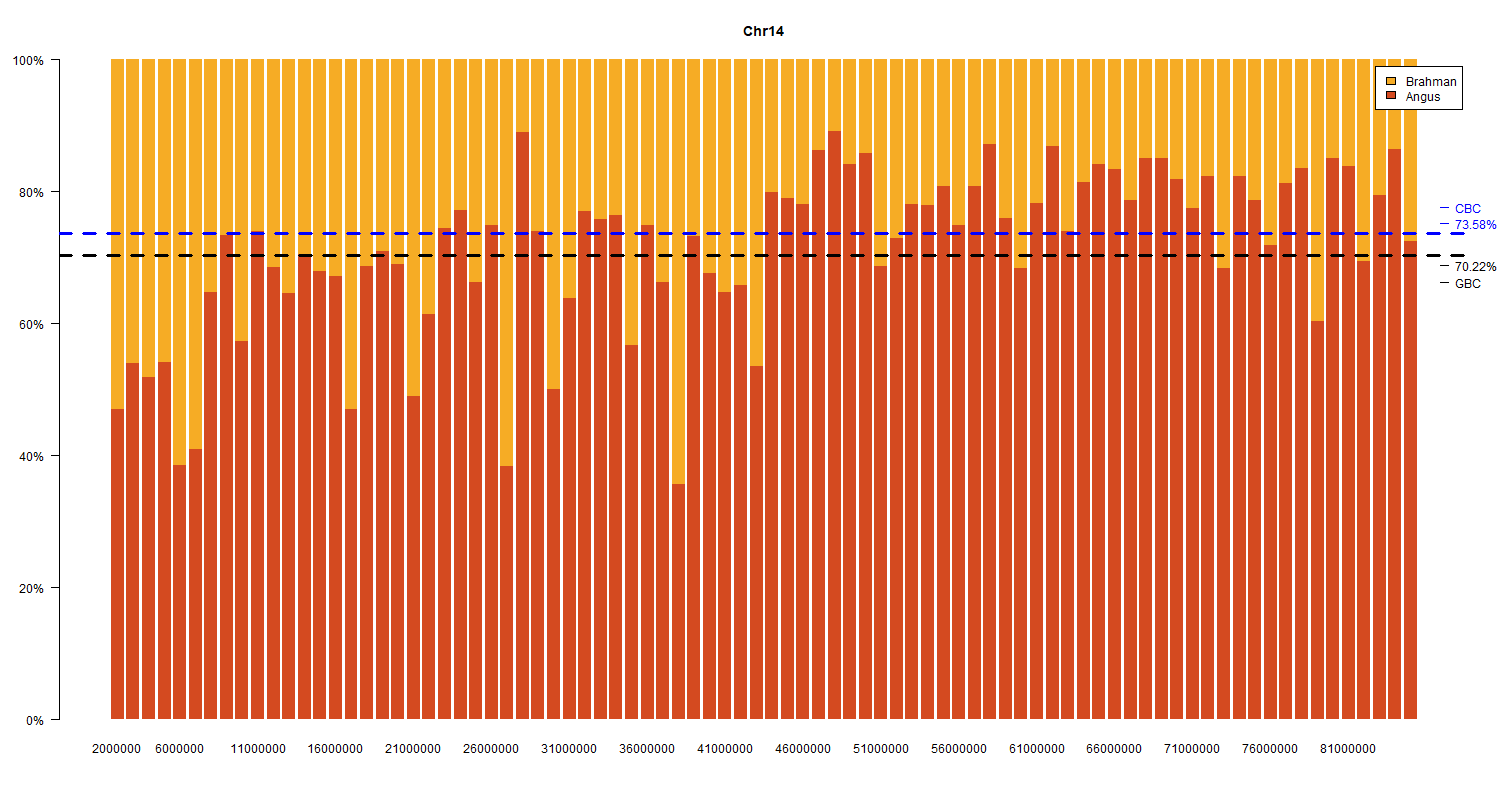

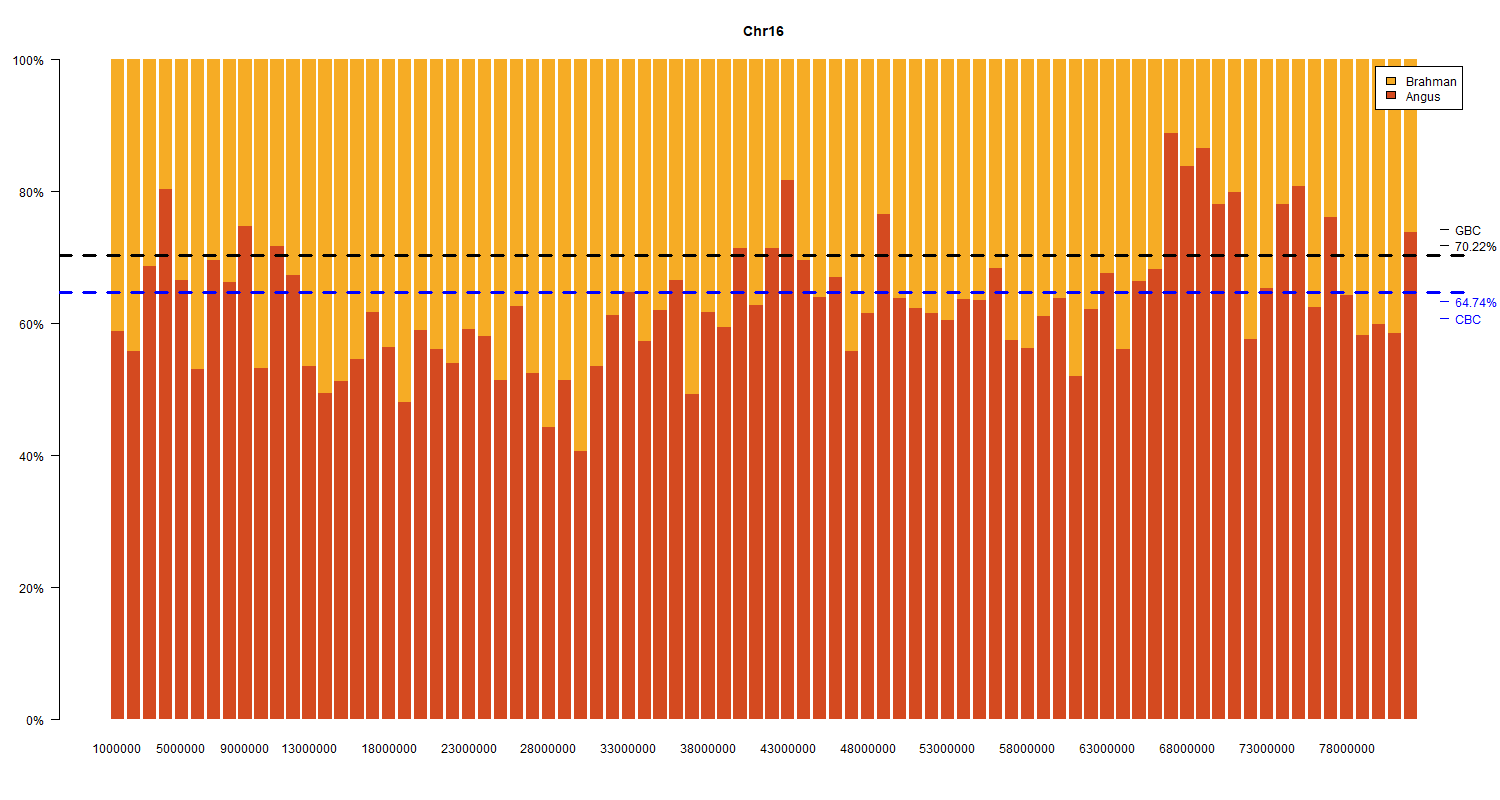

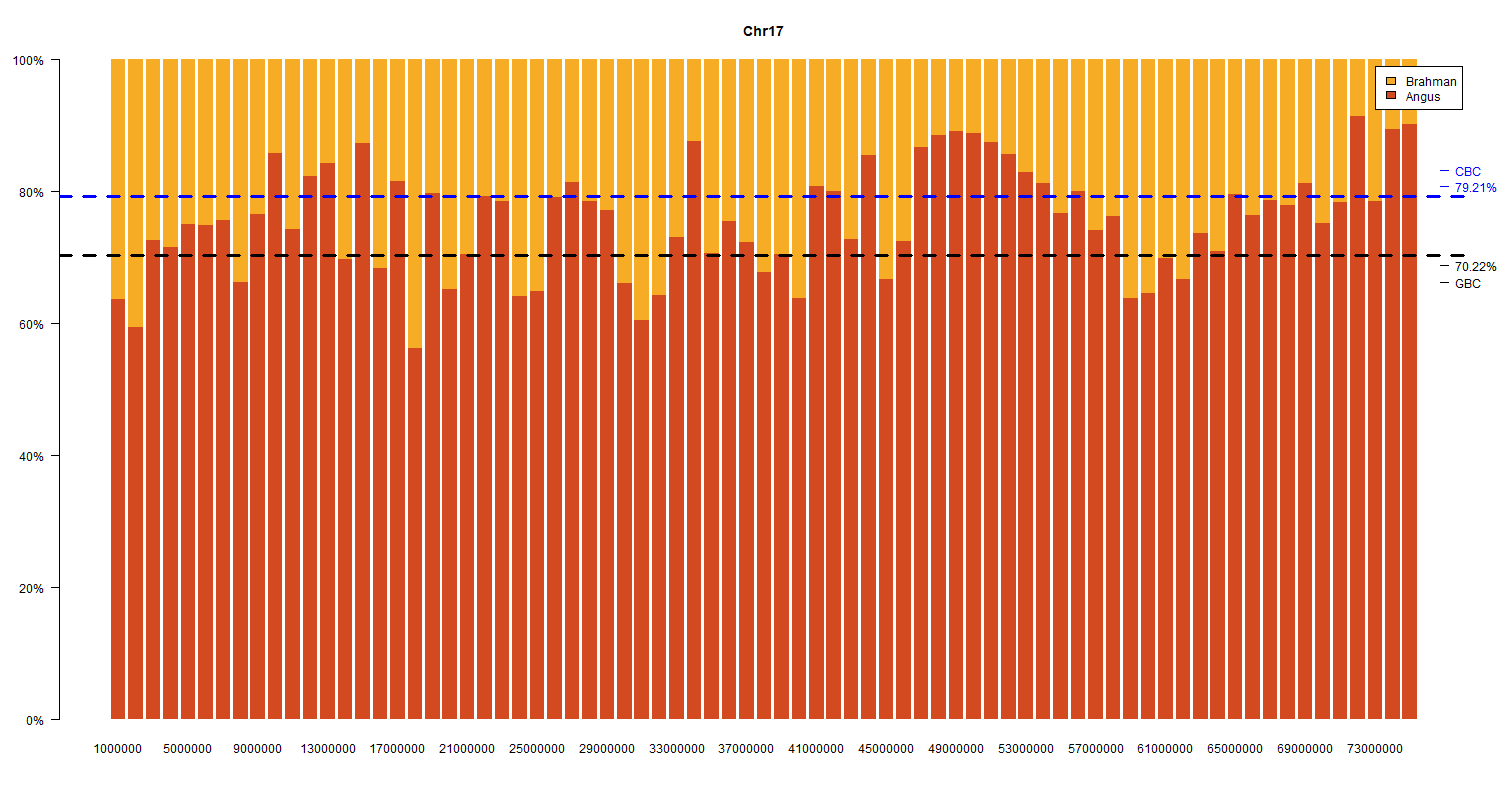

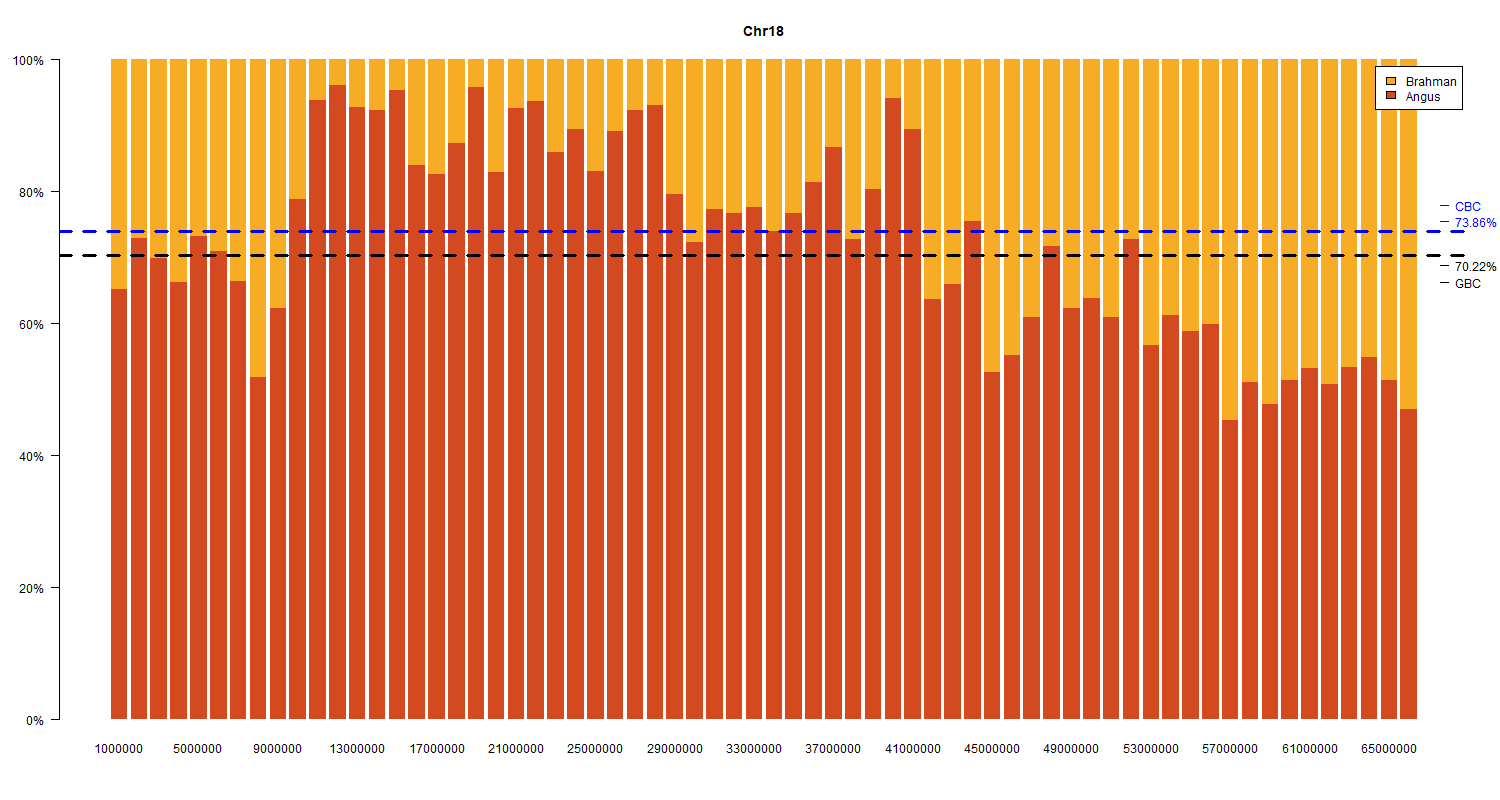

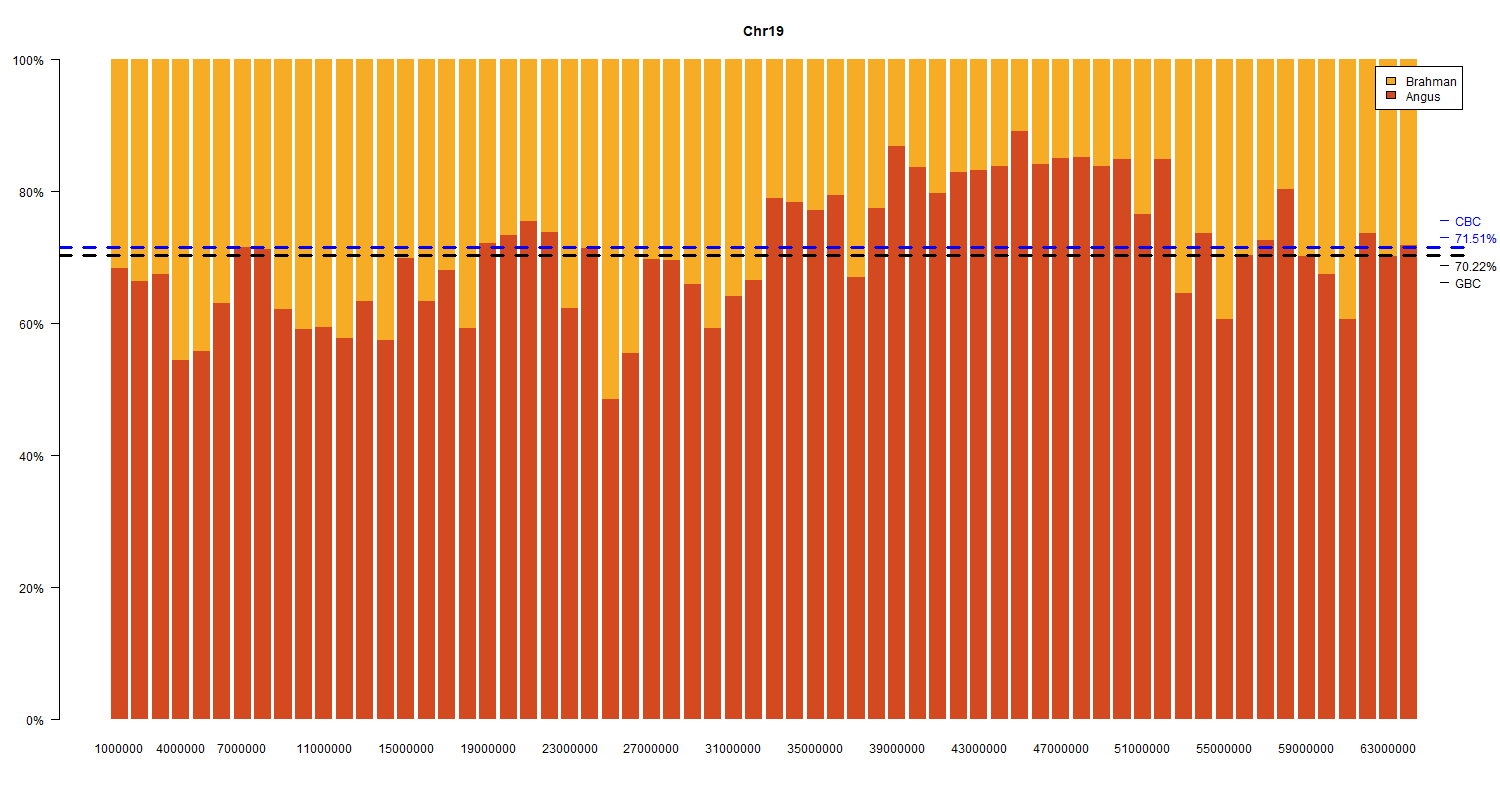

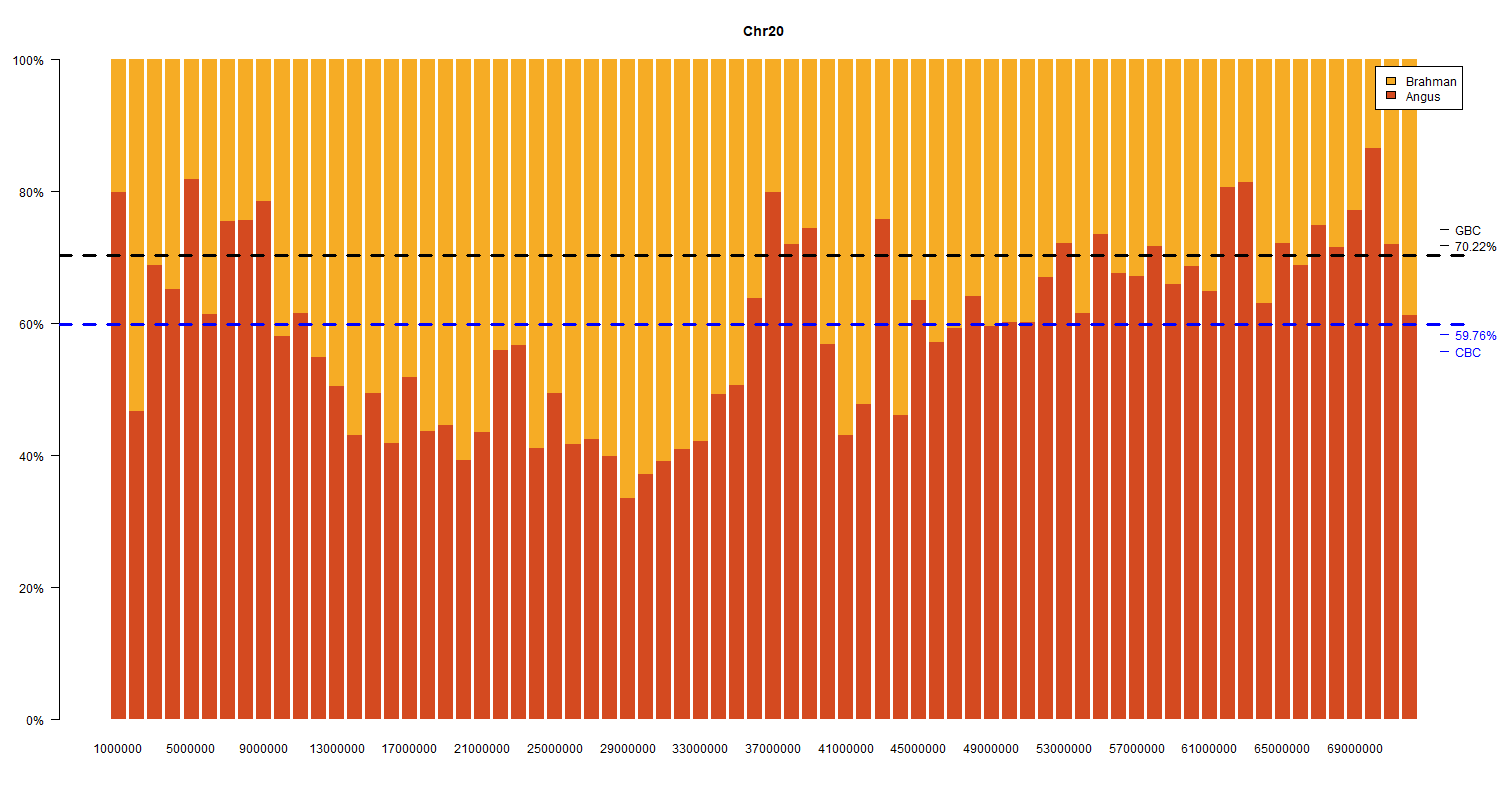

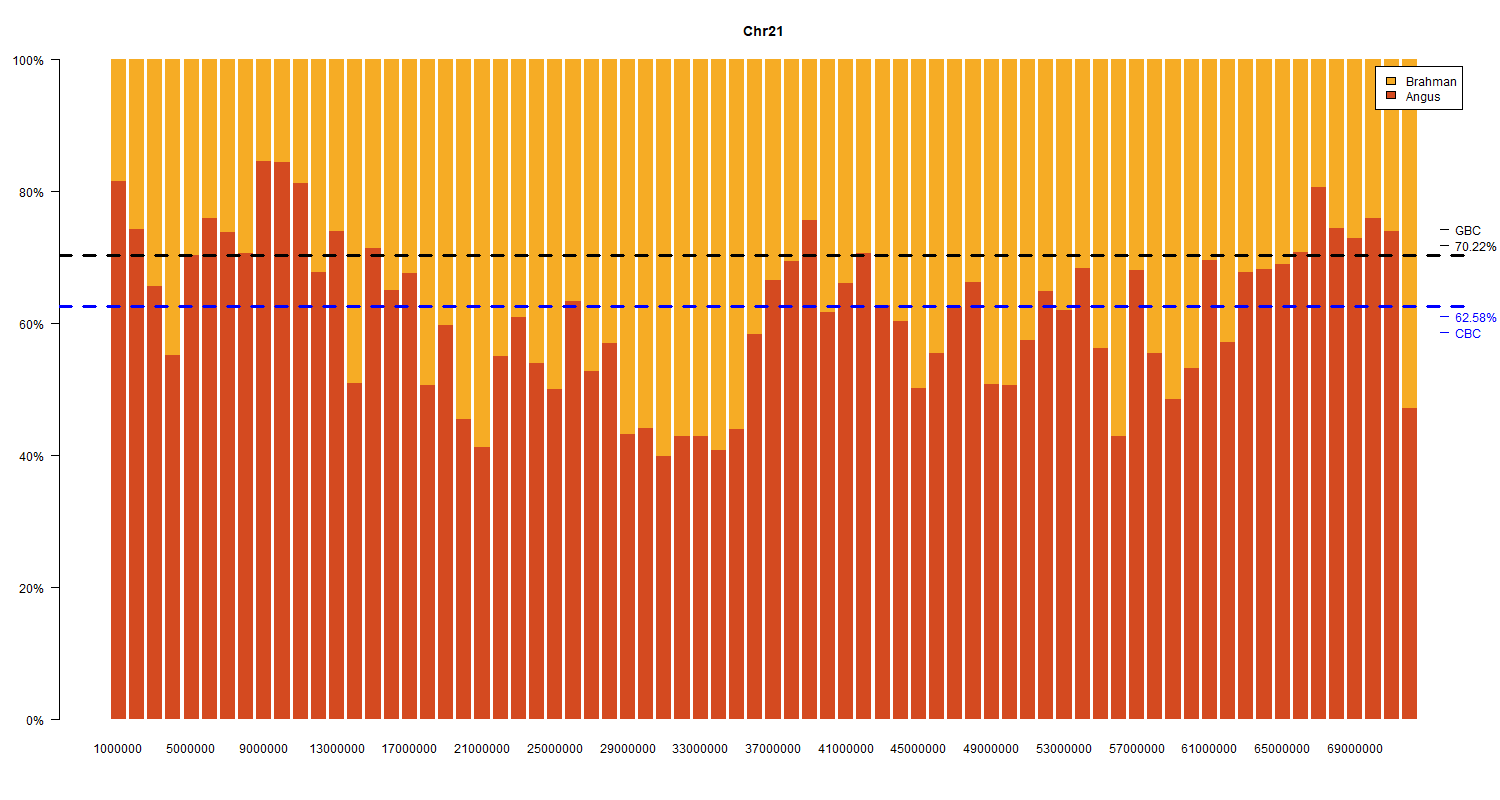

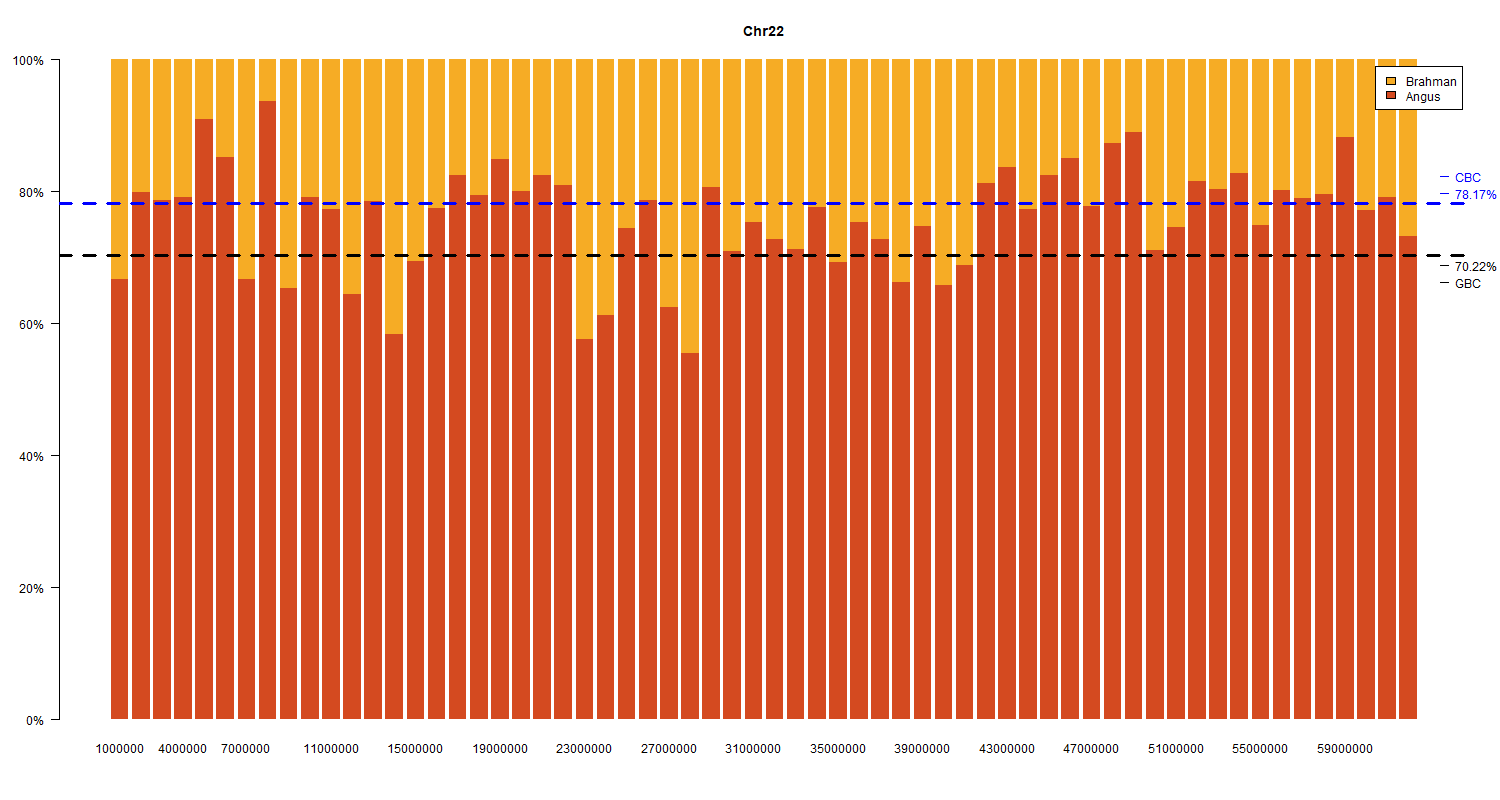

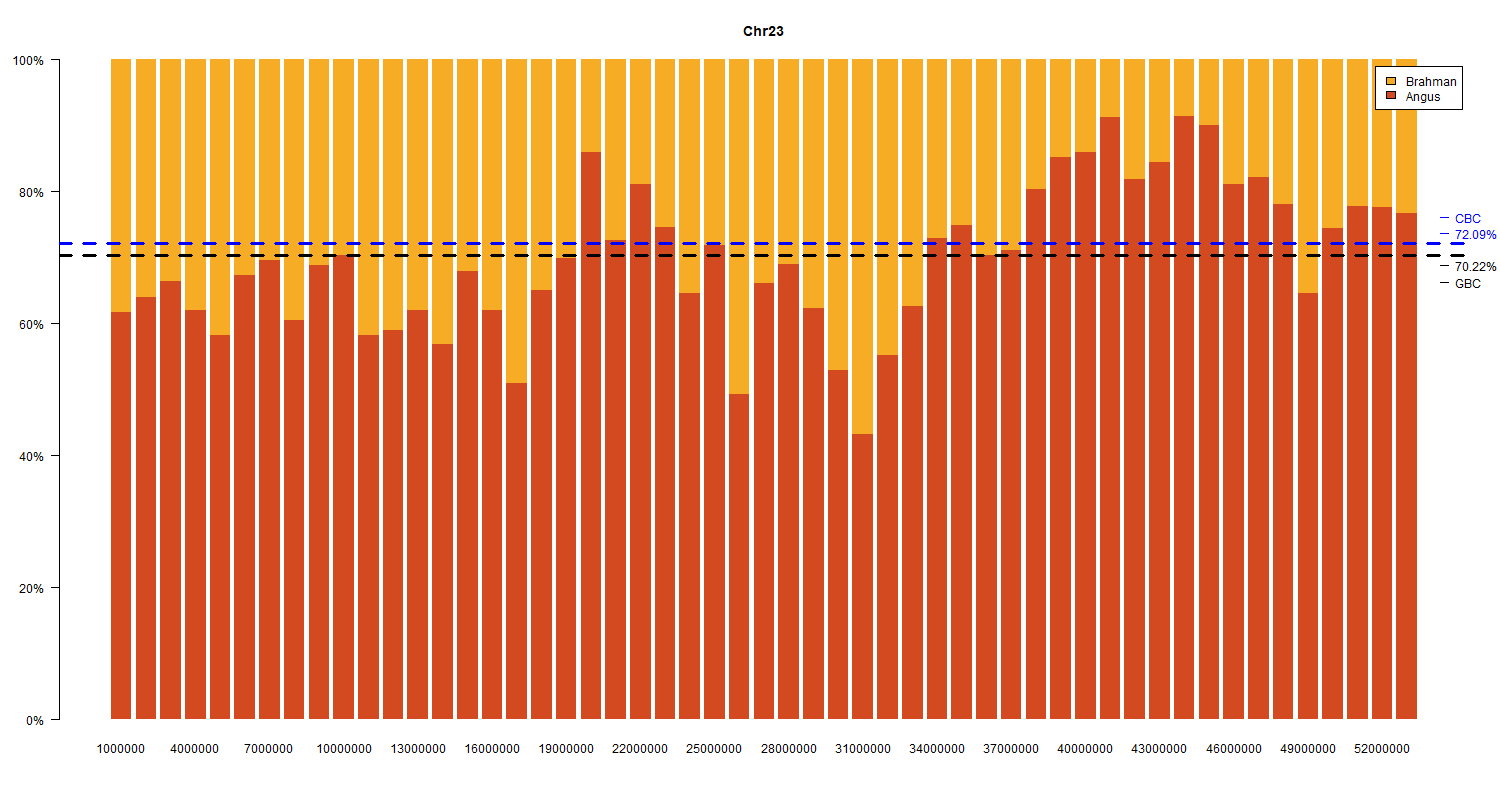

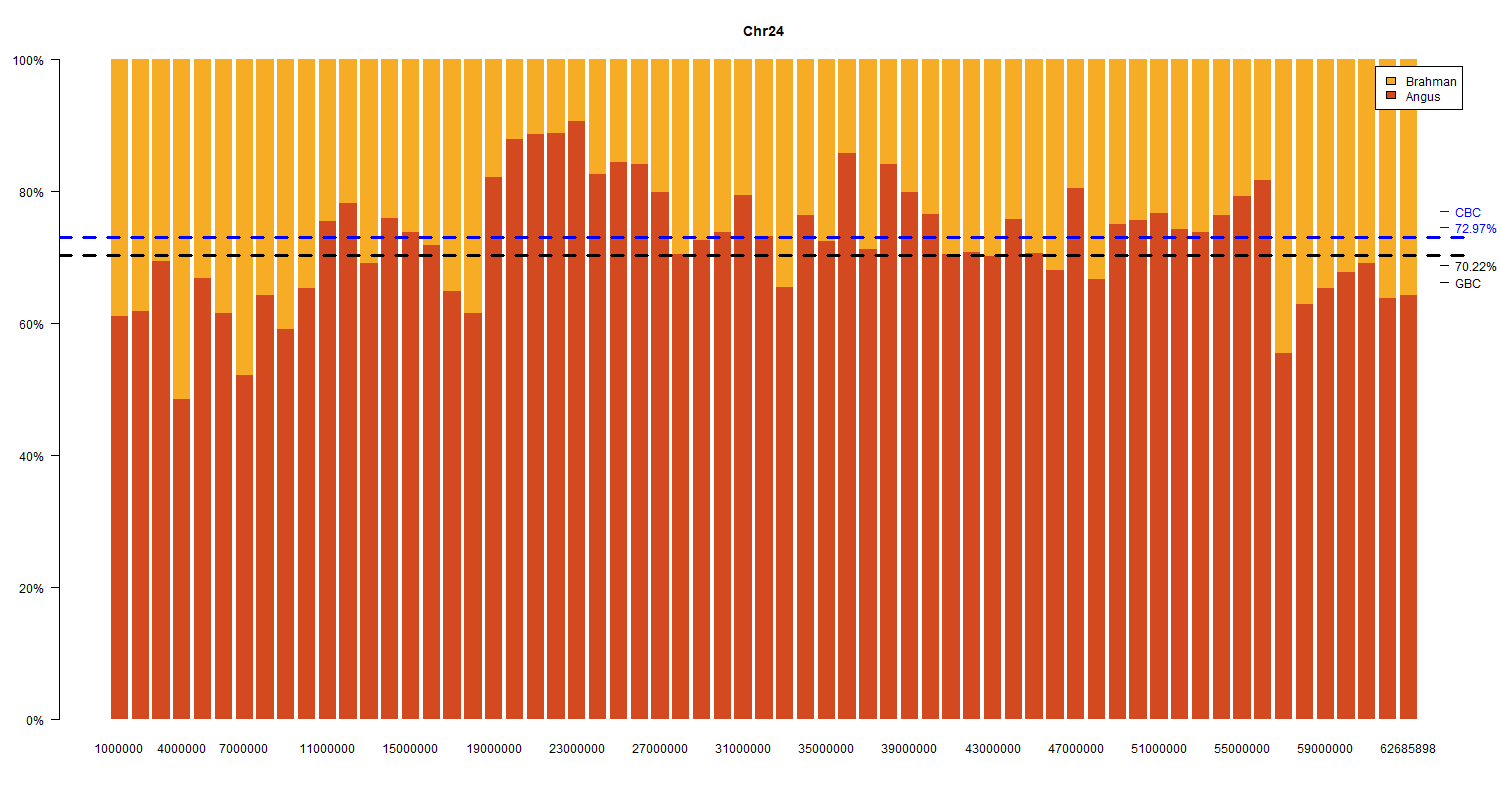

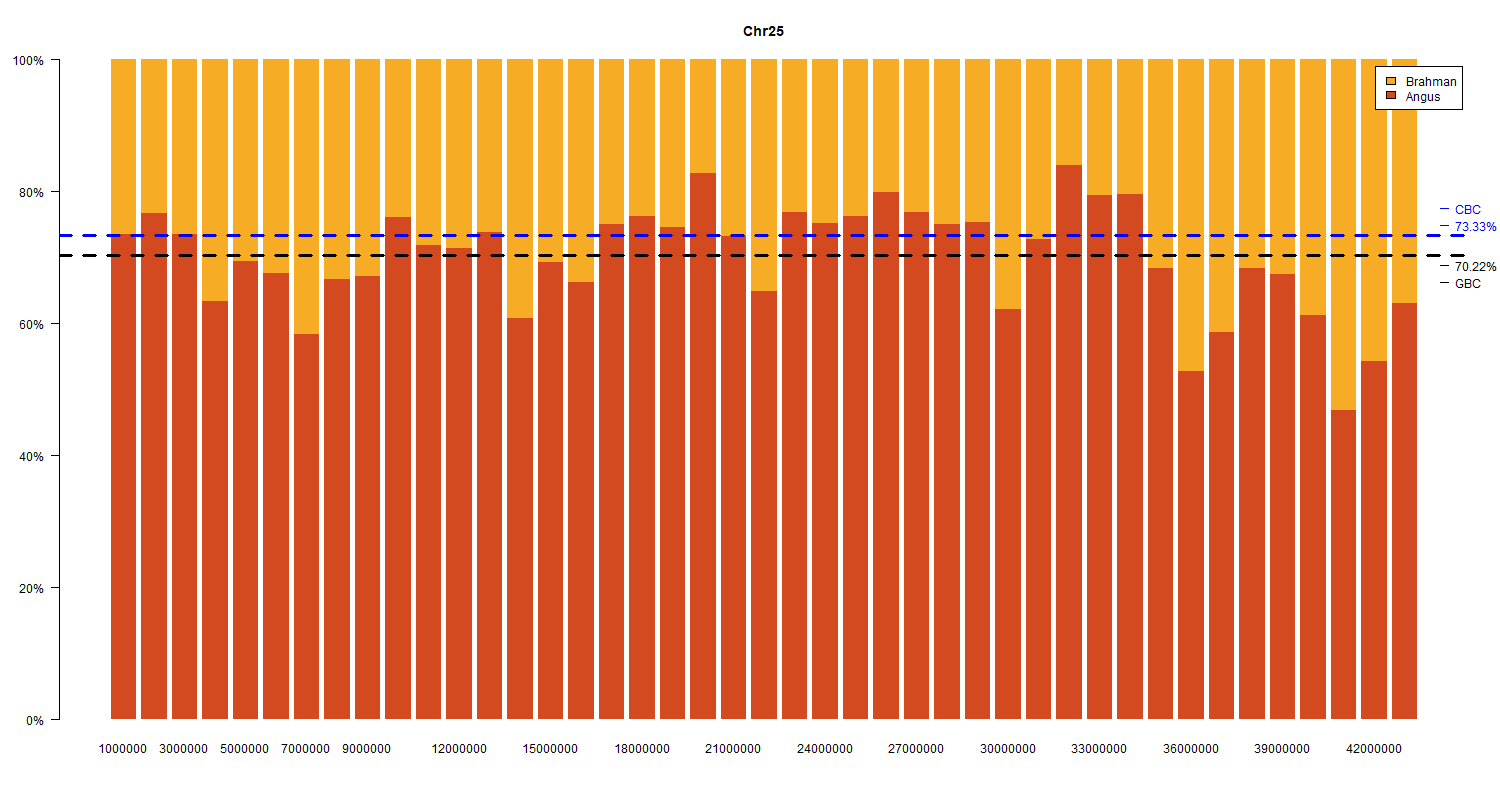

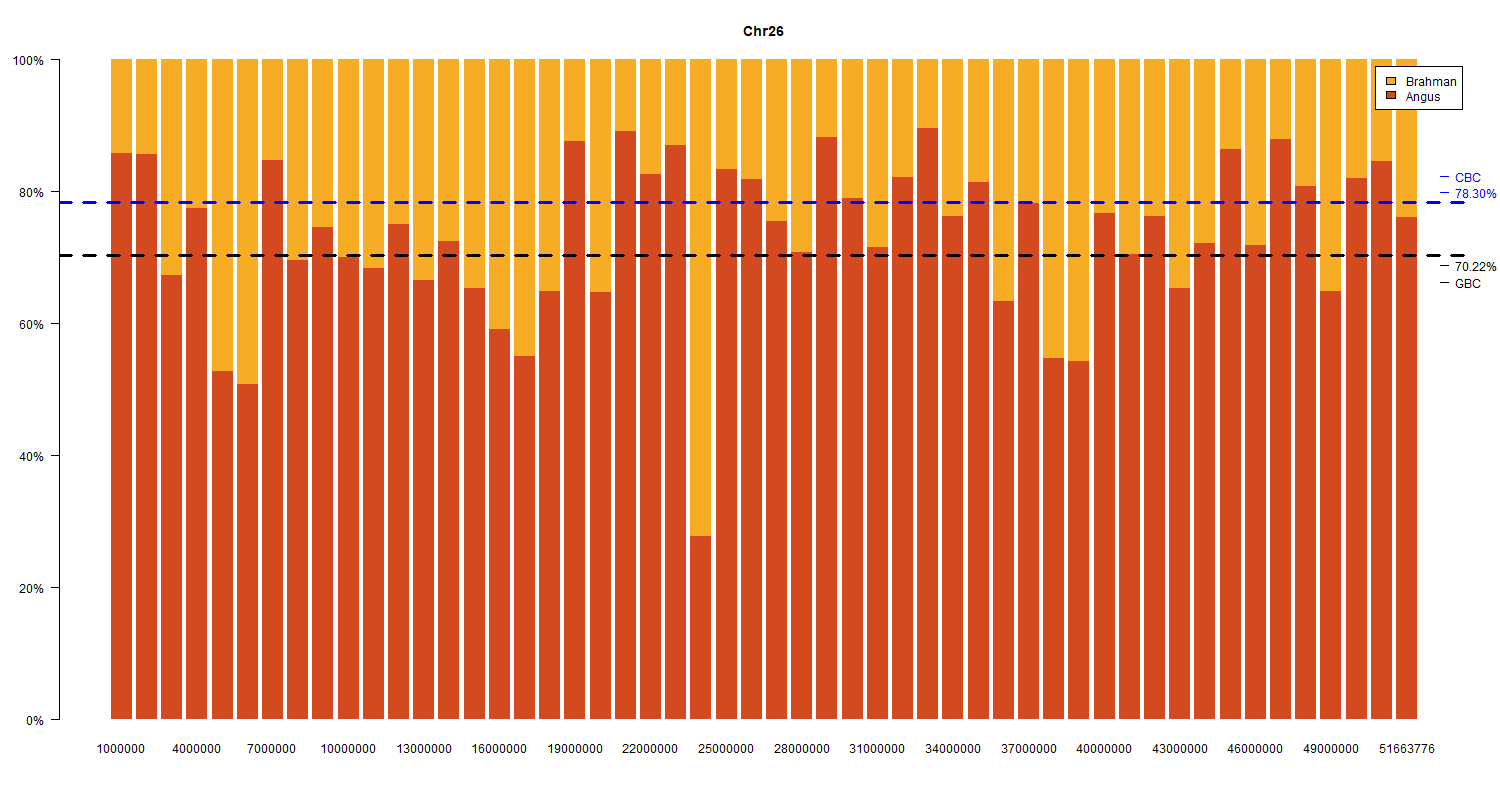

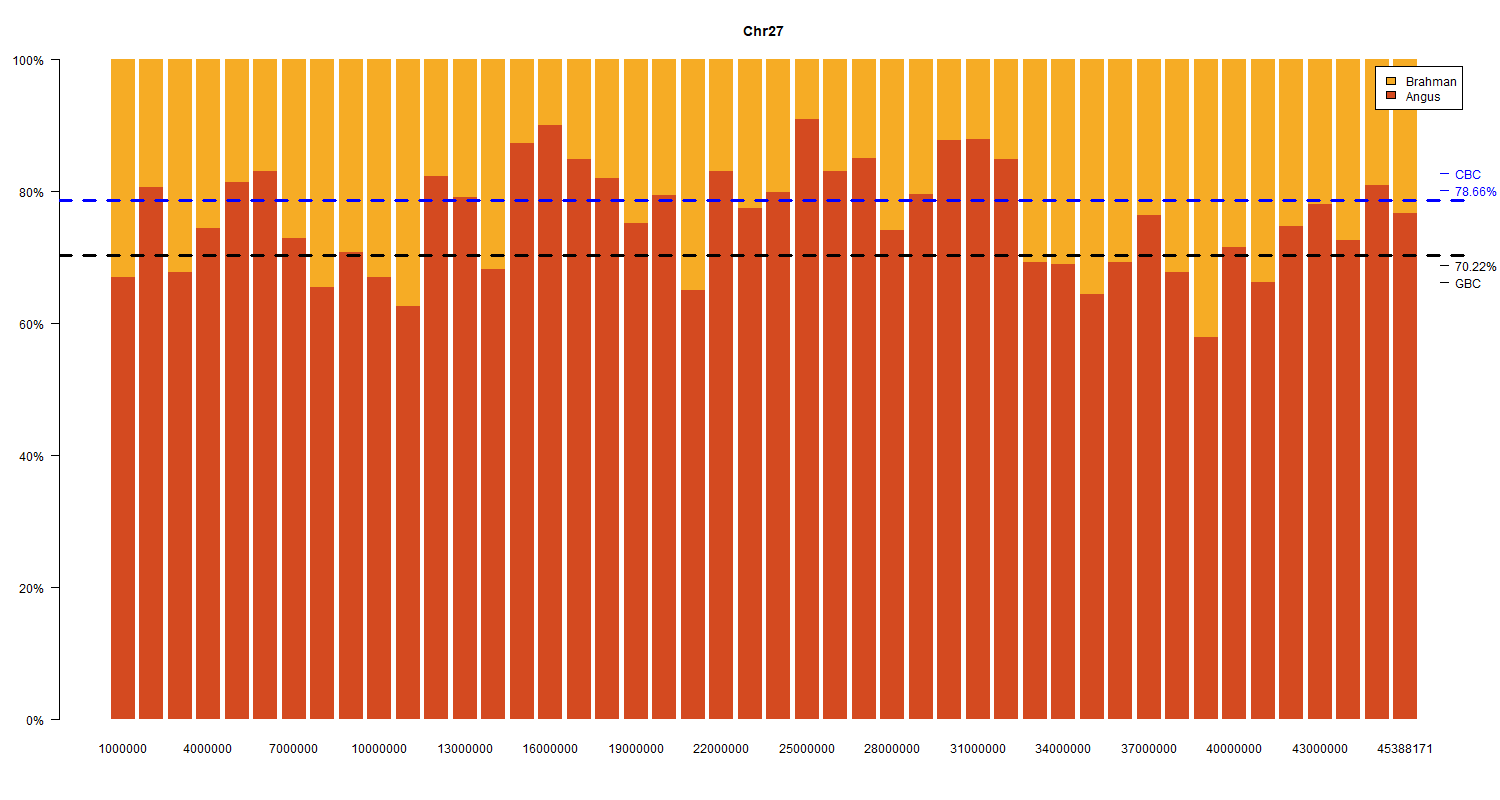

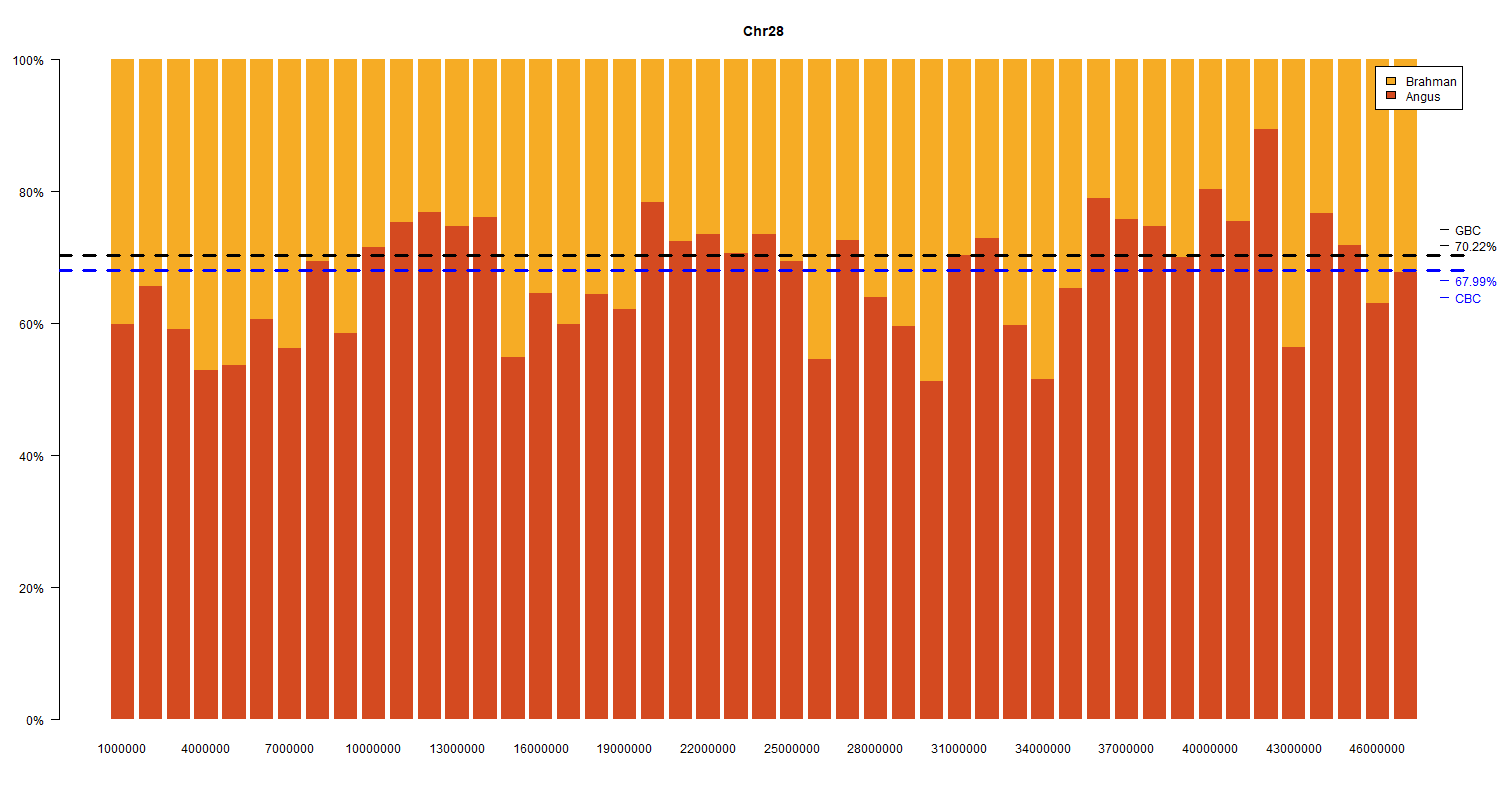

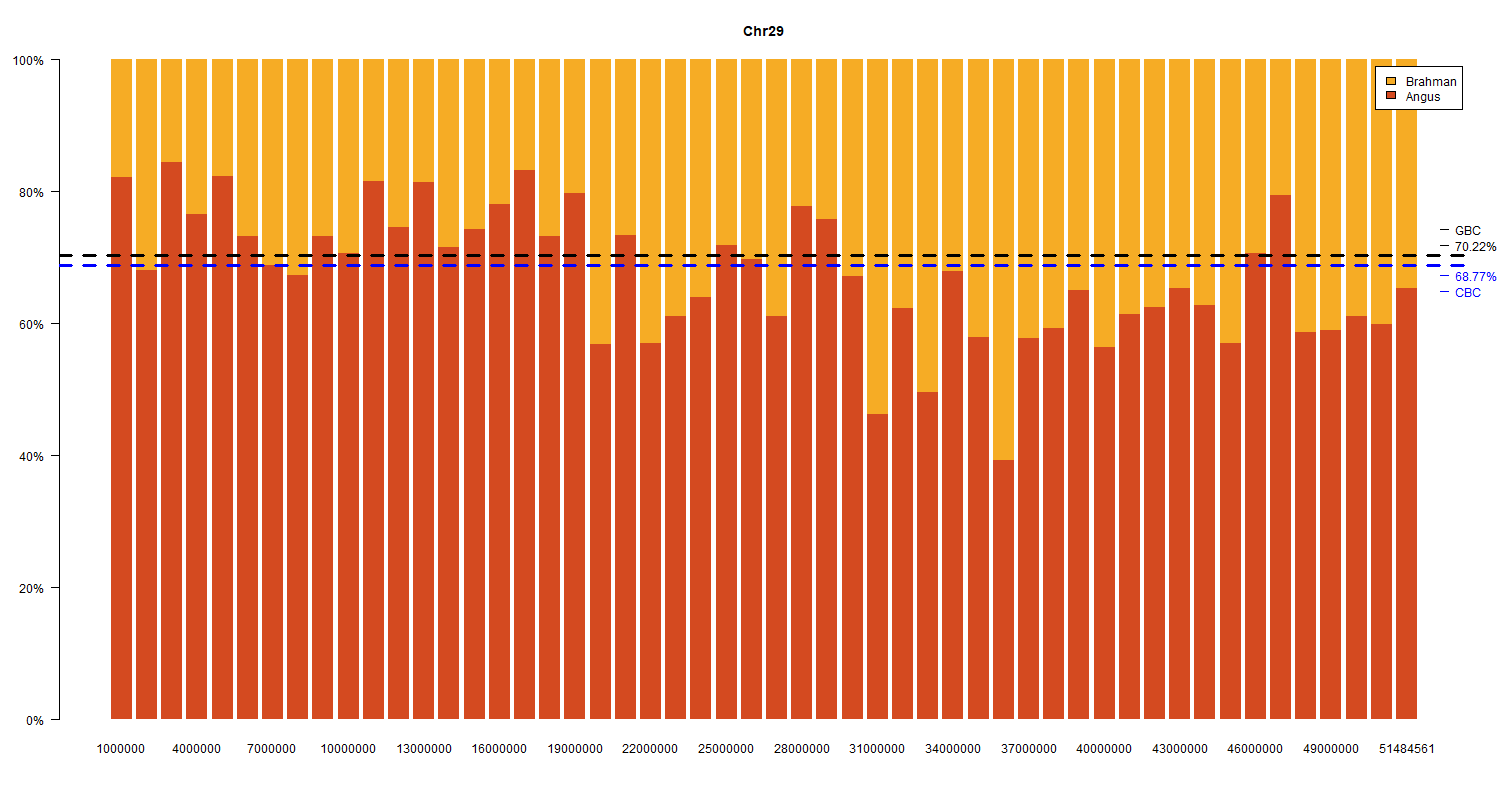


(B)


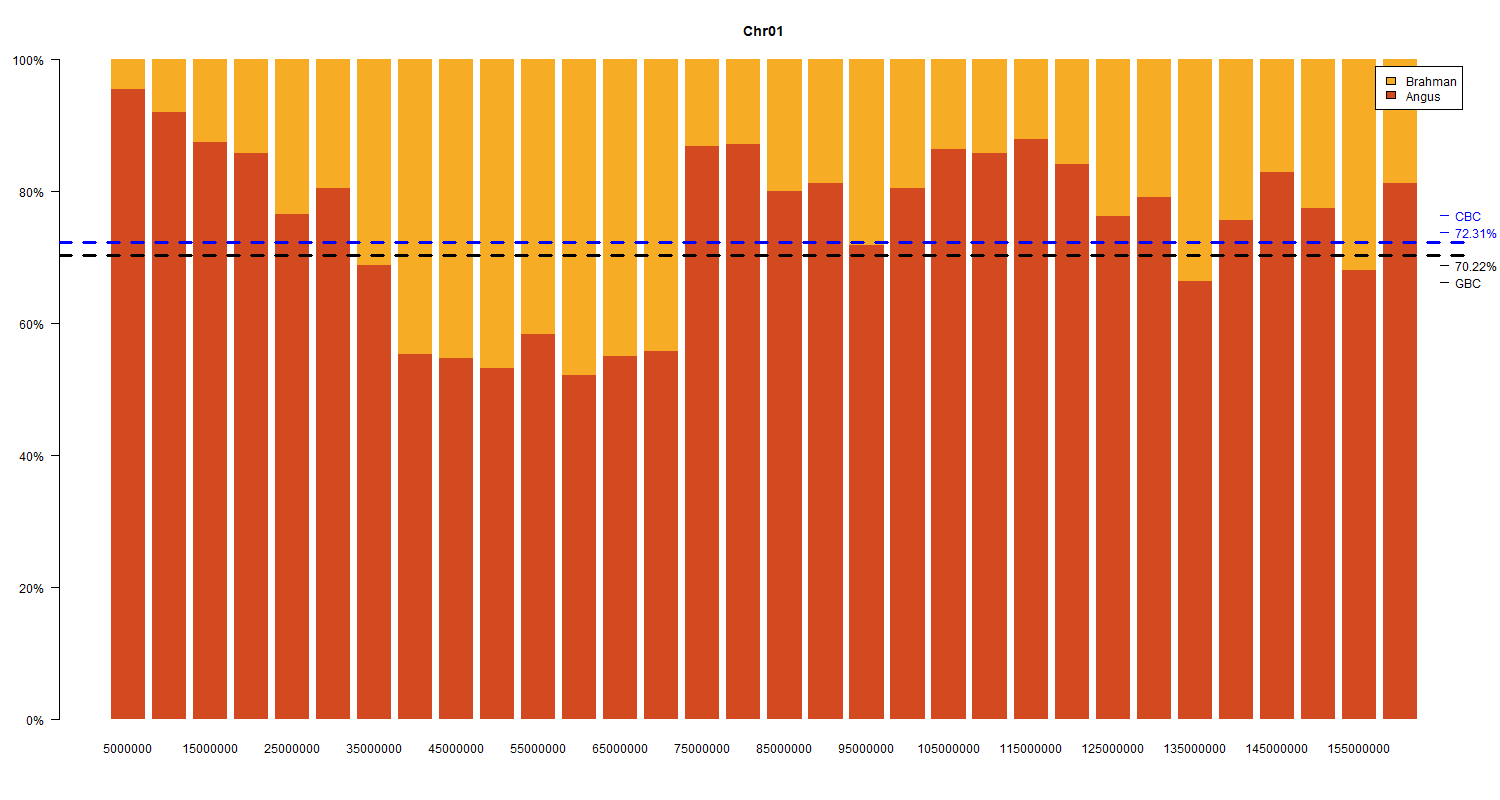

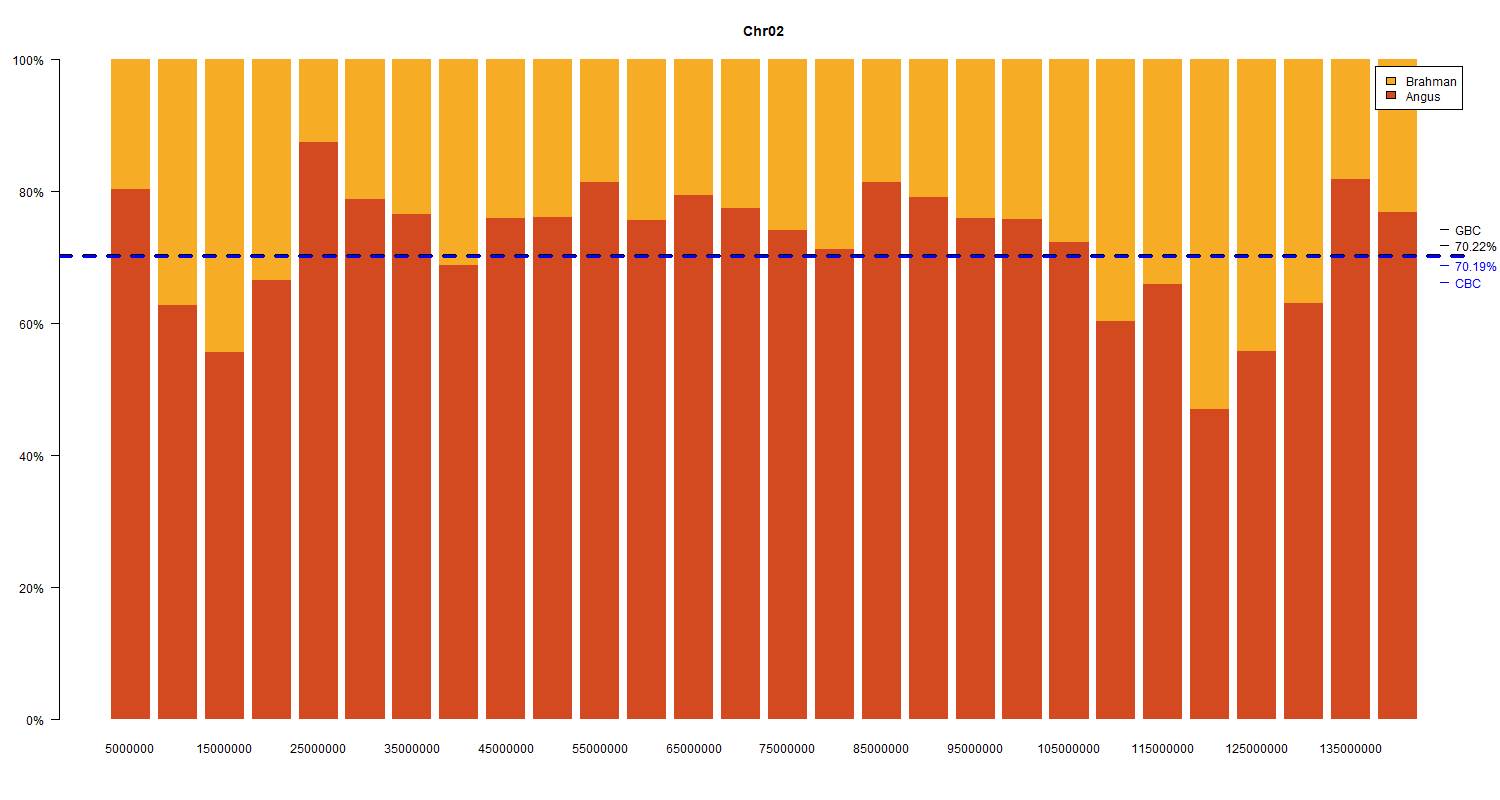

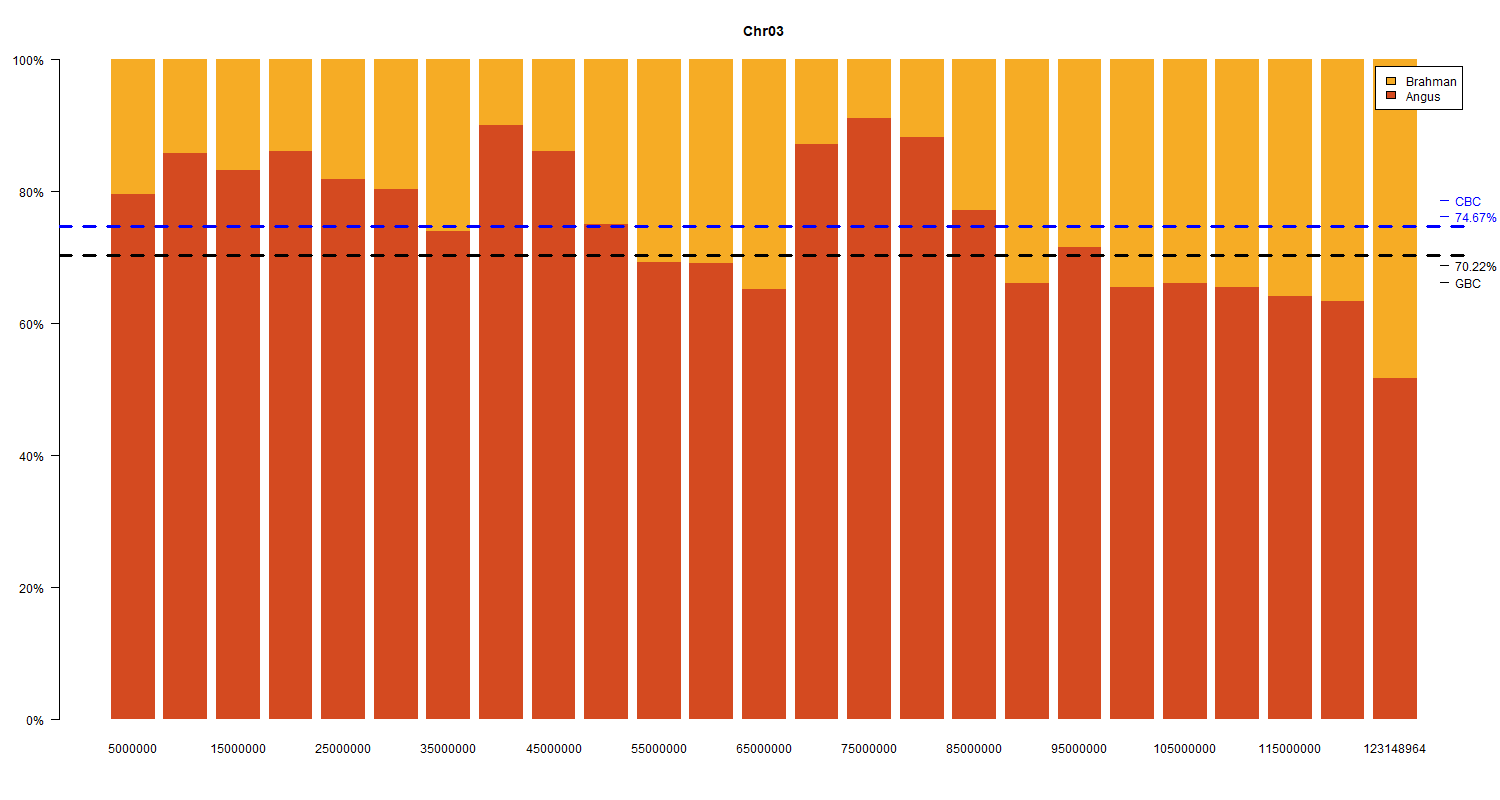

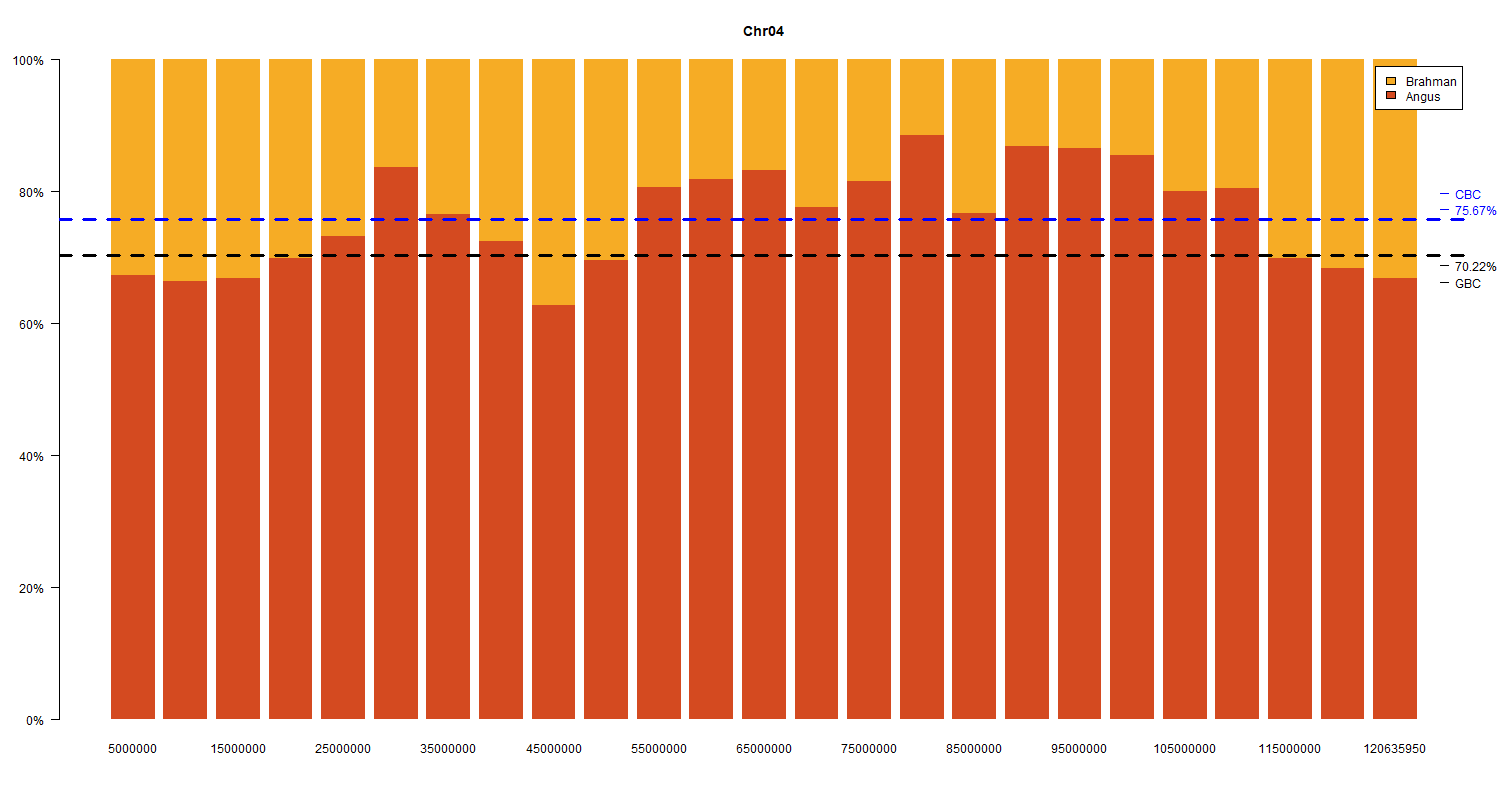

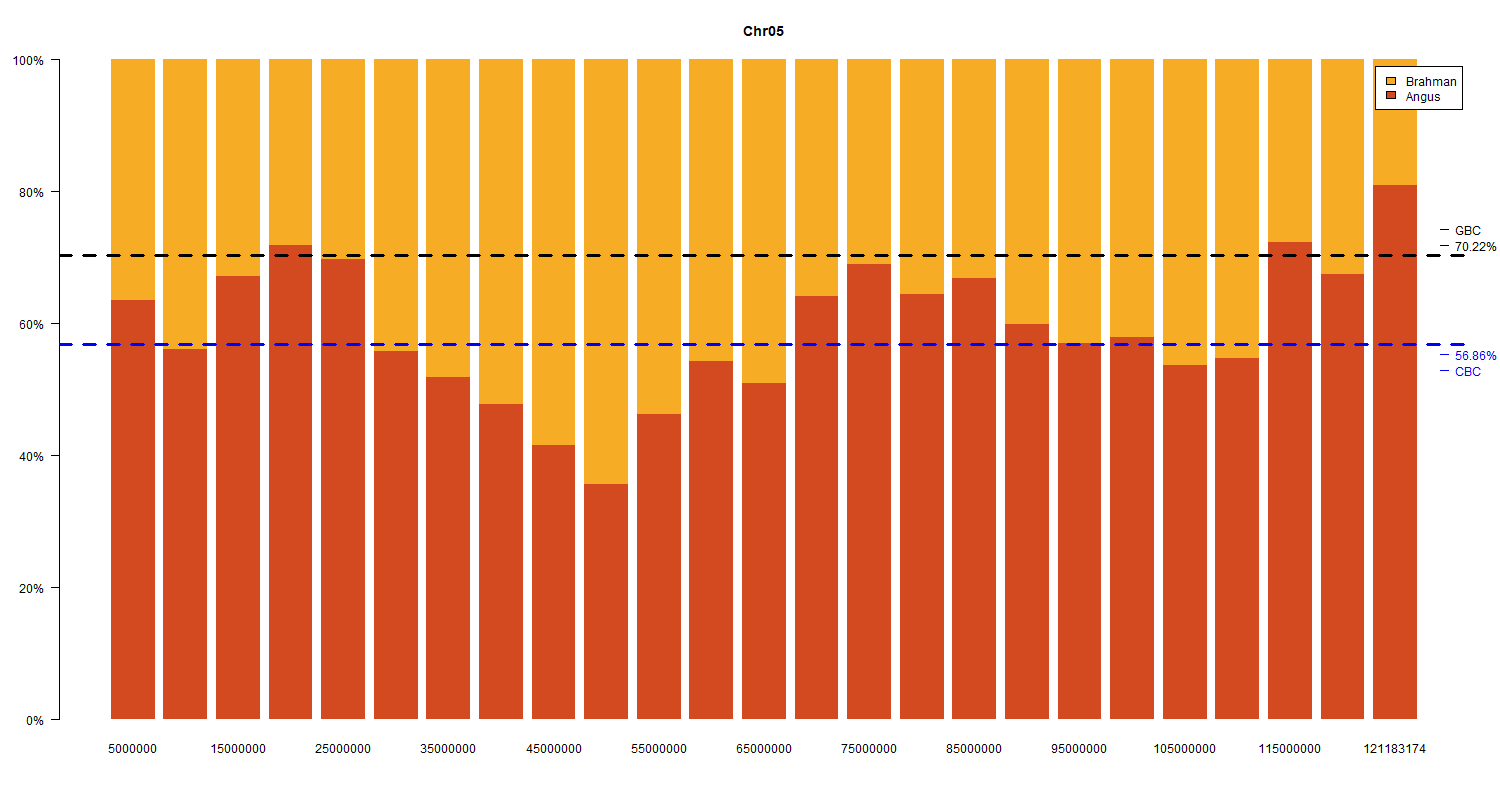

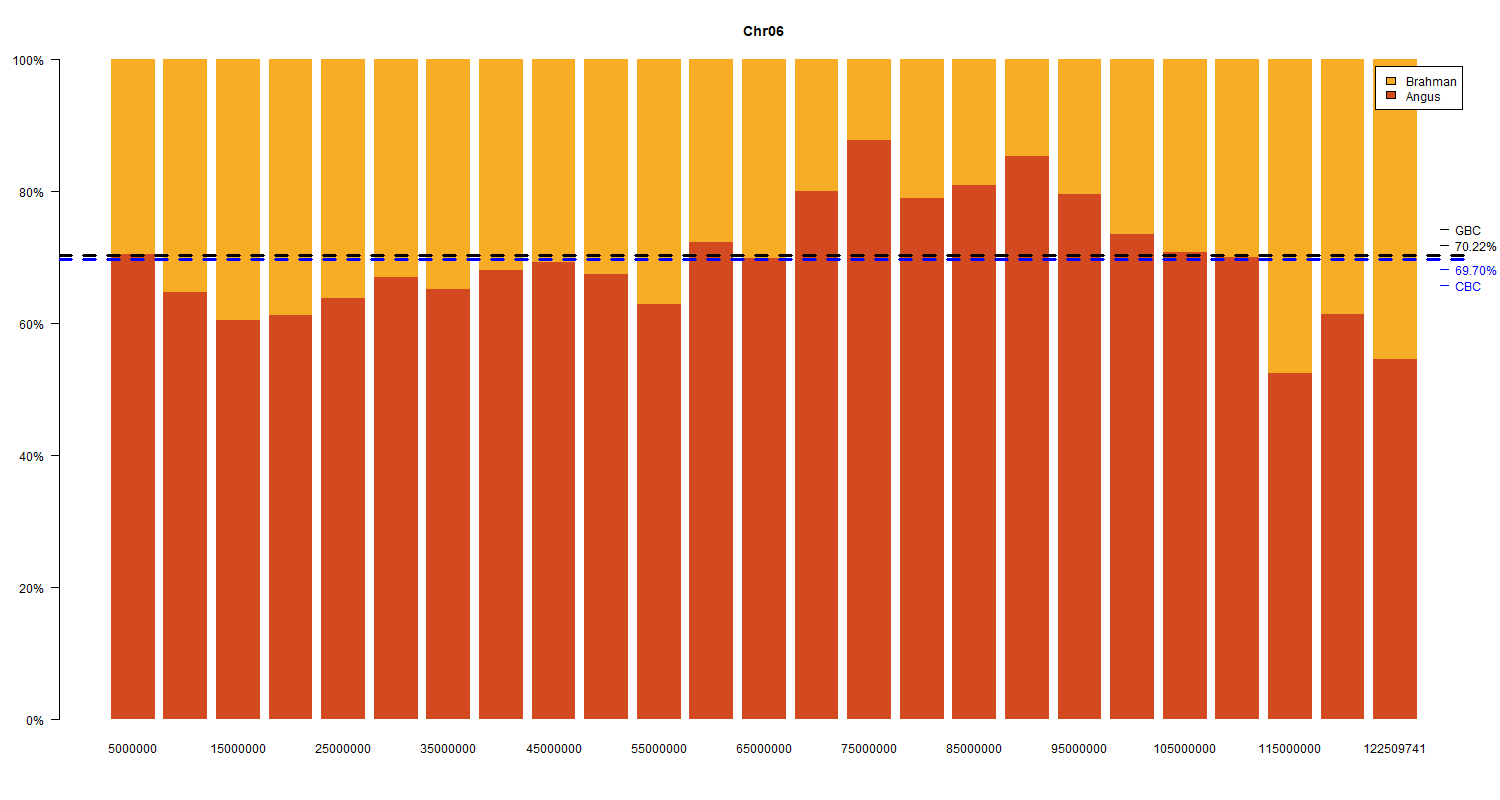

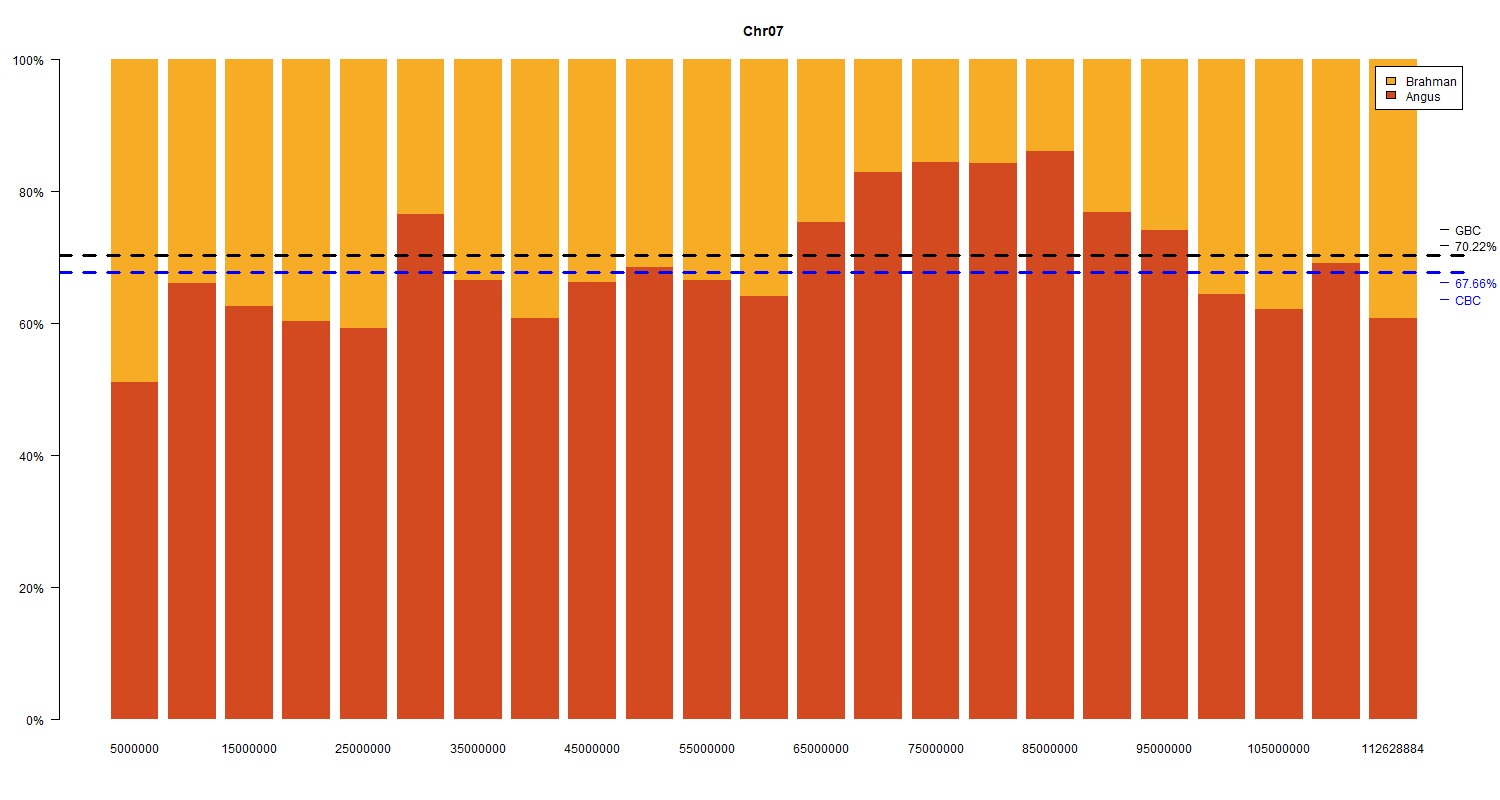

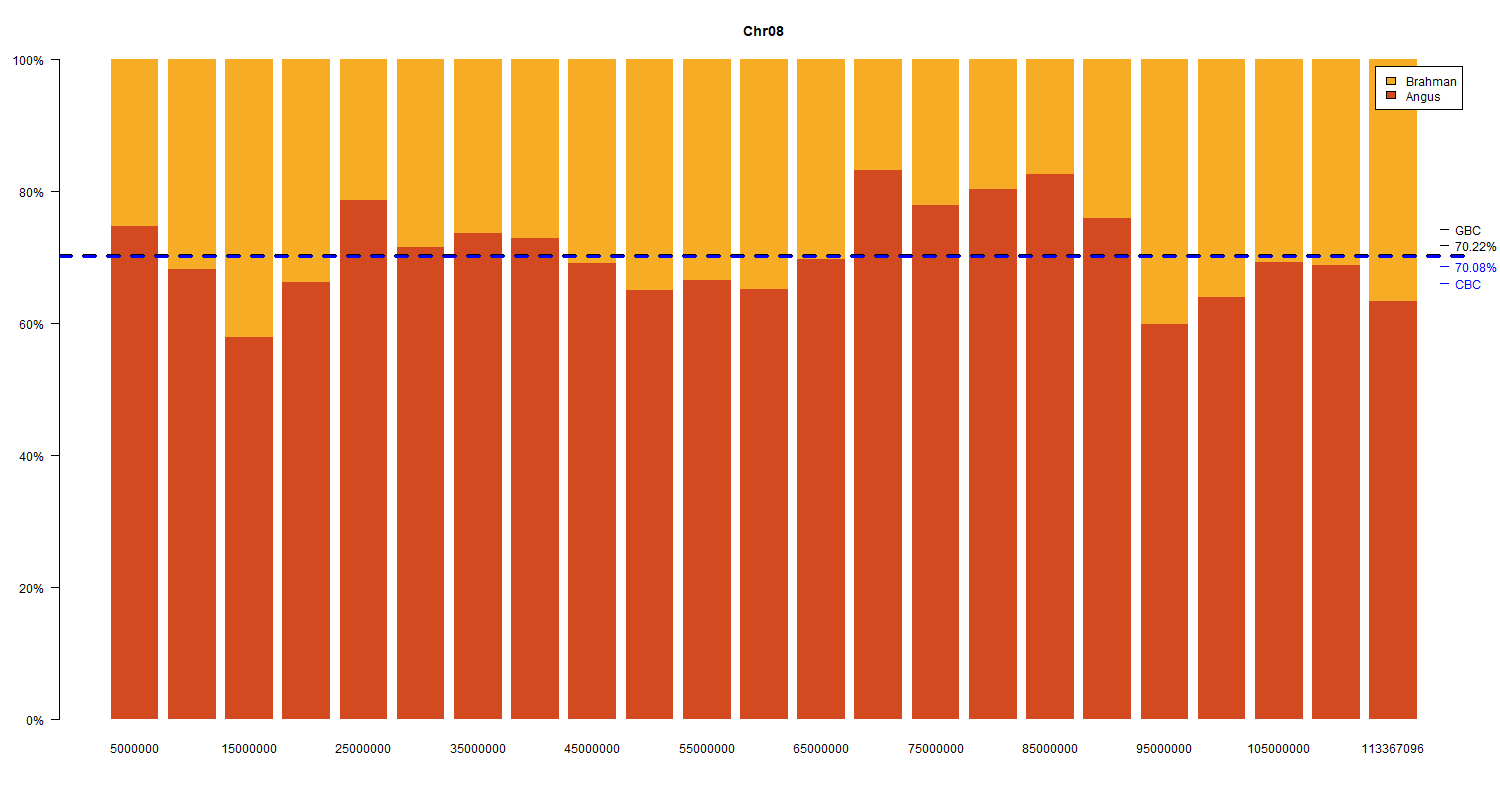

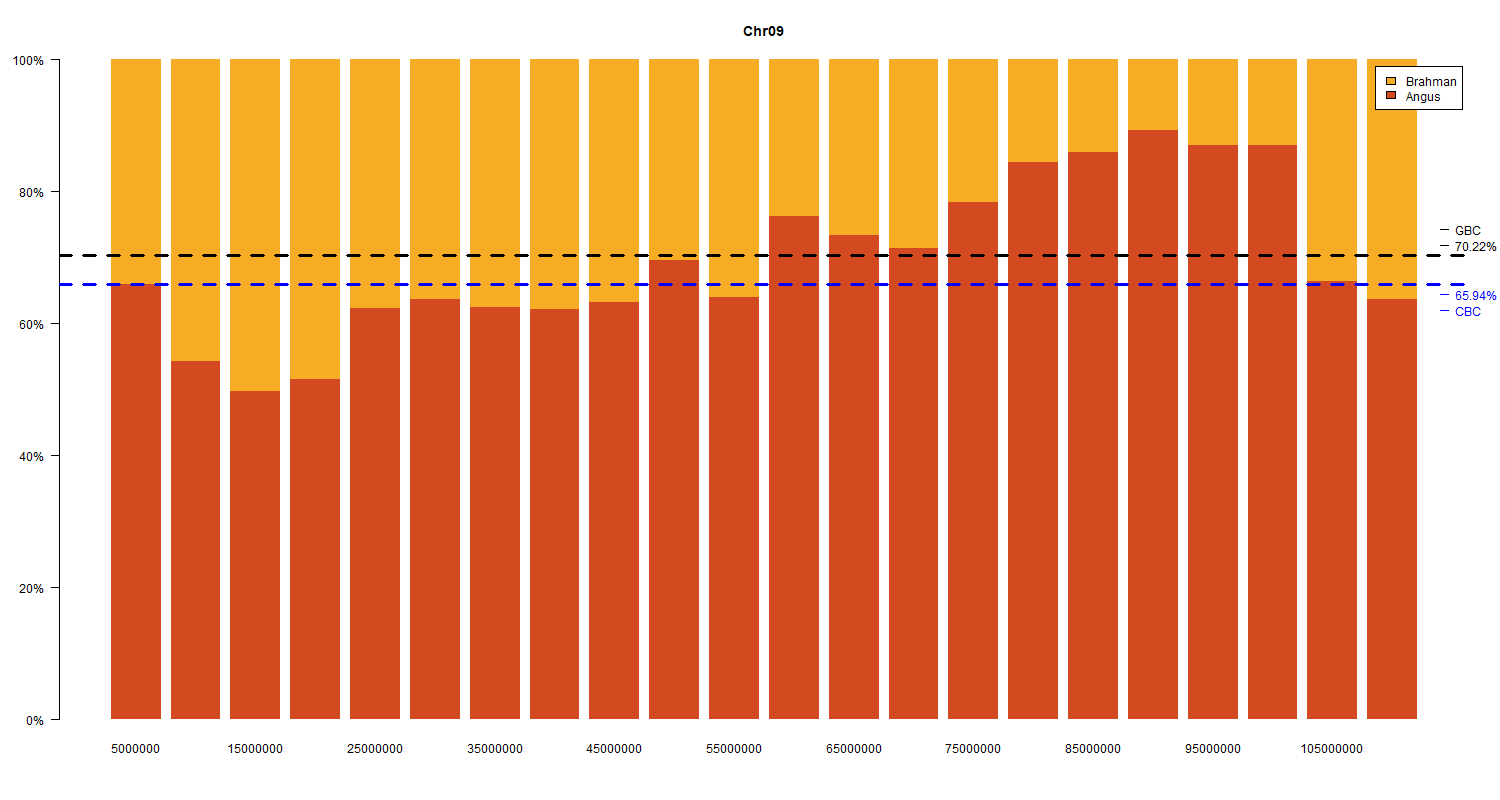

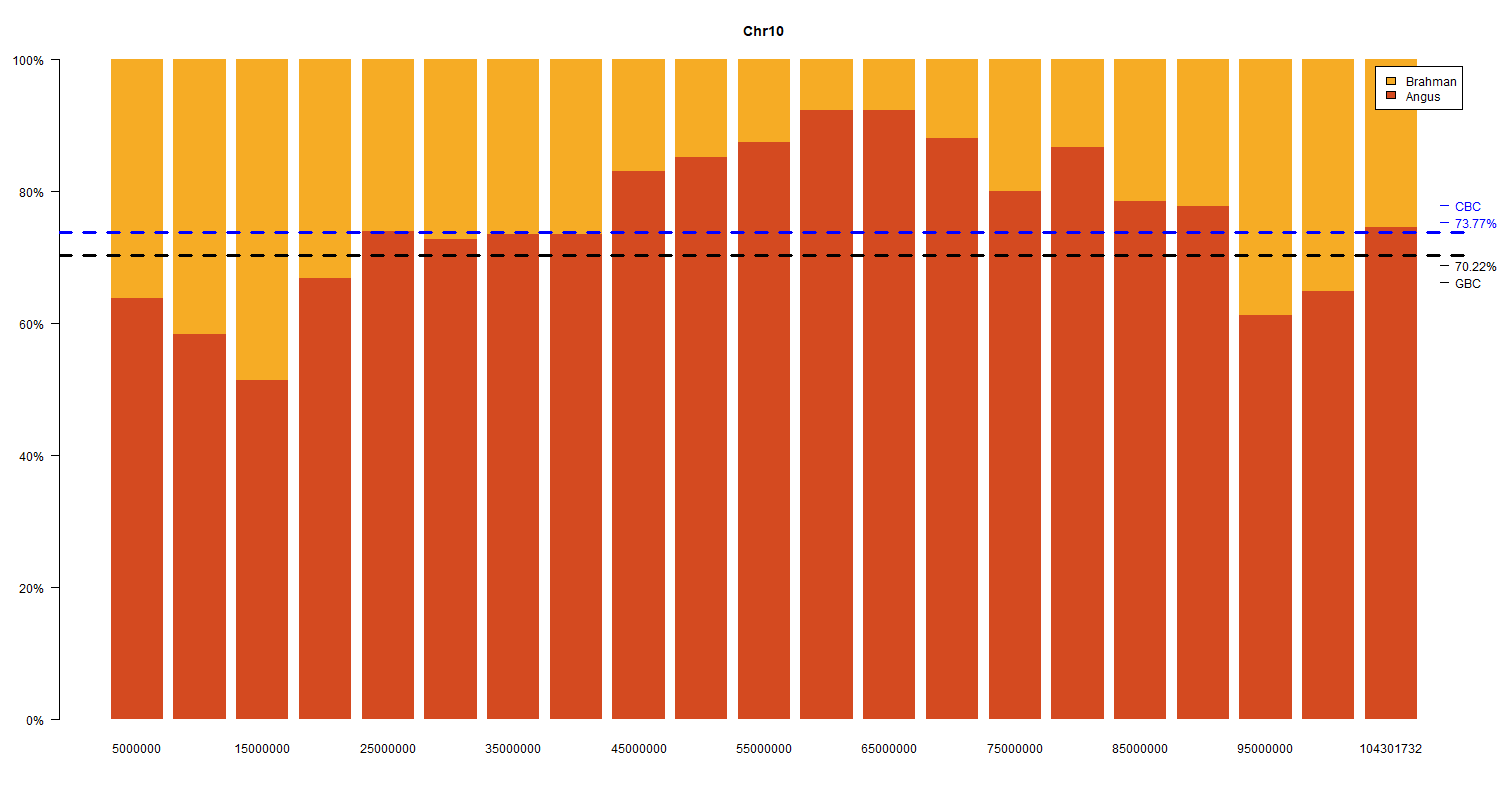

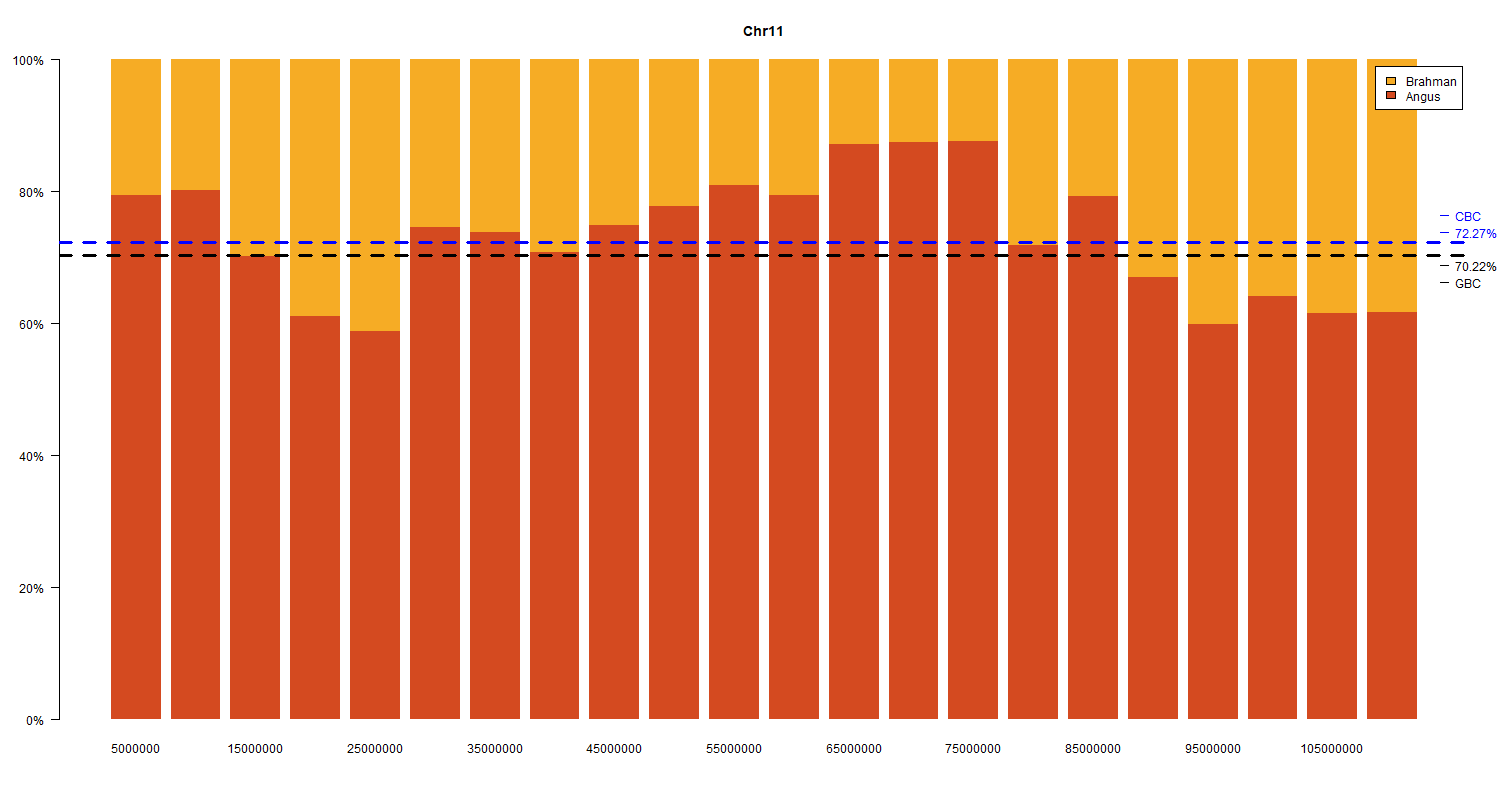

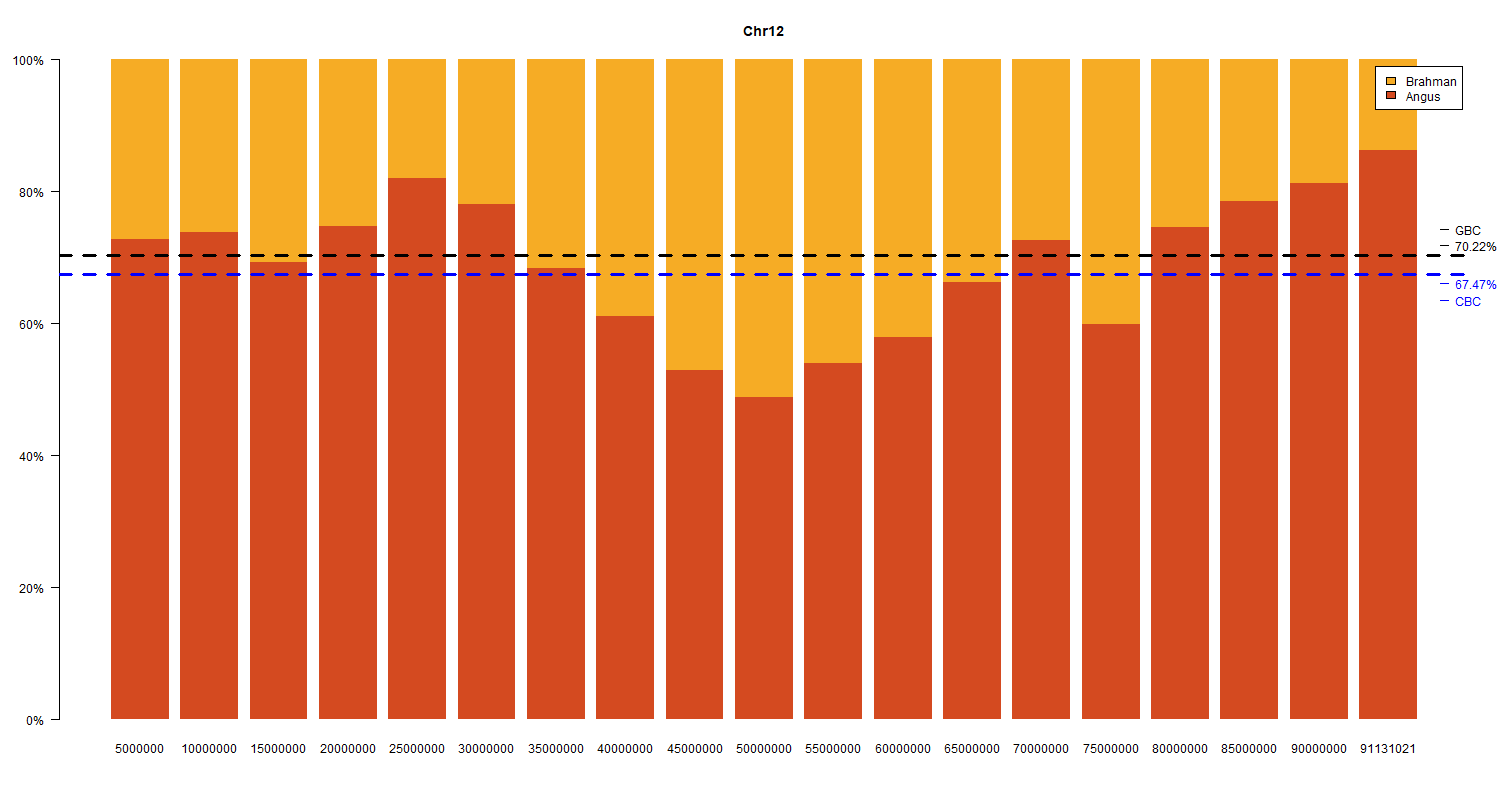

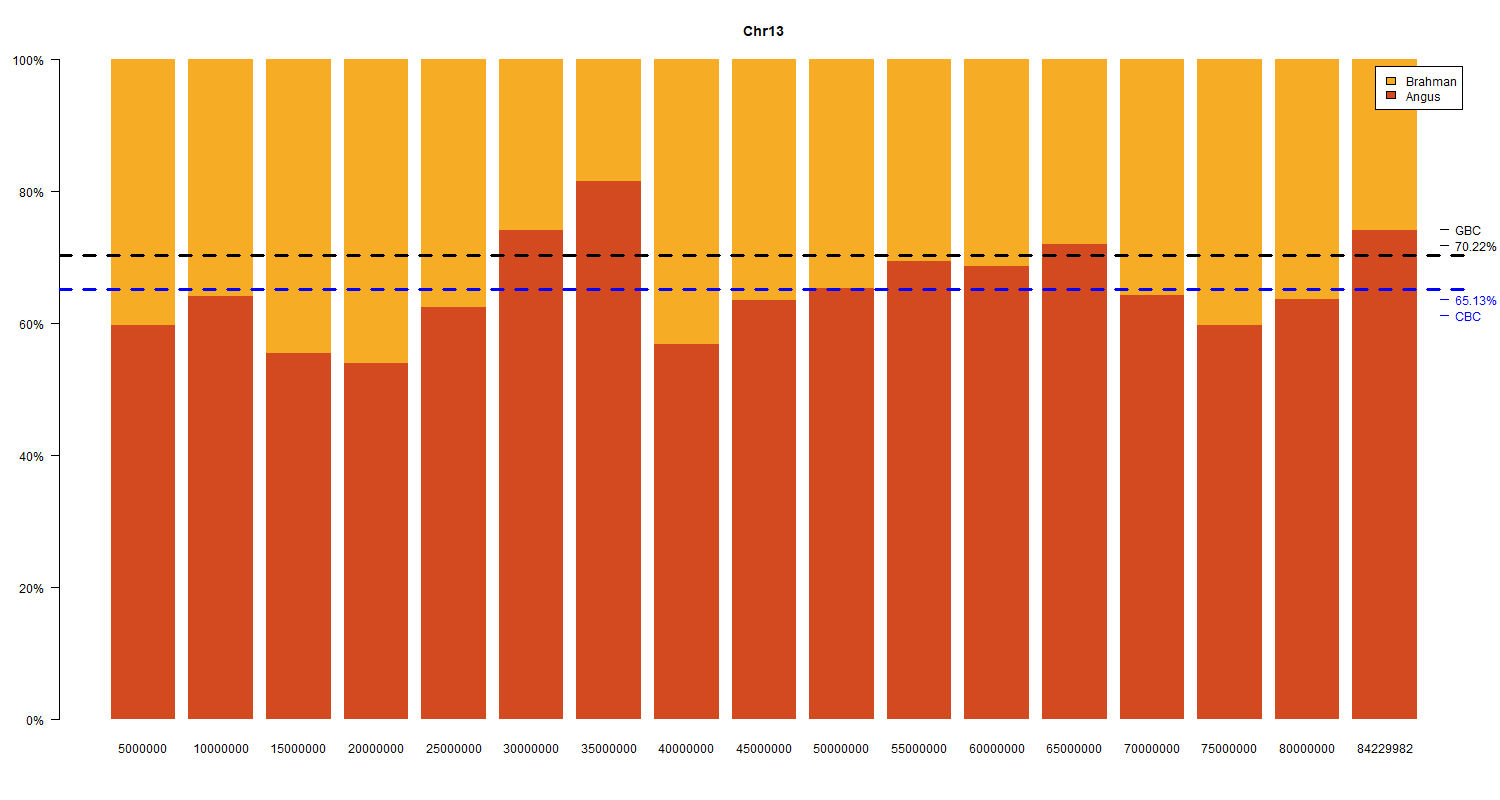

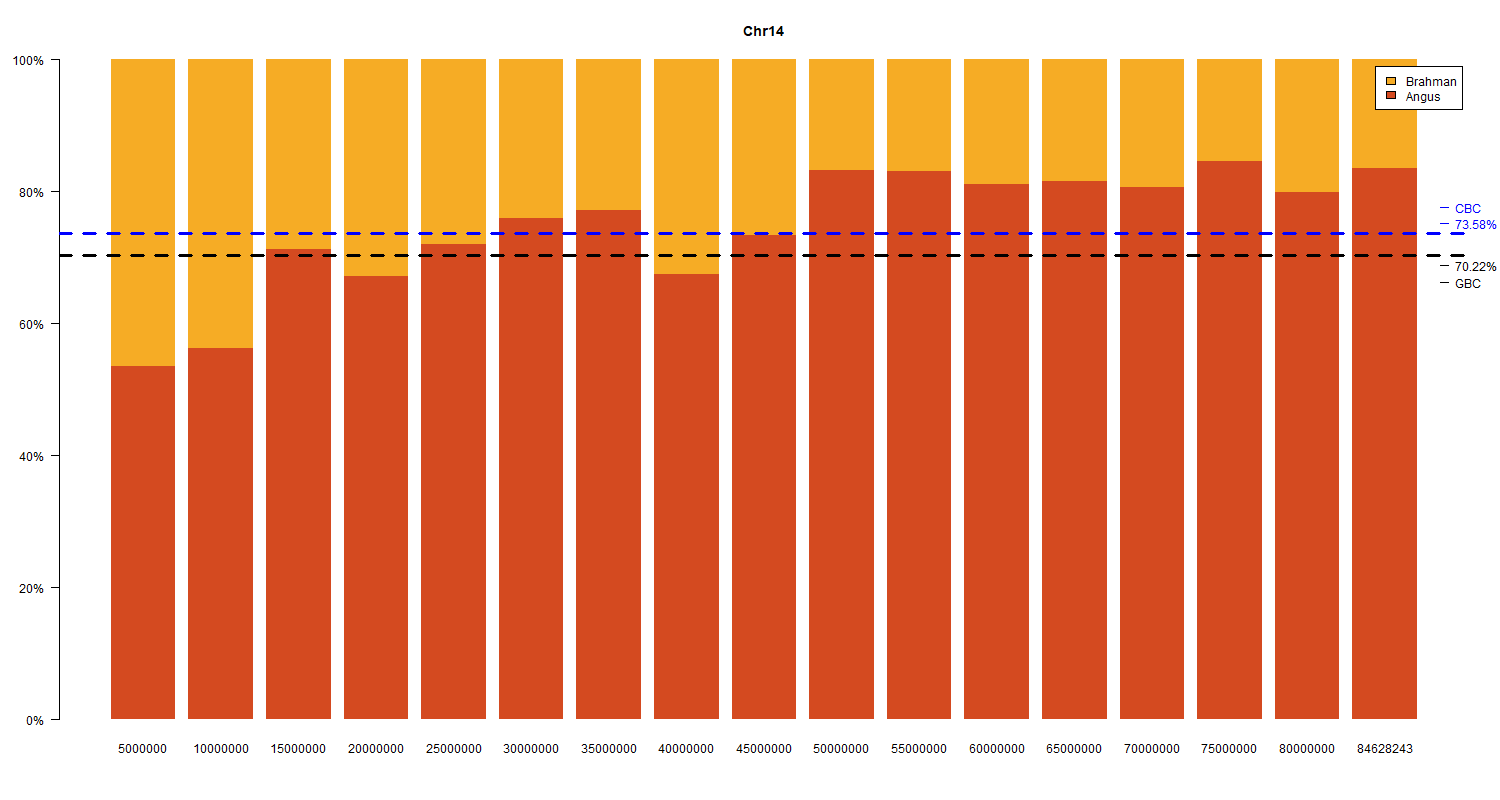

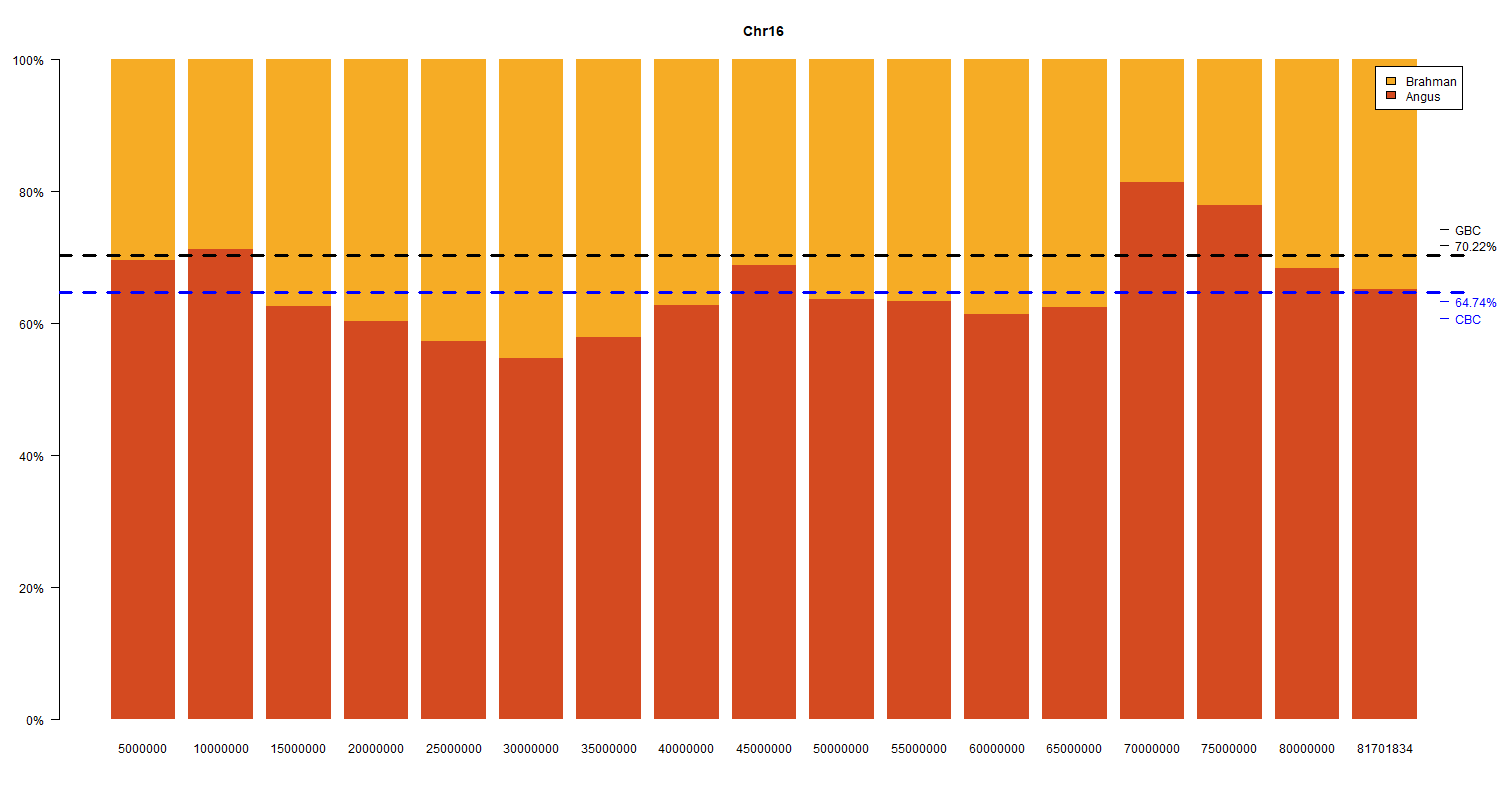

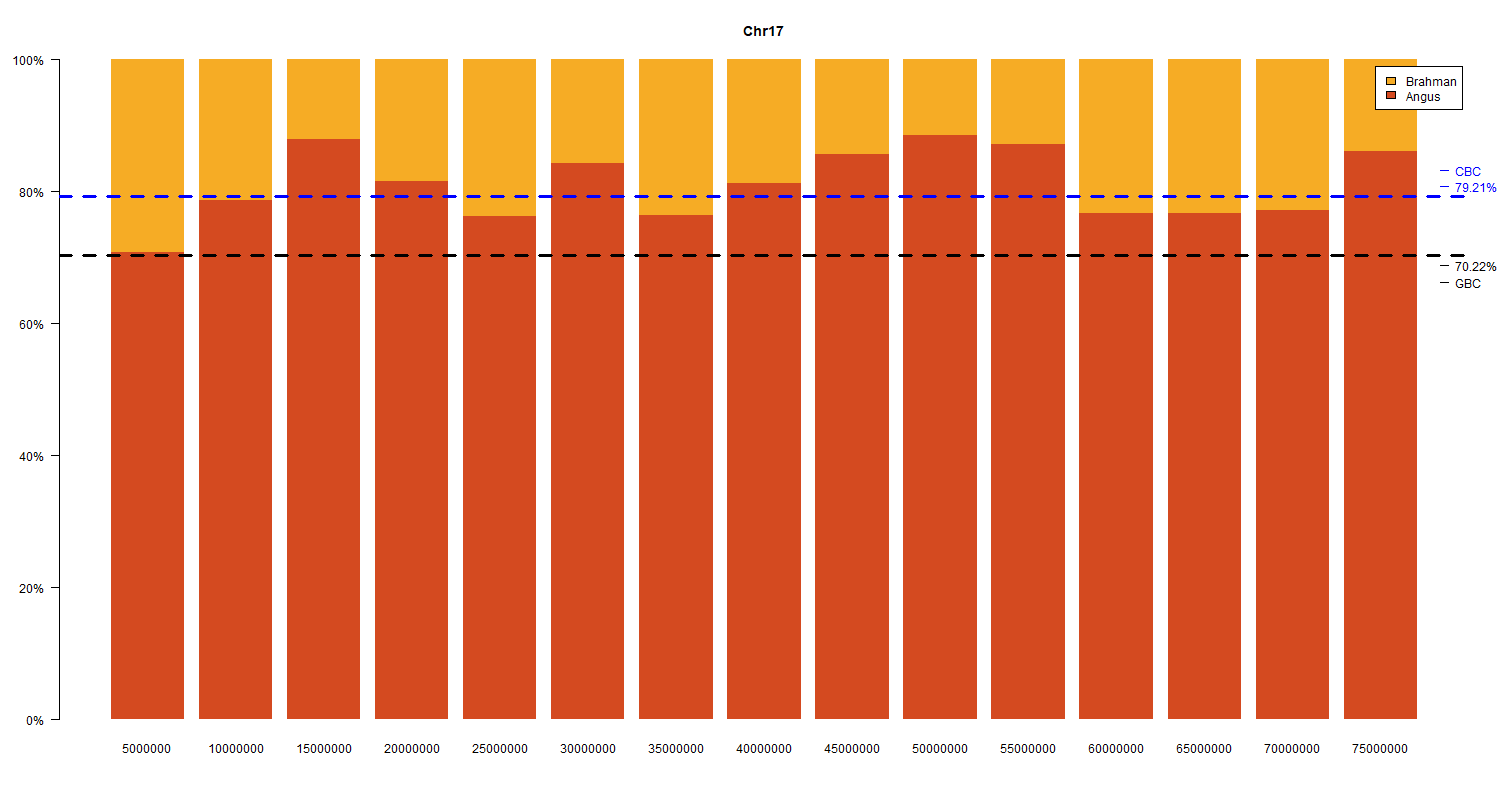

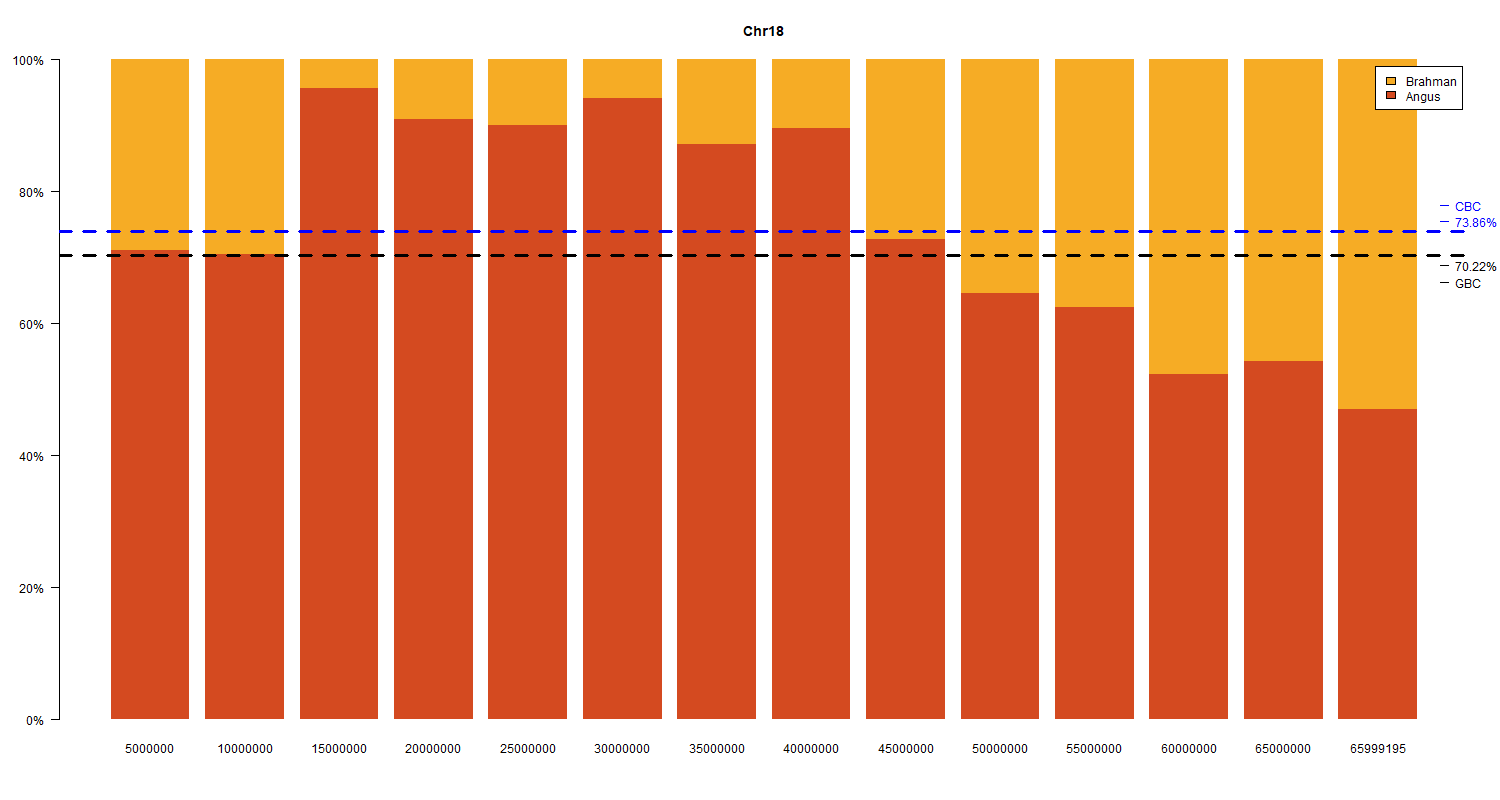

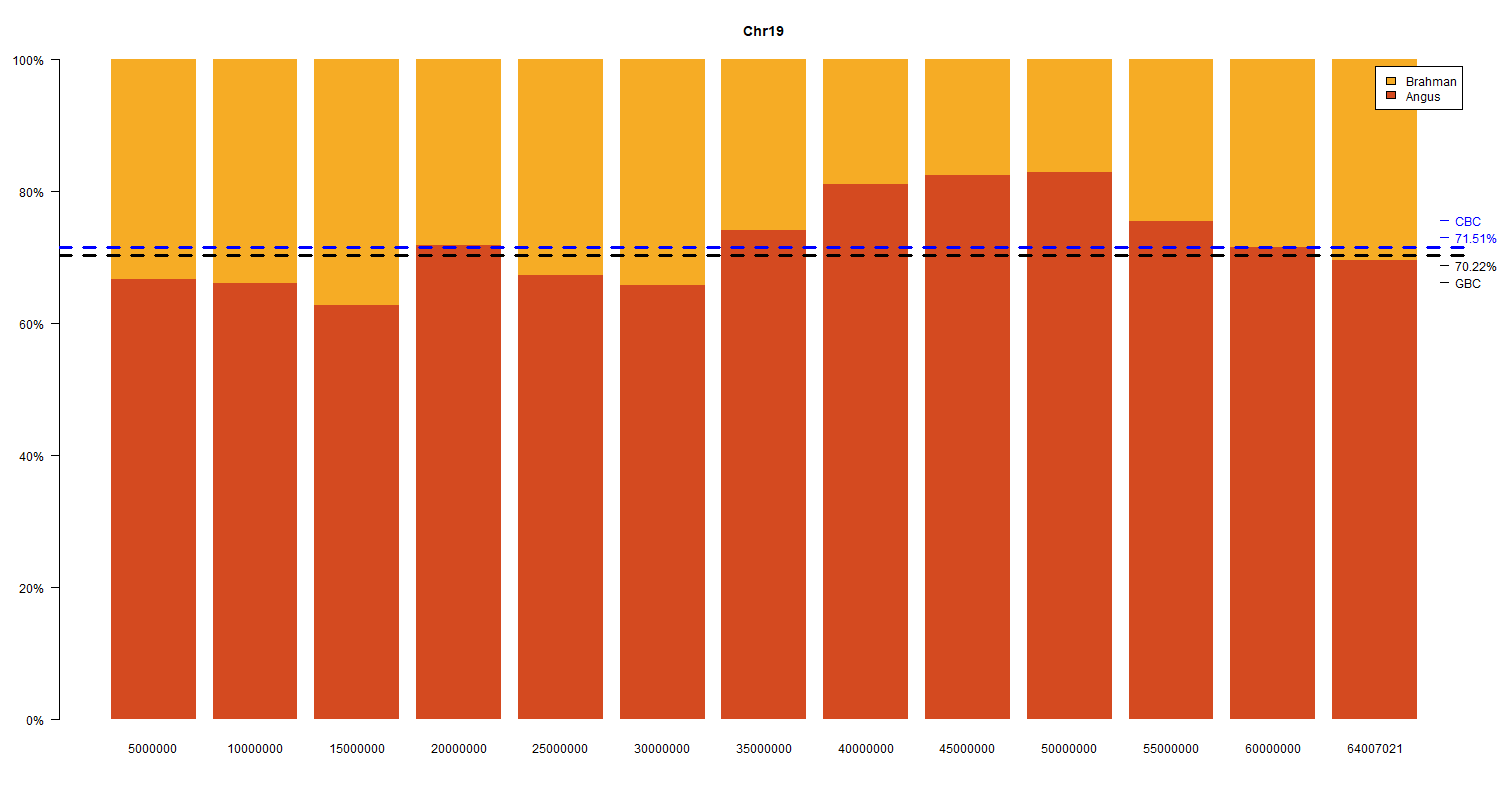

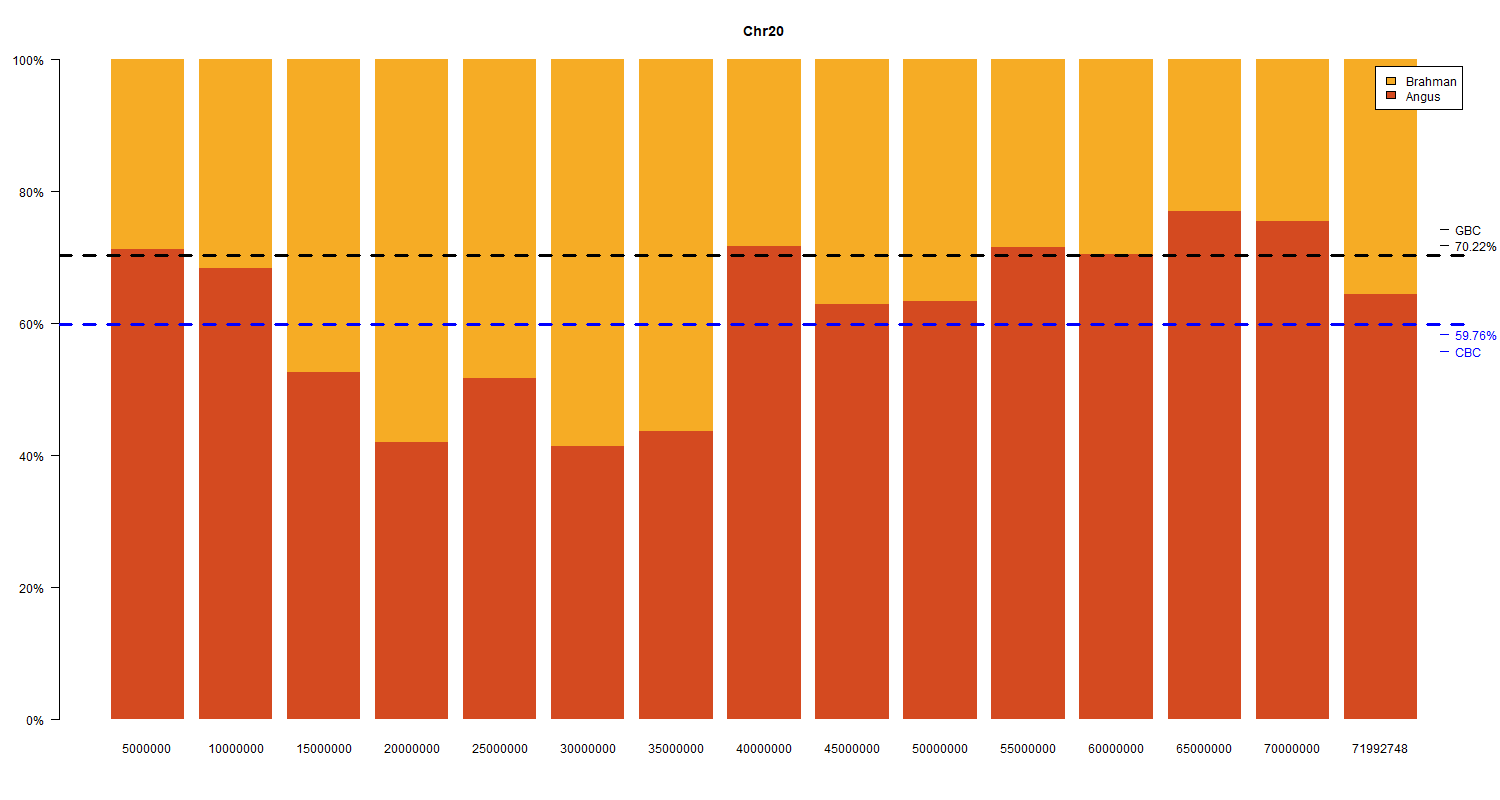

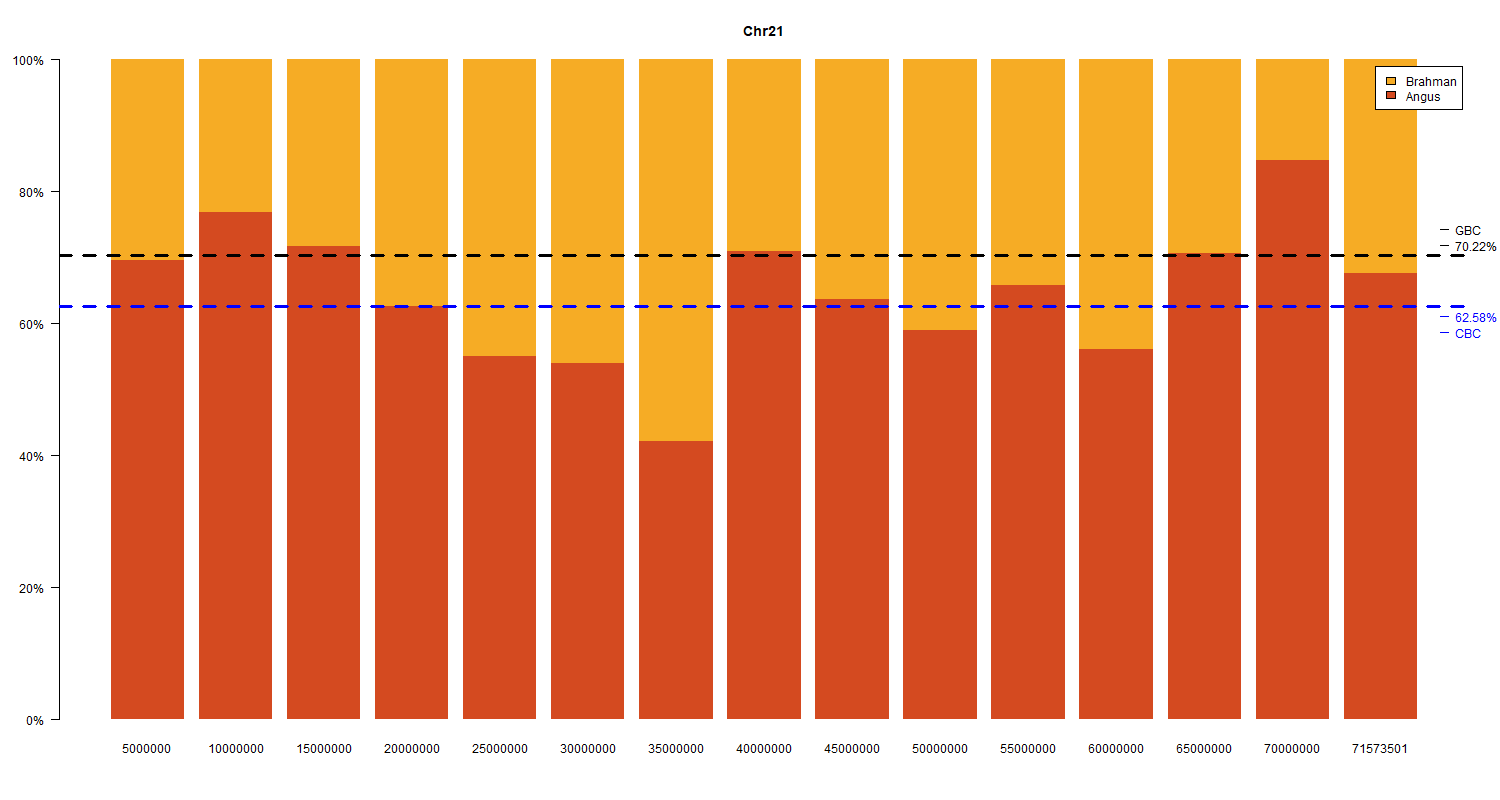

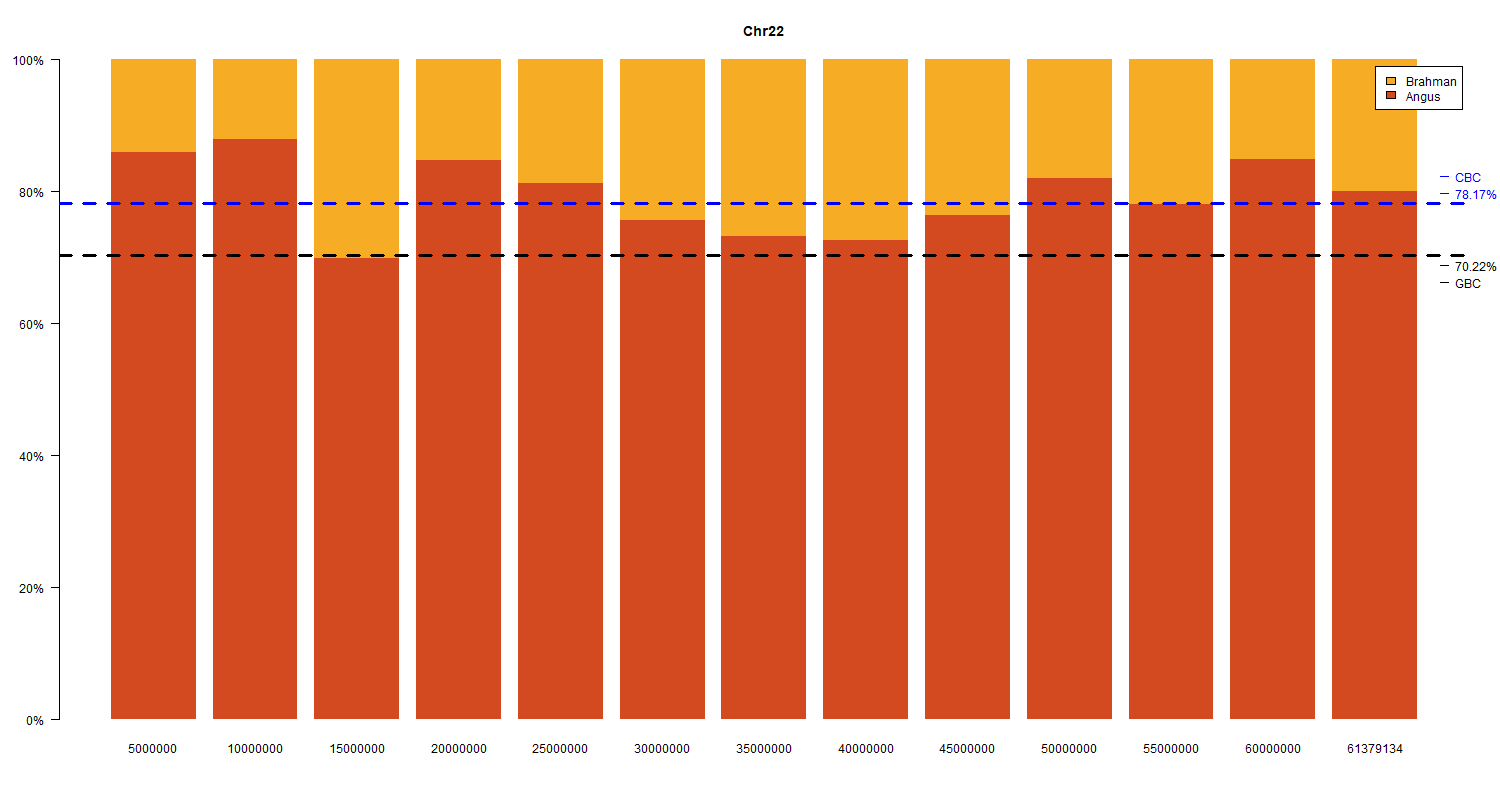

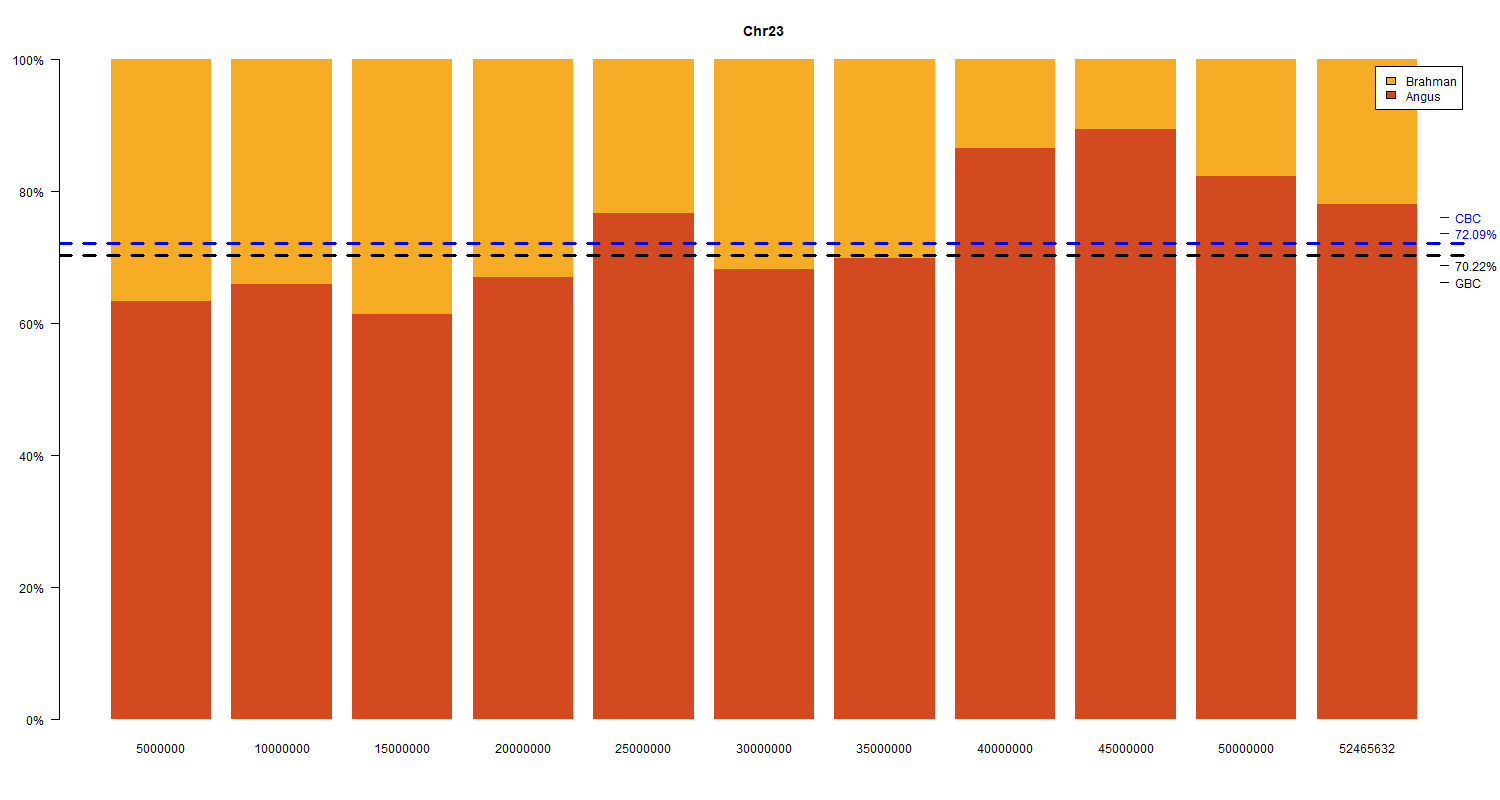

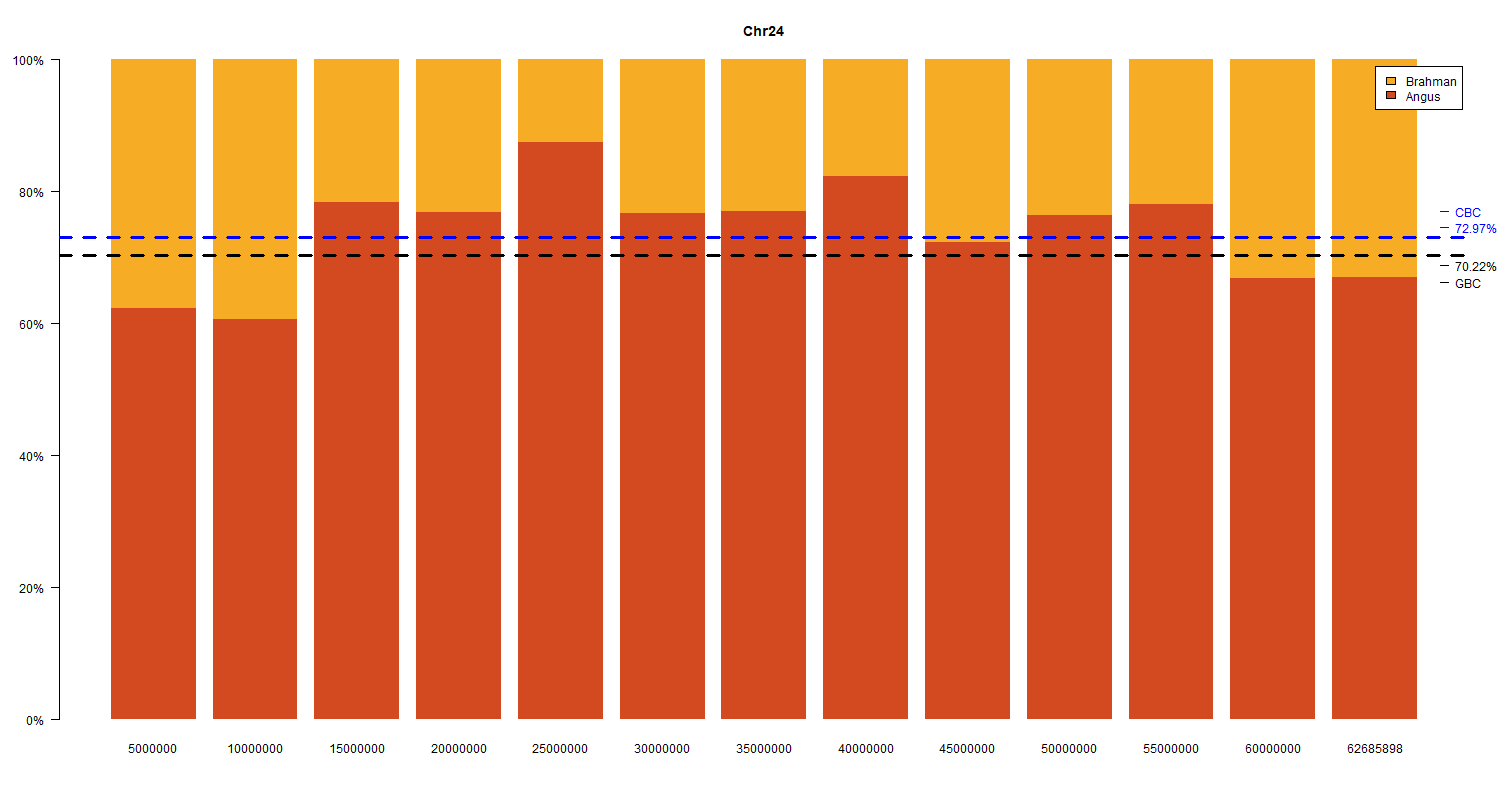

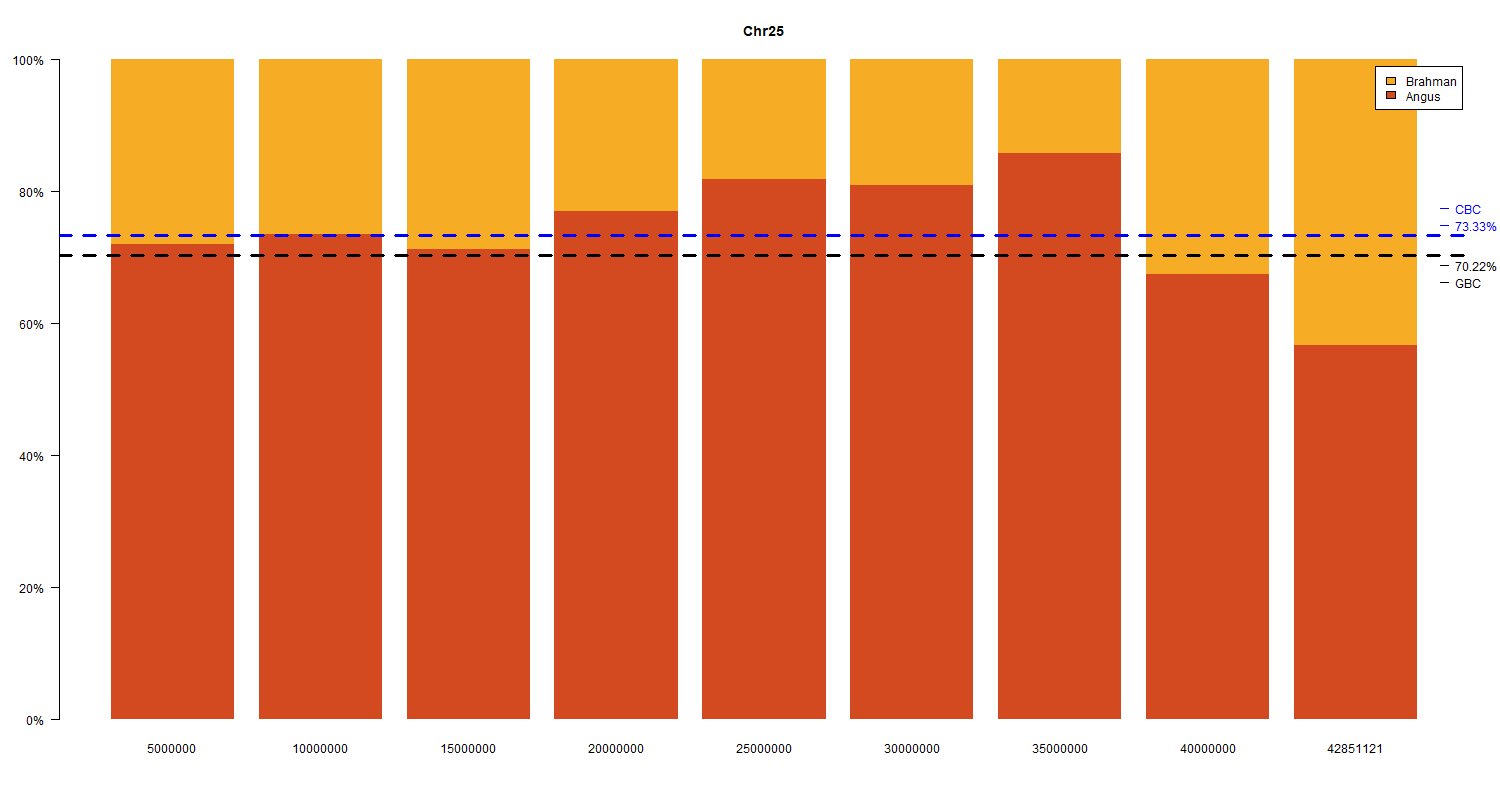

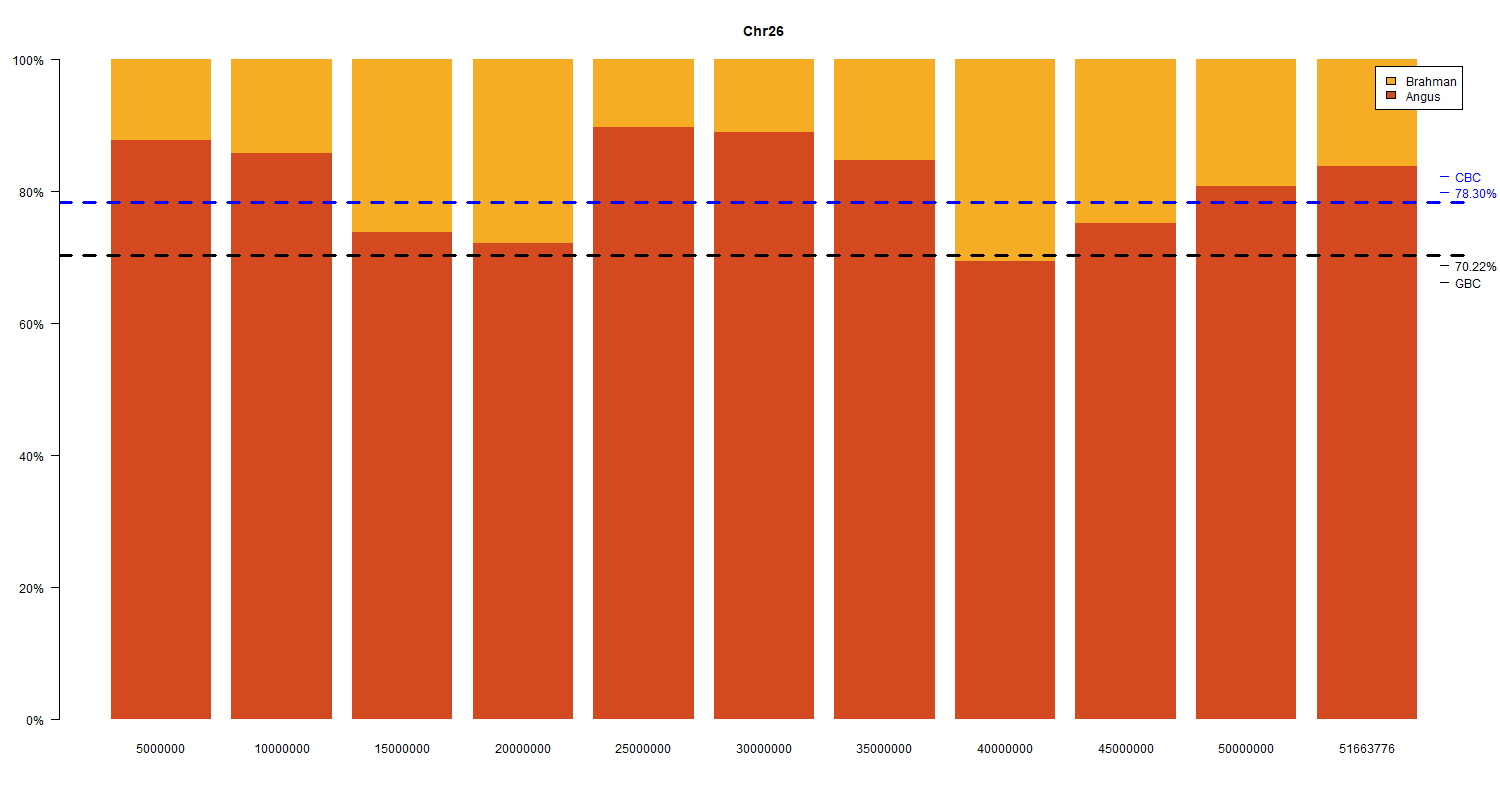

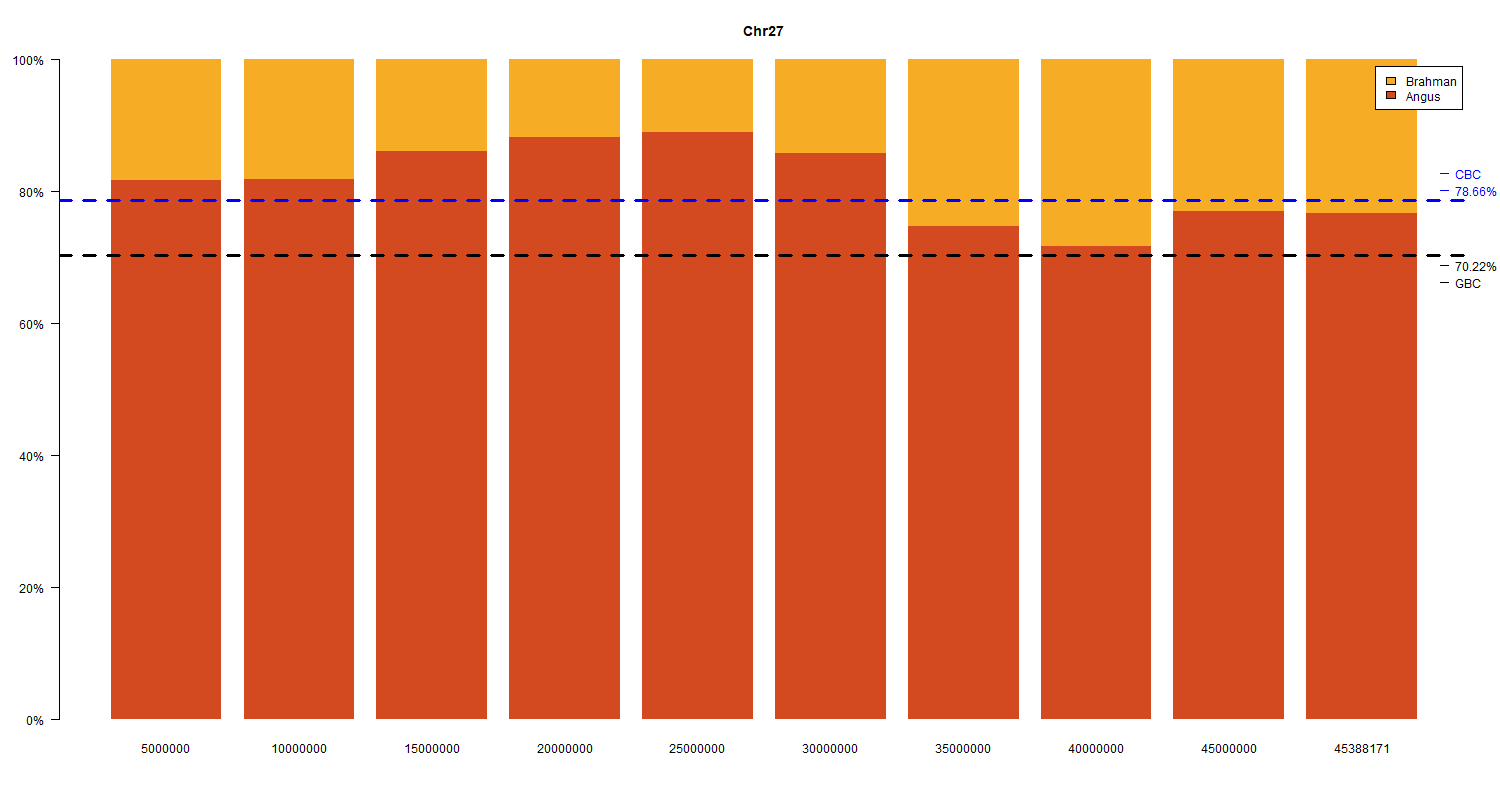

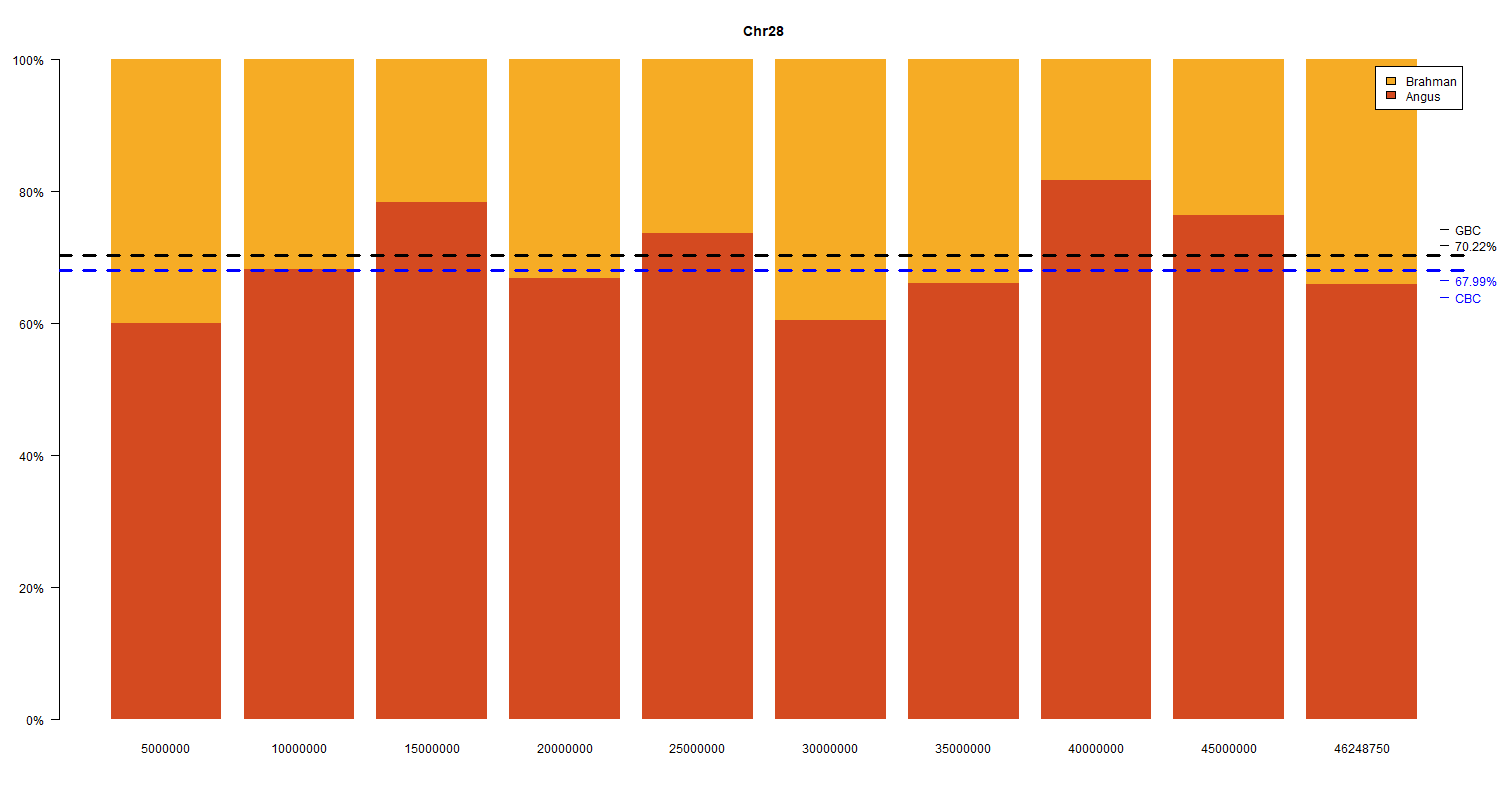

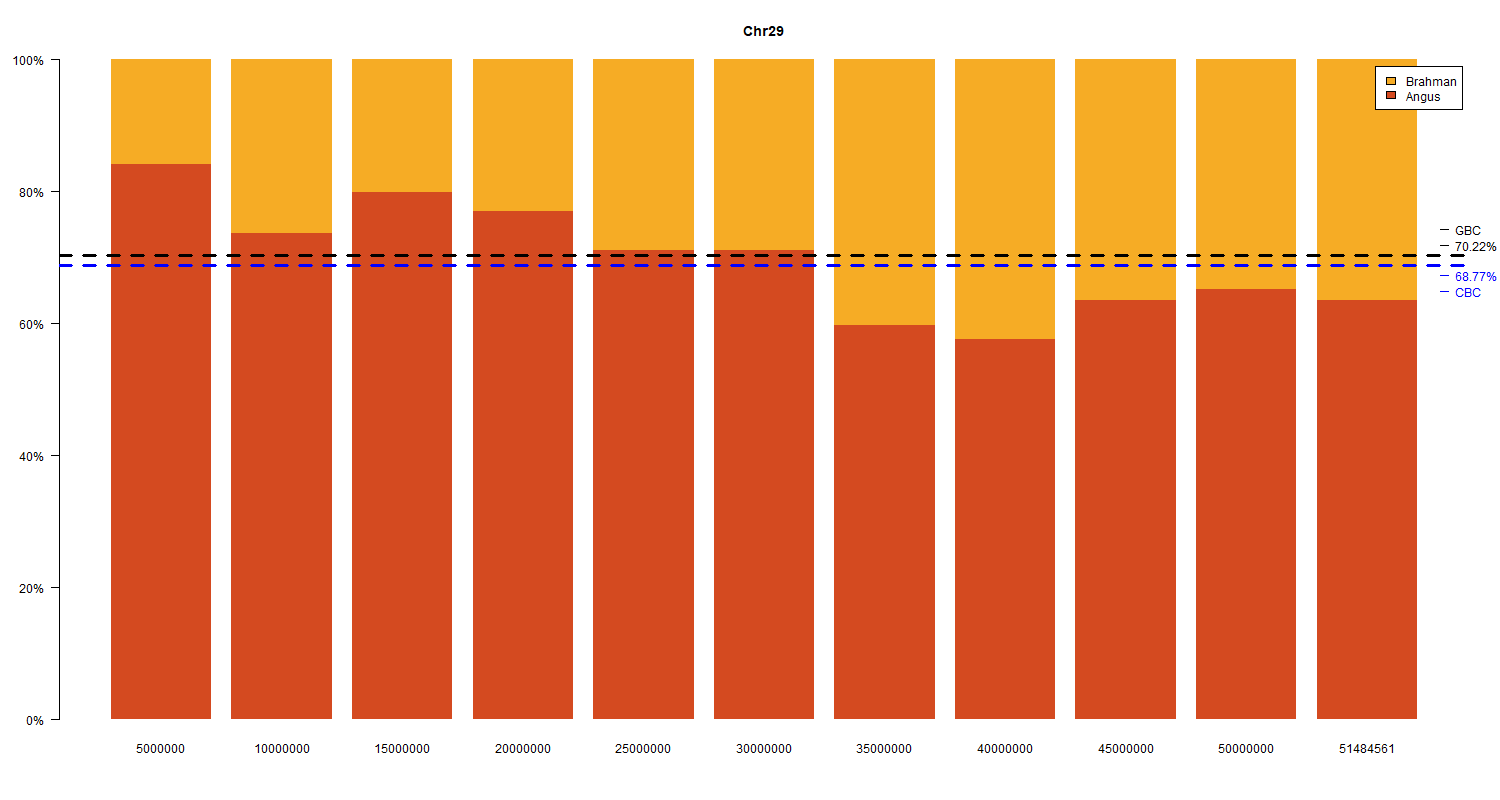


(C)


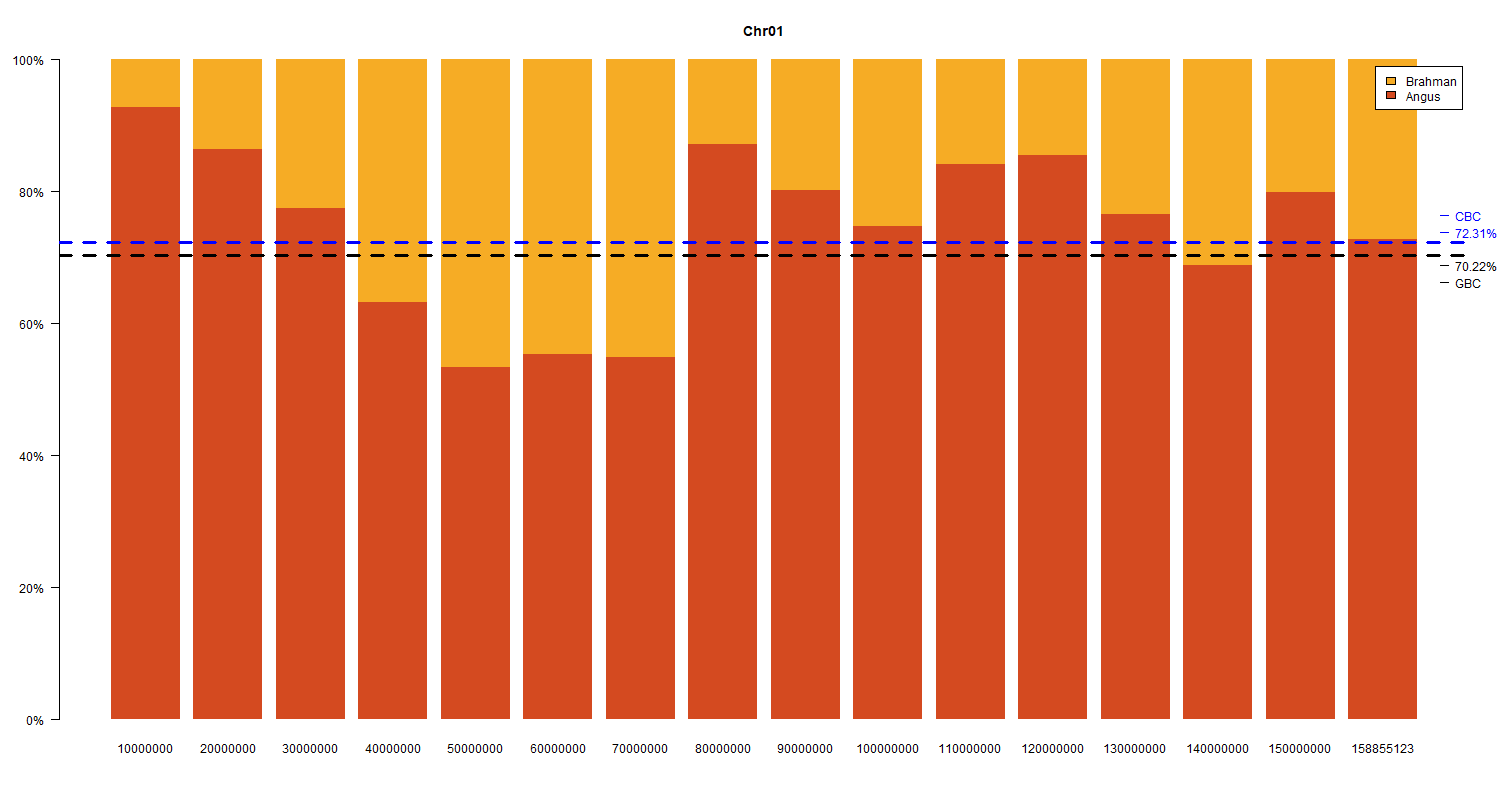

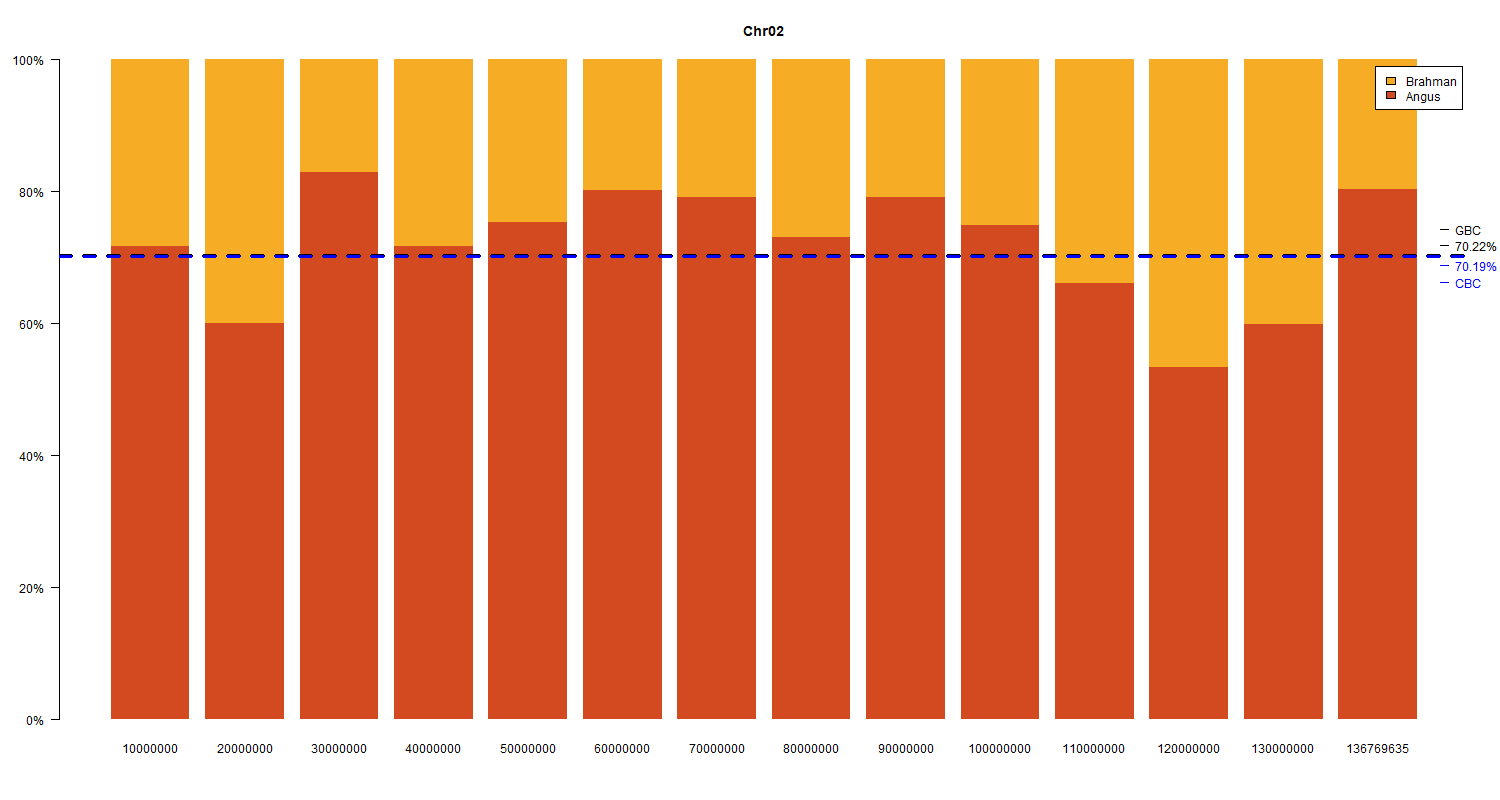

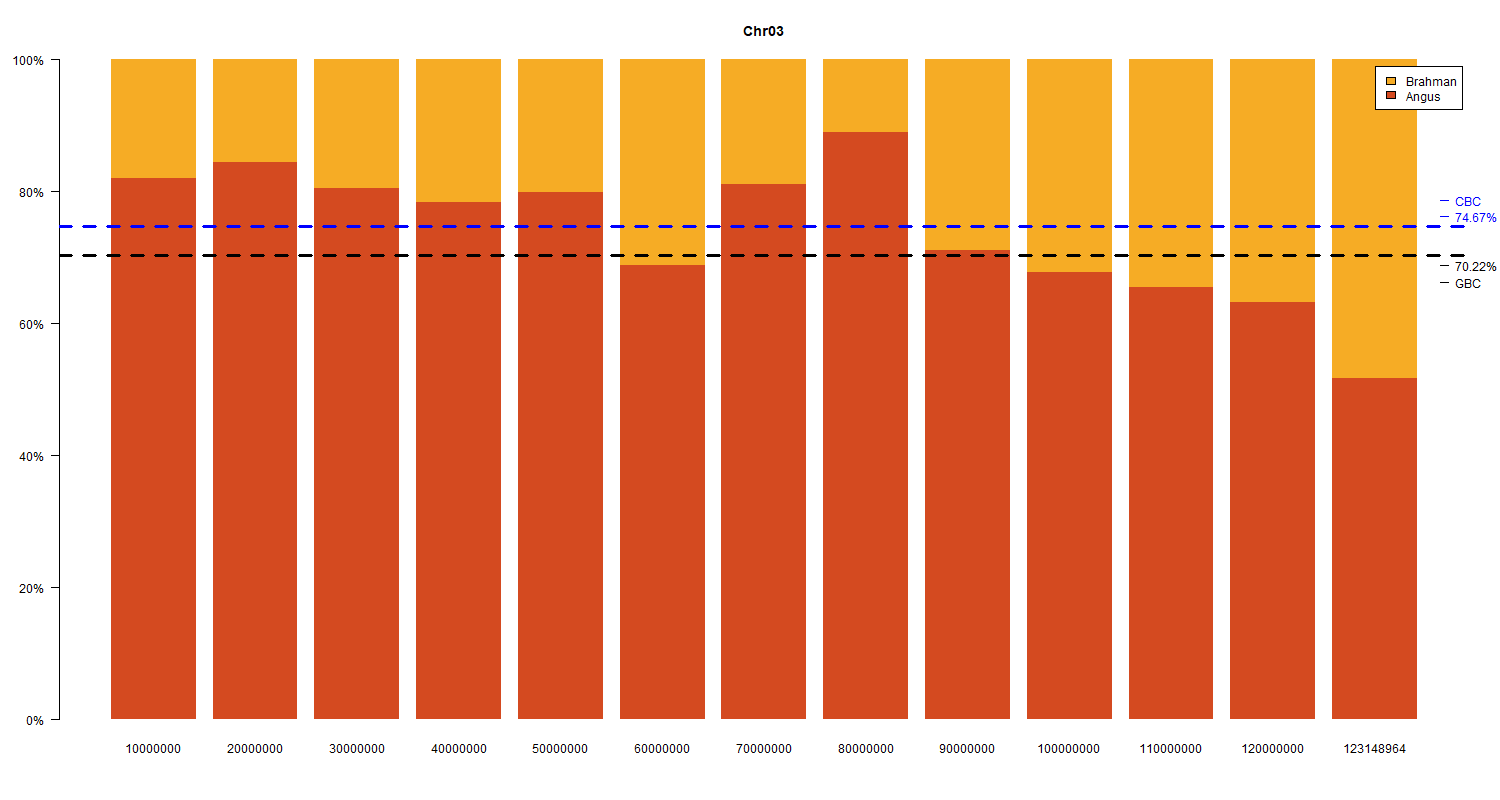

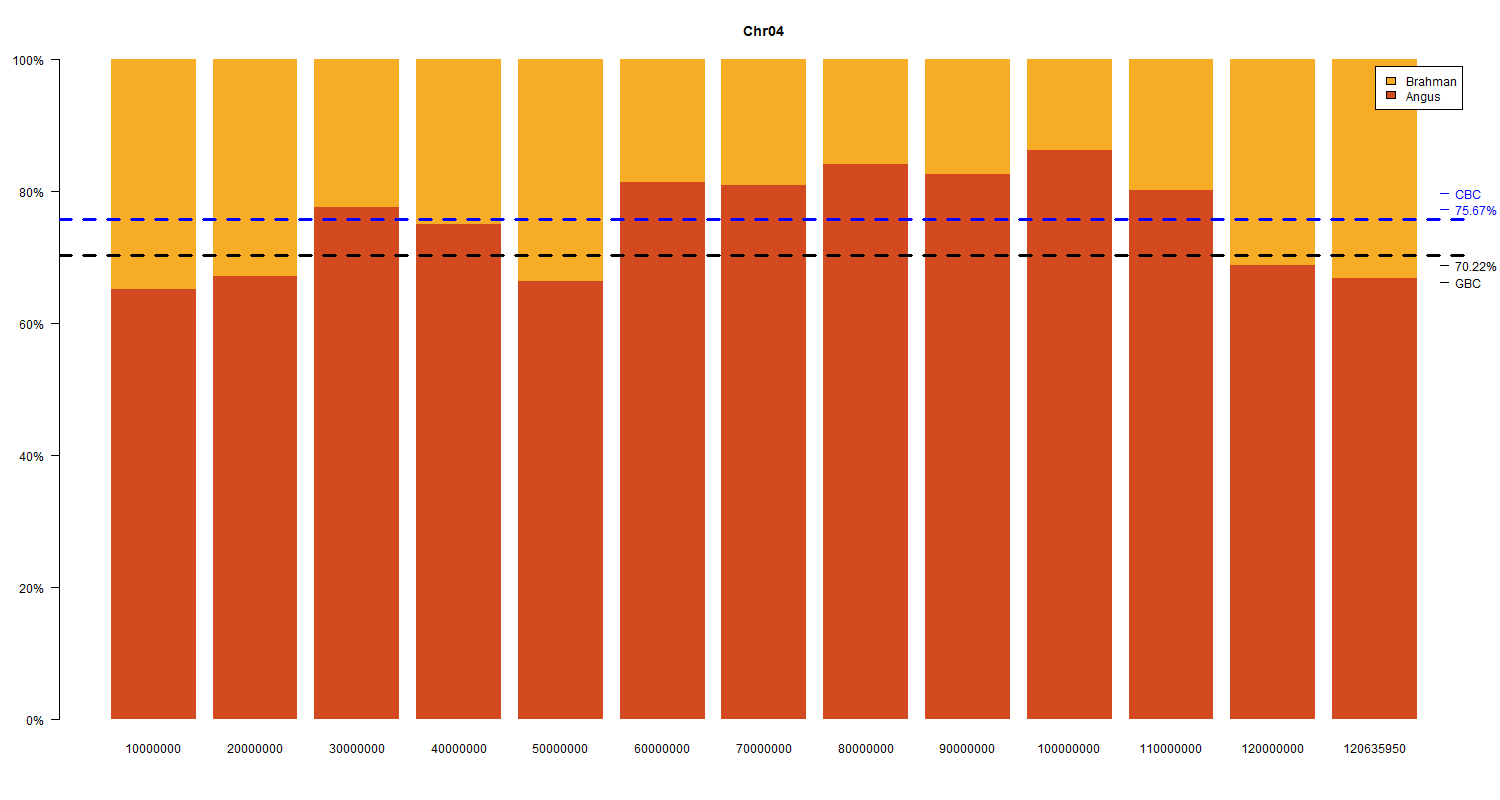

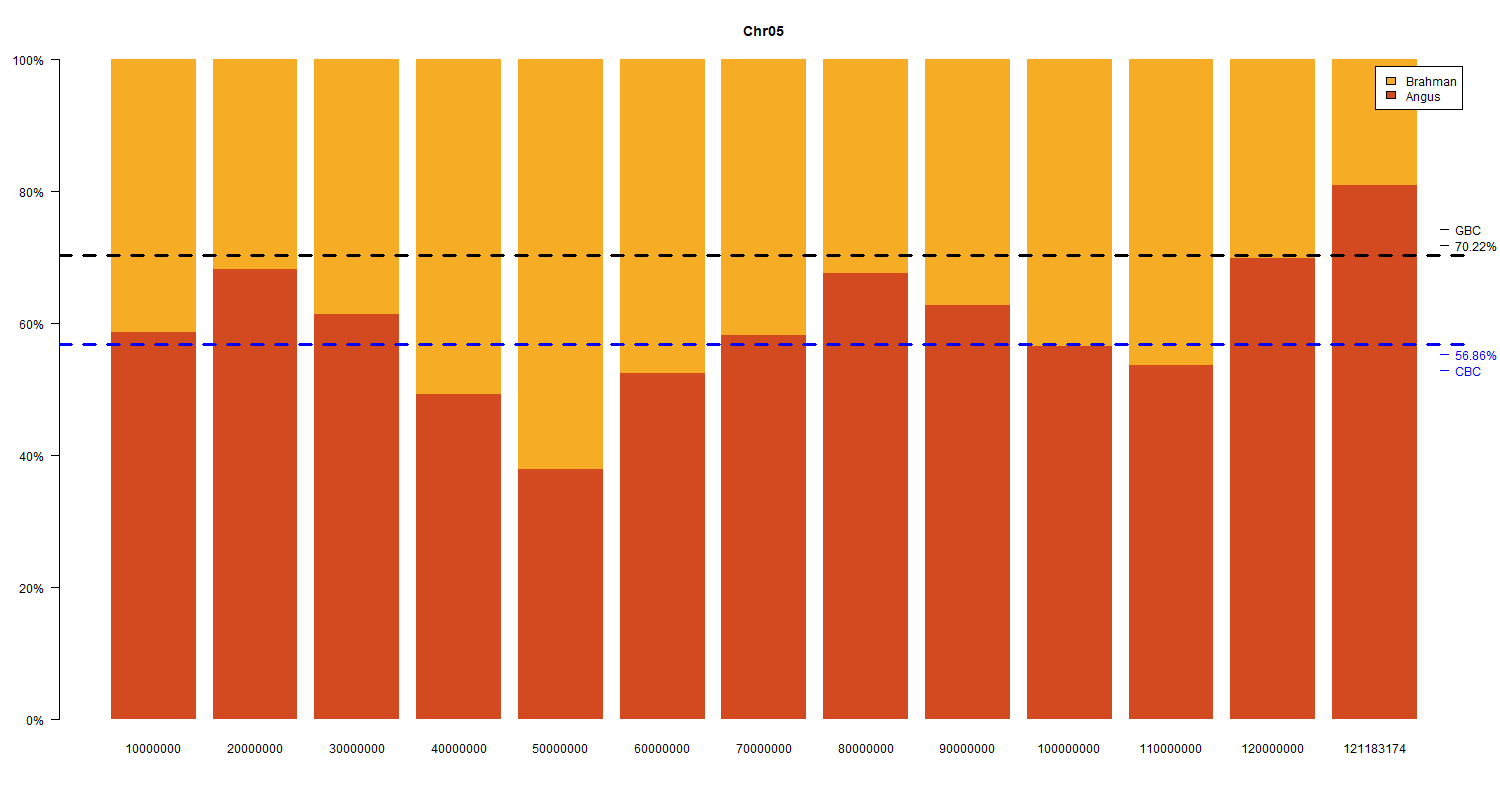

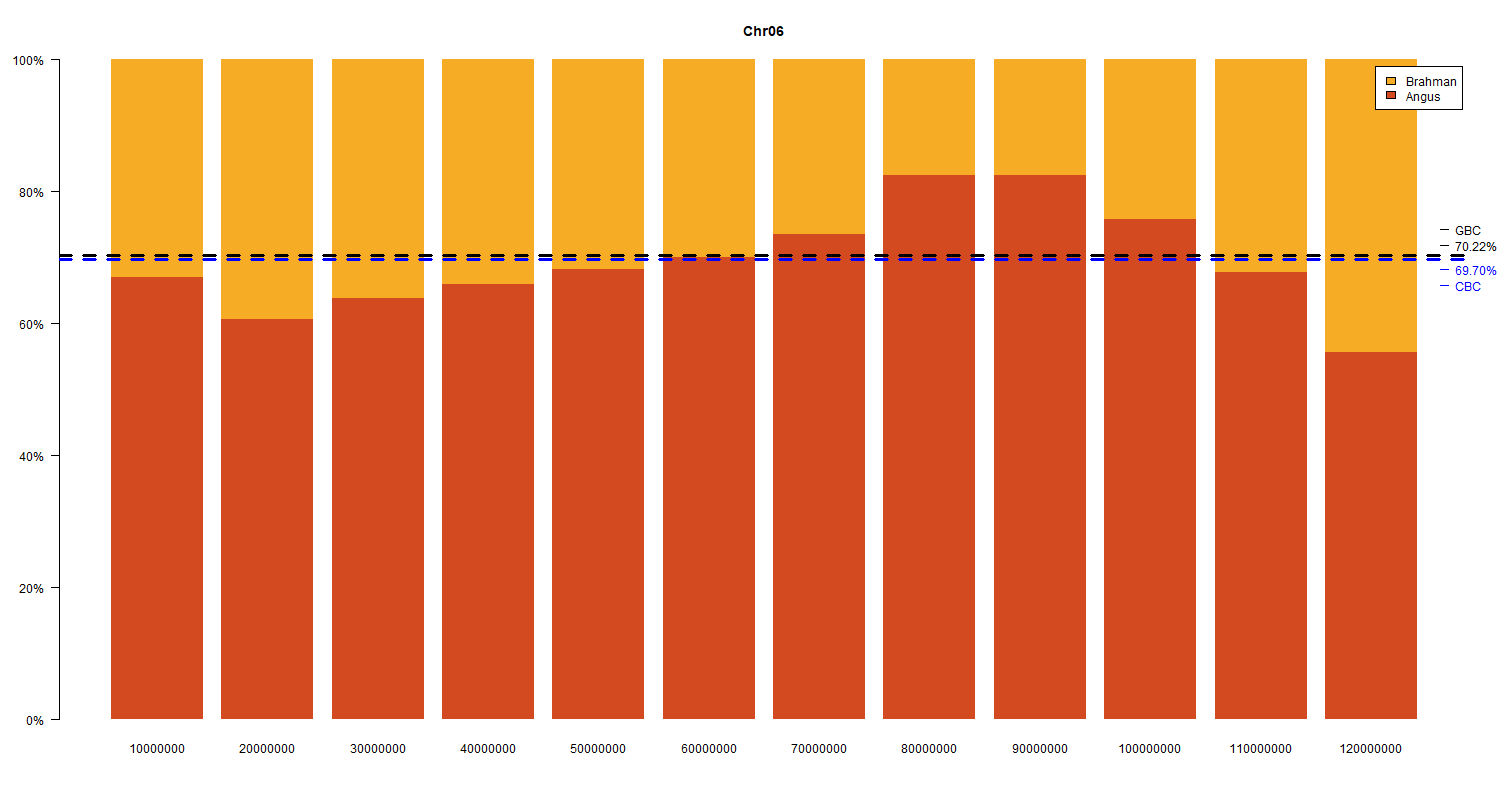

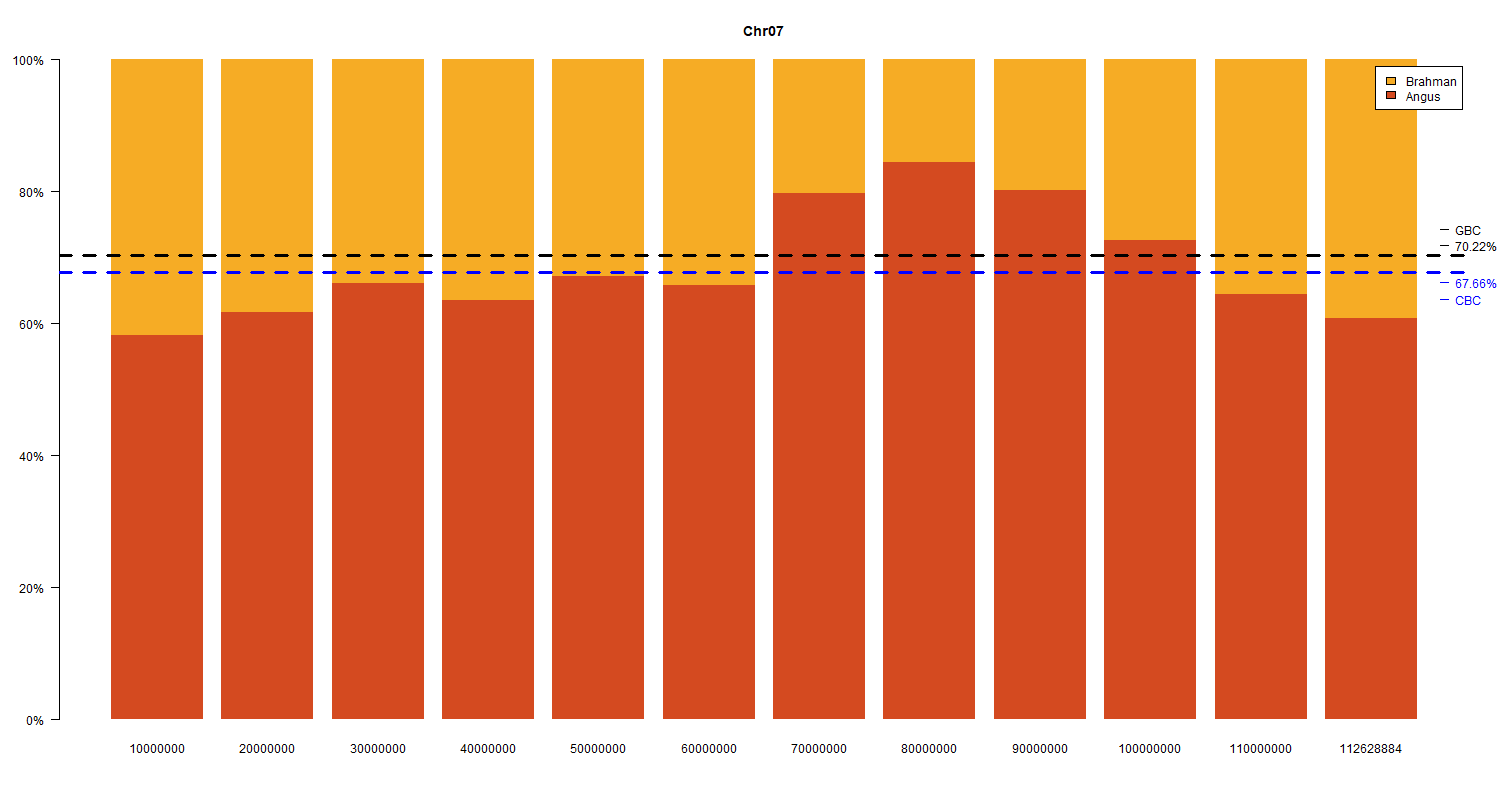

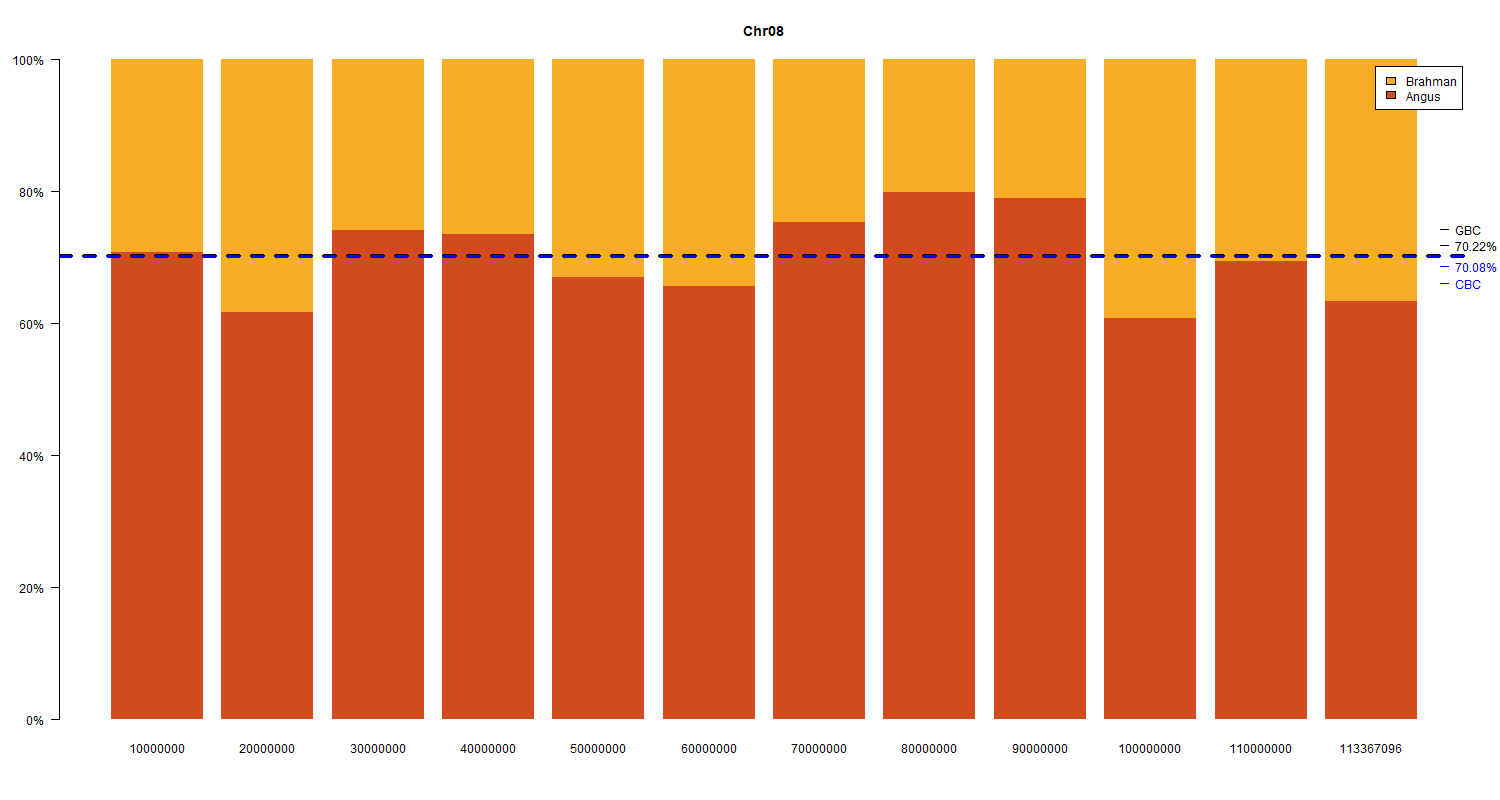

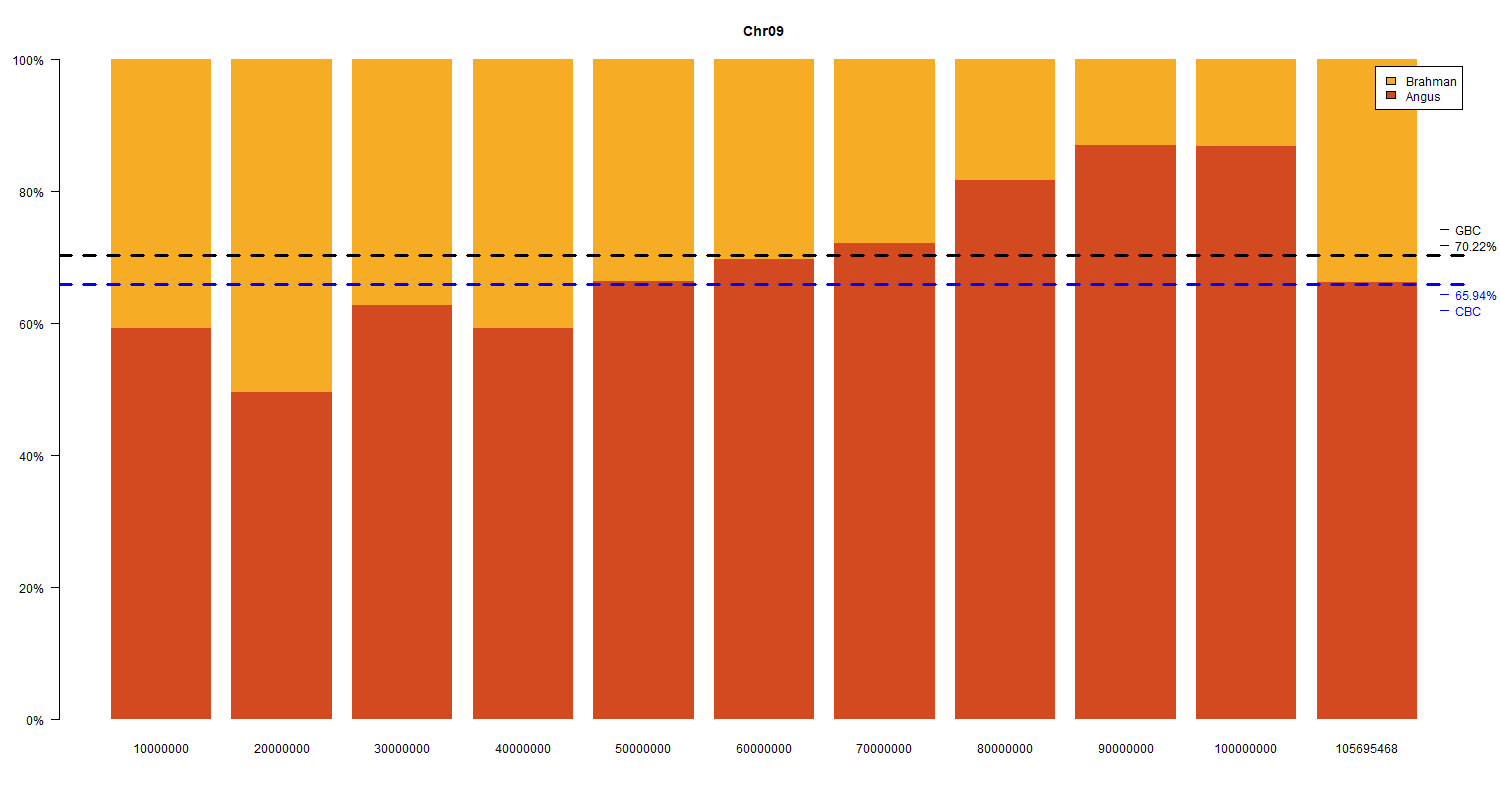

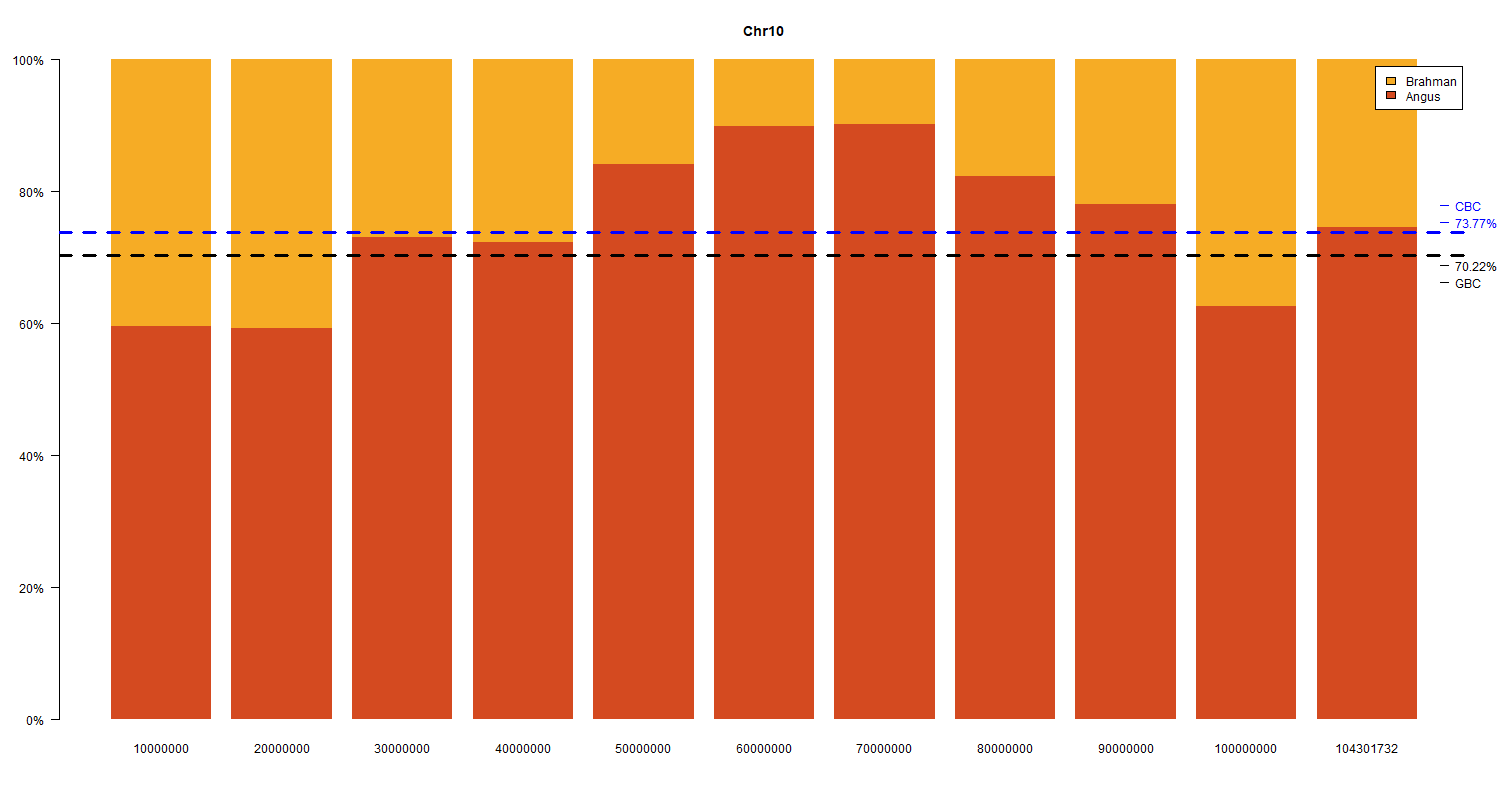

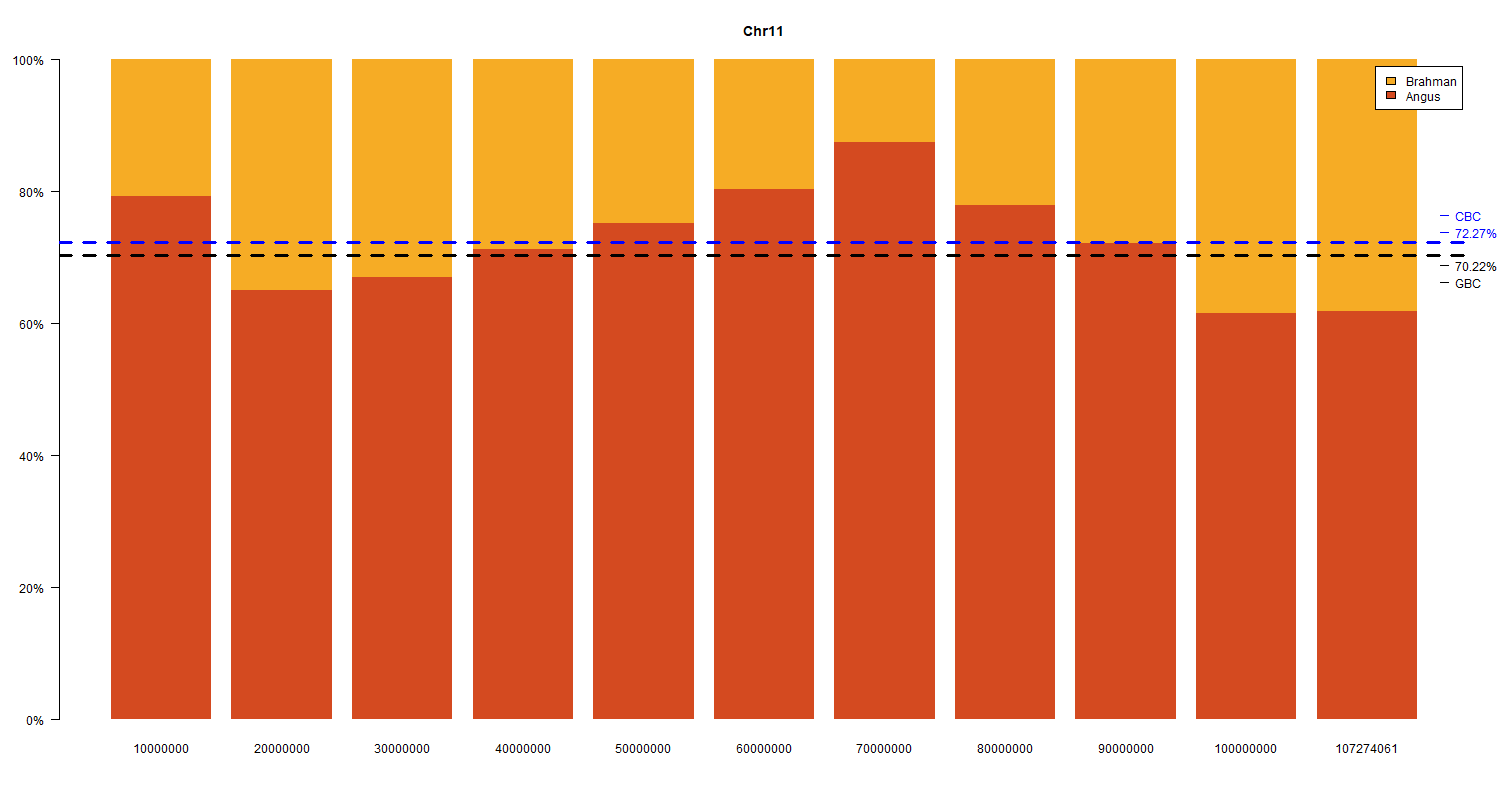

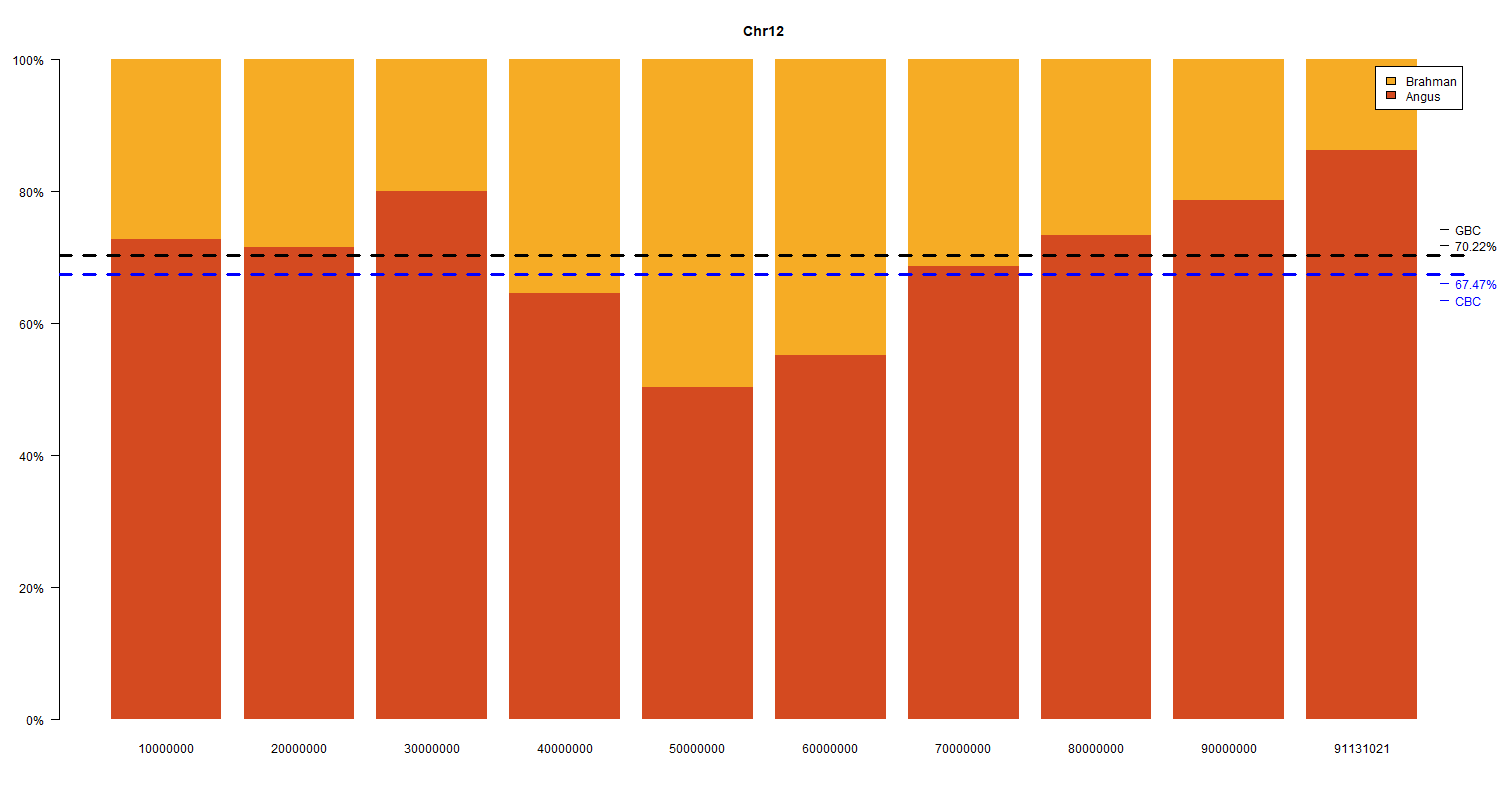

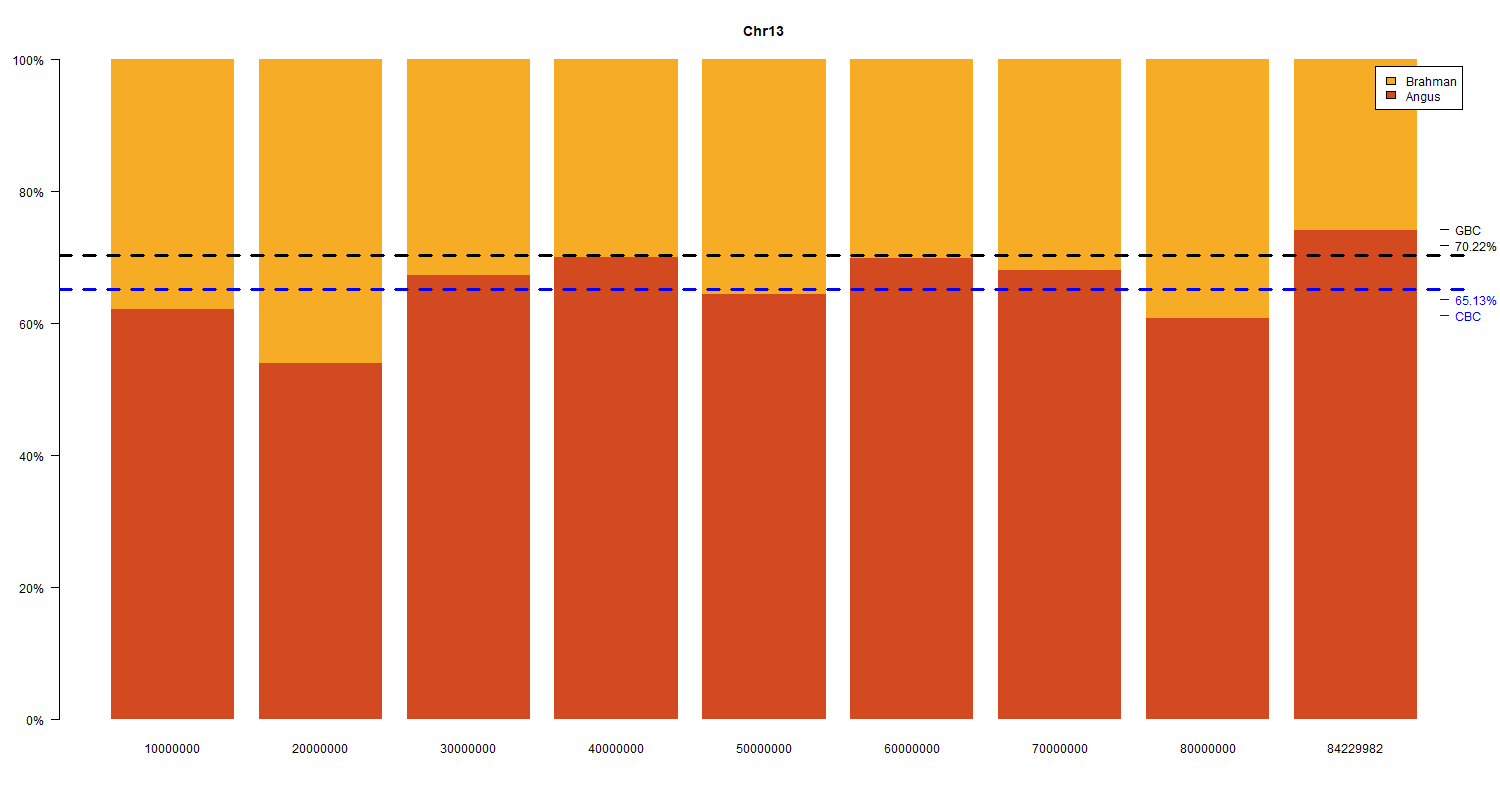

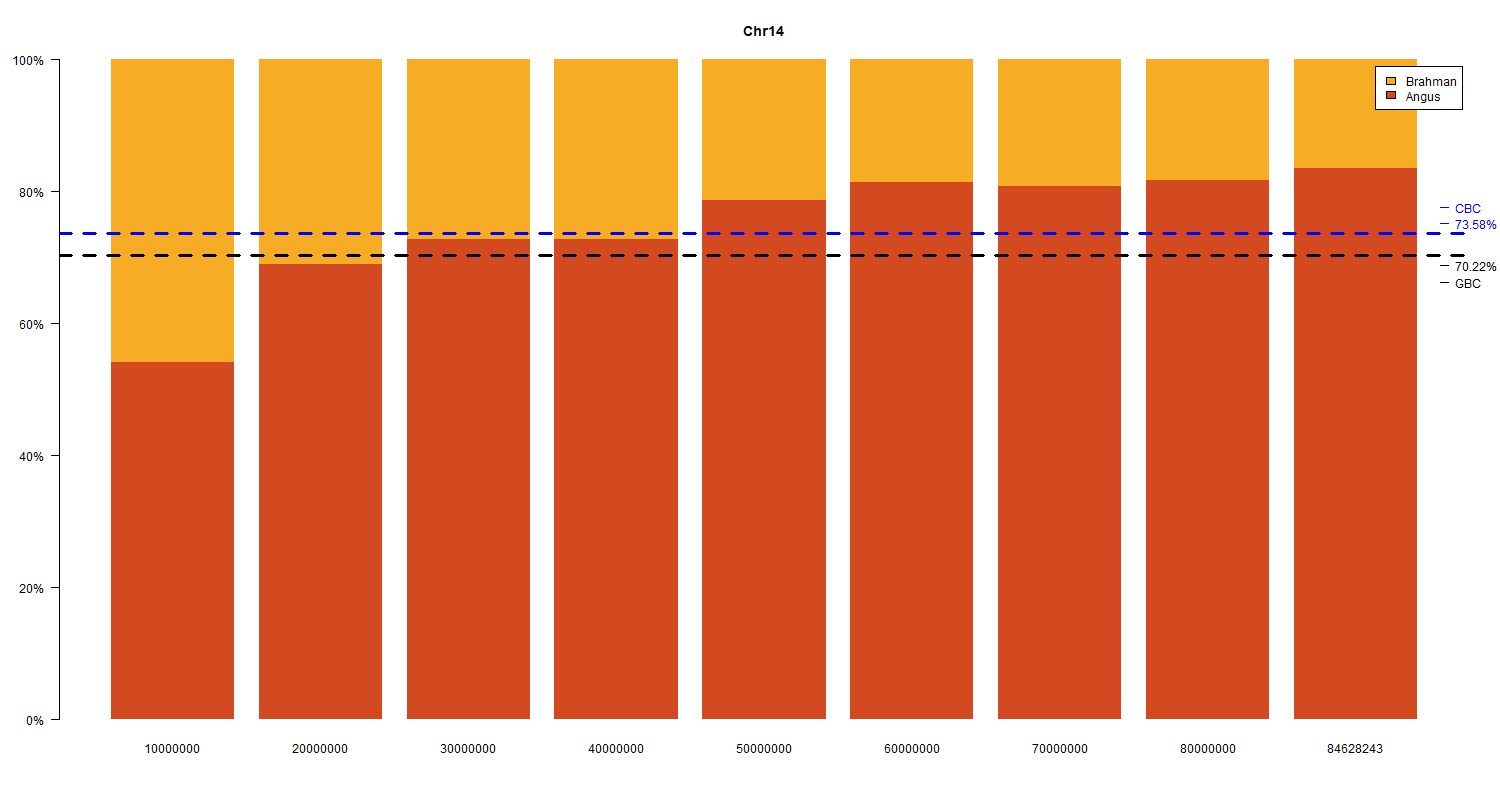

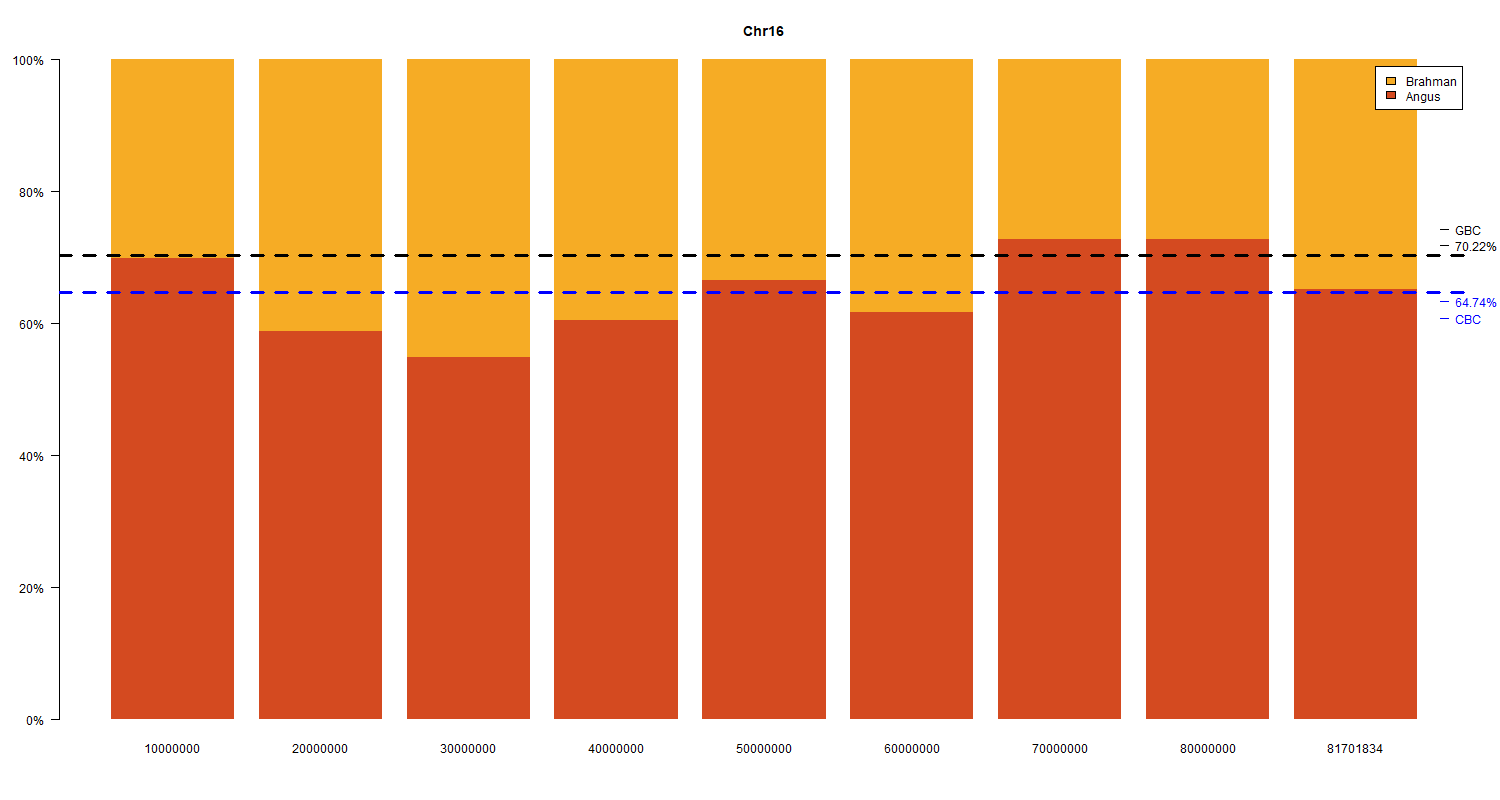

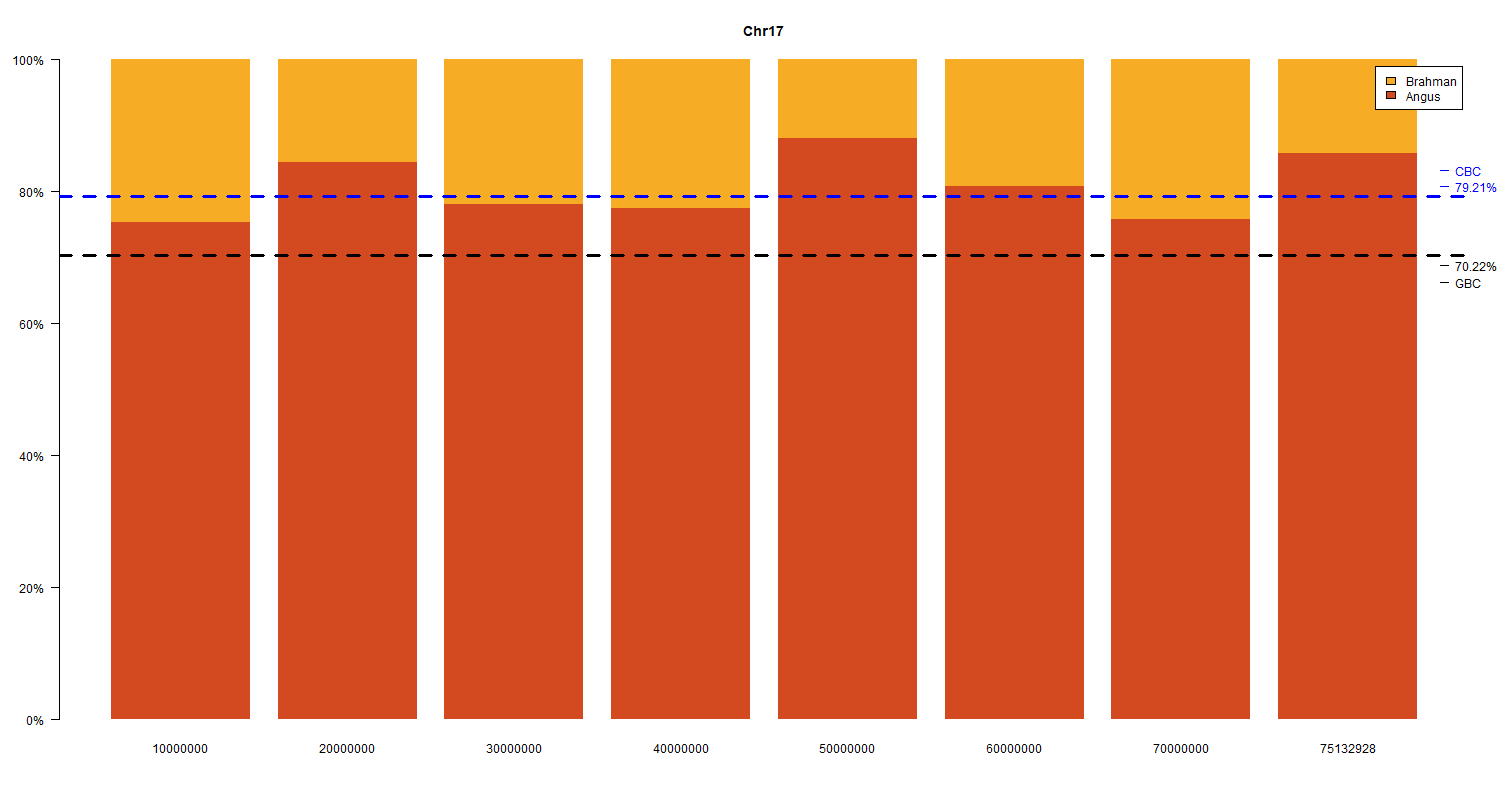

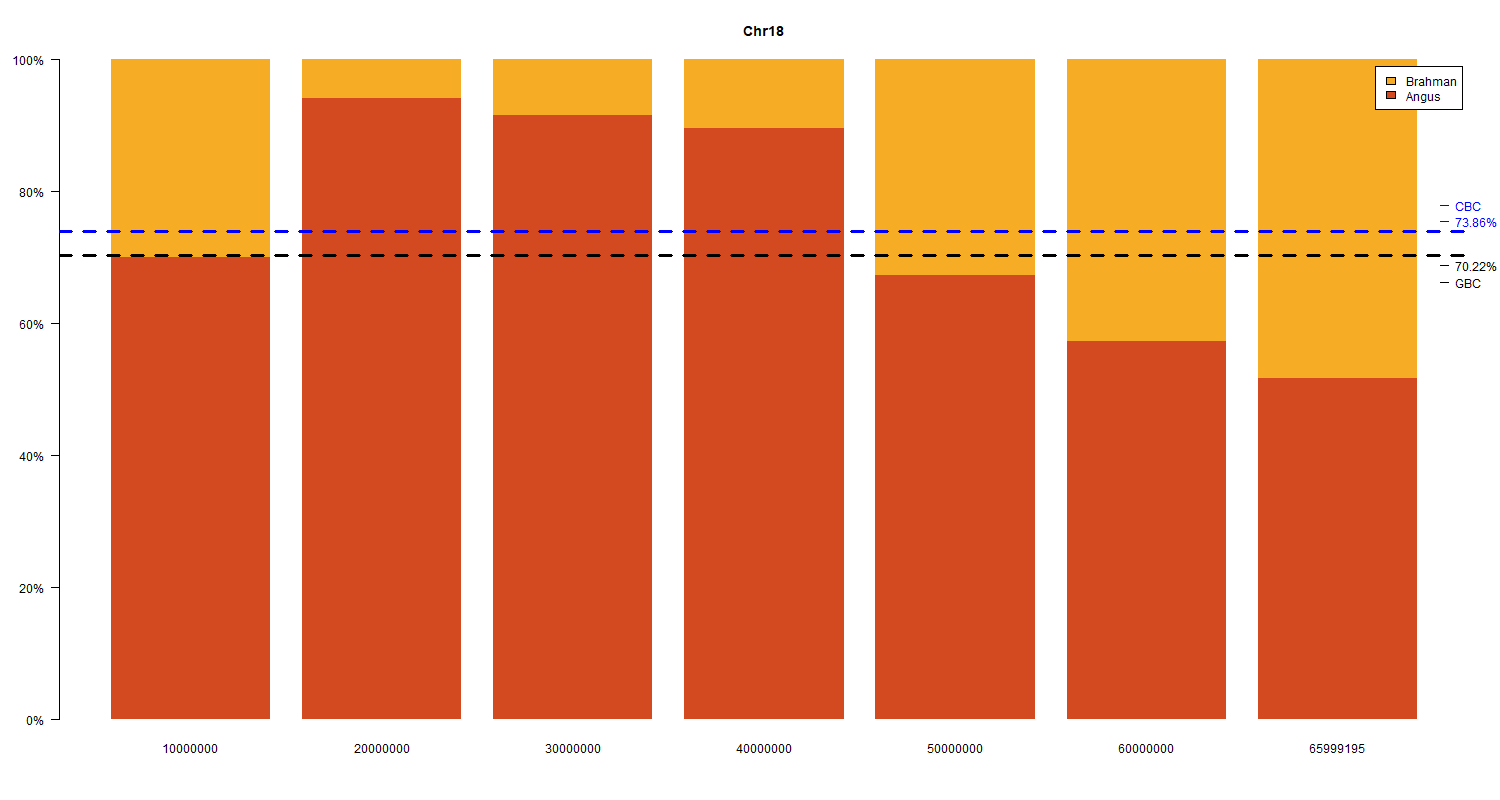

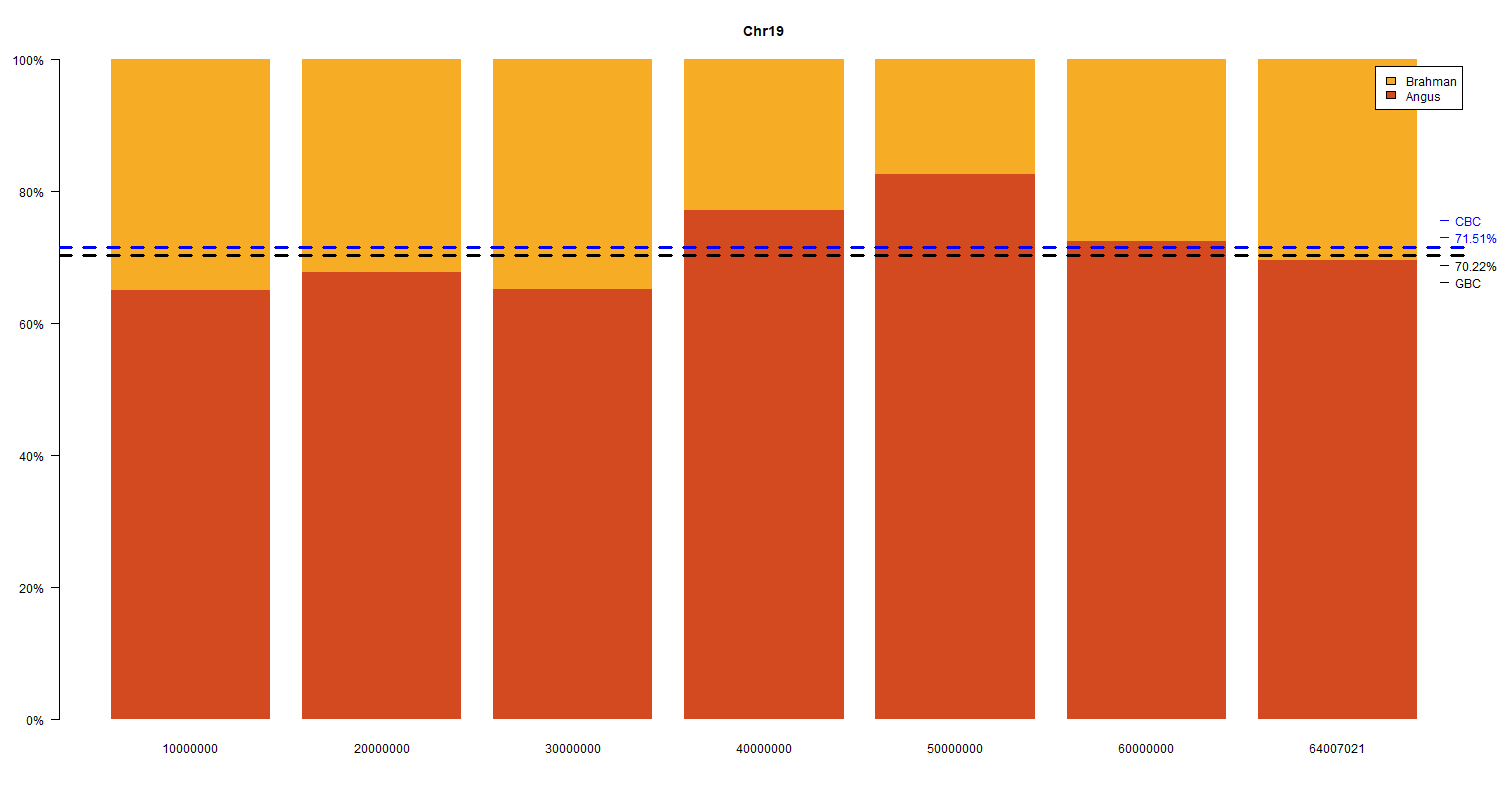

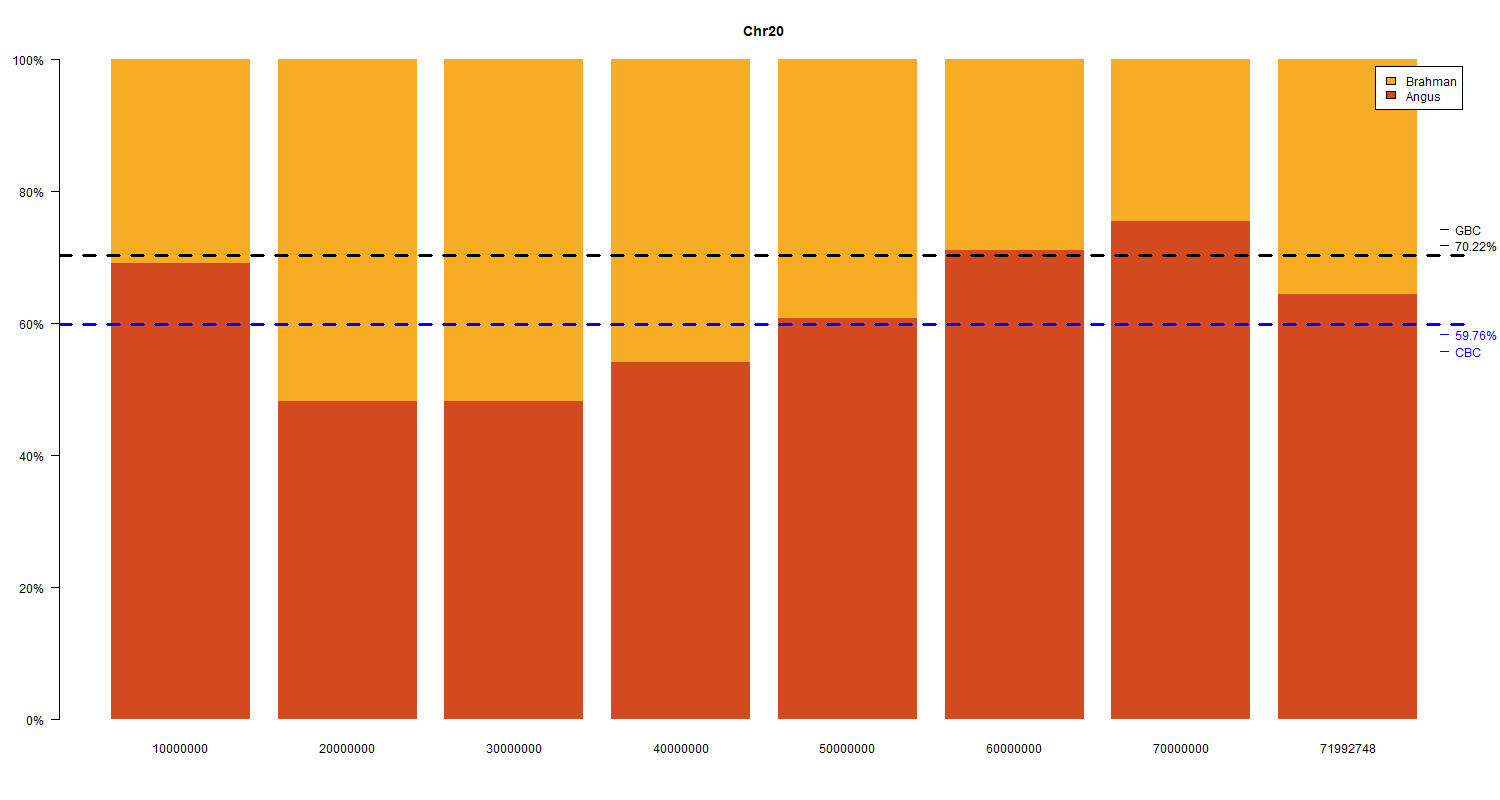

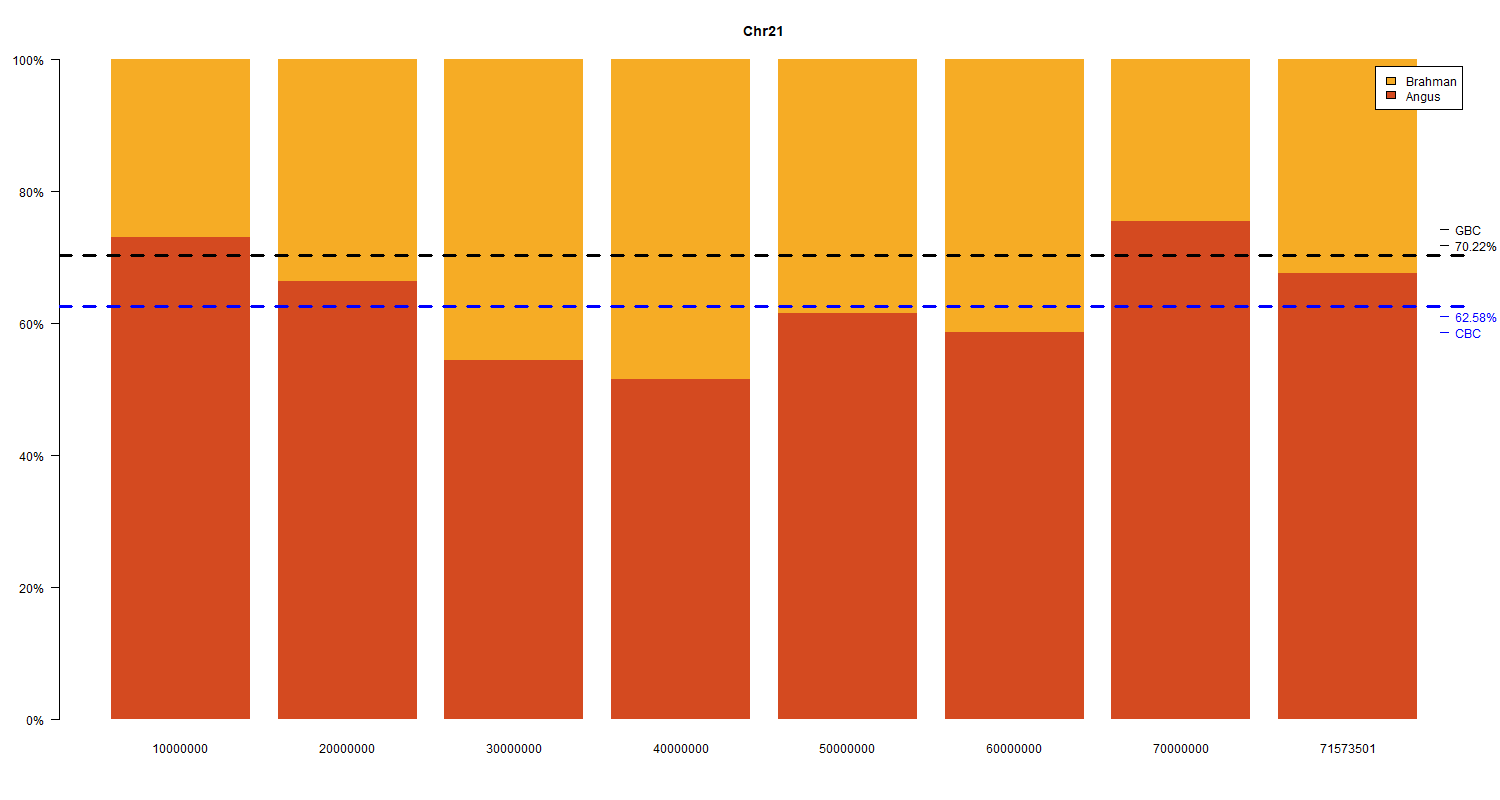

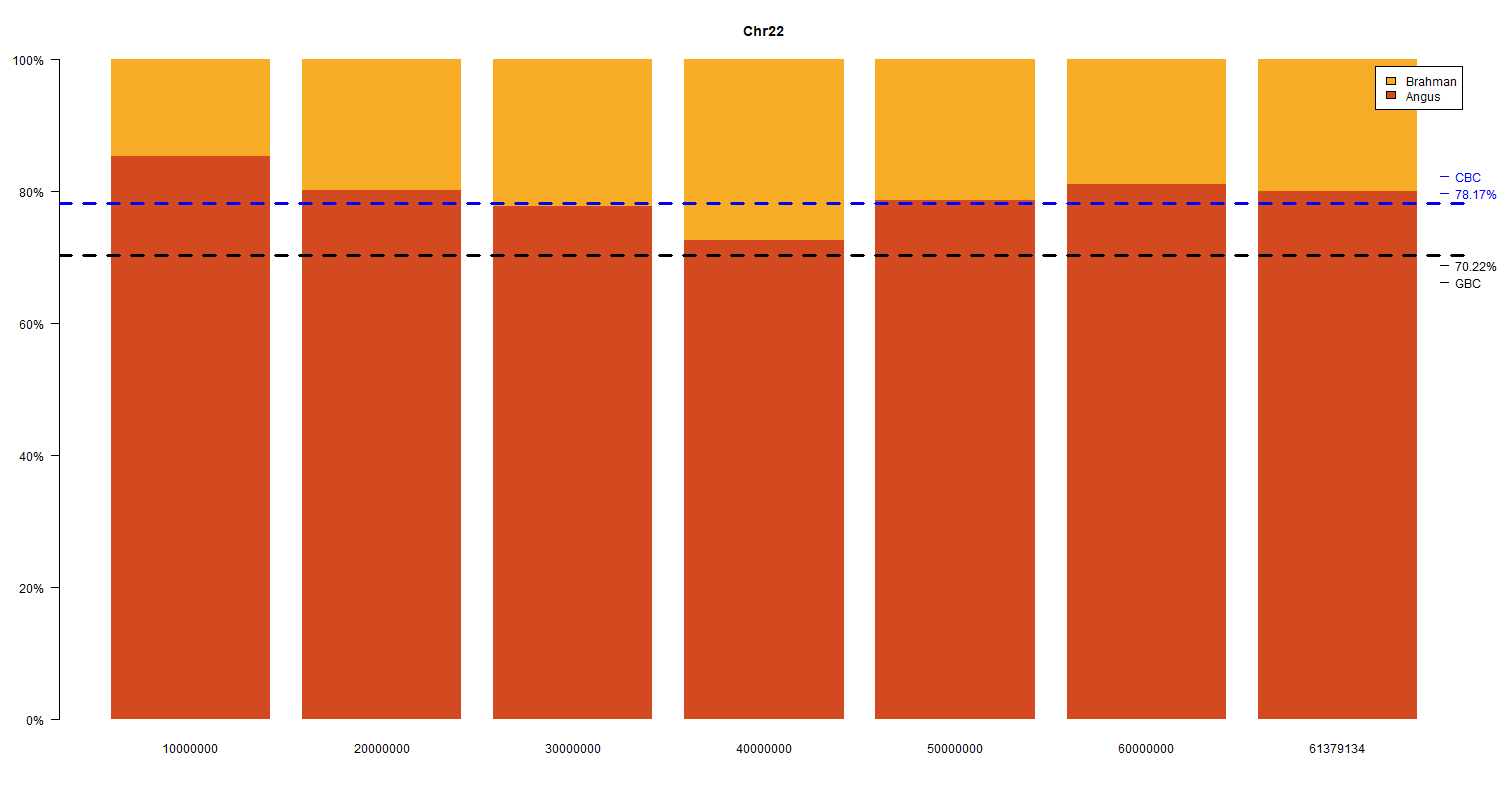

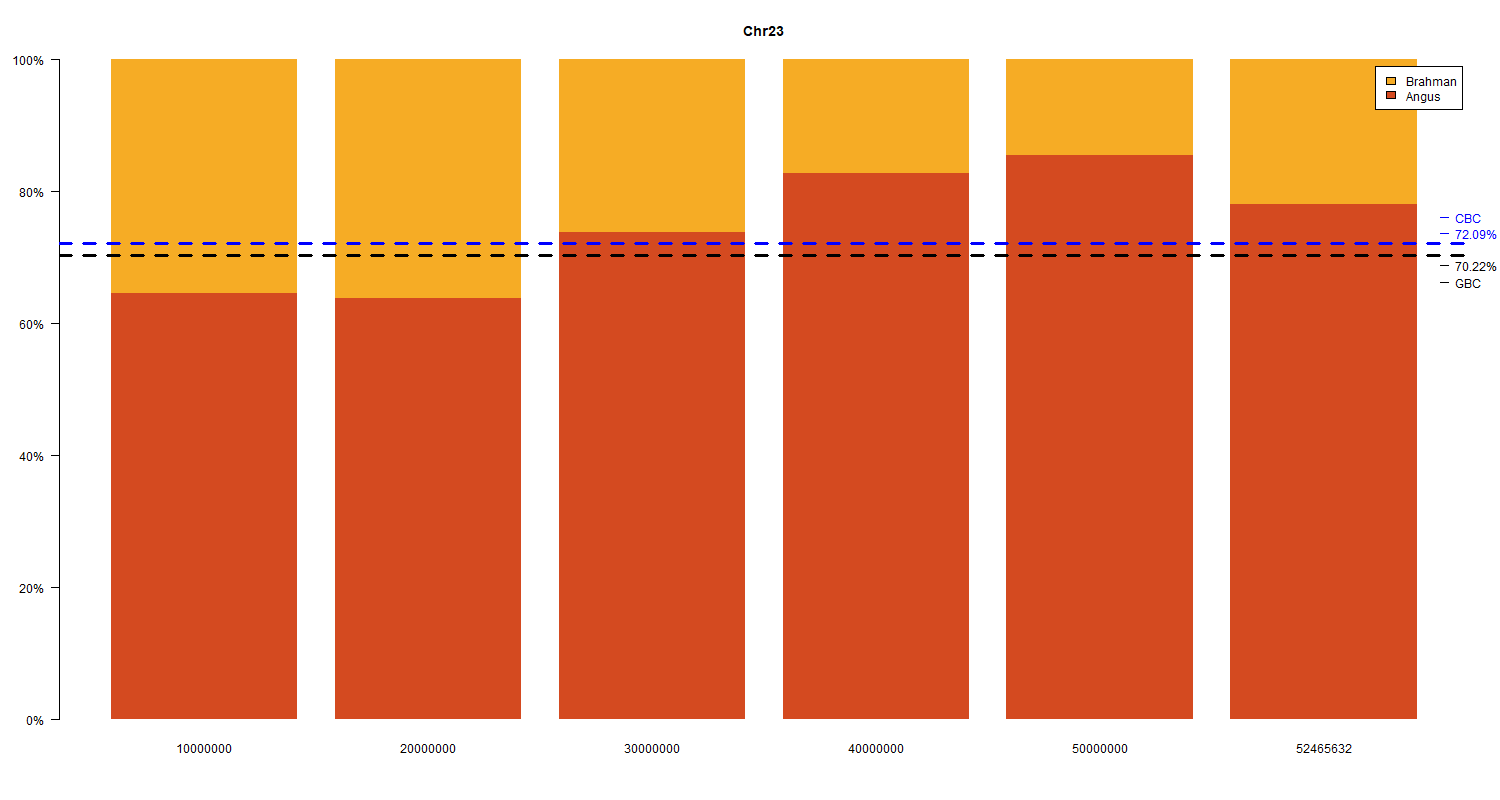

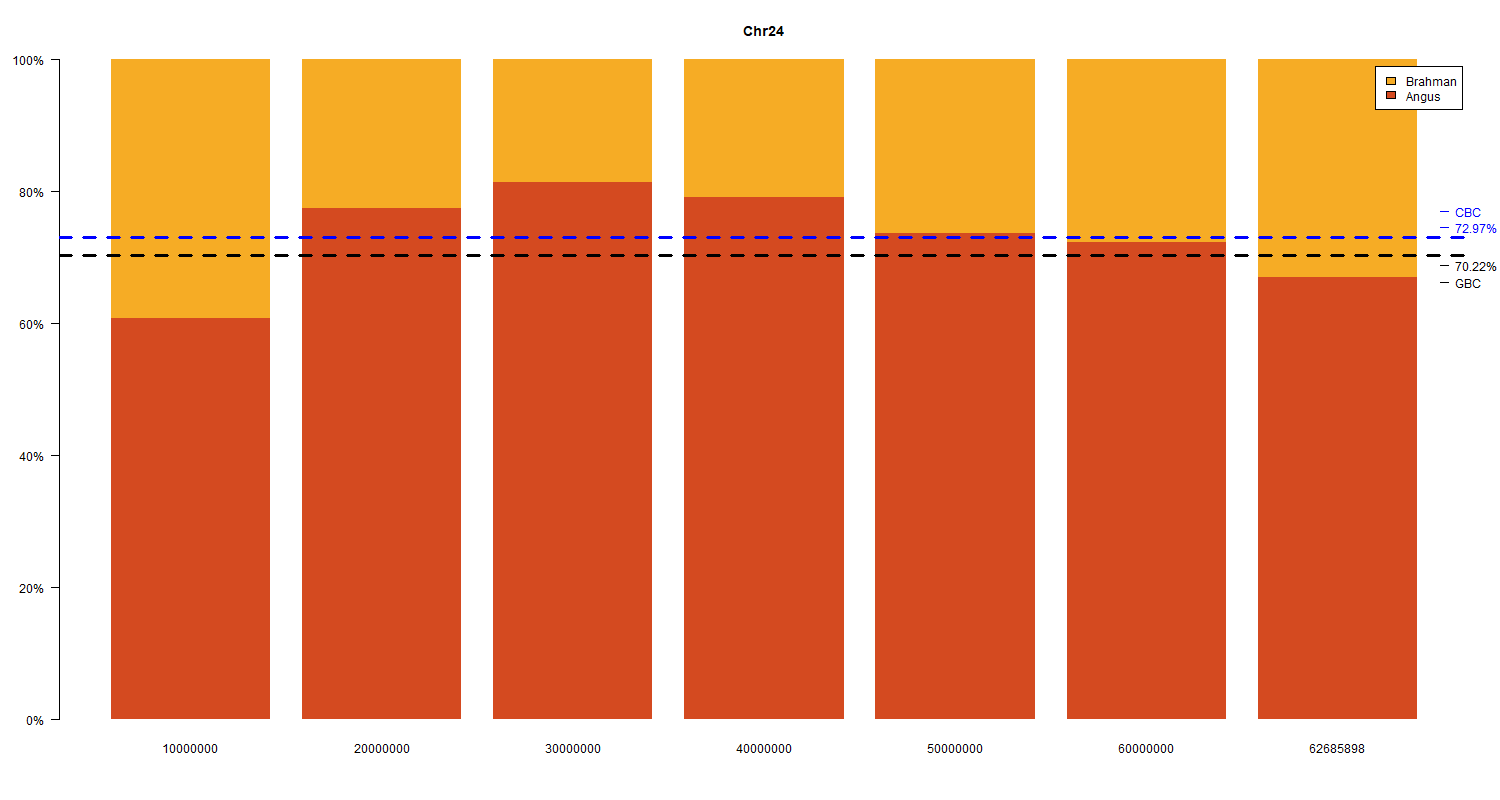

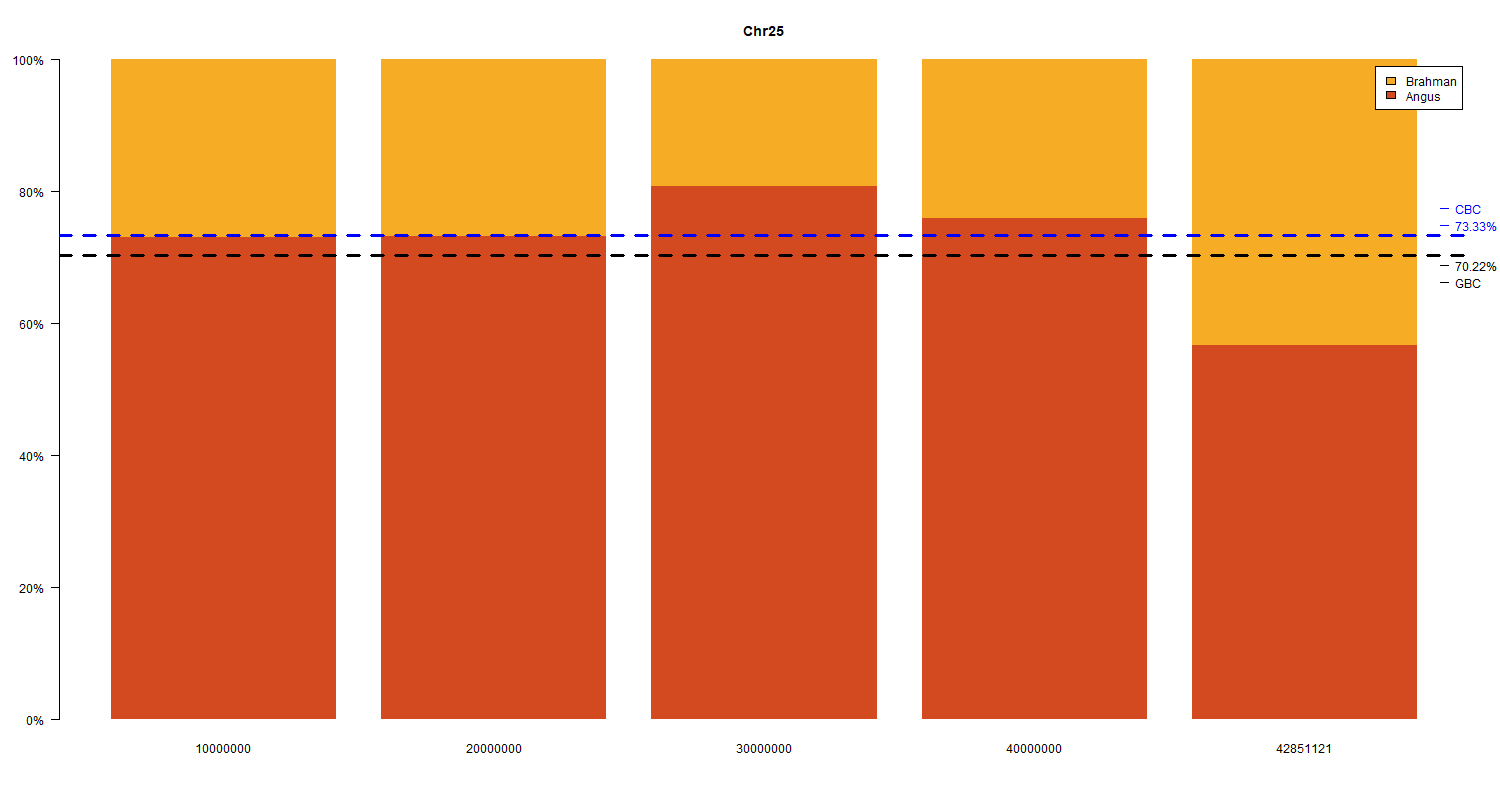

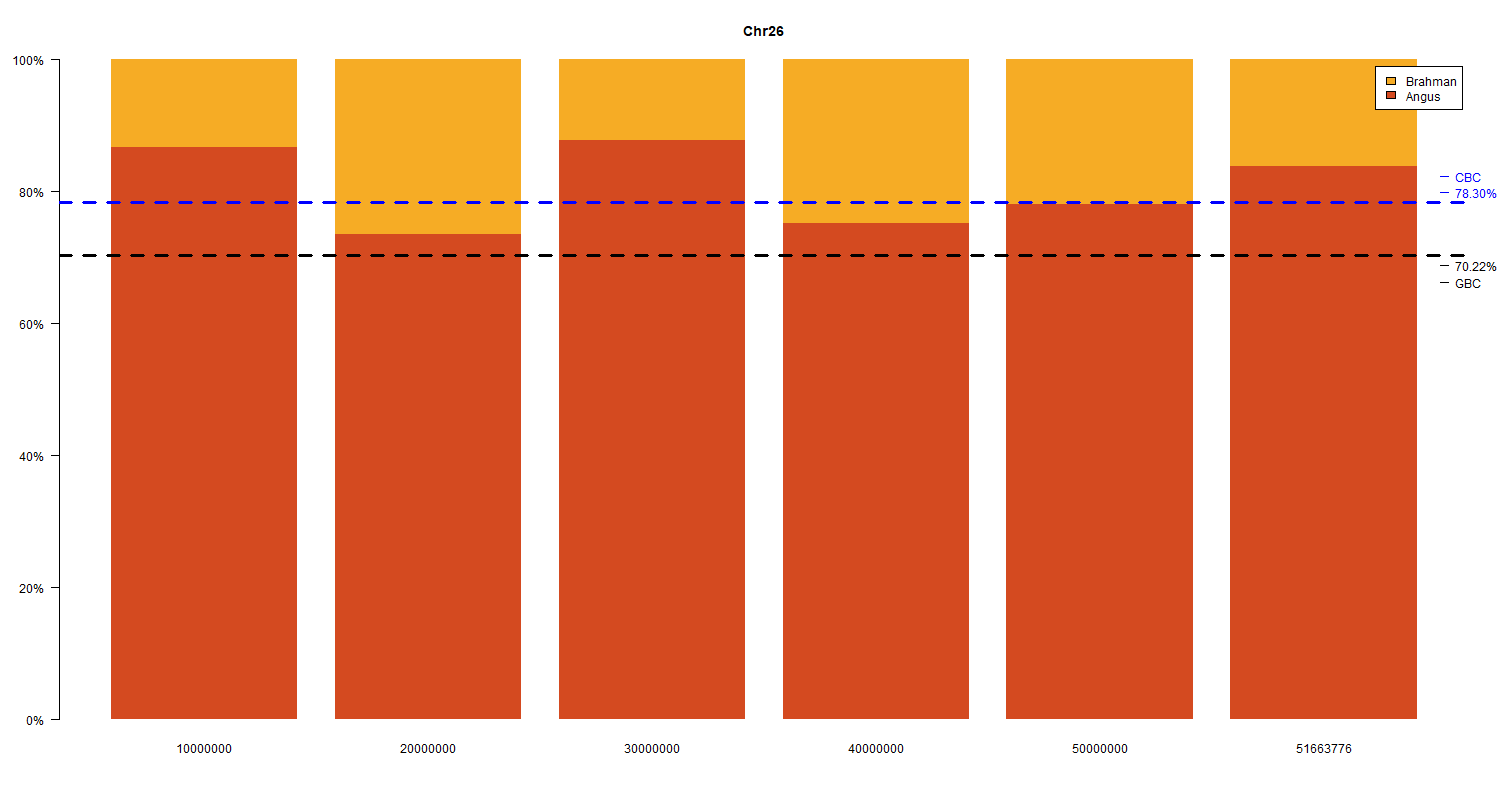

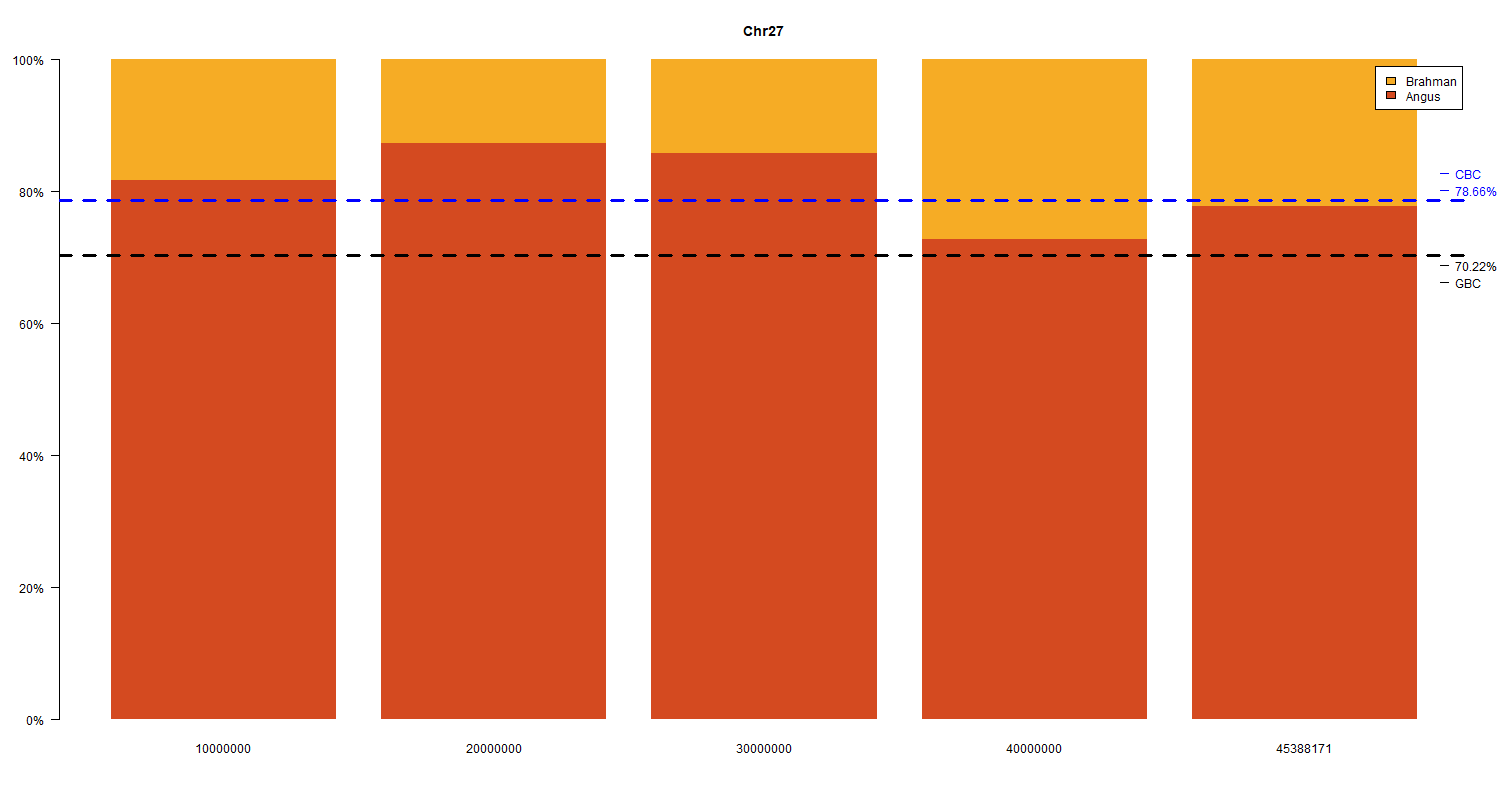

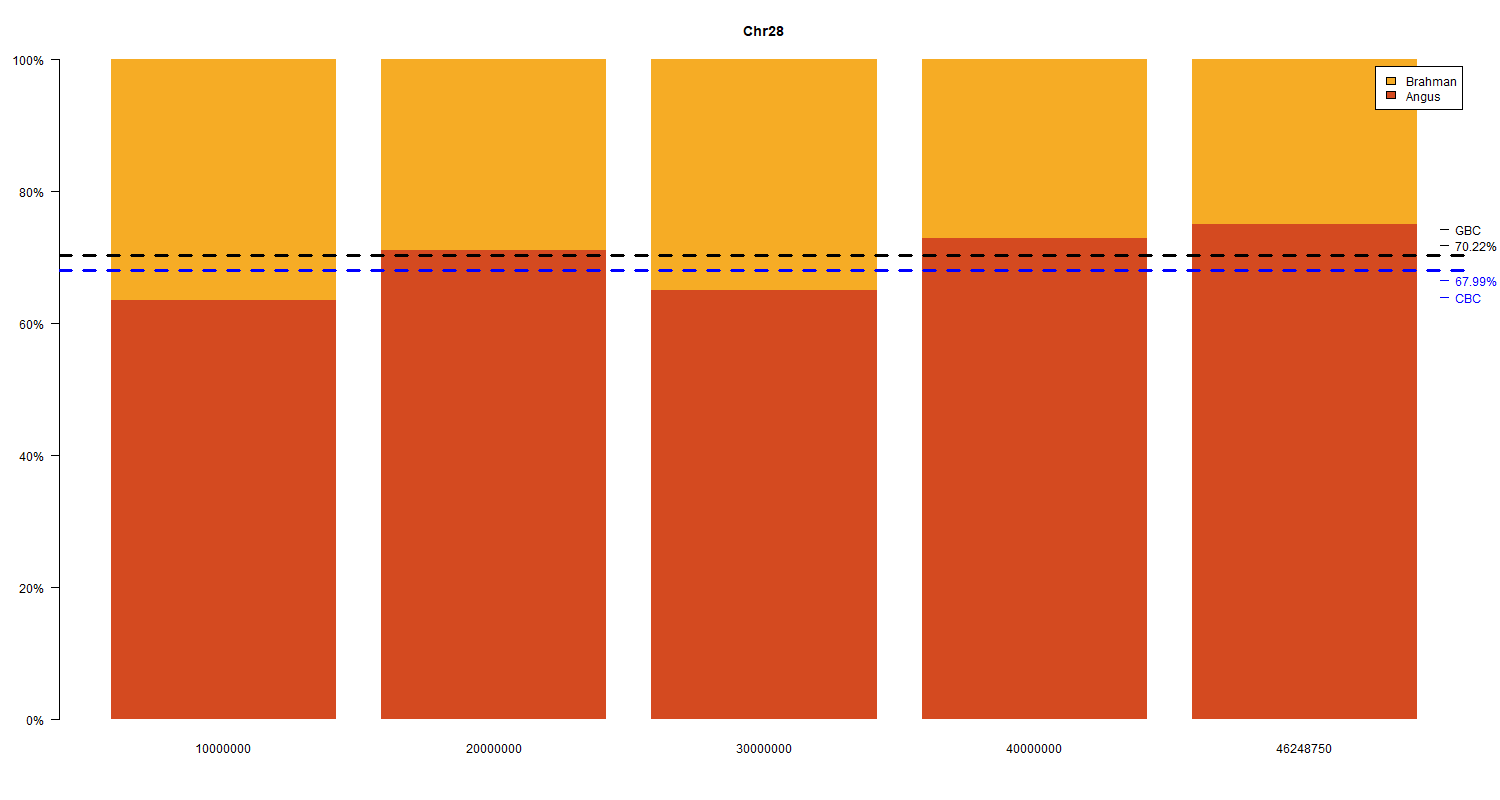

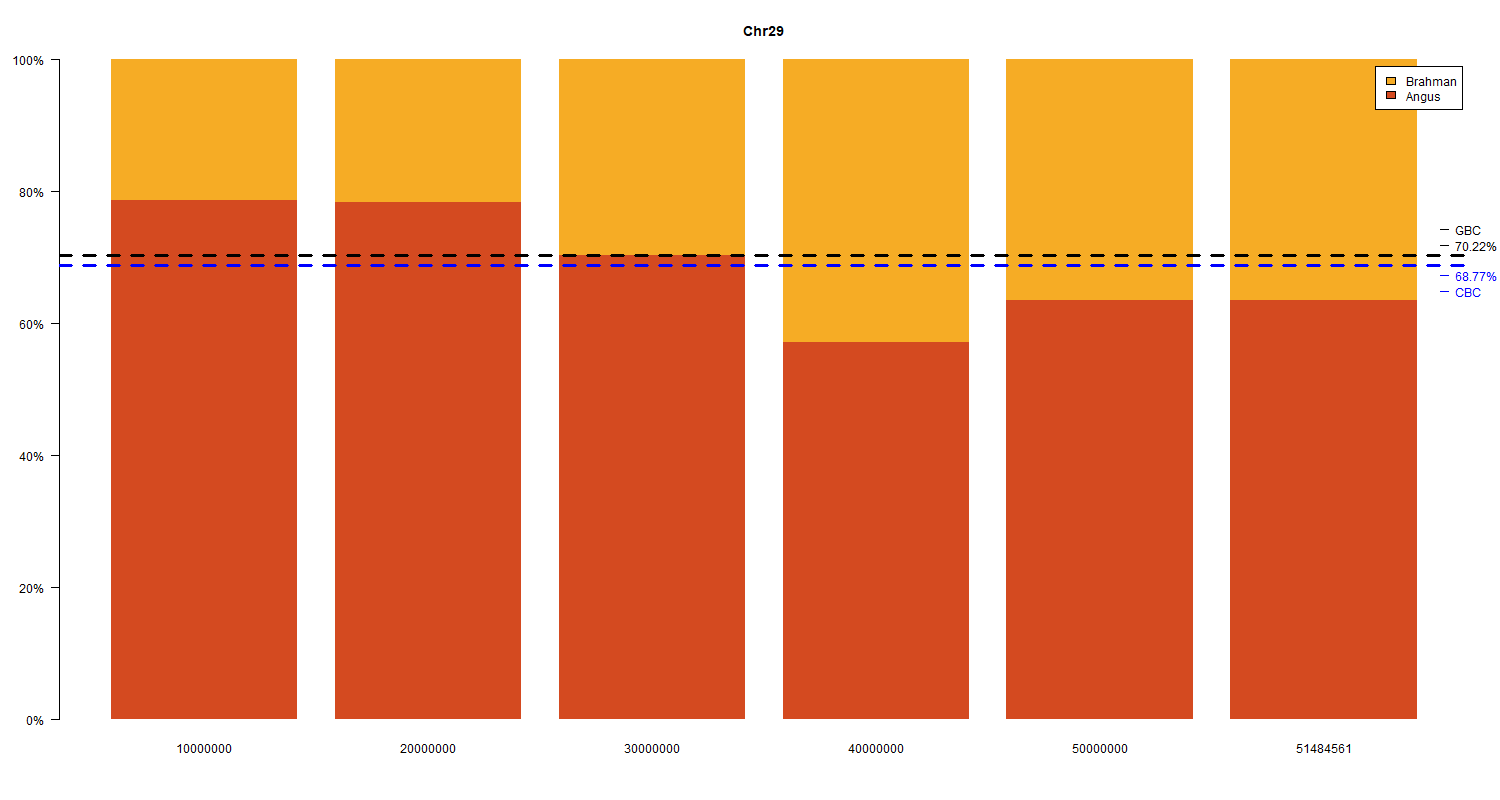


**Supplementary Figure S3.** Boxplot of the estimated Angus (red) and Braham (blue) breed proportions by three varying window sizes in 3,605 Brangus cattle: (A) window size = 1Mb; (B) window size = 5 Mb; (C) window size = 10 Mb. 1 = Angus breed proportion; 2 = Brahman breed proportion.

(A-1)


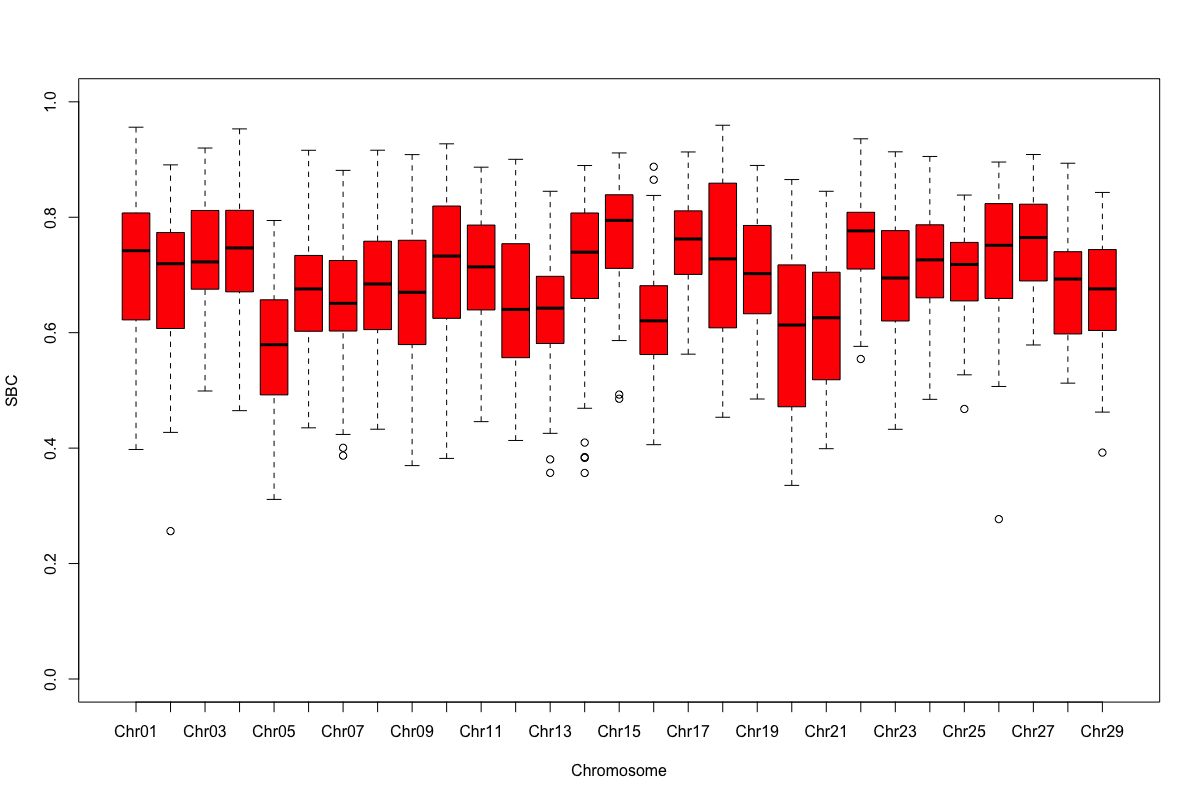


(A-2)


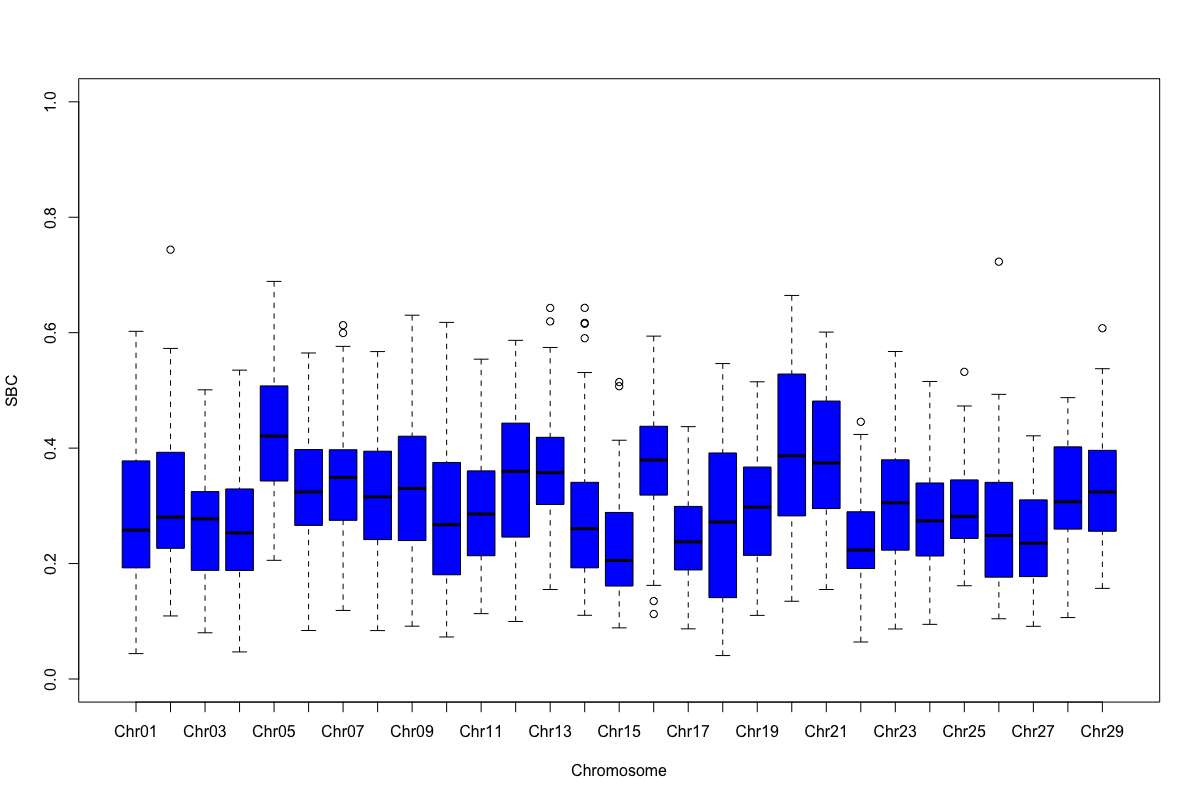


(B-1)


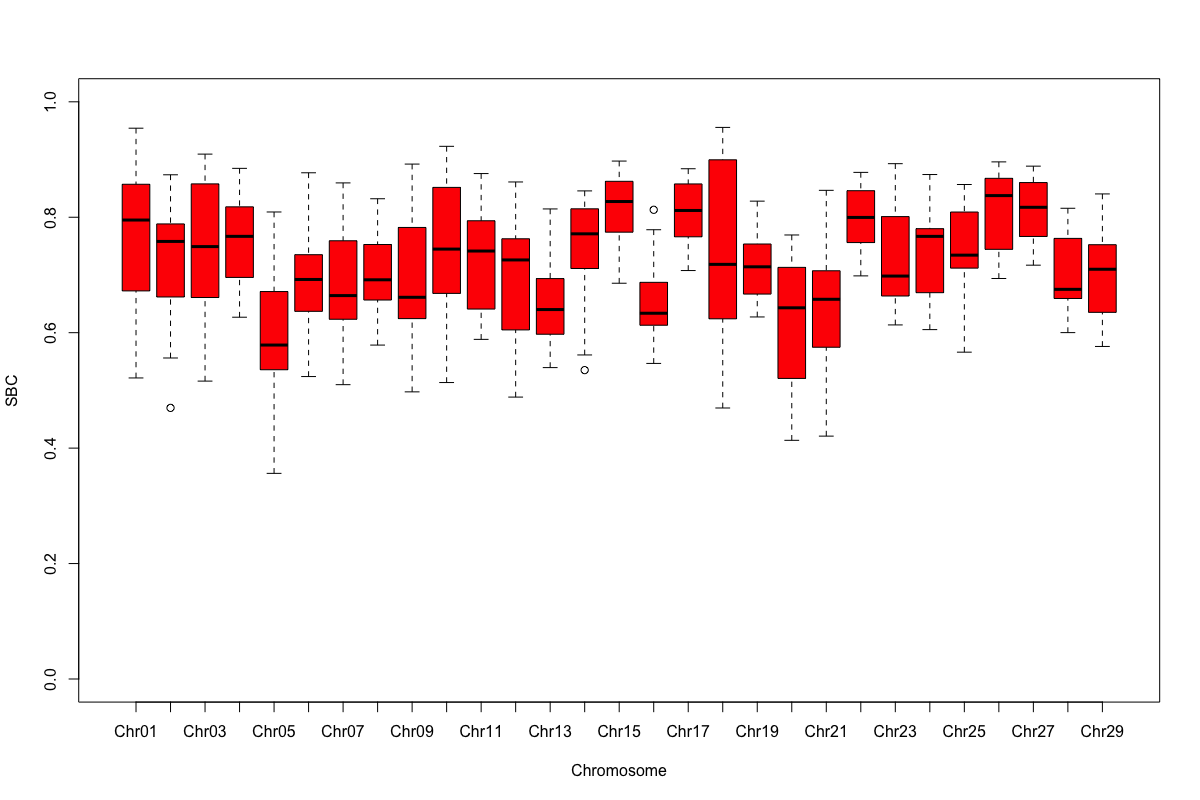


(B-2)


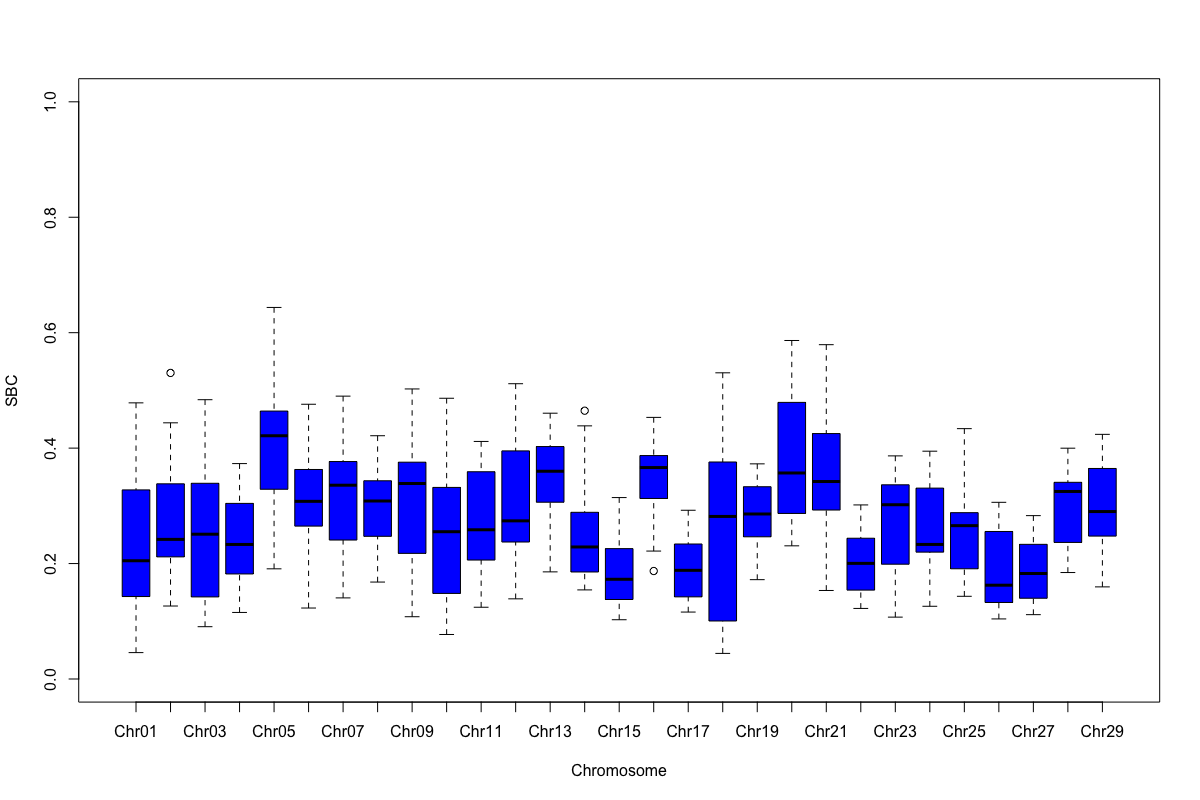


(C-1)


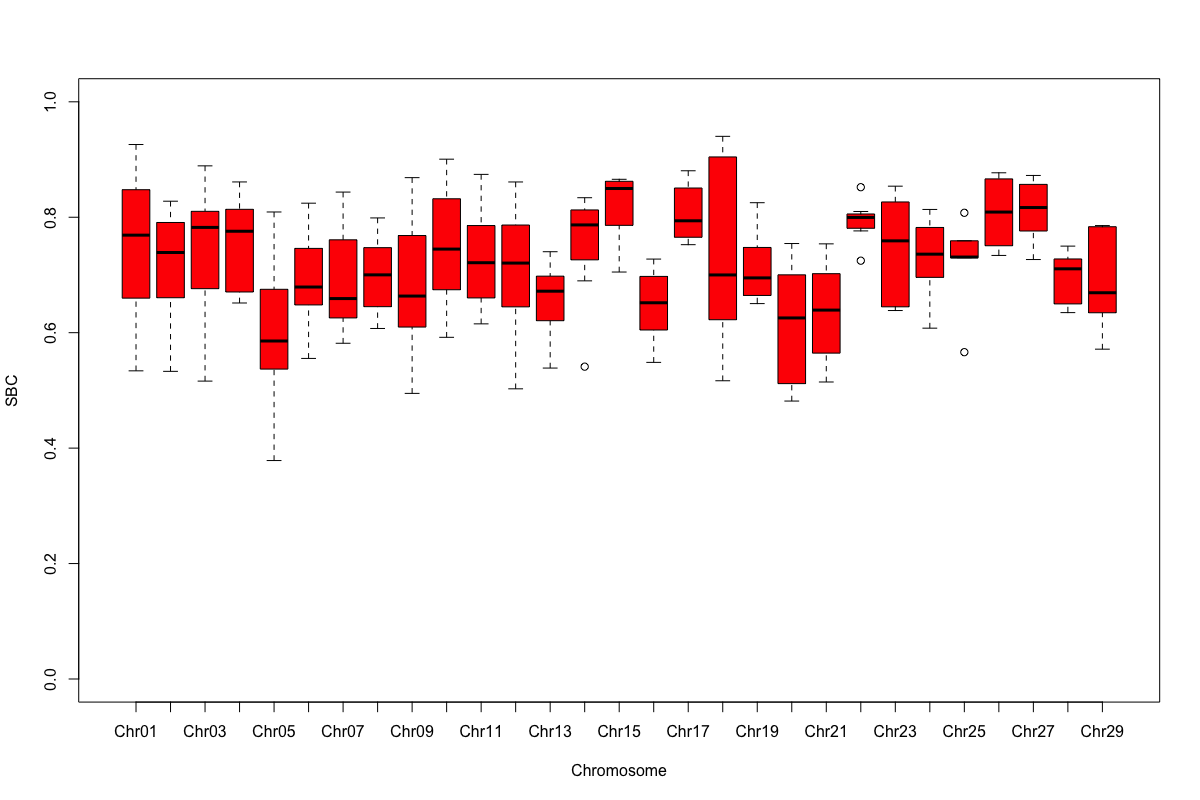


(C-2)


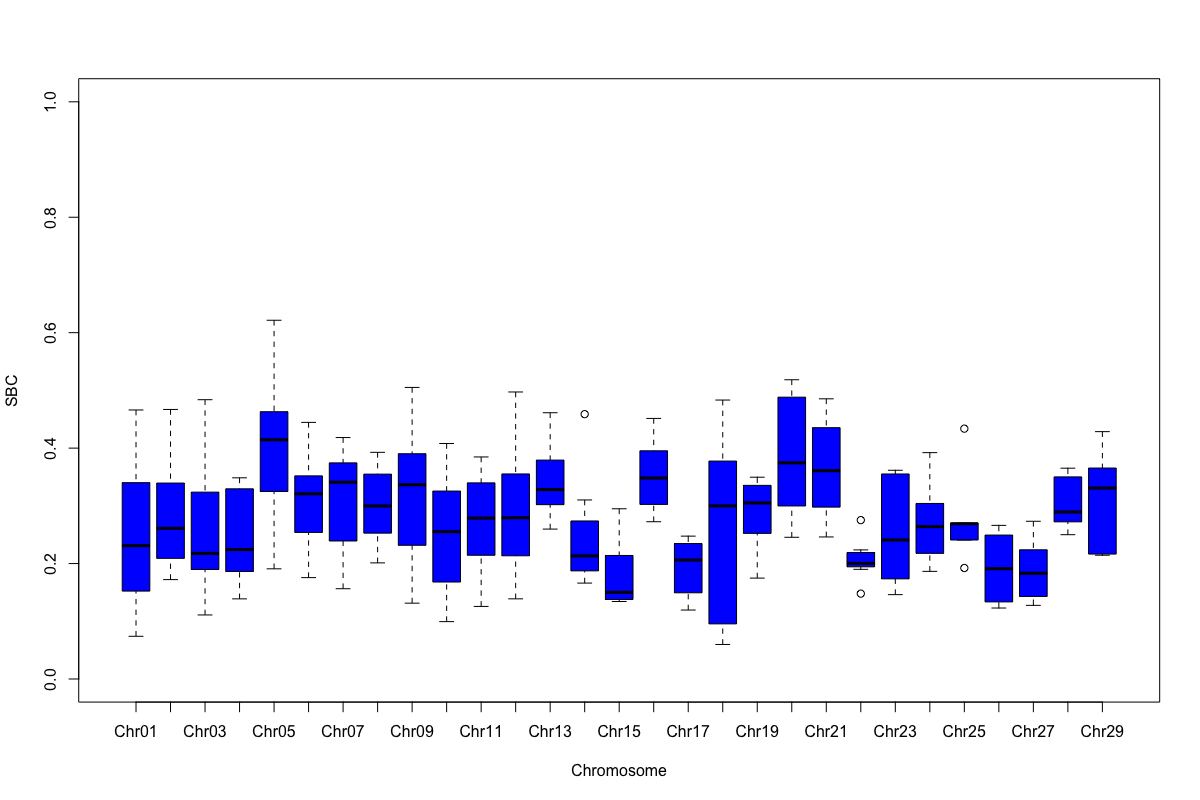

Supplement: Supplementary file 2 [file Table2.docx]
